# Structure dynamics of ApoA-I amyloidogenic variants in small HDL increase their ability to mediate cholesterol efflux

**DOI:** 10.1194/jlr.RA120000920

**Published:** 2020-11-24

**Authors:** Oktawia Nilsson, Mikaela Lindvall, Laura Obici, Simon Ekström, Jens O. Lagerstedt, Rita Del Giudice

**Affiliations:** 1 Department of Experimental Medical Science, Lund University, Lund, Sweden; 2 Amyloidosis Research & Treatment Centre, Fondazione IRCCS Policlinico San Matteo, Pavia, Italy; 3 BioMS - Swedish National Infrastructure for Biological Mass Spectrometry, Lund University, Lund, Sweden; 4 Lund Institute of Advanced Neutron and X-ray Science (LINXS), Lund, Sweden

**Keywords:** apolipoproteins, apolipoprotein A-I, amyloidosis, high density lipoprotein/HDL, cholesterol efflux, cardiovascular disease, protein structure, hydrogen-deuterium exchange/HDX, ABCA1, ATP-binding cassette A1, ABCG1, ATP-binding cassette G1, ApoA-I, apolipoprotein A-I, ApoB, apolipoprotein B, CD, circular dichroism, CVD, cardiovascular disease, FC, unesterified free cholesterol, HDL, high density lipoprotein, HDX, hydrogen-deuterium exchange, LCAT, lecithin-cholesteryl acyl transferase, PBS, phosphate buffer saline, POPC, 1-palmitoyl-2-oleoyl-sn-glycero-3-phosphocholine, rHDL, reconstituted HDL, SRCD, Synchrotron Radiation Circular Dichroism, TEV, tobacco etch virus, Tm, transition temperature, WT, wild-type

## Abstract

Apolipoprotein A-I (ApoA-I) of high density lipoproteins (HDLs) is essential for the transportation of cholesterol between peripheral tissues and the liver. However, specific mutations in ApoA-I of HDLs are responsible for a late-onset systemic amyloidosis, the pathological accumulation of protein fibrils in tissues and organs. Carriers of these mutations do not exhibit increased cardiovascular disease risk despite displaying reduced levels of ApoA-I/HDL cholesterol. To explain this paradox, we show that the HDL particle profiles of patients carrying either L75P or L174S ApoA-I amyloidogenic variants show a higher relative abundance of the 8.4-nm versus 9.6-nm particles and that serum from patients, as well as reconstituted 8.4- and 9.6-nm HDL particles (rHDL), possess increased capacity to catalyze cholesterol efflux from macrophages. Synchrotron radiation circular dichroism and hydrogen-deuterium exchange revealed that the variants in 8.4-nm rHDL have altered secondary structure composition and display a more flexible binding to lipids than their native counterpart. The reduced HDL cholesterol levels of patients carrying ApoA-I amyloidogenic variants are thus balanced by higher proportion of small, dense HDL particles, and better cholesterol efflux due to altered, region-specific protein structure dynamics.

Amyloidoses are a broad group of diseases caused by protein instability and misfolding that leads to pathological aggregation of proteins as amyloid deposits in tissues and organs (1). These amyloid deposits are either localized, as in Alzheimer's and Parkinson's diseases, or systemic, as is the case of transthyretin and light-chain amyloidoses. The different types of amyloidoses commonly lead to severe and age-related damage of the tissues where aggregation takes place. The understanding of the determinants leading to protein fibril formation and amyloidosis development is thus key for finding ways to develop efficient treatments for the affected individuals.

Apolipoprotein A-I (ApoA-I), associated with hereditary systemic amyloidosis, has a key role for the transportation of cholesterol and lipids in the circulation between peripheral tissues and the liver (2). Following its synthesis in the liver and intestines, ApoA-I mediates this transportation by the formation of high density lipoprotein (HDL) particles. These load cholesterol and lipids from macrophages at the artery wall via a regulated efflux mechanism catalyzed by the cellular ATP-binding cassette (ABC) A1 receptor. HDLs are then carried to the liver in the bloodstream for cholesterol processing and/or excretion as bile. This is an important process since well-balanced levels of cholesterol, particularly at the artery wall, may reduce the buildup of atherosclerotic plaques and hence the risk of cardiovascular disease (CVD). HDL particles are further modified by interaction with additional cellular receptors (ABCG1 and scavenger receptor BI (SR-BI)) and with soluble enzymes, including the lecithin-cholesteryl acyl transferase (LCAT) protein (3, 4). During these processes, the HDL particle grows from the initially formed discoidal pre-beta HDL species to larger, spherical HDL particles, a process that is facilitated by flexibility in the ApoA-I structure (5). The interaction with these receptors and soluble enzymes as well as the protein flexibility are critical for HDL biogenesis. The qualitative features and the size distribution of the HDL species, rather than the absolute quantity of ApoA-I/HDL, are indeed regarded as major determinants for function (6) and for the prevention of CVD (7).

A high degree of flexibility in the ApoA-I structure is thus important for lipid binding and functionality of ApoA-I in HDLs. However, in ApoA-I variants carrying single amino acid substitutions, this flexibility might be causative for increased susceptibility to proteolytic remodeling and subsequent protein aggregation. Indeed, this is the case for 23 currently known human amyloidogenic variants of the *APOA1* gene that lead to progressive accumulation of ApoA-I protein in the liver, heart, kidneys, larynx, skin, and/or testis (8, 9, 10, 11, 12). These mutations are localized to 2 major regions of the ApoA-I structure, either within residues 25 to 75 in the N-terminal domain or within residues 170 to 178 in the central domain of ApoA-I. The reason for the high occurrence of amyloid-prone variants in these 2 regions is not known, but this might indicate that these regions have specific functions in lipid association and/or in protein structure dynamics (13). Interestingly, carriers of several ApoA-I variants, including Gly26Arg (14), Leu75Pro (15, 16), and Leu174Ser (17, 18), have decreased blood levels of ApoA-I, yet do not have increased risk of CVD. Recently, *in vitro* cell studies using recombinant lipid-free and HDL-reconstituted ApoA-I amyloidogenic variants provided an explanation to this apparent paradox, as these variants showed a significantly higher cholesterol efflux capacity than the native protein (19). However, the structural basis for the improved catalytic function of the ApoA-I variants is not understood. It is also not clear how the complexity and HDL species distribution in circulation contribute to the improved efficacy of the variants. Here, we use clinical samples from Leu75Pro (L75P) and Leu174Ser (L174S) patients, as well as from matched control individuals, to investigate their unique HDL particle distribution and functionality. In order to explain the *in vivo* phenotype of the L75P and L174S variants, synchrotron radiation circular dichroism (SRCD), hydrogen-deuterium exchange (HDX) mass spectroscopy on reconstituted HDL (rHDL), and the measurement of cholesterol efflux from macrophages to both rHDL and patient serum samples are used to analyze the functionality and structure of discoidal pre-beta HDL particles of defined sizes.

## Materials and methods

### Serum samples from patients carrying ApoA-I amyloidogenic variants

Serum samples from patients with ApoA-I amyloidosis and from unrelated control subjects (11 controls, 11 L75P patients and 4 L174S patients, between 37 to 77 years of age, both female and male subjects) were obtained at the Amyloidosis Research and Treatment at Fondazione IRCCS Policlinico San Matteo. All patients consent to the use of their biological samples and anonymized data for research purposes on a dedicated consent form according to an institutional procedure that has been approved by the Ethics Committee of the study center.

### Apolipoprotein B depletion

Serum samples from patients carrying ApoA-I amyloidogenic variants and from control subjects were subjected to ApoB depletion prior to biochemical analyses and cholesterol efflux experiments on macrophages, as previously described (20). For this, 200 μl of serum was incubated with 80 μl of 20% polyethylene glycol (PEG) 6,000 in 20 mM glycine buffer at pH 7.4, for 20 min at 25°C, with gentle shaking. After incubation, serum samples were centrifuged at 10,000 rpm for 20 min at 4°C and supernatants were analyzed by human ApoA-I ELISA, as well as by denaturant and native Western blot.

### Determination of ApoA-I content in serum samples

**Fig. 1:**
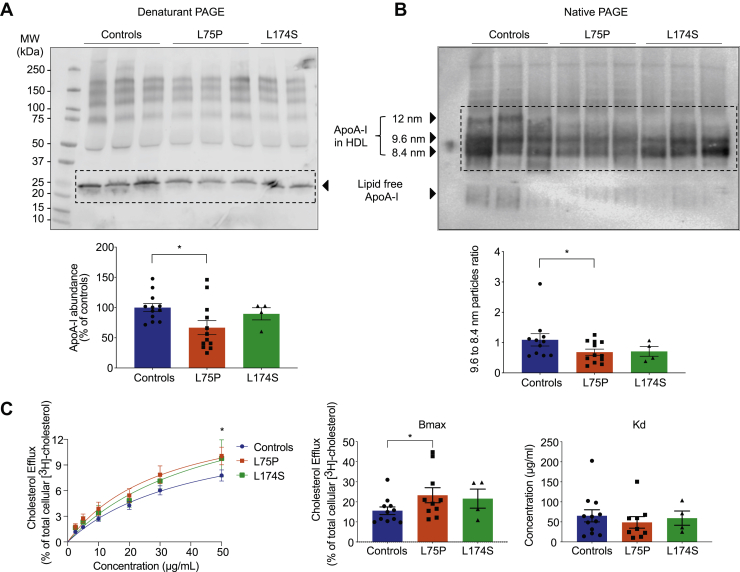
Quantitative and qualitative analysis of HDLs from patients with ApoA-I amyloidosis. (A) Patients carrying ApoA-I amyloidogenic variants possess reduced levels of plasma ApoA-I. Proteins from serum sample from patients and control subjects were separated by denaturant PAGE and analyzed by Western blot by using anti-human ApoA-I antibody (upper panel), and ApoA-I levels were quantified by using ImageJ software (lower panel). (B) Patients carrying ApoA-I amyloidogenic variants have a distinctive HDL pattern. Proteins from serum samples from patients and control subjects were separated by native PAGE and analyzed by Western blot by using anti-human ApoA-I antibody (upper panel). The ratio between the abundance of 9.6-nm and 8.4-nm particles was calculated and is displayed in the lower panel. (C) HDLs from patients show improved ability to promote cholesterol mobilization. Cholesterol efflux experiments were performed by using serum samples as acceptors of cholesterol, in dose-response experiments. Radioactive cholesterol-loaded J774 macrophages were treated with serum samples, normalized for the amount of total ApoA-I, and incubated for 4 h. The experimental data (left panel) were fitted, and Bmax (middle panel) and Kd (right panel) were calculated according to Michaelis-Menten equation. Data shown are the mean ± SEM, and significance is calculated according to two-way ANOVA (C, left panel, ∗*P* < 0.05) or one-way ANOVA (C, middle panel, ∗*P* < 0.05). N = 11 for L75P and controls, and N = 4 for L174S donors. Representative samples from 3 donors per group are shown in panels A and B.

Commercially available ApoA-I ELISA kit (MABTECH, 3710-1HP-2) was used to determine the ApoA-I content in serum samples from patients with ApoA-I amyloidosis and unrelated control subjects. In order to allow for correction of potential differences in detection efficiency, the ApoA-I antibody/ELISA was tested on known concentrations of purified recombinant ApoA-I variants, as well as on purified wild-type (WT) ApoA-I protein, and correctional factors were calculated and used. The corrected ApoA-I concentrations (variants and WT) agreed with the ApoA-I levels reported in Figure 1A.

### Western blot from native and denaturant gel electrophoresis

Serum samples were separated on 4%–15% Tris-Glycine precasted gels (BioRad), for the denaturant PAGE and on NativePAGE Bis-Tris Gel System 4%–16% (Invitrogen, Thermo Fisher Scientific), for the native PAGE, according to the manufacturer's instructions.

In both cases, serum proteins were transferred from the gel to PVDF membranes and probed with anti-human ApoA-I antibodies (64308, Abcam, for denaturant blot and Q0496, Dako, Agilent Technologies, for native blot). Detection was performed by using HRP-conjugated secondary antibodies (GE Healthcare) and a chemiluminescence detection substrate (Super-Signal West Femto, Thermo Fisher Scientific). Blots were imaged using the Odyssey Fc system (LI-COR Biosciences).

### Protein expression and purification

Human ApoA-I proteins, containing a His-tag and tobacco etch virus (TEV) protease recognition site at the N terminus, were expressed in the bacterial *Escherichia coli*) BL21(DE3) pLysS strain (Invitrogen, Thermo Fisher Scientific) as described in the study by Del Giudice and Lagerstedt (21).

Recombinant proteins were then purified using immobilized metal affinity chromatography (His-Trap-Nickel-chelating columns, GE Healthcare) followed by treatment with TEV protease and a second immobilized metal affinity chromatography step to remove the His-tag. TEV cleavage results in 2 additional amino acid residues (Ser and Leu) at the N terminus of the purified ApoA-I proteins. Protein samples were analyzed by SDS-PAGE followed by Coomassie staining, and concentration was determined by using a NanoDrop 2000c spectrophotometer (Thermo Fisher Scientific). As previously shown, the purity of the recombinant lipid-free ApoA-I proteins was about 80% as determined by mass spectrometry analysis and, importantly, none of the contaminant constituted more than 1% of total protein species (21).

### Preparation of reconstituted HDL

Lyophilized POPC (1-palmitoyl-2-oleoyl-sn-glycero-3-phosphocholine, Avanti Polar Lipids) and cholesterol (unesterified free cholesterol [FC]) (Avanti Polar Lipids) were dissolved in 3:1 chloroform-methanol, and the solvent was evaporated by overnight incubation under a stream of nitrogen gas. POPC and FC were dissolved in phosphate buffer saline (PBS), and lipoparticles were generated by using the cholate dialysis method (22). For this, POPC and POPC-FC lipoparticles were produced by incubating POPC and cholesterol, diluted in sodium deoxycholate, with ApoA-I variants at 80:4:1 or 40:2:1 M ratio (to produce 9.6- and 8.4-nm particles, respectively) and at a 1 mg/ml protein concentration. Mixtures were incubated at 37°C for 1 h and then dialyzed against PBS for 72 h. At the end of the incubation, homogeneous 9.6-nm and 8.4-nm POPC and POPC-FC-ApoA-I particles were isolated by size-exclusion chromatography by using a preparative Superose 6 increase 10/300 GL column (GE Healthcare). Samples were eluted at a flow rate of 0.5 ml/min, in PBS, and analyzed by Blue Native PAGE using the NativePAGE Bis-Tris Gel System 4%–16% (Invitrogen, Thermo Fisher Scientific) according to the manufacturer's instructions, flushed with nitrogen and stored at –80°C prior to experiments. The stability and integrity of the particles after freezing is shown in supplemental Figure S1.

### Cholesterol efflux from macrophages

Cholesterol efflux assay was performed as described in the study by Del Giudice *et al.* (19). J774 macrophages (TIB-67, ATCC) were plated into 24-well plates, in RPMI 1640 (Gibco) supplemented with 10% FBS and 50 μg/ml gentamicin, at a cell density of 150,000 cells/well. Twenty-four hours after the plating, cells were loaded with 4 μCi/ml ^3^H-cholesterol (Perkin Elmer) in RPMI 1640 containing 5% FBS, 2 μg/ml ACAT inhibitor (inhibits formation of cholesteryl esters) (Sandoz 58-035, Sigma), and gentamicin. Upon 24-h incubation, the medium was replaced with RPMI 1640 supplemented with 0.2% BSA (low free fatty acids and low endotoxin, Sigma), 2 μg/ml ACAT inhibitor, 0.3 mM Cpt-cAMP (promotes expression of ABCA1) (Abcam), and gentamicin for 18 h. At the end of incubation, cells were washed twice with serum-free RPMI 1640 and then triplicate wells were treated with either ApoA-I amyloidogenic variants or the WT protein in POPC particles, or serum from patients and control subjects, in RPMI 1640 supplemented with 0.2% BSA, at the indicated concentrations and for 4 h. Cholesterol efflux was measured by collecting the media, centrifuging at 4,000× *g* for 5 min at room temperature, and transferring of 100 μl of supernatant to a scintillation vial. Five milliliters of scintillation fluid was added to each sample before scintillation counting was performed. The measure of the total cellular ^3^H-cholesterol was obtained by incubating the cells, in triplicate, with 1% sodium deoxycholate and lysates collected for scintillation counting. Efflux for each treatment was calculated as % of the total ^3^H-cholesterol. Spontaneous basal efflux was measured in triplicate and the efflux for each treatment subtracted for this value. GraphPad Prism software was used to fit the efflux experimental data according to the Michaelis-Menten equation and to calculate Kd and Bmax values.

### Synchrotron radiation circular dichroism

SRCD experiments were performed using a nitrogen-flushed Module-A end-station spectrophotometer, equipped with a 6-cell turret, at B23 Beamline at the Diamond Light Source (23, 24, 25). POPC particles were produced in McIlvaine buffer, pH 7, and analyzed by SRCD at 0.15 mg/ml in a 0.2-mm quartz cuvette. Spectra were acquired at 25°C in the far-UV range 185–260 nm, with 1-nm wavelength increment. All the spectra were corrected by subtracting the background signal of the buffer. Secondary structure estimations from CD spectra were carried out using the software CD Apps (26) and applying CONTILLN algorithm with reference data SP 43 (27). The molar ellipticity ([Θ]) was calculated according to the equation described in the study by Brouillette *et al*. (28).

### Hydrogen-deuterium exchange mass spectrometry

Two different preparations of 8.4-nm POPC-ApoA-I particles containing WT, L75P, or L174S were analyzed by HDX and data combined for the final HDX analysis. All chemicals for the HDX-MS analyses were purchased from Sigma Aldrich, except n-dodecyl-b-D-maltopyranoside which was from Thermo Scientific. pH measurements were made using a SevenCompact pH meter equipped with an InLab Micro electrode (Mettler-Toledo), and prior to all measurements, a 4-point calibration (pH 2, 4, 7, 10) was performed.

The ApoA-I particles were placed in the autosampler in such a way that no sample had a permanence in the machine for longer than 12 h. The HDX-MS analysis was performed using automated sample preparation on a LEAP H/D-X PAL™ platform interfaced to an LC-MS system, comprising an Ultimate 3,000 micro-LC coupled to an Orbitrap Q Exactive Plus MS. For the HDX-MS, 5 μl of POPC:ApoA-I particles was diluted either in 25 μl of PBS, pH 7.4 or in HDX labeling buffer (PBS prepared in D_2_O, pH_(read)_ 7.0) and the HDX reactions were carried out for *t* = 0, 30, 300, 3,000, and 9,000 s at 8°C. At the end of incubation, labeling was quenched by adding 25 μl of 1% TFA, 0.2% n-dodecyl-b-D-maltopyranoside, 4 M urea, pH 2.5 at 1°C, to the samples. Then, 50 μl of the quenched sample (50 pmol protein/injection) were directly subjected to online pepsin digestion at 4°C, by injection on a pepsin column (2.1 × 30 mm, Life Technologies). In order to remove lipids from the samples, the pepsin column was directly followed by a 2 × 20 mm guard column (Upchurch Scientific) packed with a washed and equilibrated ZrO_2_ material (Sigma Aldrich, Zirconium IV oxide, powder, <5 micron). The online digestion and trapping were performed for 4 min using a flow of 50 μL/min 0.1% formic acid (FA), pH 2.5. Peptides generated by pepsin digestion were subjected to online SPE on a PepMap300 C18 trap column (1 mm × 15 mm) and washed with 0.1% FA for 60 s. Thereafter, the trap column was switched inline with a reversed-phase analytical column, Hypersil GOLD, particle size 1.9 μm, 1 × 50 mm, and separation was performed at 1°C using a gradient of 5% to 50% B over 8 min and then from 50% to 90% B for 5 min, the mobile phases were 0.1% FA (A) and 95% acetonitrile/0.1% FA (B). Following separation, the trap and column were equilibrated at 5% organic content, until the next injection. The needle port and sample loop were cleaned 3 times after each injection with mobile phase 5% MeOH/0.1% FA, followed by 90% MeOH/0.1% FA, and a final wash of 5% MeOH/0.1% FA. After each sample and blank injection, the pepsin column was washed by injecting 90 μl of pepsin wash solution 1% FA /4 M urea /5% MeOH. In order to minimize carryover, a full blank was run between each sample injection. Separated peptides were analyzed on a Q Exactive Plus MS, equipped with a heated electrospray ionization source operated at a capillary temperature of 250°C. For the undeuterated samples (*t* = 0 s), injections were acquired using data-dependent MS/MS HCD for identification of generated peptides. For the HDX analysis (all labeled samples and 1 *t* = 0 s), MS full-scan spectra at a setting of 70 K resolution, AGC 3e6, Max IT 200 ms, and scan range of 300–2000 were collected.

PEAKS Studio 8.5 from Bioinformatics Solutions Inc. (BSI, Waterloo, Canada) was used for peptide identification after pepsin digestion of undeuterated samples (i.e., 0 s timepoint). The search was done on a FASTA file with only the 3 different ApoA-I sequences; search criteria included a mass error tolerance of 15 ppm and a fragment mass error tolerance of 0.05 Da, oxidation of methionine (15.99 Da) as variable modification and allowing for fully unspecific cleavage by pepsin.

Peptides identified by PEAKS with a peptide score value of log *P* > 25 and no oxidation were used to generate a peptide list containing peptide sequences, charge state, and retention time for the HDX analysis. HDX data analysis and visualization were performed using HDExaminer, version 3.01 (Sierra Analytics Inc., Modesto, CA). Due to the comparative nature of the measurements, the deuterium incorporation levels for the peptic peptides were derived from the observed mass difference between the deuterated and nondeuterated peptides without back-exchange correction using a fully deuterated sample. HDX data were normalized to 100% D_2_O content with an estimated average deuterium recovery of 75%. The peptide deuteration level is the average of all high and medium confidence results, and the 2 first residues assumed unable to hold deuteration. The allowed retention time window was ±0.5 min. Heatmaps settings were uncolored proline, heavy smoothing, and the difference heatmaps were drawn using the residual plot as a significance criterion (±0.5 Da). The data were first analyzed allowing for unimodal HDX kinetics (EX2) only. Since previous studies have described bimodal HDX kinetics for regions of ApoA-I in HDL particles (29), data were also analyzed allowing the software to try an EX1 deuteration envelope if the EX2 score was lower than 0.9 and to accept the result if the score increased ≥0.05. The spectra for all timepoints were manually inspected; low-scoring peptides, outliers, and peptides for which retention time correction could not be made consistent were removed.

A summary of all the HDX experimental detail is reported in supplemental Table S1.

### Thermal stability analyses

CD spectroscopy measurements were performed on a Jasco J-810 spectropolarimeter equipped with a Jasco CDF-426S Peltier. POPC particles (0.1 mg/ml ApoA-I in particle) were diluted in PBS and loaded into a 1-mm quartz cuvette, and CD signal at 220 nm was acquired in the 20–98°C range, with a 2°C increment and a heating rate of 60°C/h. The estimation of the transition temperature (Tm) was performed by biphasic fitting using GraphPad Prism software.

A PCR thermal cycler (TC-Plus, Techne) was used to denature POPC particles with the same temperature increment and incubation times as in the thermal unfolding at the CD spectrometer. Samples were taken at 20, 65, 85, and 98°C and analyzed by native electrophoretic analysis (2.5 μg of ApoA-I per lane) followed by western blot with anti-human ApoA-I antibodies. The quantification of the species visualized on the gel was performed by using ImageJ software. Specifically, the rectangular selection tool was used to outline different species present in the different gel lanes; afterwards, the software generated a lane profile plots from which the density of each protein species was determined from each peak. The amounts of lipid free protein, intact HDL and fused HDL (generated as a consequence of lipid-protein dissociation and association during the heating process (30) was plotted as fold change with respect to the total signal.

### Statistical analysis

Data shown are the mean ± SD or ± SEM, as indicated. Analysis was performed by one- or two-way ANOVA, as indicated, using the GraphPad Prism software. Outliers were identified using GraphPad outlier test (alpha = 0.05).

## Results

### Patients carrying ApoA-I amyloidogenic variants have distinctive HDL patterns

Serum samples from patients carrying ApoA-I amyloidogenic variants (heterozygotic for either L75P or L174S) and from unrelated control subjects were first depleted for apolipoprotein B (potential cholesterol acceptor) prior to the analyses below in order to reduce potential variability in background cholesterol efflux. To examine the levels and profile of HDL species, equal volumes of serum samples were separated by denaturant and native PAGE and analyzed by Western blot for ApoA-I protein content by using anti-human ApoA-I antibodies. Serum from L75P heterozygotic patients presented lower amounts of total ApoA-I protein (33% reduction compared with control subjects, Fig. 1A), which agrees with previous reports (16, 17, 31), whereas ApoA-I levels in serum of L174S patients were similar to those of control subjects. Analysis of the samples by native Western blot supported this observation and also revealed differences in the distribution of HDL species (Fig. 1B). Specifically, serum from control subjects displayed an additional species of particles around 12 nm and also a higher relative abundance of the 9.6-nm particles in relation to the 8.4-nm particles (9.6:8.4-nm particle ratio is 1.09) compared with serum from L75P and L174S patients (ratios of 0.57 and 0.71, respectively) (Fig. 1B).

### HDL from patients carrying ApoA-I amyloidogenic variants show improved ability to promote cholesterol mobilization

Reconstituted 9.6-nm HDL particles containing ApoA-I variants have been shown to be more efficient than the WT counterpart at mediating cholesterol efflux from macrophages (19). In order to test if HDLs from patients carrying L75P and L174S amyloidogenic variants possess an improved ability to mediate cholesterol mobilization, dose-response cholesterol efflux experiments were performed by using serum samples as acceptors of cholesterol. Cholesterol-loaded J774 macrophages were incubated (4 h) with serum samples from L75P and L174S patients, or from control subjects, in a dose-dependent manner normalized for the amount of total ApoA-I. Spontaneous cholesterol release into the media was measured, and the efflux for each treatment was subtracted for this value.

As shown in Figure 1C, patients with the L75P substitution displayed a 33% increase in cholesterol binding capacity (Bmax) as compared with control subjects (*P* < 0.05) but with no difference in binding affinity (Kd). A similar increase in the Bmax for L174S amyloidogenic variant was observed but did not reach statistical significance for the low number of clinical samples available for this variant. Interestingly, when cholesterol efflux data were plotted according to donor's gender (supplemental Fig. S2), data suggested that the improved efflux capacity observed for the amyloidogenic ApoA-I patients is sex-specific with male patients showing the larger increase in Bmax.

In order to evaluate if the relatively higher serum concentrations of 8.4-nm HDL particles (compared with the 9.6-nm HDL particles) contributed to the improved cholesterol mobilization, cholesterol efflux experiments were performed by using reconstituted HDL (rHDL; POPC-ApoA-I) particles with sizes of either 8.4 or 9.6 nm. The effect of FC already being present in the synthesized rHDL particles (POPC-FC-ApoA-I) was also evaluated.

**Fig. 2:**
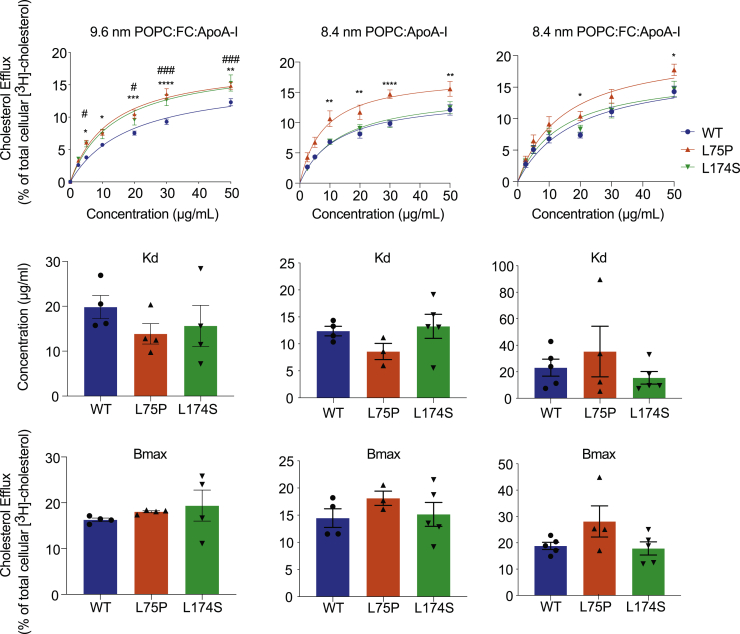
Cholesterol efflux ability of reconstituted HDLs of different sizes. 9.6- and 8.4-nm reconstituted HDLs (rHDLs) were produced by incubating recombinant ApoA-I amyloidogenic variants with POPC, in the presence or absence of cholesterol, and tested for their ability to mediate cholesterol efflux from J744 macrophages, in dose-response experiments. The experimental data (upper panel) were fitted, and Kd (middle panel) and Bmax (lower panel) were calculated according to Michaelis-Menten equation. Data shown are the mean ± SEM, and significance is calculated according to two-way ANOVA (∗*P* < 0.05, ∗∗*P* < 0.01, ∗∗∗*P* < 0.001, ∗∗∗∗*P* < 0.0001, and ##*P* < 0.01, ###*P* < 0.001 for L75P and L174S rHDL, respectively, as compared with WT rHDL). N = 4–5.

In general, several of the rHDL particles containing the amyloidogenic variants displayed greater ability to promote cholesterol efflux than WT ApoA-I (Fig. 2). In particular, 9.6-nm POPC-FC-ApoA-I particles containing either L75P or L174S ApoA-I protein were characterized by a higher cholesterol efflux capacity (from 20% to 30% higher cholesterol efflux at all concentrations tested; Fig. 2, top panels) than WT rHDL. Similarly, it was previously observed that 9.6-nm POPC-ApoA-I rHDL (i.e, rHDL synthesized without cholesterol) containing either L75P or L174S amyloidogenic variant showed improved cholesterol efflux ability, however, in that case due to a higher affinity to cholesterol (19). Interestingly, in the case of 8.4-nm rHDL, only the particles containing the L75P variant showed an increased cholesterol binding capacity. This increase was particularly pronounced for L75P rHDL particles synthesized without FC (POPC-ApoA-I), which were also characterized by a higher cholesterol binding affinity (lower Kd) than WT and L174S-containing rHDL. These observations suggest that the improved efflux ability of HDL from L75P patients (Fig. 1C) is due to the higher levels of 8.4-nm particles compared with controls. However, as the impact of functional variability in other HDL subspecies could not be excluded, we next compared the structure and function of the 8.4-nm HDL particles specifically.

### ApoA-I amyloidogenic variants in 8.4-nm particles are characterized by a higher structural flexibility

**Fig. 3:**
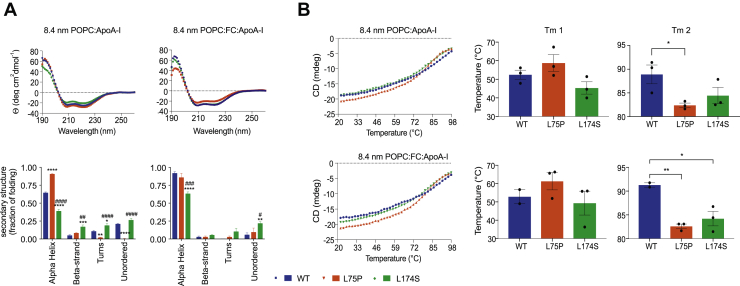
Conformation and stability of ApoA-I amyloidogenic variants in 8.4-nm rHDLs. (A) SRCD spectroscopy analysis of ApoA-I variants in 8.4-nm rHDL particles, in the presence or absence of FC. SRCD spectra (upper panels) were deconvoluted with the CONTINLL algorithm for the estimation of the ApoA-I proteins' secondary structure (lower panels). (B) Thermal stability of ApoA-I variants in 8.4-nm rHDL particles, in the presence or absence of FC. The unfolding curves (left panels) were obtained by monitoring the CD signal amplitude at 222 nm as a function of temperature, and Tm (right panels) were calculated by fitting the experimental data with a biphasic nonlinear regression. Data shown are the mean ± SEM, and significance is calculated according to (A) two-way ANOVA (∗*P* < 0.05, ∗∗*P* < 0.01, ∗∗∗*P* < 0.001, ∗∗∗∗*P* < 0.0001, and ##*P* < 0.01, ###*P* < 0.001 ####*P* < 0.0001 for L75P and L174S rHDL, respectively, as compared with WT rHDL, or (B) 1-way ANOVA (∗*P* < 0.05, ∗∗*P* < 0.01). Data shown are the mean ± SEM. N = 3.

Far-UV CD spectroscopy was used to analyze protein conformation and stability of the ApoA-I variants, as well as the WT protein, in 8.4-nm rHDL. The estimation of the secondary structure of the proteins in rHDL, performed by synchrotron radiation CD, revealed interesting differences in the folding of the amyloidogenic variants and the native protein (Fig. 3A). In the absence of FC (POPC-ApoA-I), the L75P variant in 8.4-nm rHDL particles showed a remarkably high content of α-helices (90% for L75P compared with 64% for WT) and a low content of turns and unordered structures. The L174S variant, instead, was characterized by a lower percentage of α-helical structure (39%) accompanied by higher proportions of turns, beta-strands and unordered structures. In the presence of FC, the amounts of α-helical structure of both WT and L174S in 8.4-nm rHDLs were increased compared with the non-FC particles; however, the L174S α-helical content was still significantly lower than that for both WT and L75P.

ApoA-I proteins in 8.4-nm rHDL were next tested for their thermal stability by monitoring changes in the CD signal at 222 nm. As already reported for 9.6-nm POPC-ApoA-I particles (32), thermal denaturation of the rHDL particles follows a biphasic unfolding process, showing 2 transition temperatures (Tm). These are considered to reflect structural rearrangement of the protein (Tm1) and dissociation of protein from lipids (Tm2). As shown in Fig. 3B, the 2 ApoA-I variants showed similar Tm1 when compared with the WT (middle panels) but were both characterized by lower Tm2 (right panels), suggesting a higher protein flexibility and/or a lower affinity for lipids. Of note, the thermal denaturation profiles appeared to be unaffected by the presence of FC, suggesting that cholesterol does not contribute to the thermal stability of neither the ApoA-I protein nor the protein-lipid association in rHDLs.

**Fig. 4:**
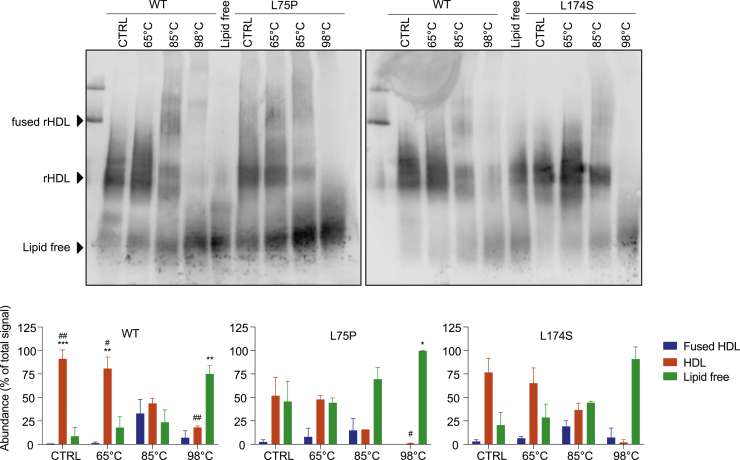
Temperature-induced unfolding and lipid dissociation of ApoA-I amyloidogenic variants in 8.4-nm rHDLs. 8.4-nm rHDL particles, in the absence of FC, were incubated at 20°C, 65°C, 85°C, and 98°C, separated by native PAGE and analyzed by Western blot by using anti-human ApoA-I antibody (upper panels). Lipid-free ApoA-I was used as the control. The species obtained upon thermal denaturation were quantified, and the amount of each species was expressed as percentage with respect to the total signal (lower panels). Data shown are the mean ± SD, and significance is calculated according to two-way ANOVA (∗*P* < 0.05, ∗∗*P*< 0.01, ∗∗∗*P* < 0.001 for groups as shown respect to fused HDL, #*P* < 0.05, ##*P* < 0.01 for groups as show respect to lipid-free protein). N = 2 (L75P and L174S rHDL) or 4 (WT rHDL).

To further explore the thermal denaturation process and the species formed, 8.4-nm POPC-ApoA-I rHDLs were incubated at 20°C, 65°C (temperature above Tm1), 85°C (slightly above Tm2 for the variants but below Tm2 of the WT), and 98°C (temperature at the end of the thermal unfolding process and clearly above Tm2 for all 3 samples). The collected samples were then separated by native PAGE and analyzed by Western blot (Fig. 4). Both ApoA-I variants were characterized by higher amounts of the lipid-free species at equilibrium (20°C), which was particularly pronounced in the case of the L75P variant (5.3-fold higher level of lipid-free L75P protein than WT). The relative abundances of lipid-bound and lipid-free species remained unchanged upon heating at 65°C. At 85°C, the L75P sample showed the highest amount of lipid-free species, followed by the L174S sample, in agreement with the observed lower Tm2 (Fig. 3B). At the end of the thermal denaturation process (98°C), almost all of the L75P and L174S proteins were in their lipid-free states, whereas a significant amount of the WT protein (18%) was still associated with lipids. The more pronounced dissociation of the variants from lipids and the lower temperature at which this occurred indicate that the ApoA-I variants in rHDLs are characterized by a much higher flexibility than the WT protein.

**Fig. 5:**
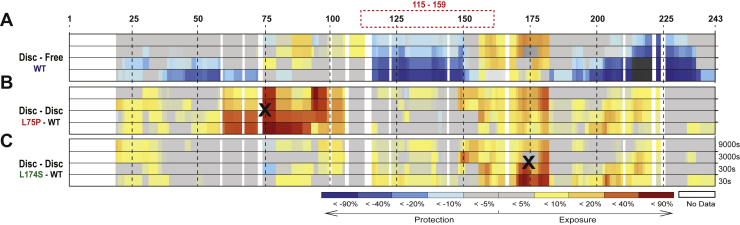
Hydrogen-deuterium exchange mass spectrometry reveals regions with high flexibility in 8.4-nm rHDLs. Differential HDX heatmap of (A) lipid-bound vs lipid-free states for WT ApoA-I, (B) L75P versus WT in 8.4-nm POPC particles, and (C) L174S versus WT in 8.4-nm POPC particles. HDX peptide coverage is shown by the bars above each heatmap. The heatmaps show the deuterium uptake at the different timepoints (30 s, 300 s, 3,000 s, and 9,000 s). Cold color = slower exchange; warm color = faster exchange. The red dashed box (top) surrounding 115–159 sequence denotes the region where bimodal HDX kinetics (EX1) could be observed for the rHDL particles. Amino acidic substitutions are indicated with a black X.

SRCD and thermal denaturation provide information on the global protein secondary structure, as well as on protein stability and flexibility, but do not describe which protein regions or domains are specifically affected by the L75P and L174S substitutions. To address this, HDX-MS was used to analyze domain-specific protein flexibility of the ApoA-I proteins in 8.4-nm rHDLs. The differential heatmaps between the variants and the WT rHDL (Fig. 5B, C) indicated an overall increase in deuterium uptake for both the ApoA-I amyloidogenic variants (heatmaps for the individual proteins are shown in supplemental Fig. S3). Notably, both L75P and L174S variants showed region-specific structural flexibility, with the L75P variant presenting a stronger destabilizing effect in the regions close to the mutation site (50–105 region), whereas the L174S variant was characterized by a decrease in stability in the 126–140 and 170–180 regions (deuterium uptake for all the peptides used in the analysis are shown in supplemental Data File S1). Furthermore, the L75P variant presented a more flexible N-terminal domain in rHDL, particularly in the region 50–75, where no significant difference between the WT and L174S rHDL could be detected (Fig. 5C), whereas the L174S showed a higher exchange rate in the 170–180 region at the shortest deuterium exposure time (Fig. 5B).

## Discussion

Subjects affected by ApoA-I hereditary systemic amyloidosis are characterized by low levels of serum ApoA-I and HDLs; however, although they also present reduced levels of HDL cholesterol, patients do not show higher risk of developing CVD (15, 16, 17, 18). We previously showed that ApoA-I amyloidogenic variants in reconstituted 9.6-nm HDL particles exhibit improved cholesterol efflux capability, and we therefore hypothesized that this enhanced functionality could serve to compensate the unfavorable lipid profile of amyloidogenic ApoA-I carriers (19). The present study translates these findings and verifies that the amyloidogenic ApoA-I variant L75P serum from human patients displays improved cholesterol efflux activity (Fig. 1C). A similar trend for the L174S variant was observed but was not statistically significant, possibly due to the low number of available serum samples from L174S patients.

This study also establishes that the ApoA-I amyloidogenic variants L75P and L174S present unique patterns in HDL particle distribution in serum from the patients. In particular, the level of smaller HDL particles (8.4-nm as compared with 9.6-nm particles) is higher in L75P and L174S patients than in the controls, and the larger 12-nm particles were relatively more abundant in the controls (Fig. 1B). HDL particle size has been shown to be a critical determinant of ABCA1-mediated cholesterol efflux in macrophages, with small dense particles being the most efficient mediators (6, 33).

The findings here described are in line with previous reports on the R173C and R151C variants, also known as ApoA-I Milano and Paris, respectively, whose carriers display lower levels of plasma ApoA-I and HDL, without showing increased risk of CDV (34, 35). In particular, ApoA-I Milano gives rise to smaller particles than the WT counterpart, characterized by an additional HDL subclass with an estimated diameter of 7.8 nm, particularly efficient at mediating cholesterol efflux *in vitro* (36).

The shift toward a larger proportion of small, dense particles in serum from patients could therefore potentially provide the explanation for the observed improvement in cholesterol efflux. However, a contributing role of the amino acid substitutions of the variants *per se* could not be ruled out. To investigate this possibility, we compared the functionality of small, dense, and variant-specific rHDL particles with defined sizes and protein/lipid compositions. The fact that the cholesterol efflux to the 9.6-nm rHDL acceptor particles was higher for both variants than the WT ApoA-I rHDL particles clearly indicated that the substitution at either residue 75 or 174 has a direct impact on protein/rHDL functionality. Interestingly, in 8.4-nm rHDL particles, only the L75P variant showed elevated capacity for cholesterol efflux, and this difference appeared to be particularly significant for particles reconstituted without free cholesterol. The data thus indicate that the L75P and L174S substitutions affect, at least partly, different molecular transitions/mechanisms and that this occurs in a particle-size-dependent manner. This may not be surprising, as ApoA-I is likely to adopt specific conformations in HDL particles with different sizes (5). Similarly, since ApoA-I proteins showed differences in functionality between the cholesterol/noncholesterol particles, ApoA-I may adopt specific conformations that also depend on the HDL lipid composition.

The organization of the primary structure into amphipathic alpha helices is a key feature in the lipid binding process of the ApoA-I protein. The L75P variant in 8.4-nm rHDLs, without free cholesterol, was shown to have a significantly larger proportion of alpha helices than both L174S and WT rHDL particles (Fig. 3A, left column). The high alpha helical content of the L75P protein in 8.4-nm rHDLs may thus indicate a readiness of the protein to accept cholesterol and that preloading of the particles with cholesterol (Fig. 3A, right column) triggers structural transitions to higher proportions of alpha helical secondary structure also in the L174S and WT proteins. Considering the central role of ApoA-I amphipathic alpha helices in HDL particle formation, the high level of beta-strand/turns and unordered structure in the L174S variant in 8.4-nm rHDLs, in particular the cholesterol-free particles, is intriguing. How the characteristic structural organization of L174S in 8.4-nm HDLs relates to protein function, and also to the amyloidogenic propensity of L174S protein, is not clear, but may partly explain the observed differences in cholesterol efflux capacity of the 2 variants (Fig. 2; 8.4 nm rHDL), as well as the reduced phospholipid binding capacity of lipid-free L174S (19).

We also found that the overall stability of the variants in 8.4-nm rHDL particles is not affected (Tm1 in Fig. 3B), although their interactions with lipids are weaker than that observed for the WT protein (Tm2 in Fig. 3B). This finding indicates that the amyloidogenic amino acid substitutions impose regional changes in protein backbone flexibility without substantially affecting the structural elements that contribute keeping the integrity of the entire protein. These conclusions are further supported by the qualitative analysis of rHDL integrity, which shows that the variants, in particular the L75P variant, are more prone to dissociate from the phospholipids/cholesterol of the lipid-protein complexes. A similar behavior has also been described for the Milano variant, for which a weaker interaction between lipids and lower HDL thermal stability has been reported (32, 37).

Sequence analysis of the ApoA-I protein described 1 globular domain (residues 1–43) followed by consecutive alpha helices (h1 to h10) with lengths of 11 or 22 amino acids (38). In the discoidal HDL particles, 2 ApoA-I molecules are organized in an antiparallel fashion (double belt) with an h5-h5 interaction between the 2 monomers (39). The L75P and the L174S substitutions are in the centers of h2 and h7, respectively. The h2 and h7 helices from the 2 ApoA-I monomers in discoidal HDL particles are partially overlapping, which brings residues 75 and 174 in relatively close proximity in the HDL particles. Moreover, a proline at position 75 is likely to create a kink that effectively breaks the 22mer h2 helix into 2 shorter 11mer helices, i.e., the same length as h3 and h9.

**Fig. 6:**
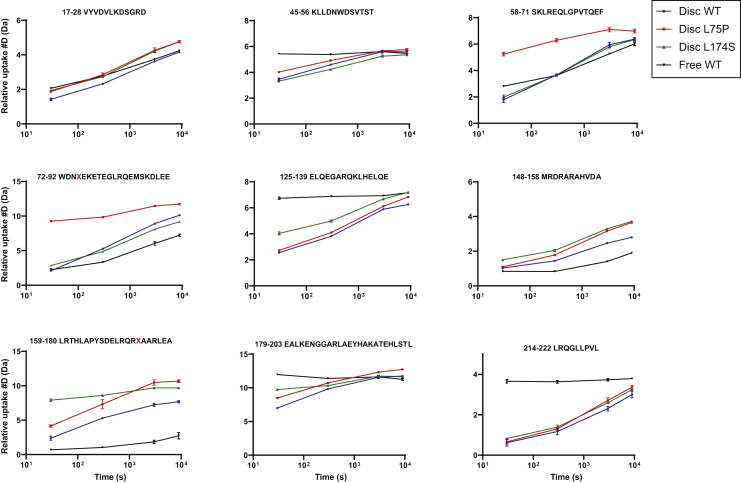
Deuterium uptake of selected ApoA-I peptides. Individual deuterium uptake plots for ApoA-I were the amino acidic substitutions led to a decreased structural stability compared with WT rHDL state (blue trace). Deuterium uptake for peptides from the lipid-free WT protein (black trace) is also reported as comparison. Amino acidic substitutions are indicated with a red X in the sequence of relevant plots. Data shown are the mean ± SD. N = 2. Mass envelopes, mass, and retention time difference for point mutations can be found in the supplementary information file (supplemental Fig. S5).

HDX-MS has previously been successfully applied for the study of the ApoA-I dynamics in rHDL (29, 40, 41, 42). In their recent publication, Wilson *et al*. (29) performed HDX on both lipid-free and lipid-bound forms of 3 ApoA-I variants (F71Y, L159R, and L170P). Although these variants were studied in rHDL particles with a different size (11–12 nm for F71Y, L159R, and L170P vs 8.4 nm for L75P and L174S) and lipid composition (DMPC vs POPC), and despite the fact that comparing HDX data is inherently complicated (43), the present study describes common trends with previous HDX studies. Indeed, a general reduction in uptake when going from lipid-free to lipid-bound form was observed (Fig. 5), as well as a decreased protection of the region spanning the h5 pair (as in L159R and L170P variants). Moreover, an increase in uptake for regions that are protected in the lipid-free protein (71–110 and 148–180) was also observed (Figs. 5A, and 6, and supplemental Figs. S4 and S5).

In agreement with previous reports, bimodal HDX kinetics (EX1) (44, 45), indicative of 2 conformational populations, was observed in the region spanning 115–159 (41). The observed EX1 behavior was mainly limited to short uptake times, where an increase of the fast exchanging populations could be observed for both variants (L75P and L174S) compared with WT. Although the point mutations affect the structural dynamics of the rHDL particles, and some differences were also observed in bimodal kinetics of the variants, an in-depth analysis of the origin and functional implications is beyond the scope of the current work. Examples of the observed bimodal kinetics can be found in the supplementary information (supplemental Fig. S6, S7 and S8). Nevertheless, regardless of the applied HDX analysis mode (unimodel or bimodal), the HDX-MS analysis of the 8.4-nm rHDL particles (Fig. 5) shows a specific increase in backbone flexibility in the 55 to 89 region for the L75P variant and in the 170–178 region for the L174S variant (Fig. 6) and a general increase in solvent accessibility for the variants compared with the WT protein in 8.4-nm rHDL particles (Fig. 5). Overall, the variants provide a more dynamic protein-lipid interaction that appears to be beneficial for their function.

The HDX-MS analysis of L75P and L174S in rHDL showed that deuterium uptake increases in regions that are already relatively flexible in the WT protein. Uptake is greatly increased at the mutation site, but an increase in deuteration in the h5/5 region and a broadening of solvent exposure toward the 4/6 helices could also be observed.

It appears that substitutions that break or weaken the alpha-helix hydrophobic face and/or salt bridges of the ApoA-I double belt in HDL particles result in an increase in solvent exposure as well as in flexibility of the protein. It is reasonable to assume that this will affect the lipid dynamics and, hence, both the binding to and the ability to mediate cholesterol efflux. In a recent paper, Manthei *et al.* (46) present a convincing model that indicates the h4/6 as the site for LCAT-HDL interaction, a site in close proximity to the dynamic h5/5 region. Thus, it is possible to speculate that the affinity of the interaction between LCAT and HDL might change as a consequence of the increased and expanded flexibility around the 4/6 helices observed in ApoA-I amyloidogenic variants in rHDLs.

The significance of the findings is not fully clear but may indicate that structural relaxation of this region improves the ApoA-I function as a cholesterol acceptor.

The increased mobility of the C-terminal domain is responsible for a faster binding to lipids in the case of ApoA-I Milano and Paris variants, as proposed by Gursky *et al*. (47). Interestingly, the authors also propose that the spacing between the C-terminal segments of these nonpathogenic variants during the homodimer formation is restricted, as compared with the WT protein, and allows the sequestration of smaller amounts of lipids, probably giving rise to smaller HDL particles.

In conclusion, we have shown that the 2 amyloidogenic amino acid substitutions L75P and L174S lead to increased local structure flexibility, which in turn affects the cholesterol efflux capacity in a positive manner. The importance of understanding the mechanistic details in cholesterol efflux is of general interest and extends beyond explaining the variant-specific differences. For example, it has been shown that impaired cholesterol efflux capacity, due to low (48) and dysfunctional (49, 50, 51, 52) HDL cholesterol, may be an important mediator of immunoactivation (51, 52) and subsequent CVD in chronic kidney disease (53, 54). Moreover, HDL subpopulation distribution and particle size has been shown to play a role in people with coronary heart disease with or without diabetes (54). The same study concludes that abnormal particle distribution and particle size may contribute to higher risk of developing coronary heart disease in patients with diabetes (54). Therefore, finding solutions to improve (or restore) HDL's functionality is of interest. This could potentially be done by introducing destabilizing factors that lead to greater region-specific flexibility in the ApoA-I structure. However, in such endeavors, care should be taken to not increase the amyloidogenic potential of the ApoA-I structure.

### Data availability

All data are contained in the manuscript or the supplementary files. The mass spectrometry proteomics data have been deposited to the ProteomeXchange Consortium via the PRIDE (55) partner repository with the dataset identifier PXD021266.
