# Supplementary material for: Structure dynamics of ApoA-I amyloidogenic variants in small HDL increase their ability to mediate cholesterol efflux
Source: J Lipid Res. 2020 Nov 24;62:100004. doi: 10.1194/jlr.RA120000920 (PMC7890215; doi:10.1194/jlr.RA120000920)
Supplement: Supplemental Table and Figures [file mmc1.pdf]

Supplementary information to:

Structure dynamics of ApoA-I amyloidogenic variants in small HDL increase their ability to mediate cholesterol efflux

Oktawia Nilsson[1], Mikaela Lindvall[1], Laura Obici[2], Simon Ekström[3], Jens O. Lagerstedt[1,4,*],

Rita Del Giudice[1,*,#]

[1] *Department of Experimental Medical Science, Lund University, SE-221 84 Lund, Sweden;* [2] *Amyloidosis Research & Treatment Centre, Fondazione IRCCS Policlinico San Matteo, Pavia, 27100, Italy;* [3] *BioMS - Swedish National Infrastructure for Biological Mass Spectrometry, Lund University, SE-221 84 Lund, Sweden;* [4] *Lund Institute of Advanced Neutron and X-ray Science (LINXS), SE-221 84 Lund, Sweden*

* Corresponding authors:

Jens O Lagerstedt; jens.lagerstedt@med.lu.se

Rita Del Giudice, rita.del_giudice@med.lu.se

[#] Author's current address: Malmö University, Department of Biomedical Science, 214 32 Malmö, Sweden. Email: rita.del-giudice@mau.se

Data included in this file are Supplementary Table S1 and Figures and Legends (S1-S8)

Supplemental Table S1: summary of the HDX experimental data

Data Set	Nanodiscs-WT	Nanodiscs-L75P	Nanodiscs-L174S	Lipid free-WT
HDX reaction details	0.01 M phosphate buffered saline (NaCl 0.138 M; KCl - 0.0027 M) prepared in D_2O, pH_{read} = 7.018, 25 °C			
HDX time course (s)	30, 300, 3000 and 9000			
HDX control samples	N.A. (relative comparison)			
Back-exchange (mean / IQR)	N.A (no 100% exchange control only relative comparison)			
# of Peptides	150			
Sequence coverage	93%			
Average peptide length / Redundancy	14.5 / 8.9			
Replicates (technical)	2			
Repeatability (Da)	0.0704 *	0.0882 *	0.0440 *	0.0753 *
Significant differences in HDX	0.50 D (95% CI)			

* average standard deviation

Supplemental Figure S1

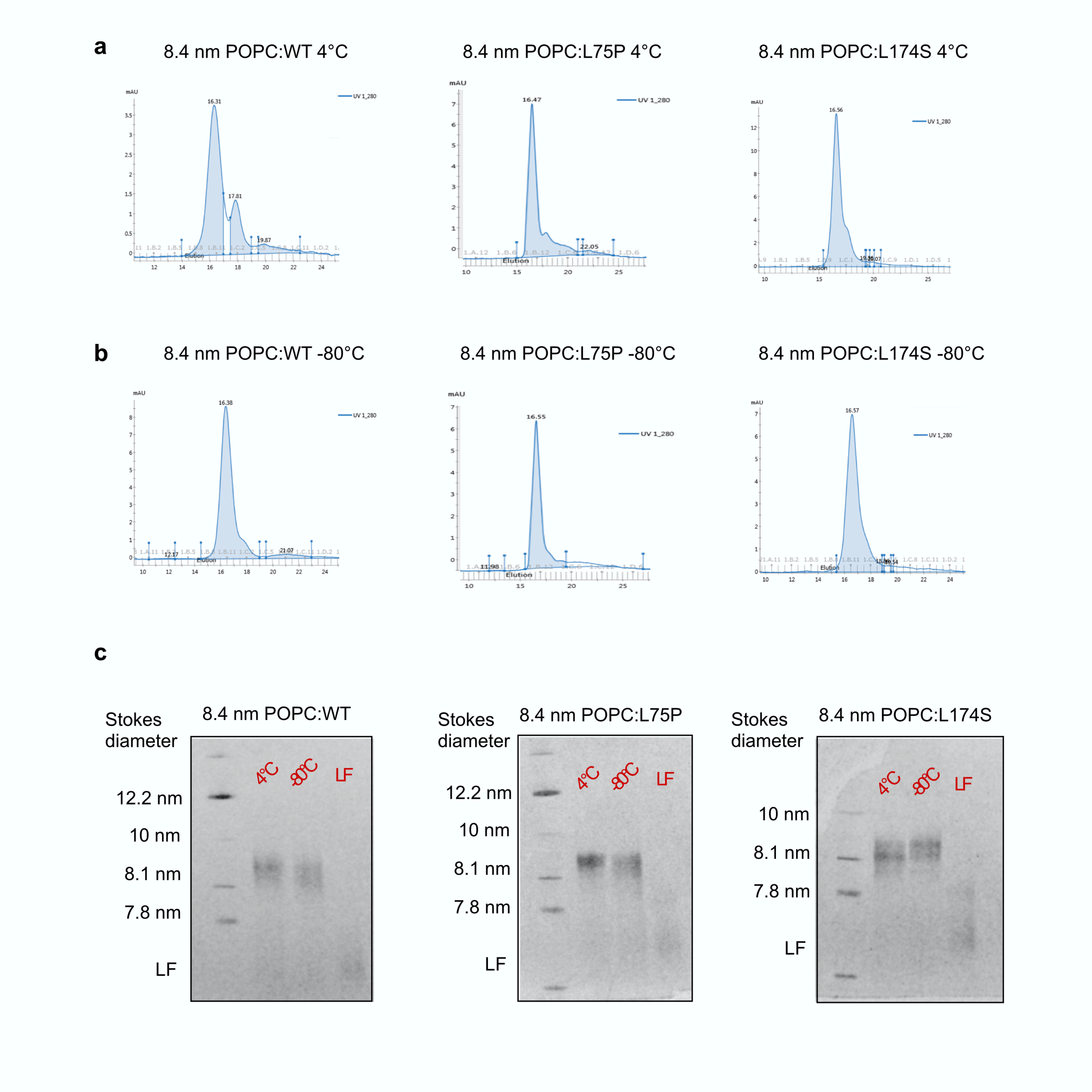

Supplemental Figure S1: No dissociation of lipid free protein from the HDL particles is observed after freezing at -80°C. **a**, Newly prepared HDL particles containing wild type (WT), L75P or L174S ApoA-I were separated by SEC to ensure homogeneous particle preparation. **b**, The peak fraction was frozen at -80°C, thawed, and analysed by SEC. **c**, Native Page representing peak fraction corresponding to 8.4 nm POPC particles containing WT, L75P or L174S ApoA-I protein stored at 4°C or frozen at -80°C. Lipid free protein (LF) was added for comparison. 2.5 µg of protein were loaded per lane.

Supplemental Figure S2

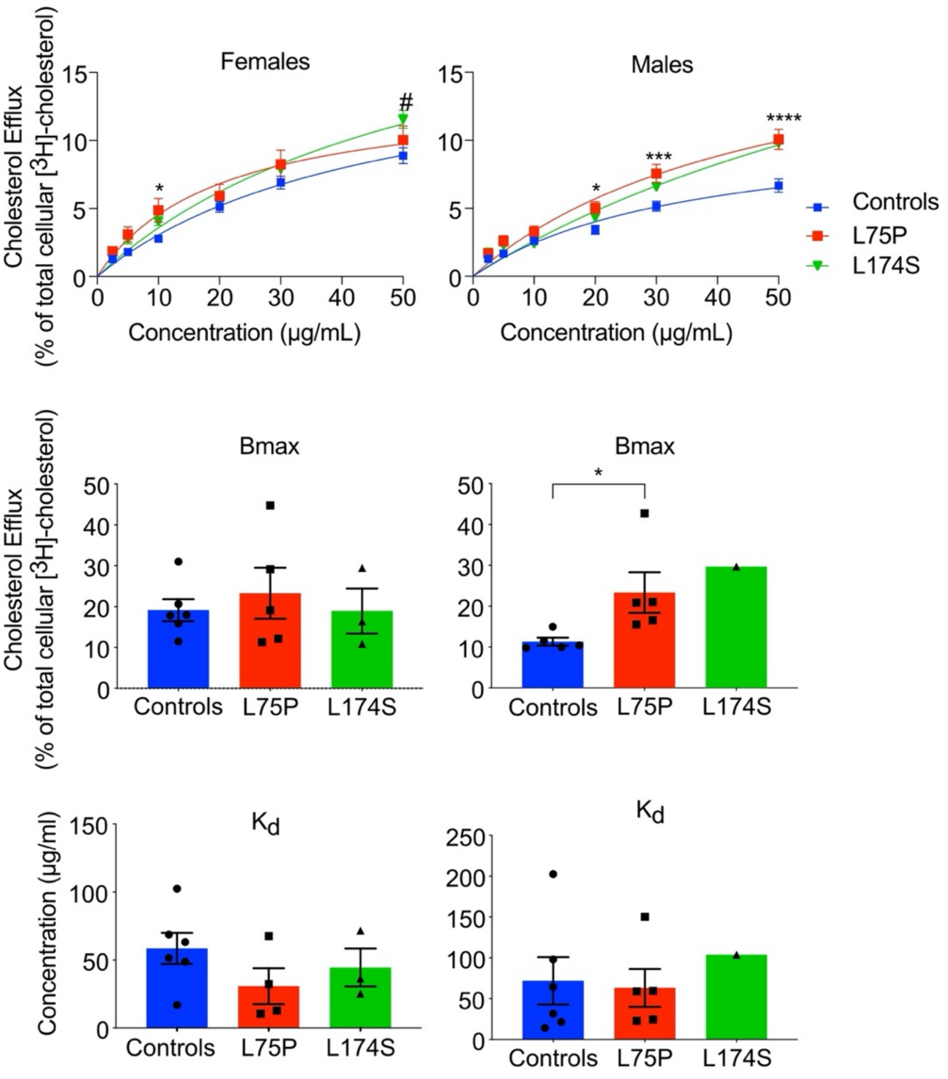

Supplemental Figure S2. The improved efflux capacity of the amyloidogenic ApoA-I patients is sex specific

Cholesterol efflux data obtained from experiment shown in Figure 1c were plotted according to donor's sex. Experimental data (upper panels), calculated Bmax (middle panels) and Kd (lower panels). Data shown are the mean ± SEM and significance is calculated according to two-way ANOVA (*$p<0.05$, ***$p<0.001$, ****$p<0.0001$ for L75P patients as compared to healthy donors).

Supplemental Figure S3

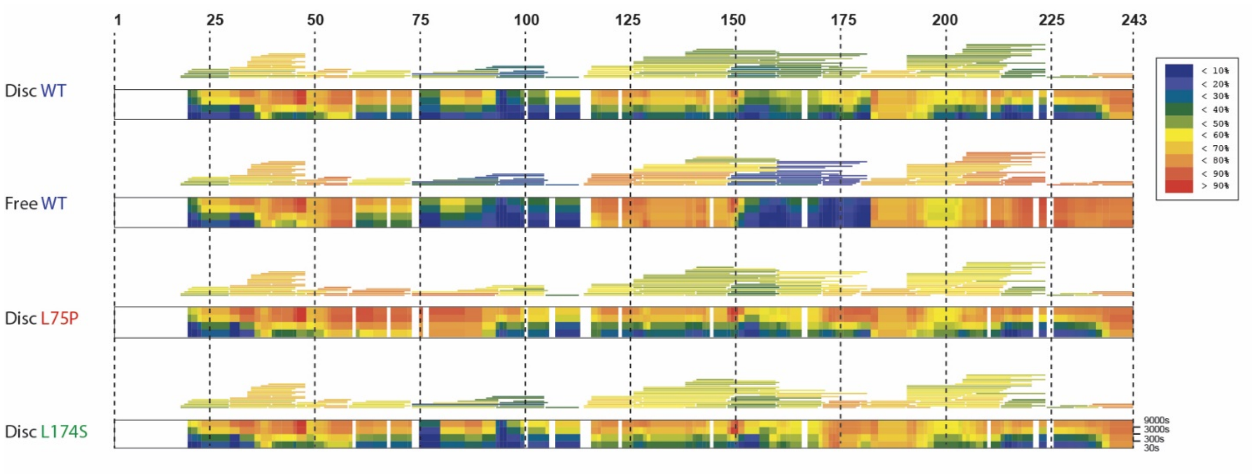

Supplemental Figure S3. Individual HDX heatmaps of WT, L75P and L174S ApoA-I in 8.4 nm rHDL

Individual state heatmaps and coverage bars used for generating the differential heatmaps in figure 5. Peptide coverage is illustrated by the bars above each heatmap and colored coded to the average deuterium uptake over all observed time points. The heatmaps shows the deuterium uptake at the different time points (30 s, 300s, 3000s and 9000s), and are color coded based on a least square averaging of observed deuterium uptake. Cold color = slower exchange; warm color = faster exchange. see color key to the right. Individual deuterium uptake curves for all observed peptides can be found in the supplementary file, Supplementary datafile S1.pdf.

Supplemental Figure S4

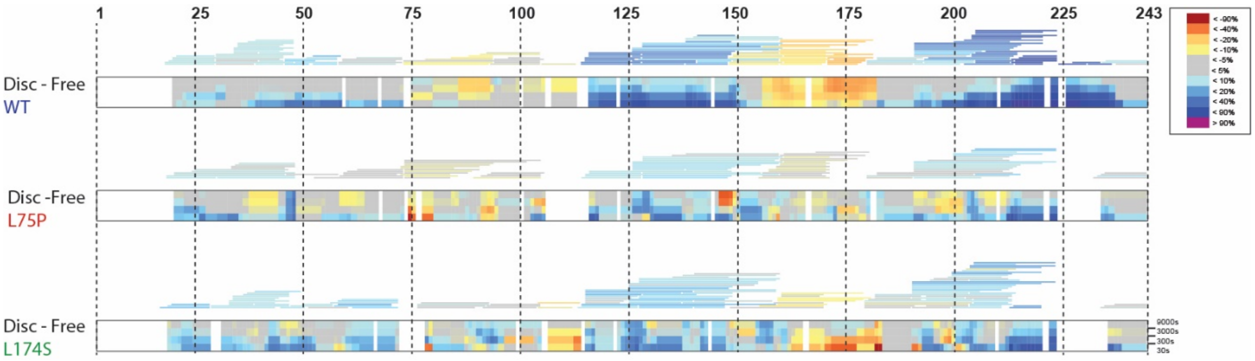

Supplemental Figure S4. Differential heatmaps of lipid bound vs lipid free form of WT, L75P and L174S proteins

Top panel, Disc-Free WT is same data as shown in figure 5A, here also showing coverage bars. The differential peptide coverage is illustrated by the bars above each heatmap and colored coded to the average deuterium uptake over all observed time points. The coverage bars for each variant show that most regions are characterized by the same protection/exposure pattern as the WT. The heatmaps shows the deuterium uptake at the different time points (30 s, 300s, 3000s and 9000s, note direction of time to the left), and are color coded based on a least square averaging of observed deuterium uptake. Cold color = slower exchange; warm color = faster exchange. See color key to the right.

Supplemental Figure S5

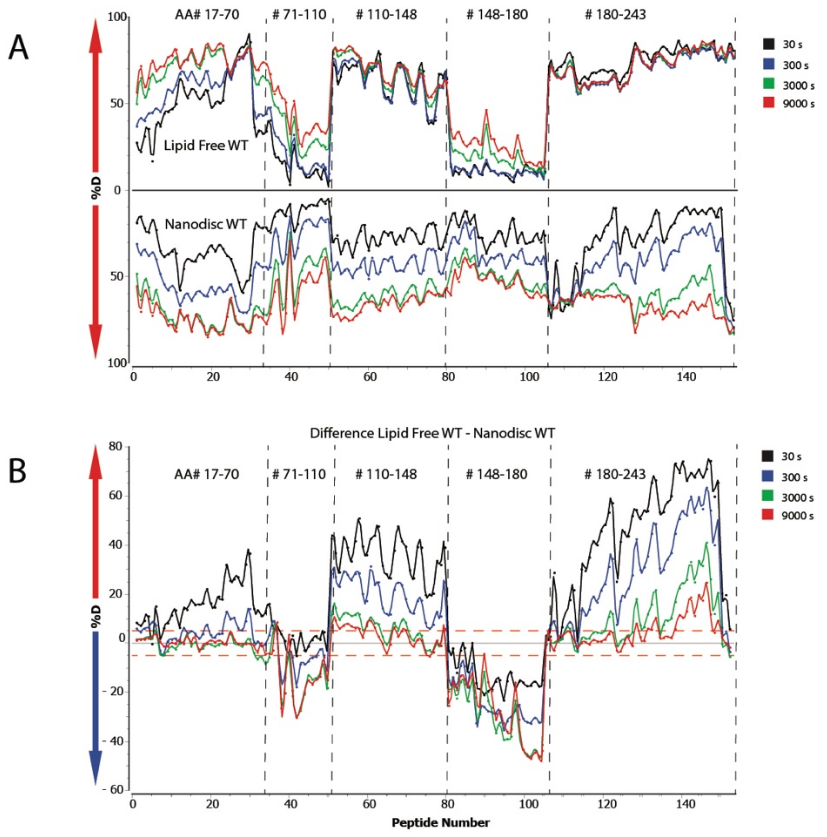

Supplemental Figure S5. Butterfly plot of lipid bound vs lipid free form of WT ApoA-I

A, butterfly plot with a central zero between plots for WT ApoA-I in the lipid free form and in nanodiscs. Red arrows indicate increase in uptake along the y-axis in % deuteration. The X-axis reports the peptide number (not aminoacidic residue number), whereas the dashed horizontal lines provides approximate AA (aminoacidic residue) location along the sequence. The peptide number can be used to track the corresponding individual uptake graph in the file HDX_S2.pdf. Regions showing higher uptake in the lipid free form (17-70, 110-148 and 180-223) becomes more protected upon lipidation. **B**, butterfly plot showing the residuals of plot A. Red arrow denotes comparative increase in uptake and the blue one indicates a decrease in uptake. At low exchange times there is a large protection for the nanodisc state compared to the lipid free protein (17-70, 110-148 and 180-223). Conversely, there is a decrease in the protection for the disc state at 71-110 and 148-180, where the free protein is more ordered.

The different curve colors indicate deuterium uptake at the different time points (right legend).

Supplemental Figure S6-1

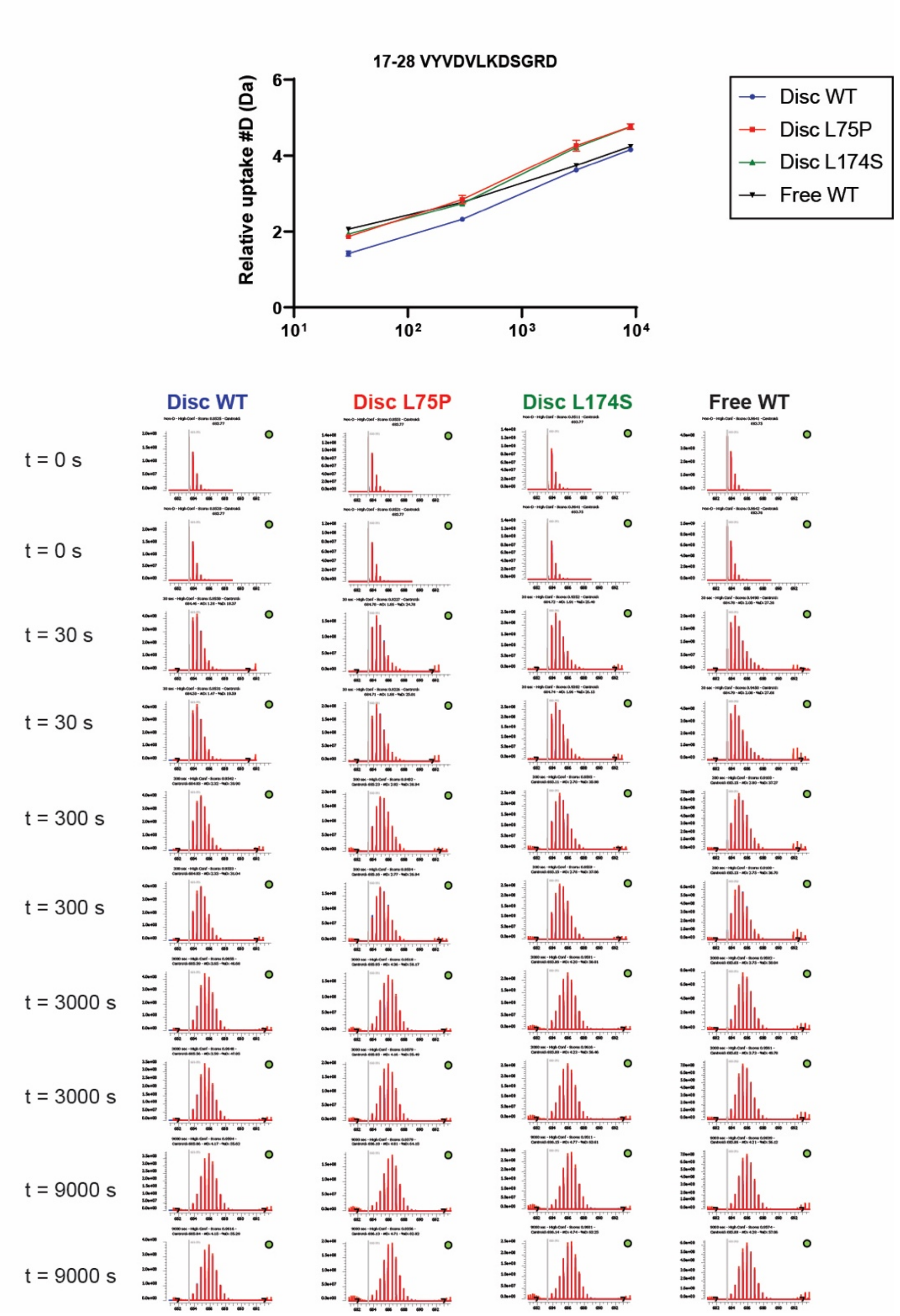

Supplemental Figure S6-1. Isotopic envelopes for all duplicate mass spectra behind the uptake curves in figure 6, as observed for each protein variant. Gray lines indicate the undeuterated peptide mass. For the peptide 17-28 no EX1 was observed.

Supplemental Figure S6-2

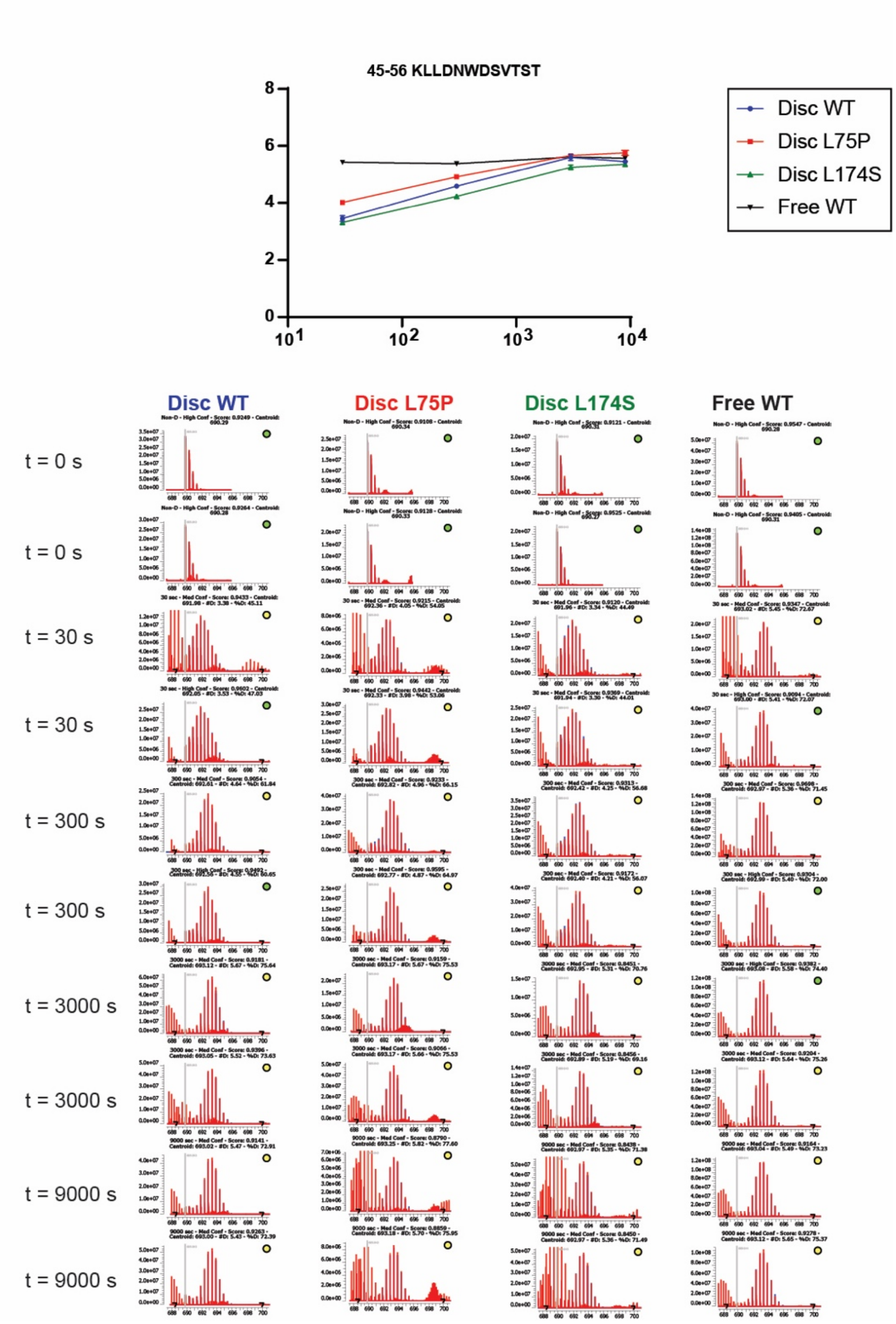

Supplemental Figure S6-2. Isotopic envelopes for all duplicate mass spectra behind the uptake curves in figure 6, as observed for each protein variant. Gray lines indicate the undeuterated peptide mass. For the peptide 45-56 no EX1 was observed.

Supplemental Figure S6-3

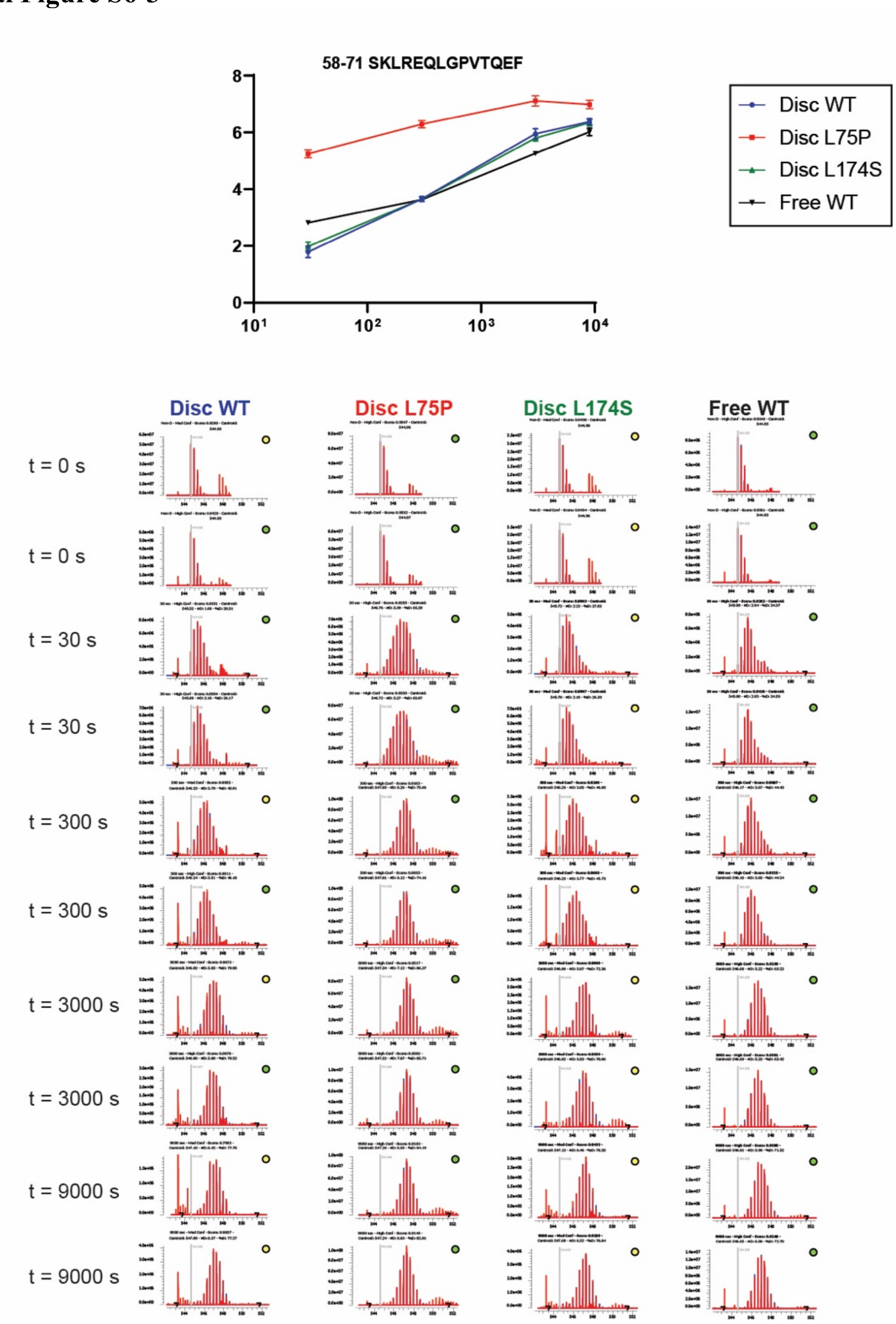

Supplemental Figure S6-3. Isotopic envelopes for all duplicate mass spectra behind the uptake curves in figure 6, as observed for each protein variant. Gray lines indicate the undeuterated peptide mass. For the peptide 58-71 some EX1 in the form of broadening at 30s can be seen.

Supplemental Figure S6-4

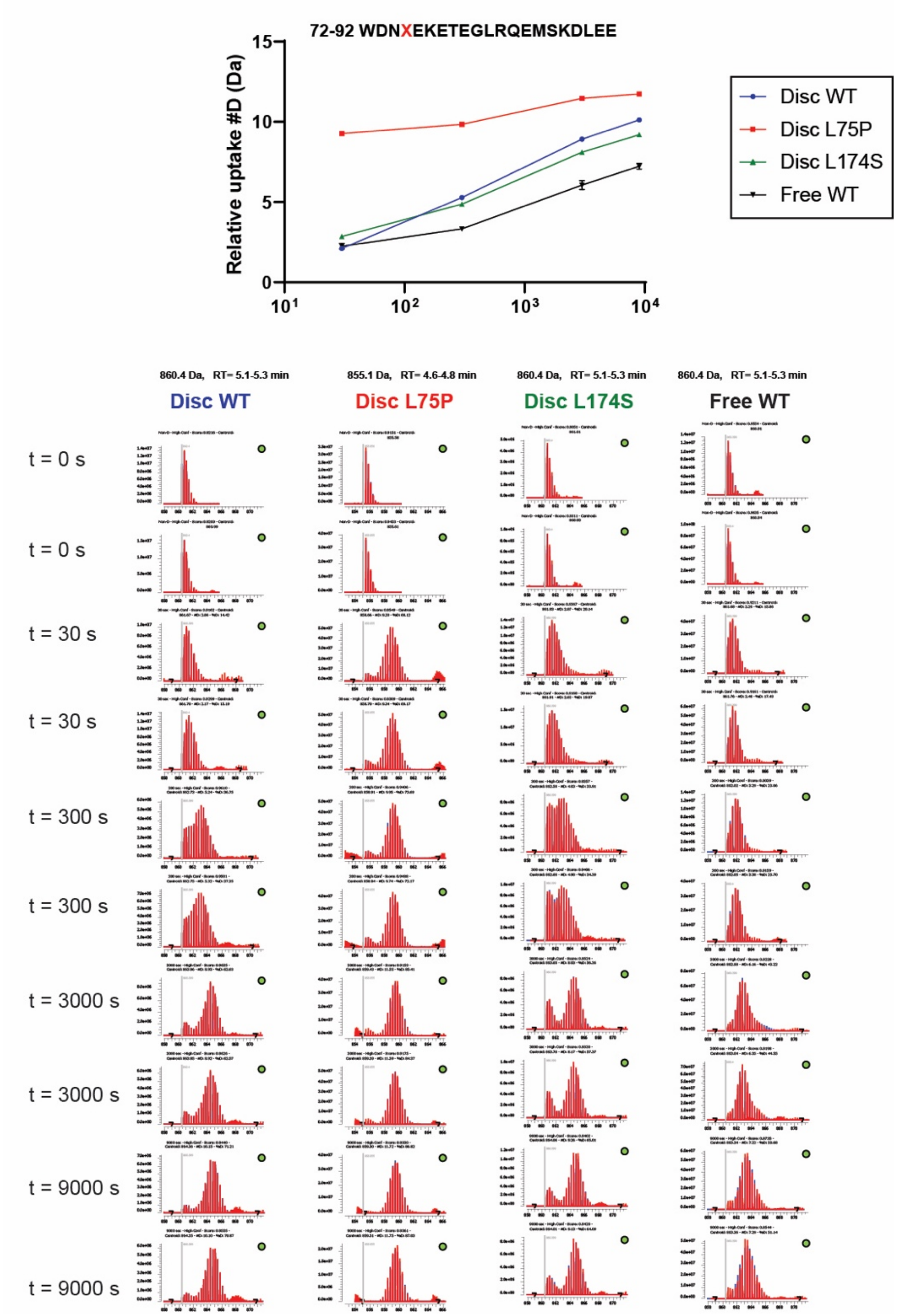

Supplemental Figure S6-4. Isotopic envelopes for all duplicate mass spectra behind the uptake curves in figure 6, as observed for each protein variant. Gray lines indicate the undeuterated peptide mass. For the peptide 72-92 complex bimodal isotope distributions can be observed, indicative of different dynamic conformational changes in this region.

Supplemental Figure S6-5

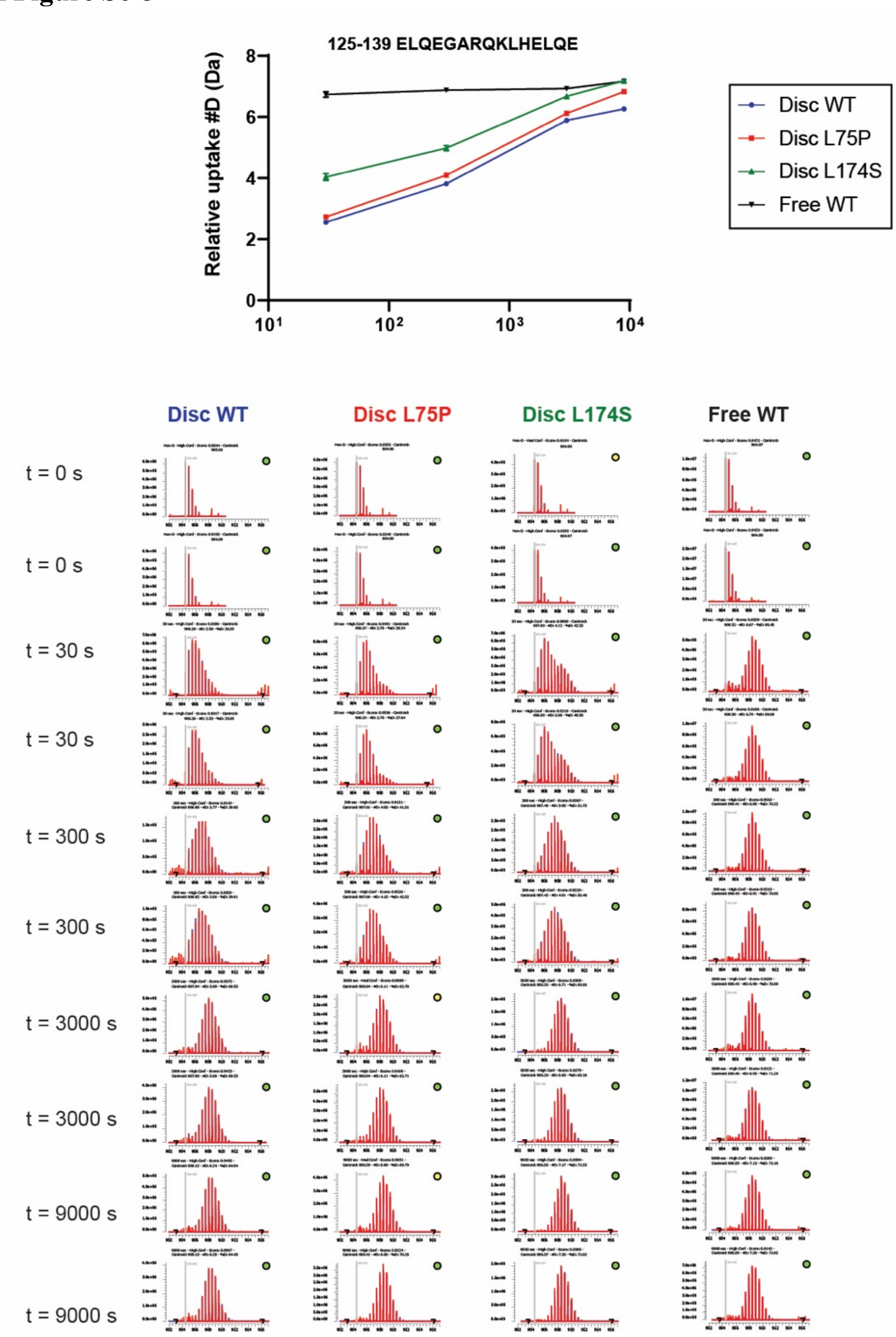

Supplemental Figure S6-5. Isotopic envelopes for all duplicate mass spectra behind the uptake curves in figure 6, as observed for each protein variant. Gray lines indicate the undeuterated peptide mass. For the peptide 125-139 some EX1 can be observed, most obvious for L174S at 30-300s, it should be noted that this peptide covers a smaller region with a more homogeneous conformational behavior than larger peptides spanning 115-159.

Supplemental Figure S6-6.

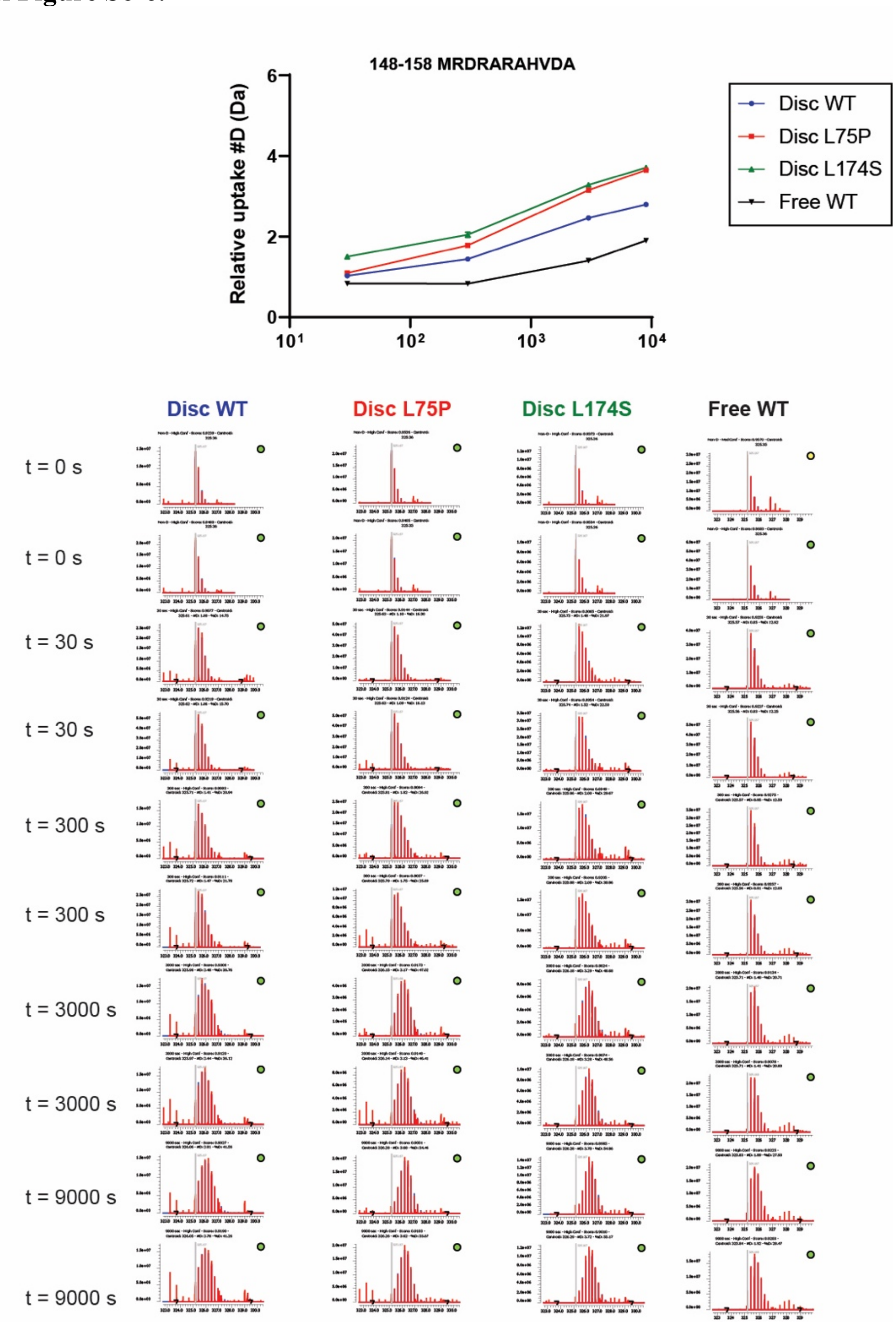

Supplemental Figure S6-6. Isotopic envelopes for all duplicate mass spectra behind the uptake curves in figure 6, as observed for each protein variant. Gray lines indicate the undeuterated peptide mass. For the peptide 148-158 no obvious bimodal isotope distributions can be observed, indicative of similar dynamics in the conformational changes for the rHDL particles in this region.

Supplemental Figure S6-7

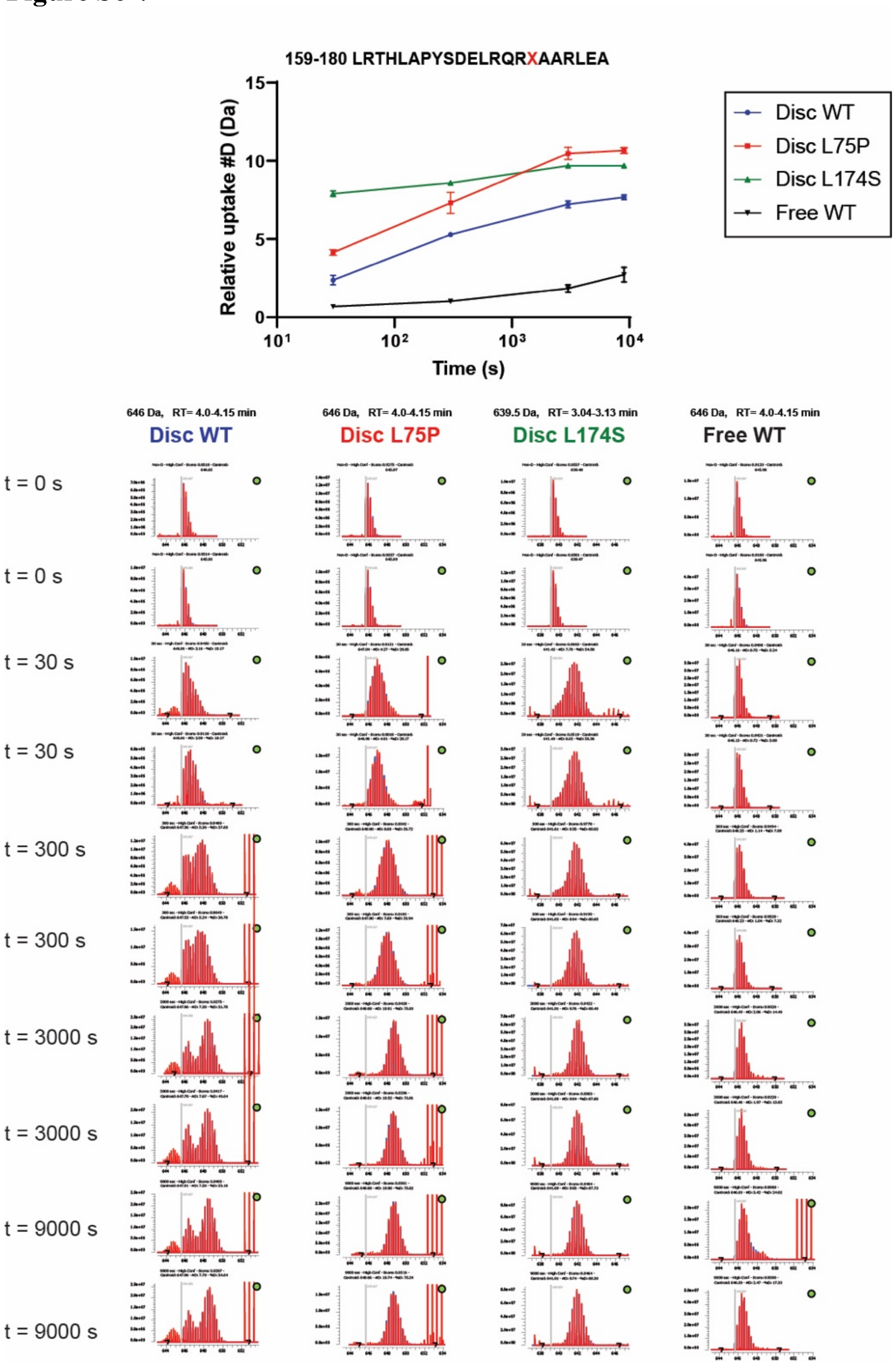

Supplemental Figure S6-7. Isotopic envelopes for all duplicate mass spectra behind the uptake curves in figure 6, as observed for each protein variant. Gray lines indicate the undeuterated peptide mass. For the peptide 159-180 some bimodal isotope distributions can be observed, here particularly obvious for the rHDL WT, were the slow exchanging population remains even at the longest labelling times. This can be seen for several disc WT peptides in this region, very unlikely due to an artefact or free protein and probably an effect of the complex dynamics of WT disc in this region (see figure S4) that then disappears in the variants that presents a unimodal faster uptake.

Supplemental Figure S6-8

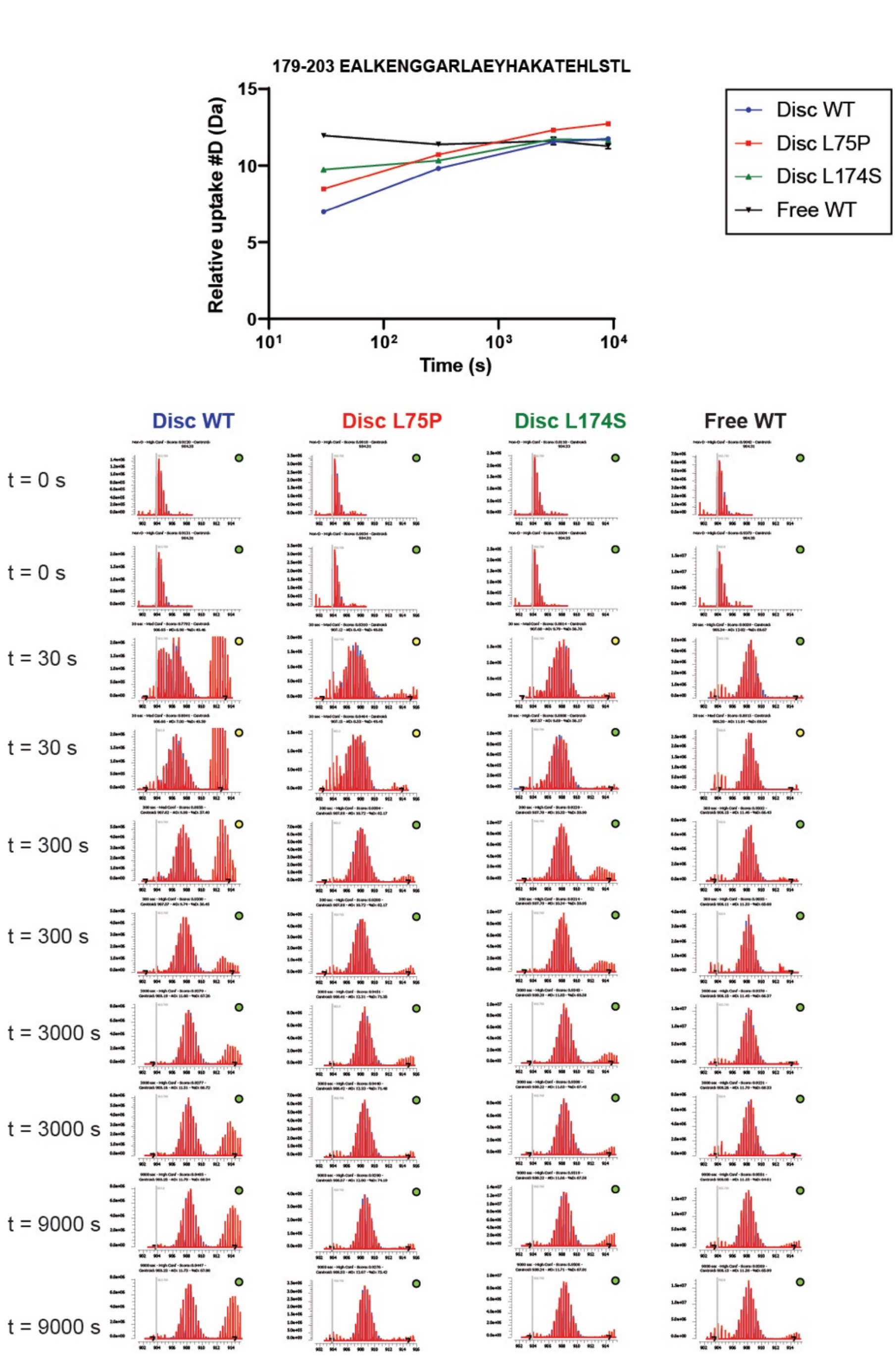

Supplemental Figure S6-8. Isotopic envelopes for all duplicate mass spectra behind the uptake curves in figure 6, as observed for each protein variant. Gray lines indicate the undeuterated peptide mass. For the peptide 179-203 there is some broadening at low times 30s, but no other signs of EX1.

Supplemental Figure S6-9

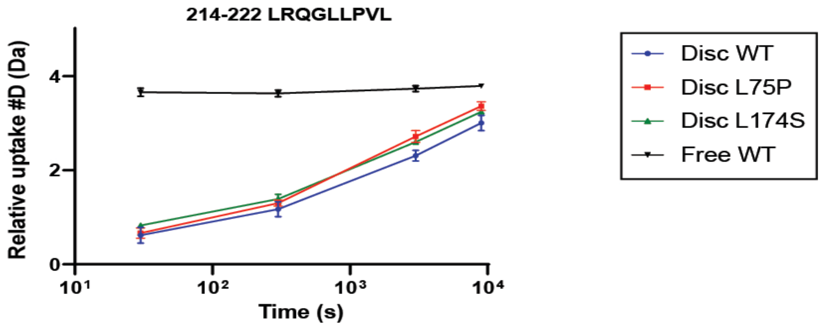

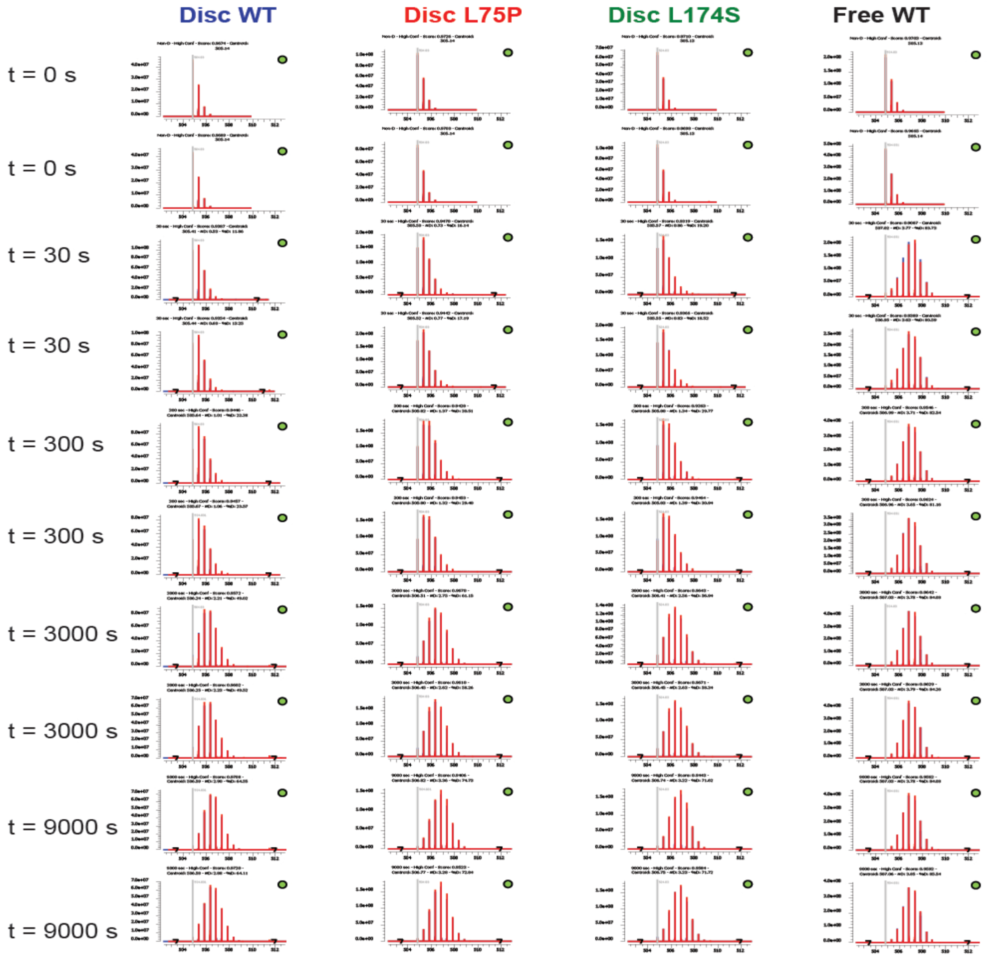

Supplemental Figure S6-9. Isotopic envelopes for all duplicate mass spectra behind the uptake curves in figure 6, as observed for each protein variant. Gray lines indicate the undeuterated peptide mass. For the peptide 214-222 no bimodal isotope distributions can be observed.

Supplemental Figure S7

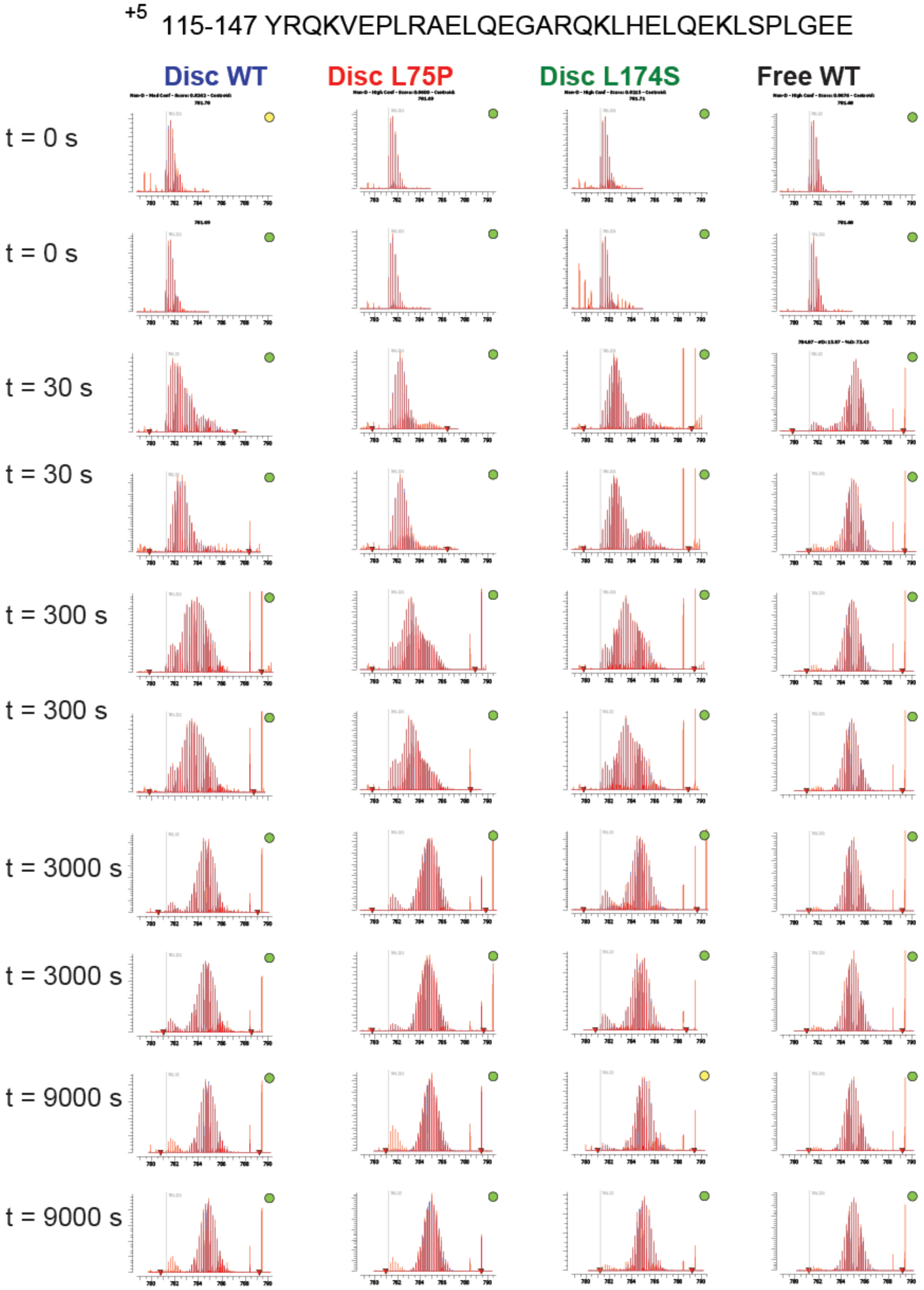

Supplemental Figure S7. Isotopic envelopes for all duplicate mass spectra for the peptide spanning 115-147, as observed for each protein variant. Gray lines indicate the undeuterated peptide mass. Here, bimodal isotope distributions can be observed for all the rHDL states at 30-300s. Moreover, L174S, which has the overall most exposed middle region, also showes the most obvious fast exchanging fraction at 30s.

Supplemental Figure S8

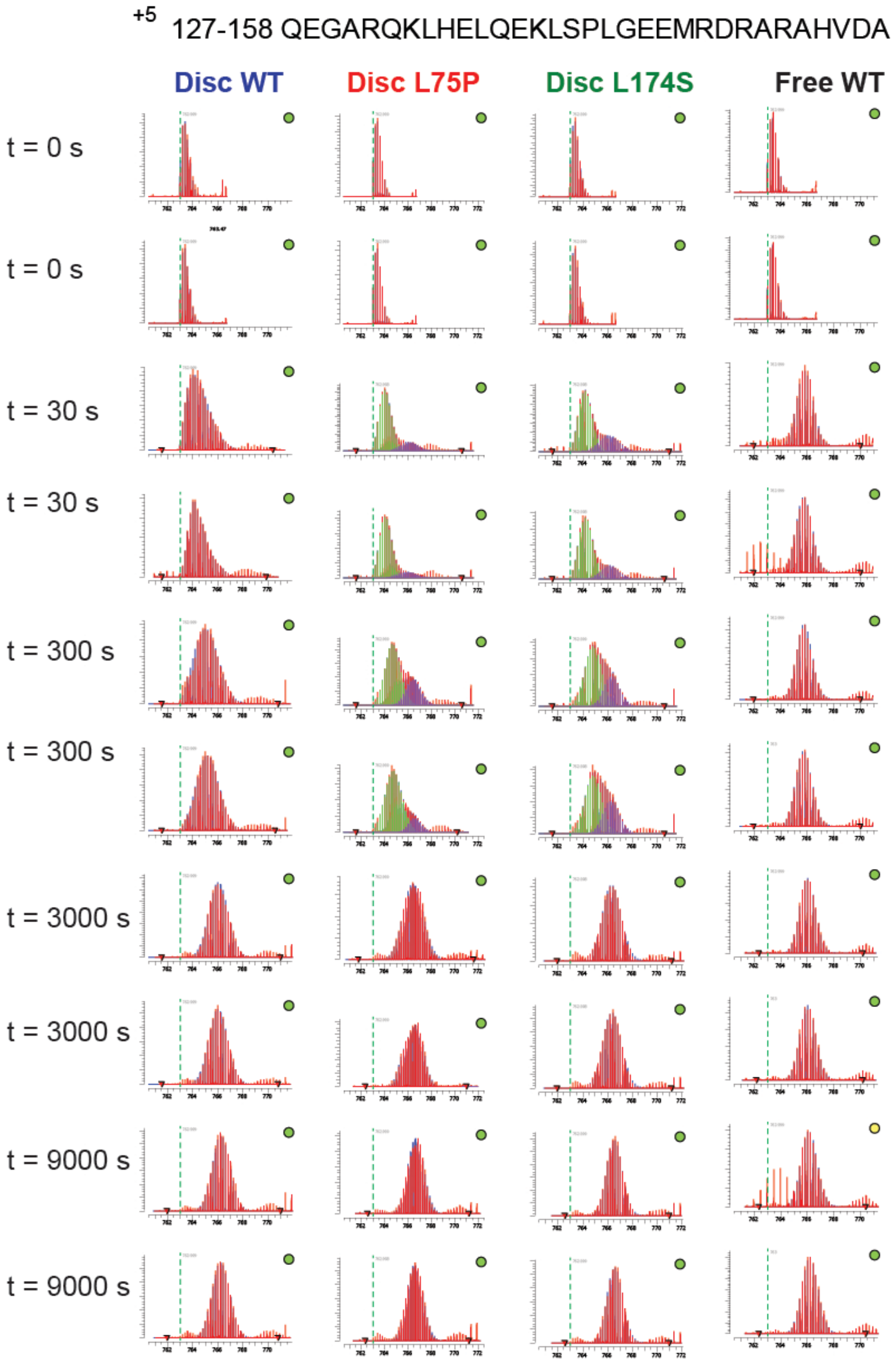

Supplemental Figure S8. Isotopic envelopes for all duplicate mass spectra for the peptide spanning 127-158, as observed for each protein variant. Green dashed lines indicate the undeuterated peptide mass, bimodal isotope distributions as identified by HDExaminer are exemplified by green (slow) and purple fast exchanging populations. Here, bimodal isotope distributions are most evident for L75P and L174S at 30-300s, also for the disc WT there is some broadening at 30s-300s, but for the lipid free WT no EX1 can be observed.
