# Supplementary material for: Structure dynamics of ApoA-I amyloidogenic variants in small HDL increase their ability to mediate cholesterol efflux
Source: J Lipid Res. 2020 Nov 24;62:100004. doi: 10.1194/jlr.RA120000920 (PMC7890215; doi:10.1194/jlr.RA120000920)

Supplementary datafile to:

Structure dynamics of ApoA-I amyloidogenic variants in small HDL increase their ability to mediate cholesterol efflux

Oktawia Nilsson[1], Mikaela Lindvall[1], Laura Obici[2], Simon Ekström[3], Jens O. Lagerstedt[1,4,*],

Rita Del Giudice[1,*,#]

[1] Department of Experimental Medical Science, Lund University, SE-221 84 Lund, Sweden; [2] Amyloidosis Research & Treatment Centre, Fondazione IRCCS Policlinico San Matteo, Pavia, 27100, Italy; [3] BioMS - Swedish National Infrastructure for Biological Mass Spectrometry, Lund University, SE-221 84 Lund, Sweden; [4] Lund Institute of Advanced Neutron and X-ray Science (LINXS), SE-221 84 Lund, Sweden

* Corresponding authors:

Jens O Lagerstedt; jens.lagerstedt@med.lu.se

Rita Del Giudice, rita.del_giudice@med.lu.se

[#] Author's current address: Malmö University, Department of Biomedical Science, 214 32 Malmö, Sweden. Email: rita.del-giudice@mau.se

Data included here: supplementary datafile S1 showing all the 150 individual deuterium uptake plots for all the peptides used in the analysis. The uptake plots have error bars representing the confidence interval of a student's t-distribution (95% confidence).

17-28: VYVDVLKDSGRD (#1)

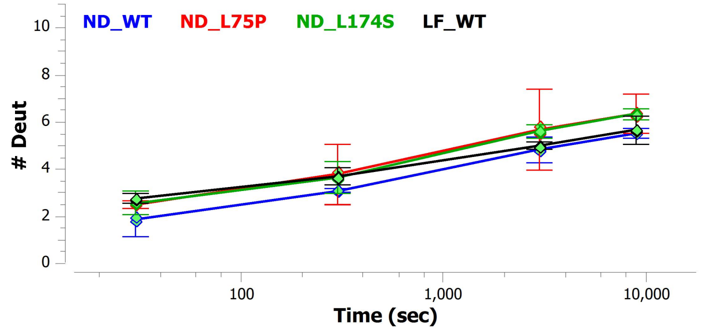

17-46: VYVDVLKDSGRDYVSQFEGSALGKQLNLKL (#2)

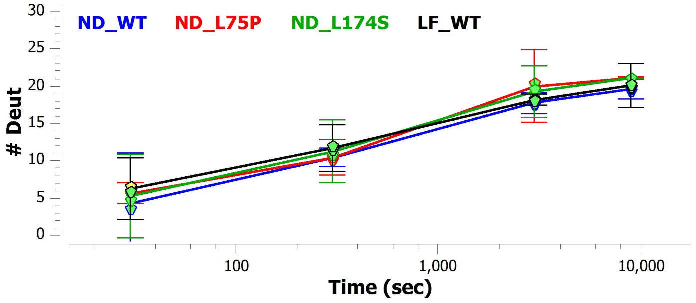

18-28: YVDVLKDSGRD (#3)

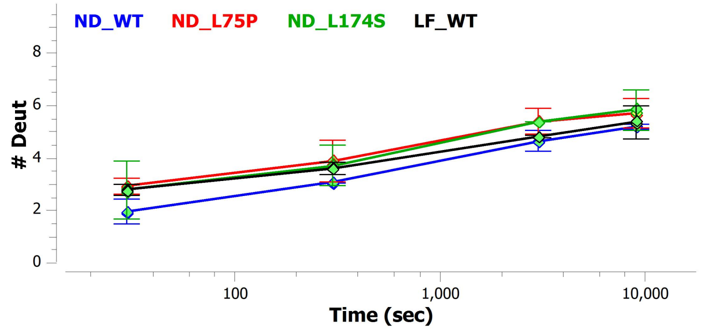

19-28: VDVLKDSGRD (#4)

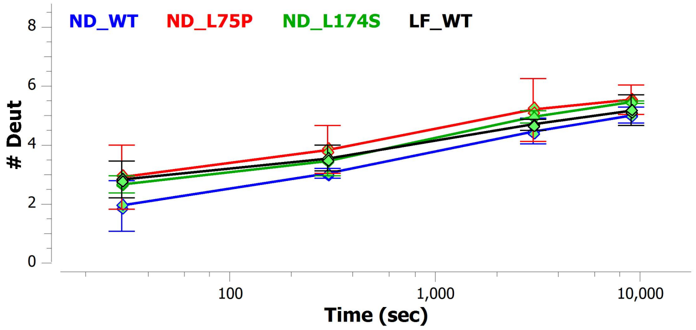

19-44: VDVLKDSGRDYVSQFEGSALGKQLNL (#5)

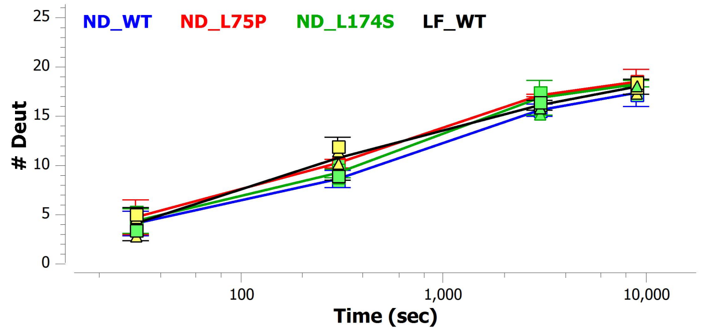

20-28: DVLKDSGRD (#6)

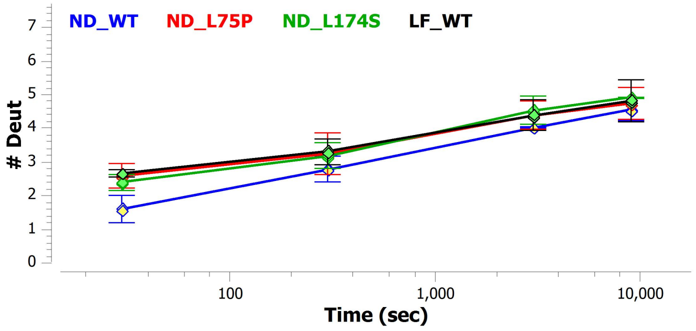

29-37: YVSQFEGSA (#7)

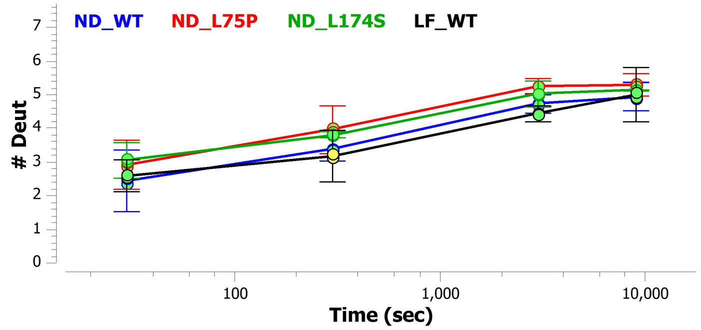

29-38: YVSQFEGSAL (#8)

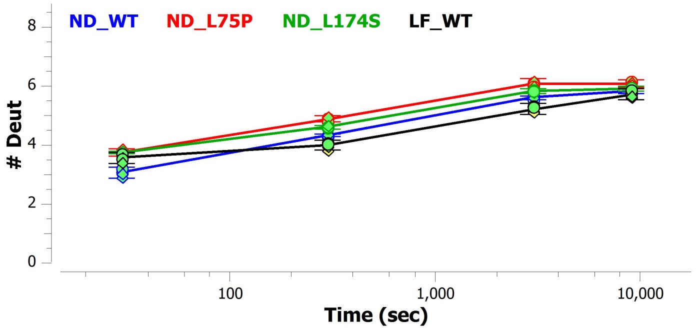

29-43: YVSQFEGSALGKQLN (#9)

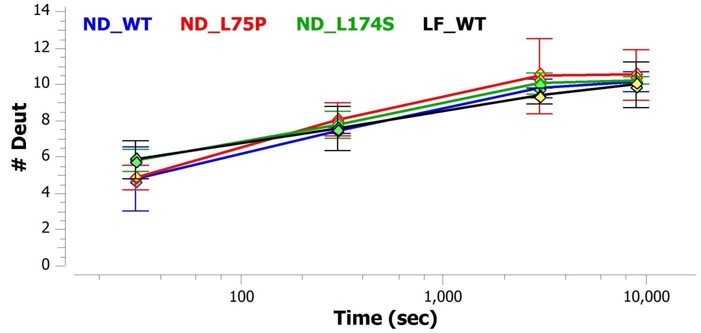

29-44: YVSQFEGSALGKQLNL (#10)

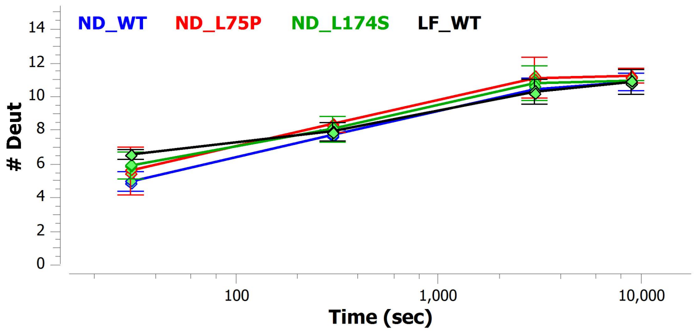

29-46: YVSQFEGSALGKQLNLKL (#11)

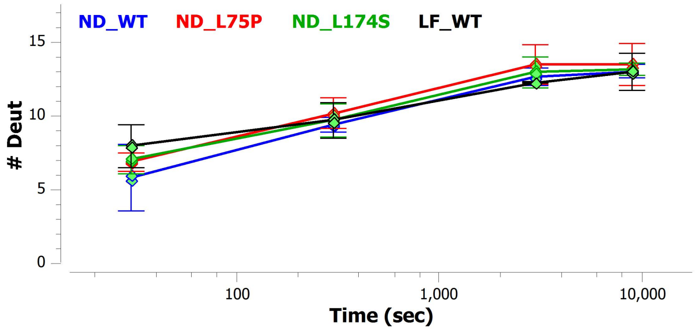

33-38: FEGSAL (#12)

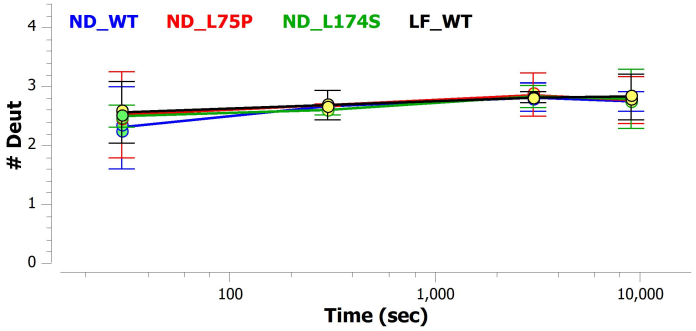

33-43: FEGSALGKQLN (#13)

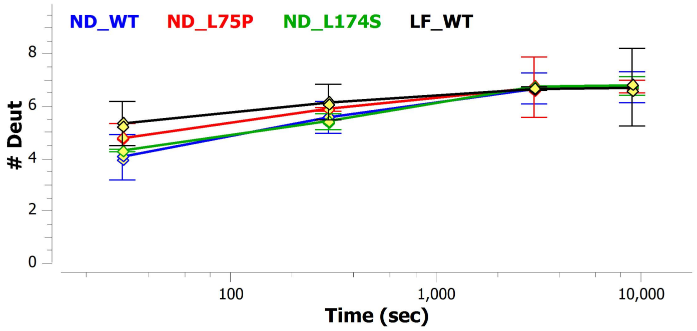

33-44: FEGSALGKQLNL (#14)

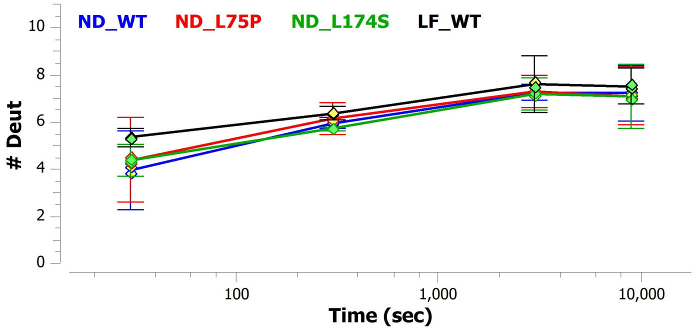

33-46: FEGSALGKQLNLKL (#15)

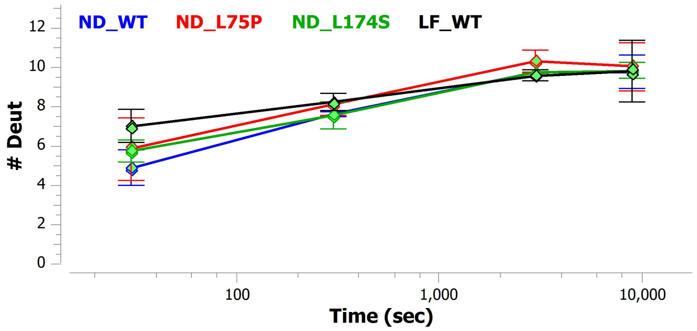

34-43: EGSALGKQLN (#16)

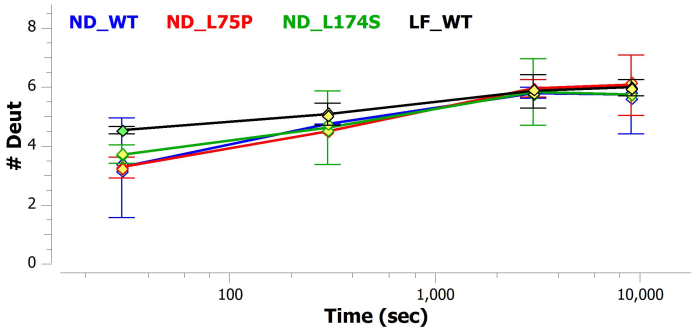

34-44: EGSALGKQLNL (#17)

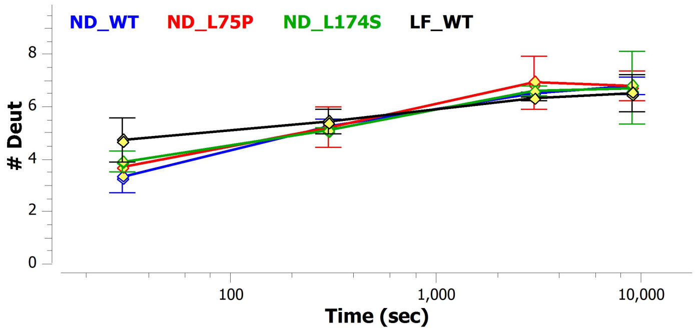

34-46: EGSALGKQLNLKL (#18)

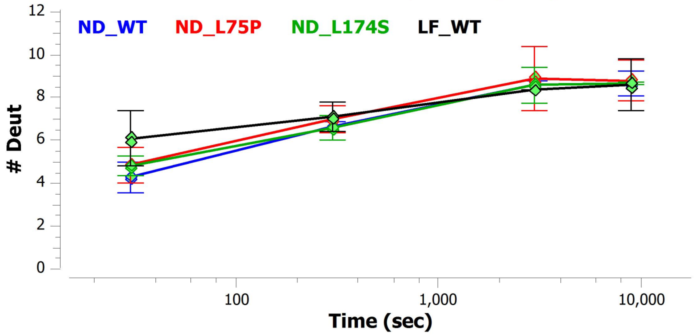

36-46: SALGKQLNLKL (#19)

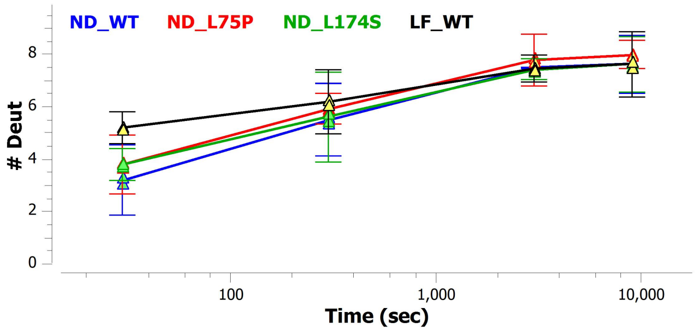

38-44: LGKQLNL (#20)

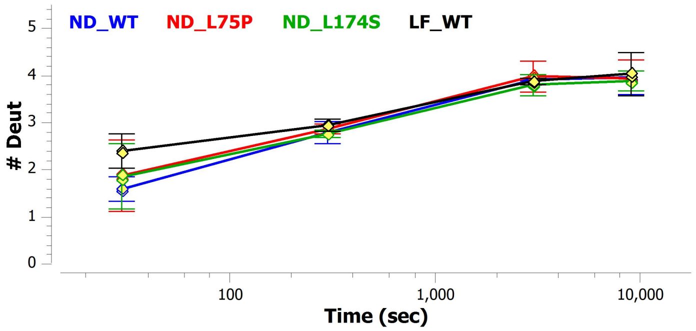

38-46: LGKQLNLKL (#21)

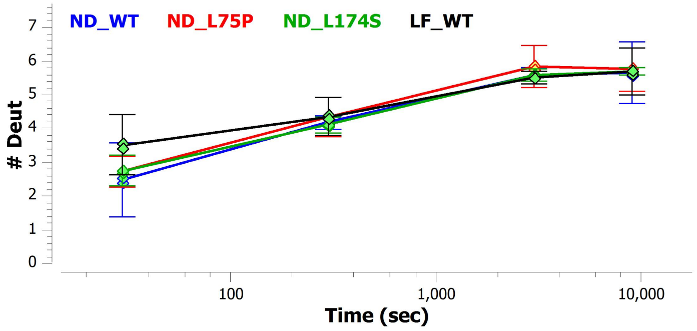

39-44: GKQLNL (#22)

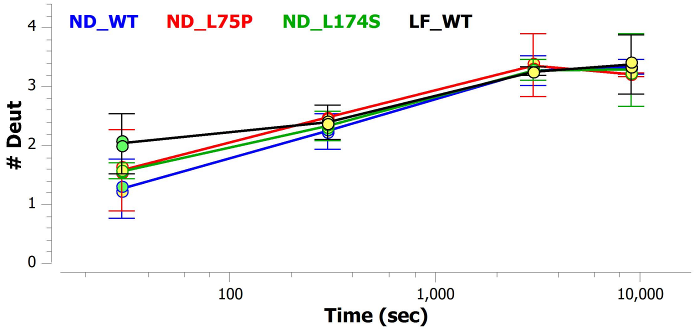

39-46: GKQLNLKL (#23)

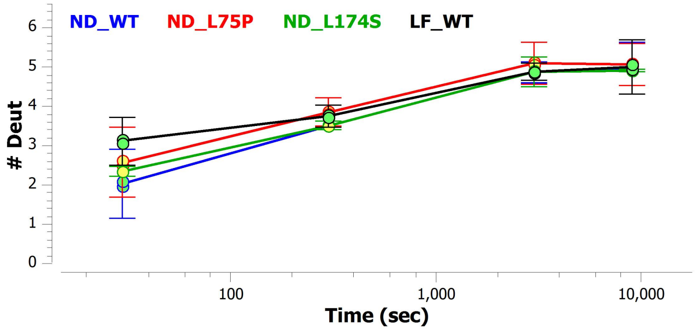

45-50: KLLDNW (#24)

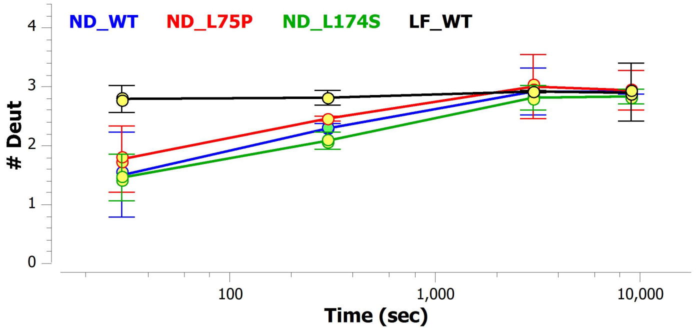

45-51: KLLDNWD (#25)

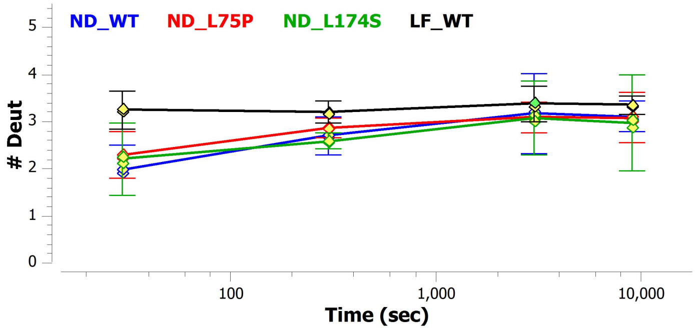

45-56: KLLDNWDSVTST (#26)

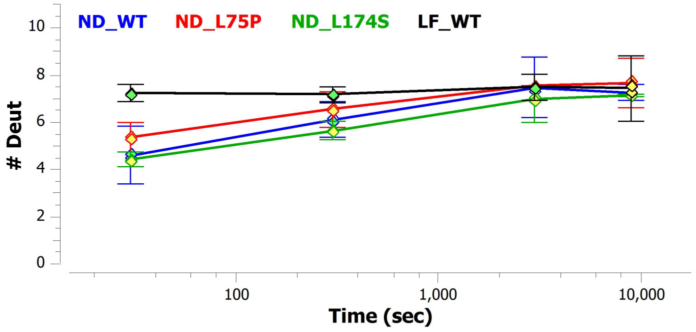

47-56: LDNWDSVTST (#27)

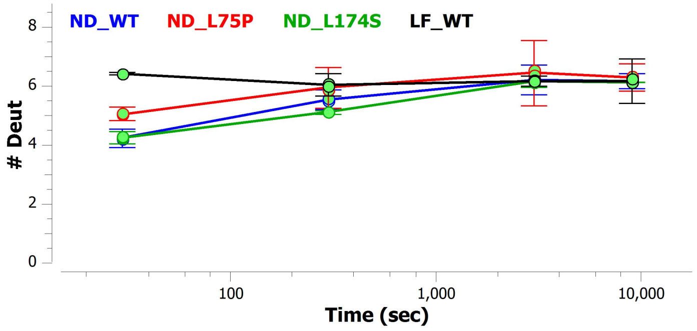

50-56: WDSVTST (#28)

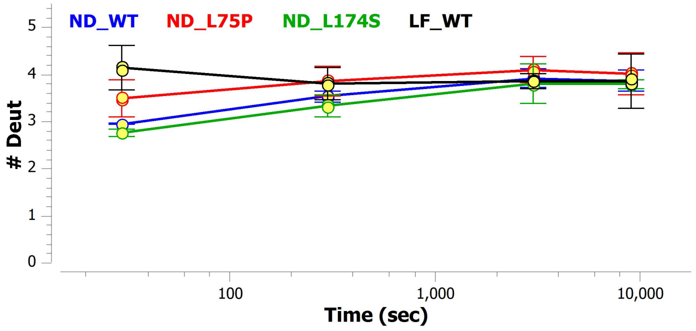

51-56: DSVTST (#29)

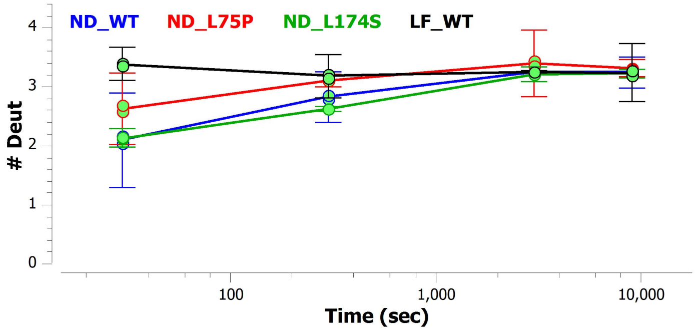

51-57: DSVTSTF (#30)

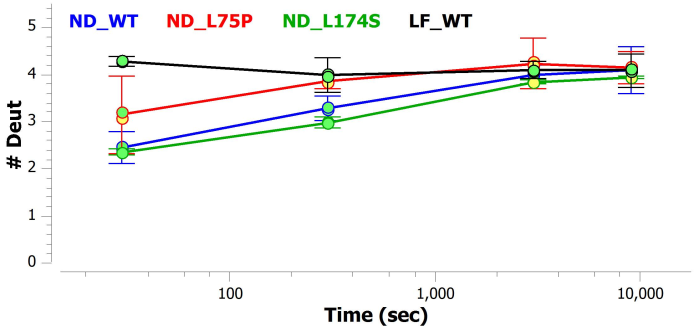

57-70: FSKLREQLGPVTQE (#31)

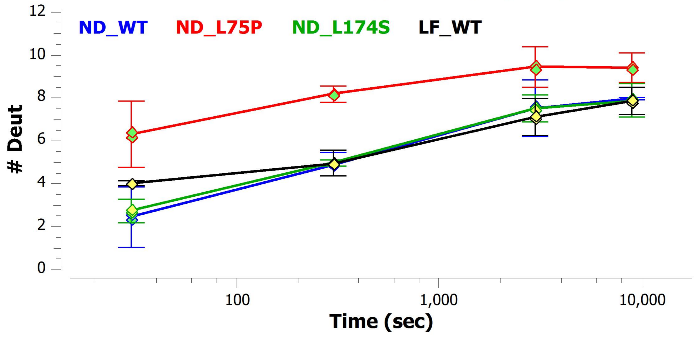

57-71: FSKLREQLGPVTQEF (#32)

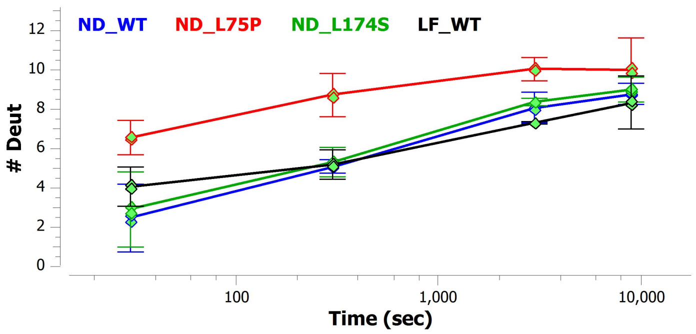

58-70: SKLREQLGPVTQE (#33)

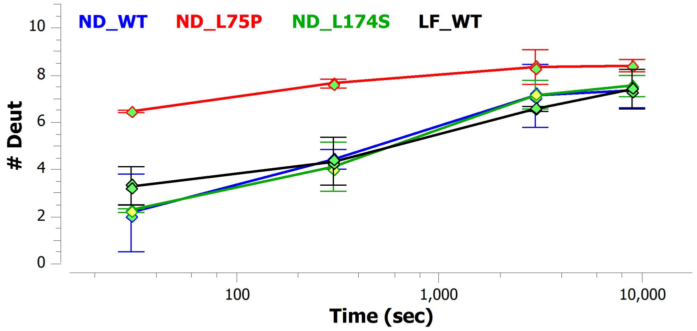

58-71: SKLREQLGPVTQEF (#34)

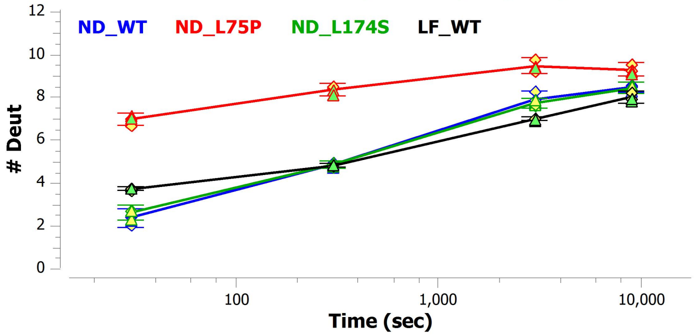

61-71: REQLGPVTQEF (#35)

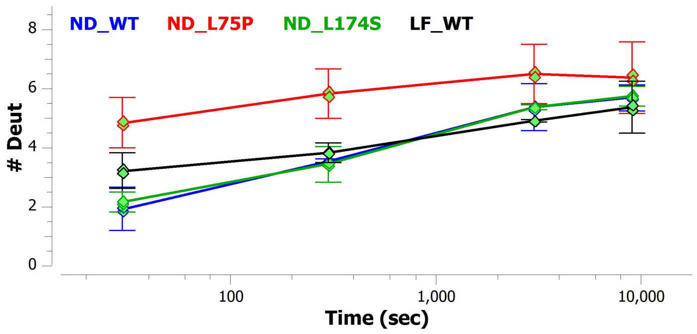

72-91: WDNXEKETEGLRQEMSKDLE (#36)

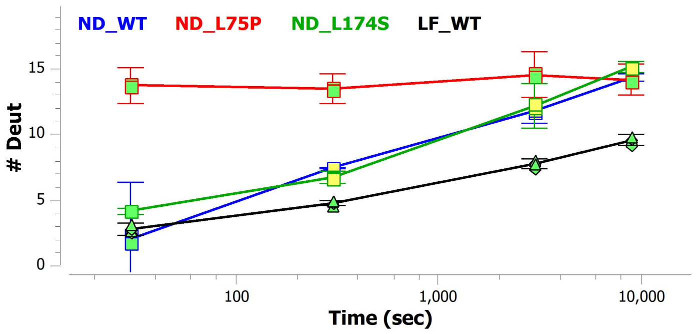

72-92: WDNXEKETEGLRQEMSKDLEE (#37)

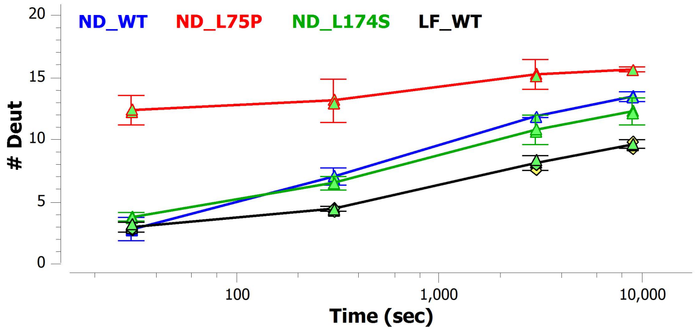

72-103: WDNXEKETEGLRQEMSKDLEEVKAKVQPYLDD (#38)

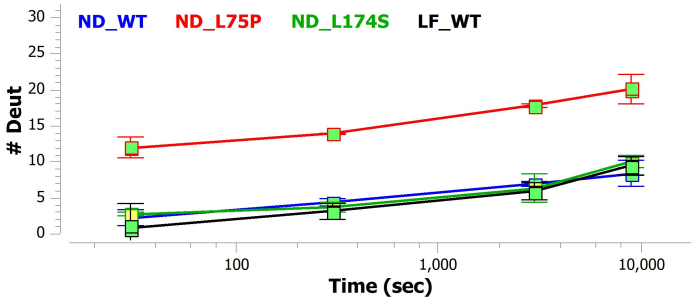

77-92: KETEGLRQEMSKDLEE (#39)

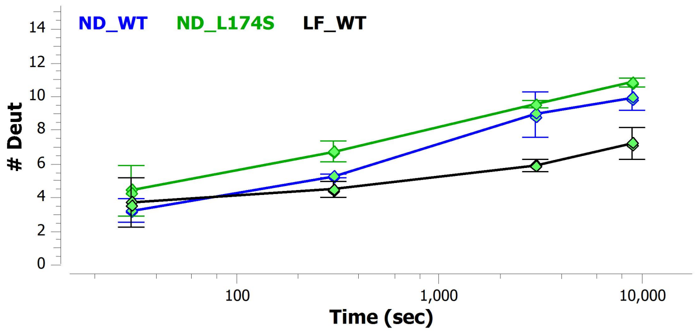

83-92: RQEMSKDLEE (#40)

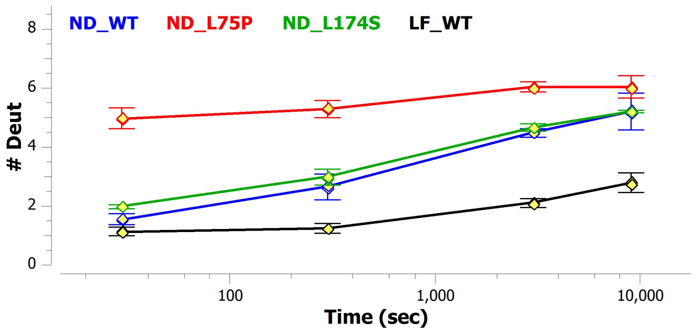

87-92: SKDLEE (#41)

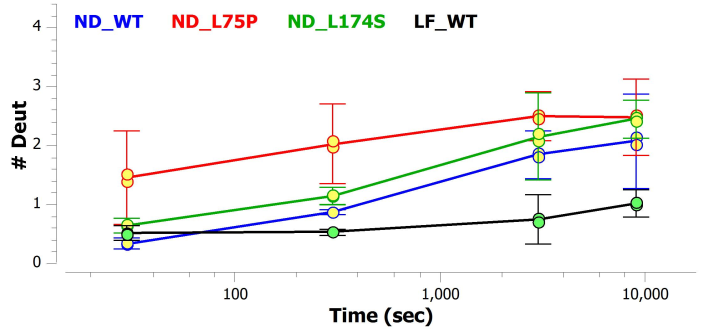

90-103: LEEVKAKVQPYLDD (#42)

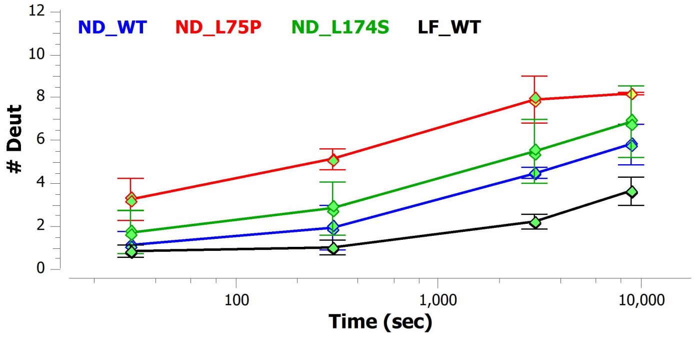

91-103: EEVKAKVQPYLDD (#43)

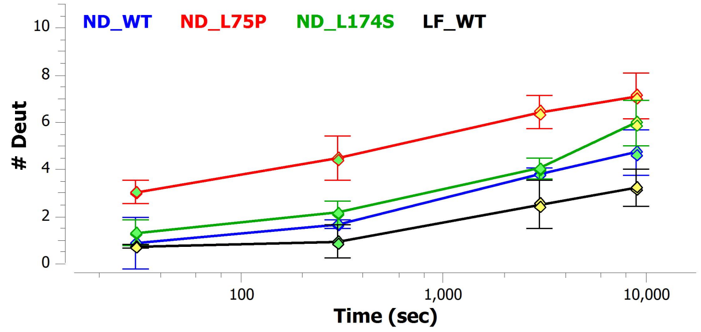

92-103: EVKAKVQPYLDD (#44)

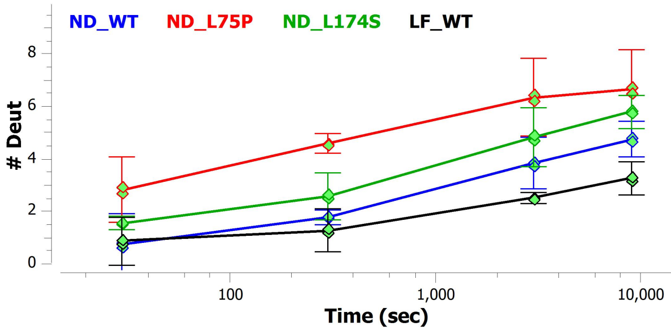

93-103: VKAKVQPYLDD (#45)

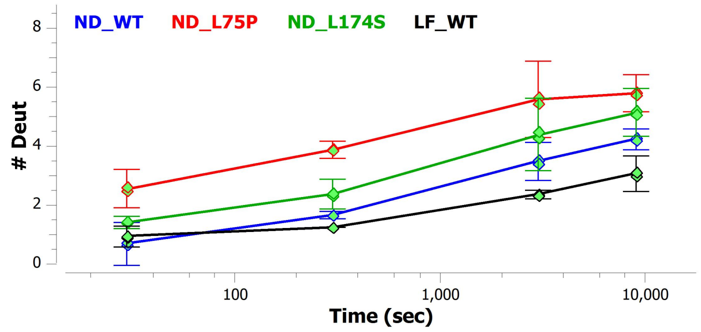

93-104: VKAKVQPYLDDF (#46)

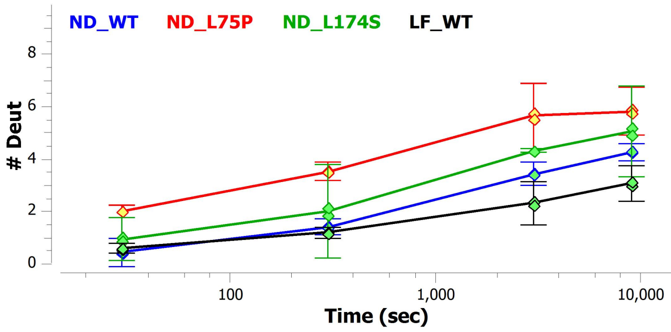

96-103: KVQPYLDD (#47)

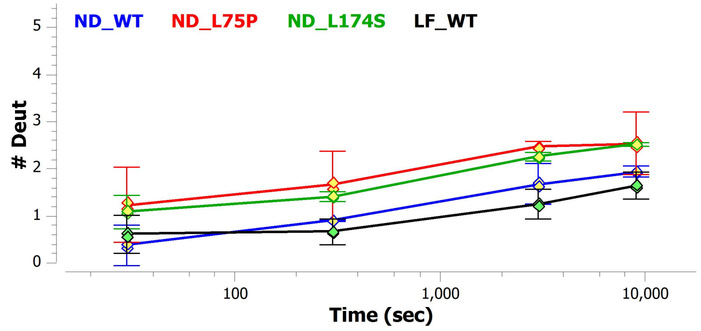

104-111: FQKKWQEE (#48)

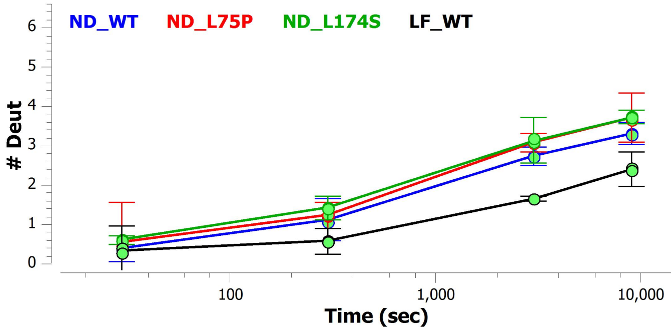

113-124: ELYRQKVEPLRA (#49)

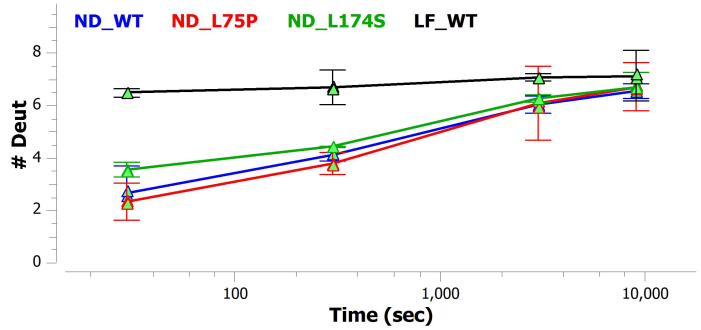

113-126: ELYRQKVEPLRAEL (#50)

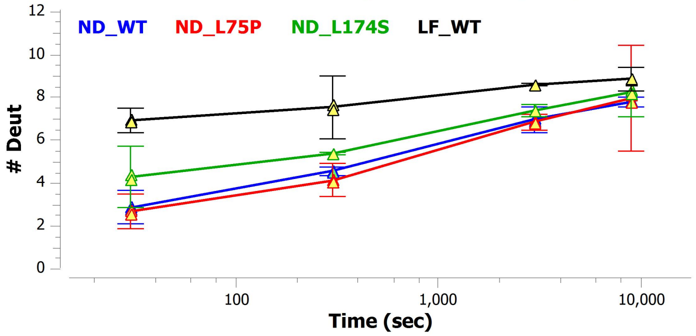

114-124: LYRQKVEPLRA (#51)

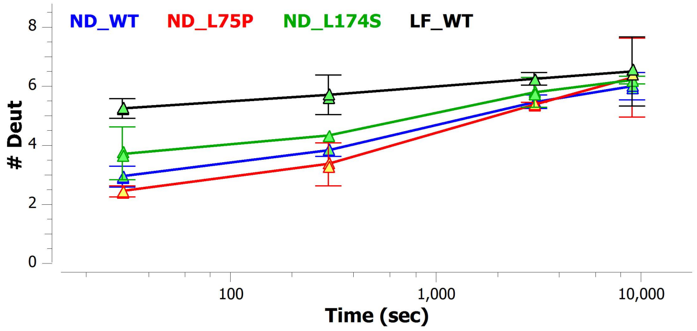

114-125: LYRQKVEPLRAE (#52)

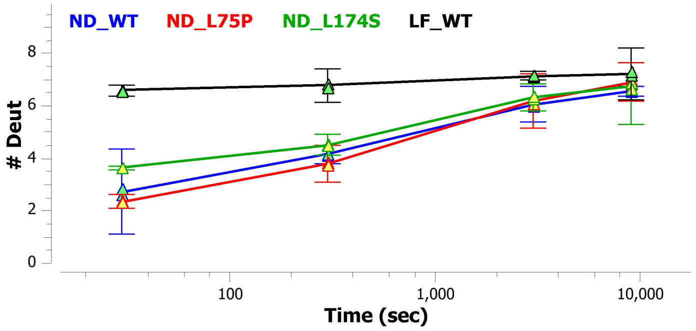

114-126: LYRQKVEPLRAEL (#53)

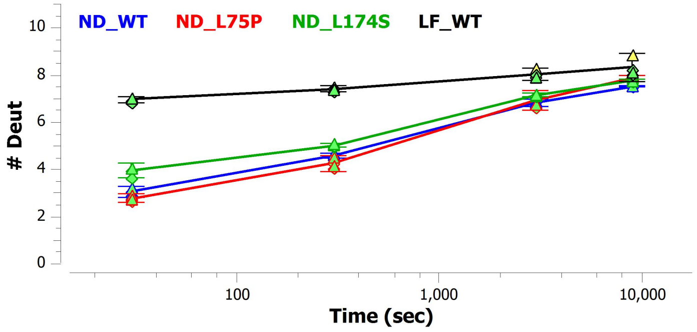

115-124: YRQKVEPLRA (#54)

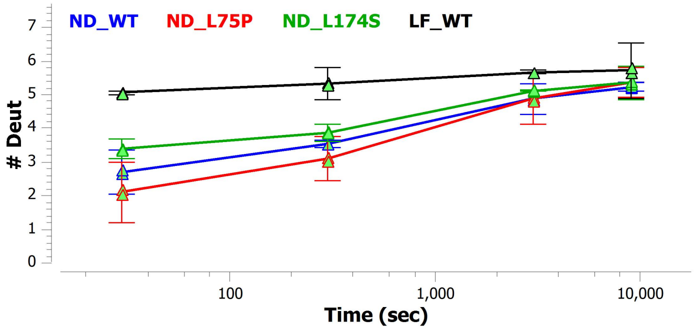

115-126: YRQKVEPLRAEL (#55)

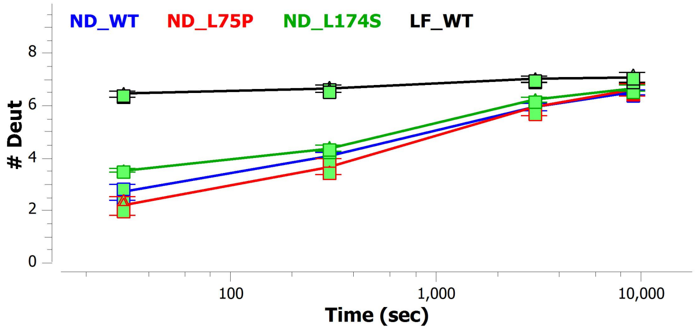

115-147: YRQKVEPLRAELQEGARQKLHELQEKLSPLGEE (#56)

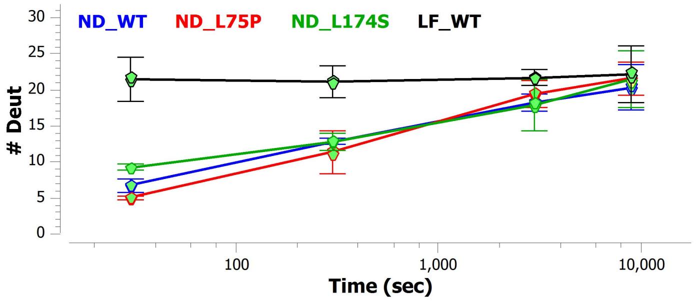

115-158: QKVEPLRAELQEGARQKLHELQEKLSPLGEEMRDRARAHV (#57)

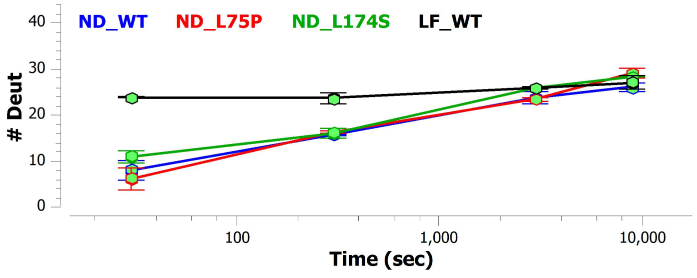

125-136: ELQEGARQKLHE (#58)

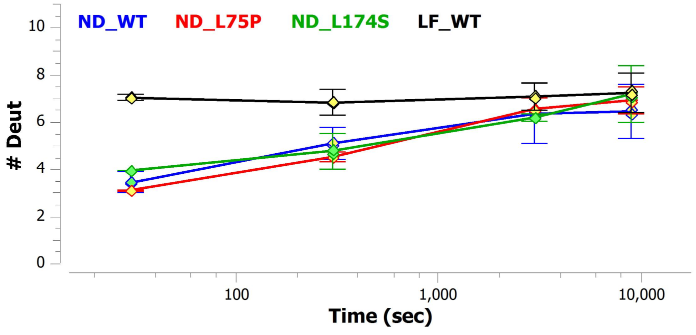

125-139: ELQEGARQKLHELQE (#59)

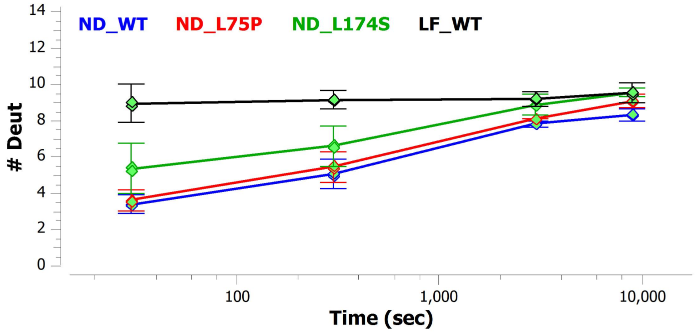

125-146: ELQEGARQKLHELQEKLSPLGE (#60)

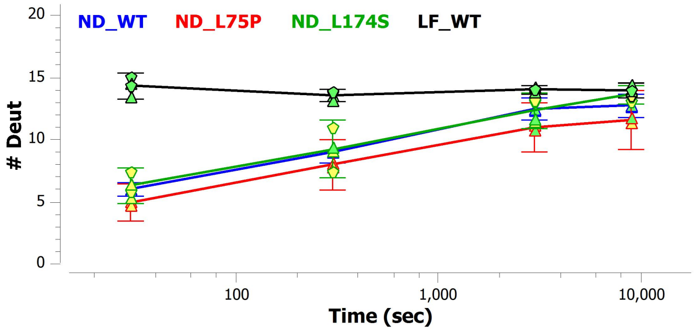

125-148: ELQEGARQKLHELQEKLSPLGEEM (#61)

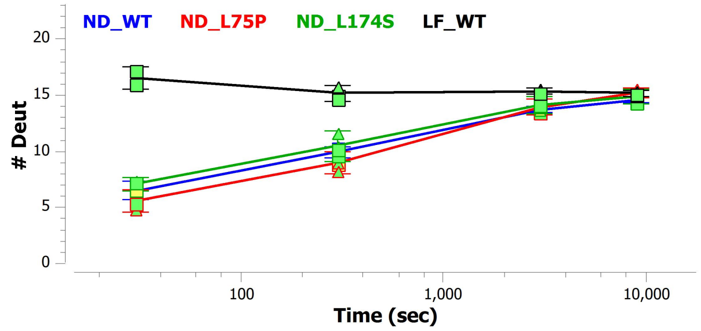

125-158: ELQEGARQKLHELQEKLSPLGEEMRDRARAHVDA (#62)

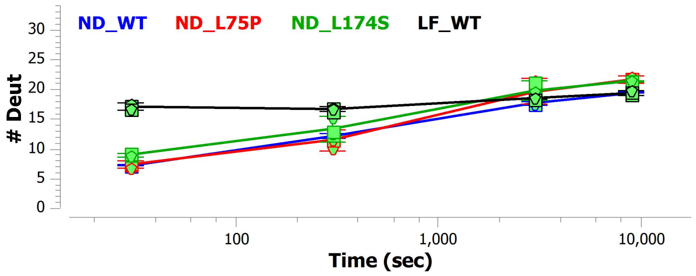

125-159: ELQEGARQKLHELQEKLSPLGEEMRDRARAHVDAL (#63)

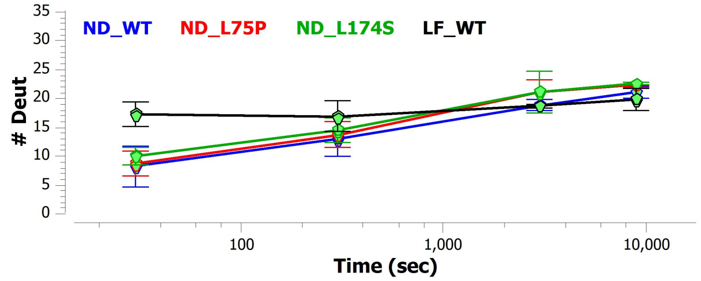

126-158: LQEGARQKLHELQEKLSPLGEEMRDRARAHVDA (#64)

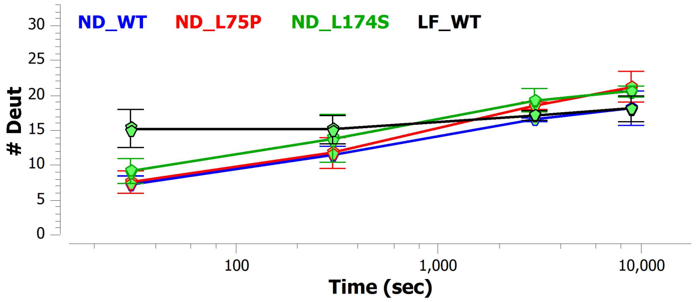

127-139: QEGARQKLHELQE (#65)

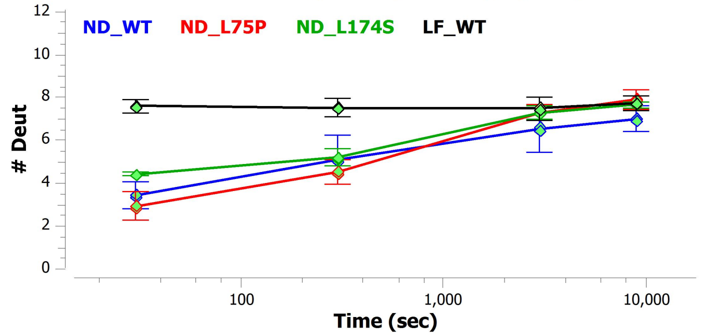

127-146: QEGARQKLHELQEKLSPLGE (#66)

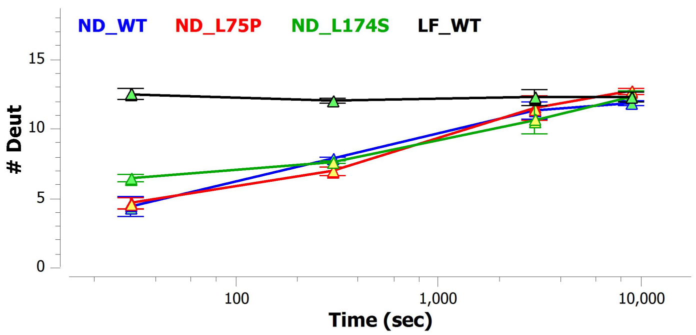

127-147: QEGARQKLHELQEKLSPLGEE (#67)

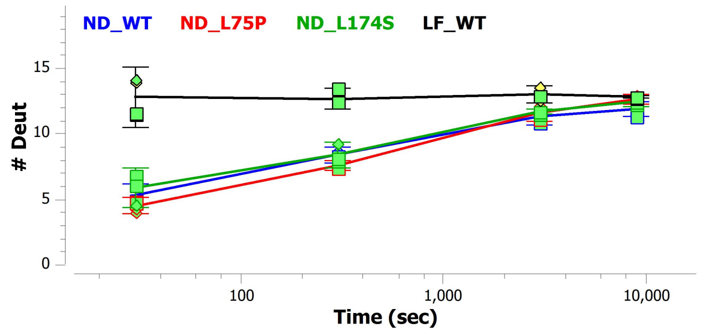

127-154: QEGARQKLHELQEKLSPLGEEMRDRARA (#68)

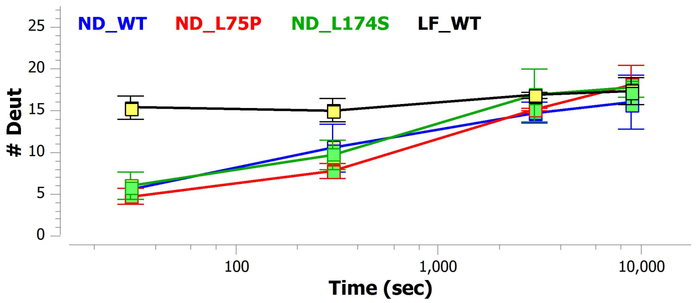

127-158: QEGARQKLHELQEKLSPLGEEMRDRARAHVDA (#69)

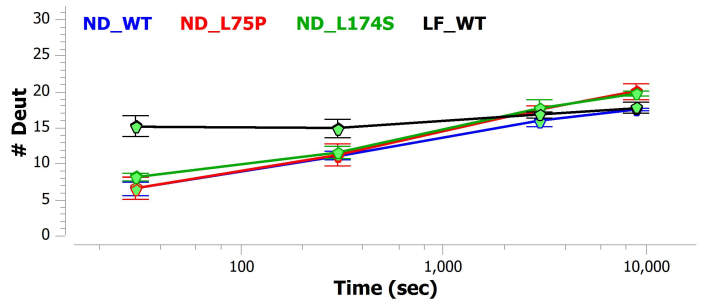

127-159: QEGARQKLHELQEKLSPLGEEMRDRARAHVDAL (#70)

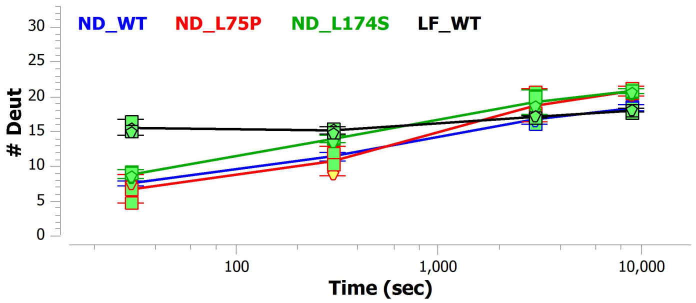

137-146: LQEKLSPLGE (#71)

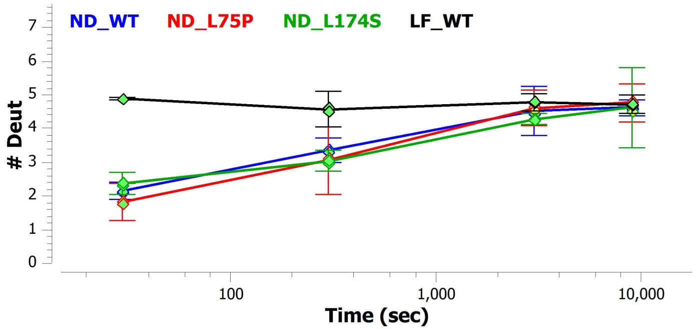

137-147: LQEKLSPLGEE (#72)

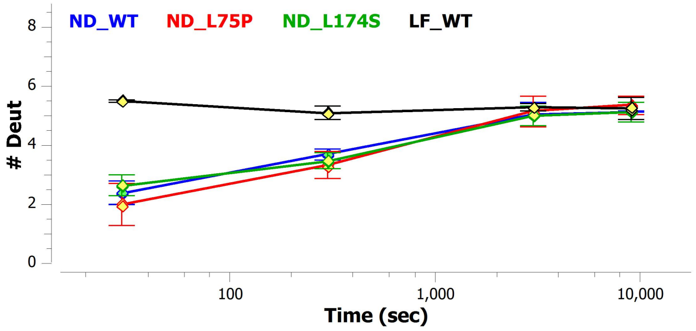

137-158: LQEKLSPLGEEMRDRARAHVDA (#73)

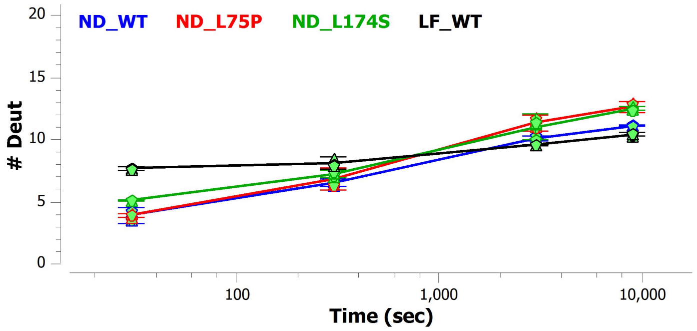

137-159: LQEKLSPLGEEMRDRARAHVDAL (#74)

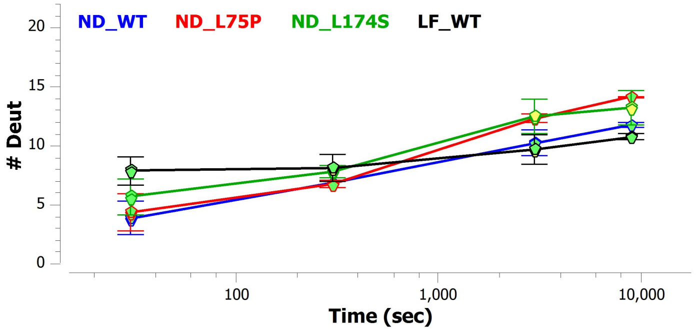

139-158: EKLSPLGEEMRDRARAHVDA (#75)

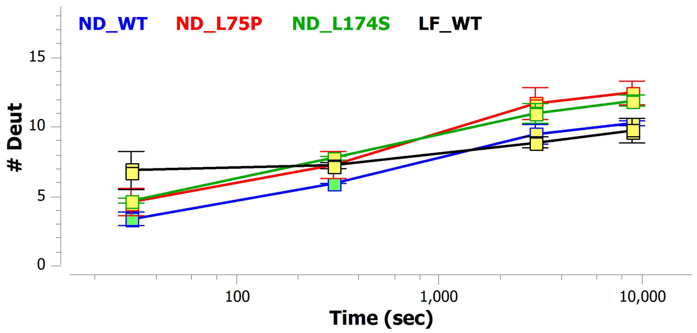

140-146: KLSPLGE (#76)

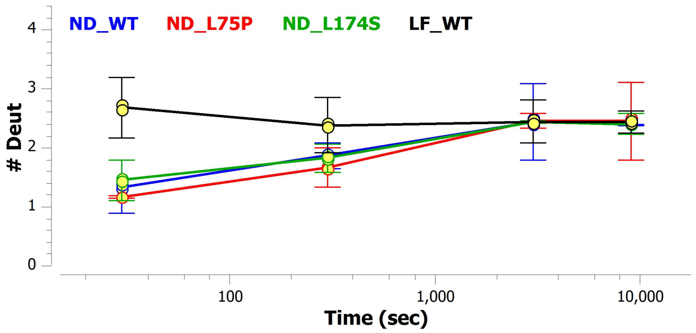

140-147: KLSPLGEE (#77)

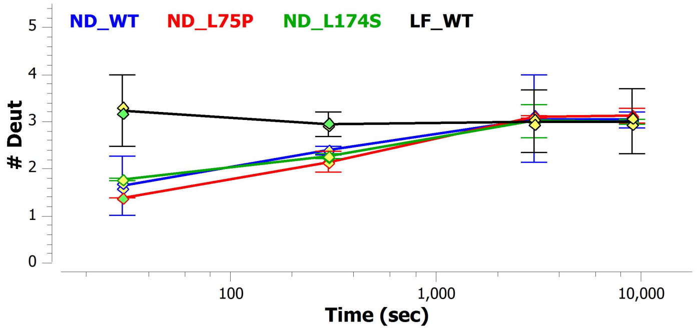

140-148: KLSPLGEEM (#78)

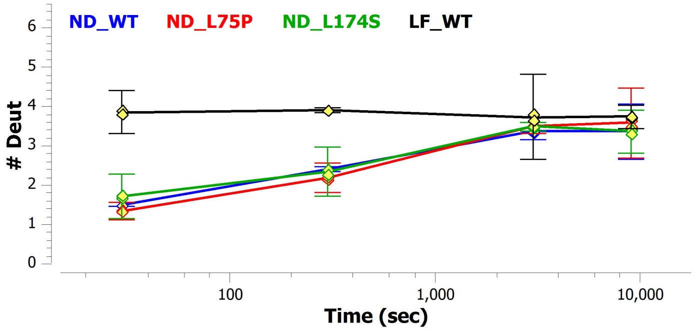

147-158: EMRDRARAHVDA (#79)

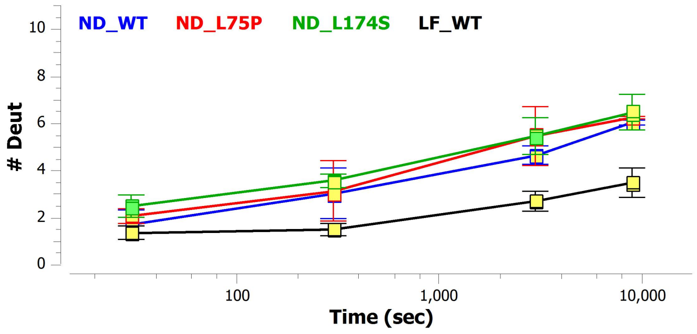

147-159: EMRDRARAHVDAL (#80)

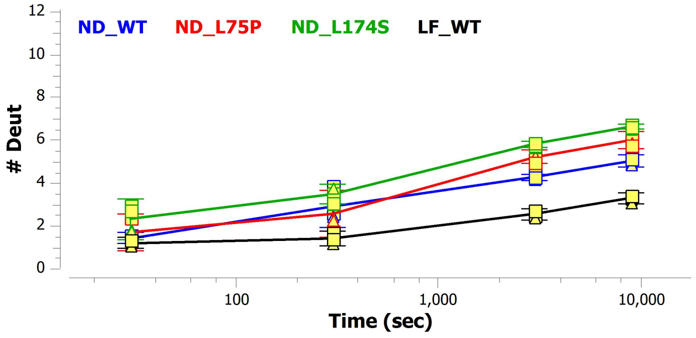

147-169: EMRDRARAHVDALRTHLAPYSDE (#81)

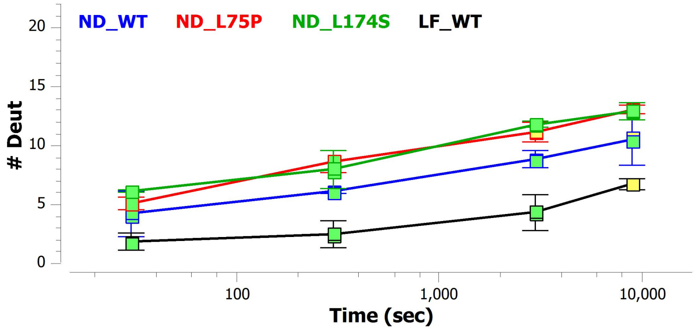

148-158: MRDRARAHVDA (#82)

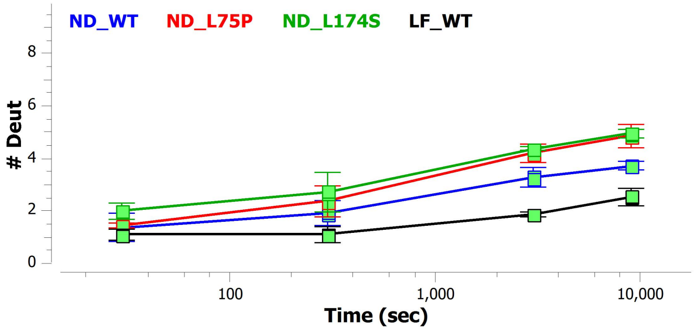

148-159: MRDRARAHVDAL (#83)

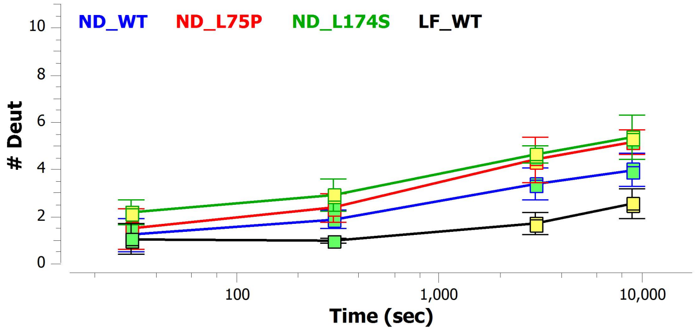

148-169: MRDRARAHVDALRTHLAPYSDE (#84)

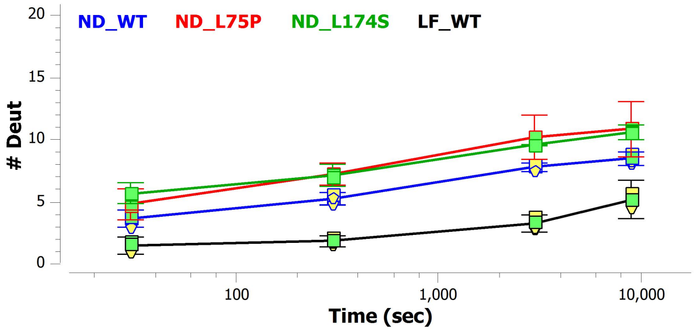

149-158: RDRARAHVDA (#85)

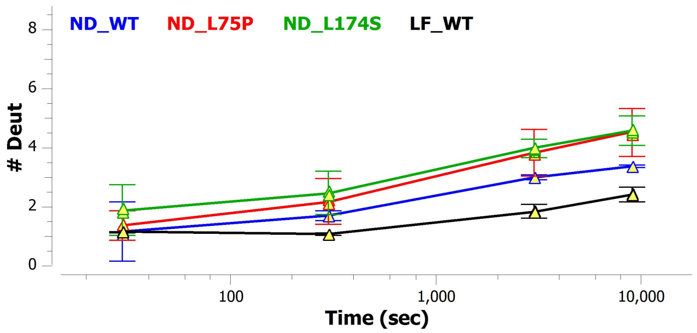

155-169: HVDALRTHLAPYSDE (#86)

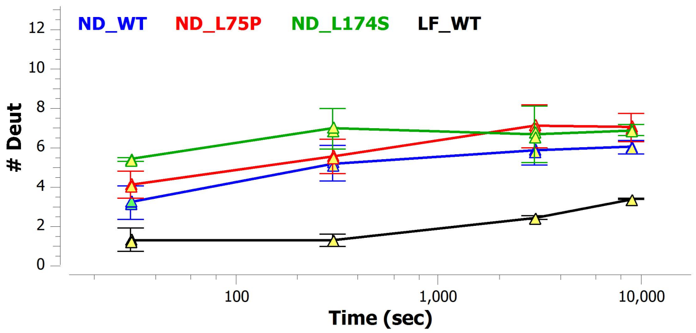

155-170: HVDALRTHLAPYSDEL (#87)

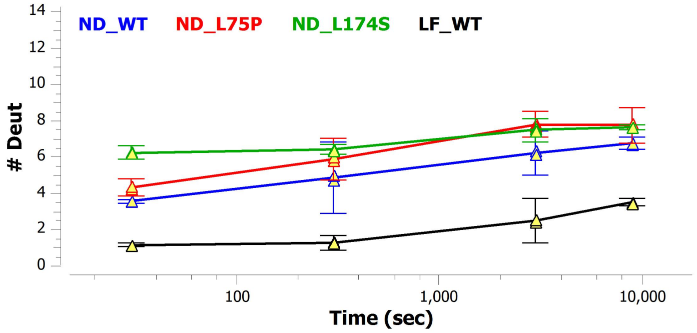

159-168: LRTHLAPYSD (#88)

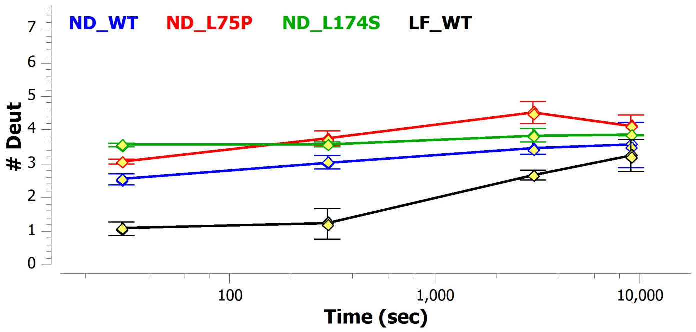

159-169: LRTHLAPYSDE (#89)

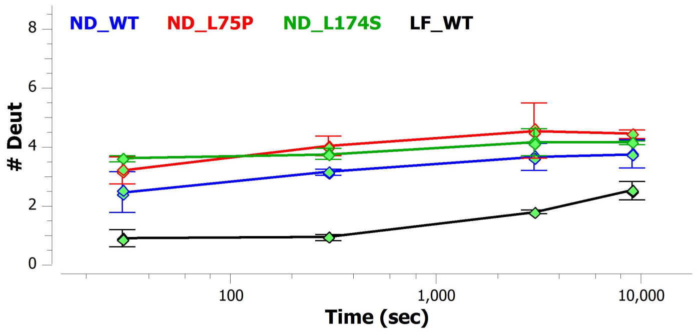

159-170: LRTHLAPYSDEL (#90)

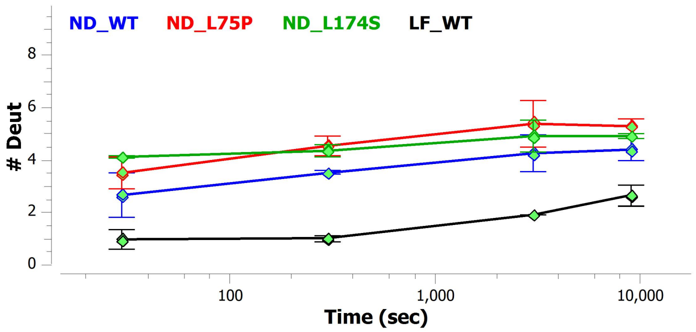

159-173: LRTHLAPYSDELRQR (#91)

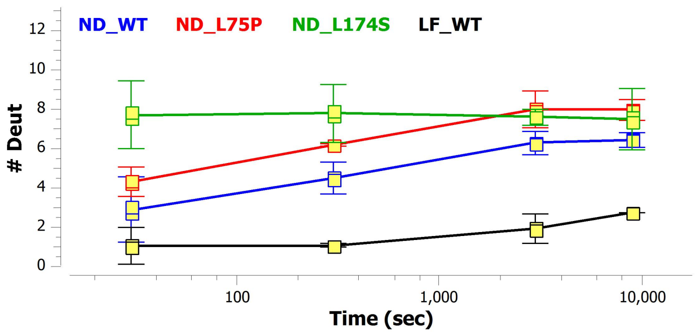

159-174: LRTHLAPYSDELRQRX (#92)

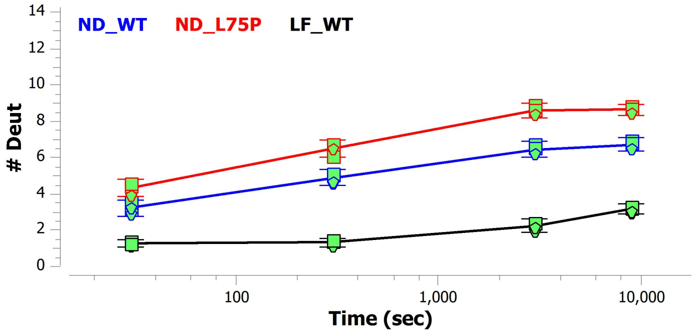

159-176: LRTHLAPYSDELRQRXAA (#93)

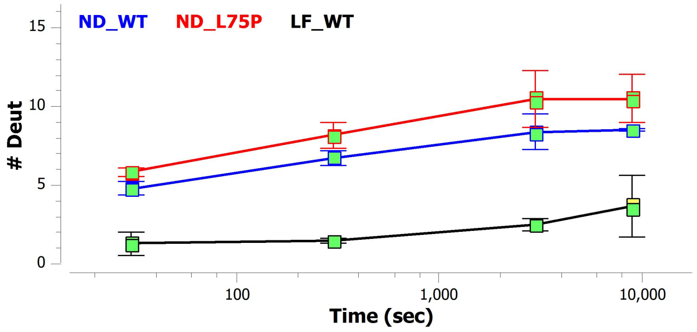

159-178: LRTHLAPYSDELRQRXAARL (#94)

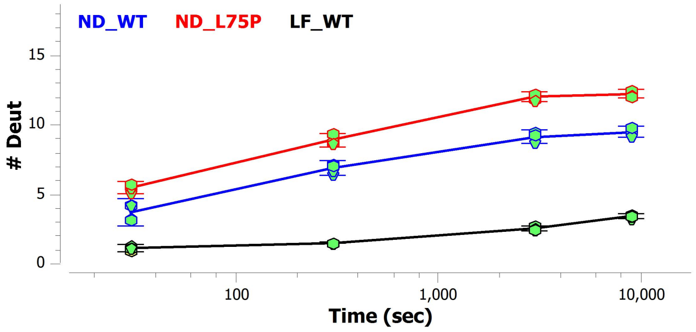

159-180: LRTHLAPYSDELRQRXAARLEA (#95)

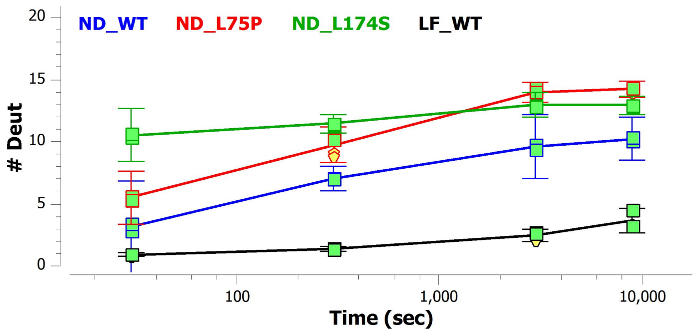

160-169: RTHLAPYSDE (#96)

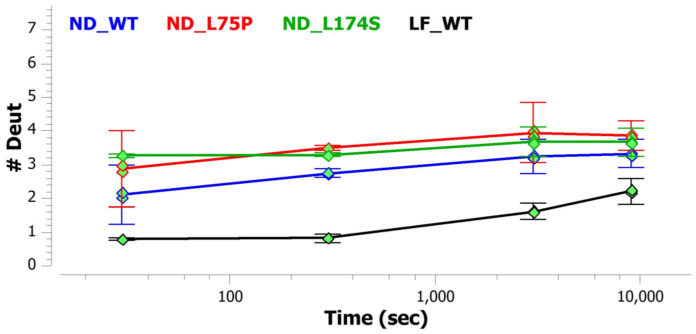

160-174: RTHLAPYSDELRQRX (#97)

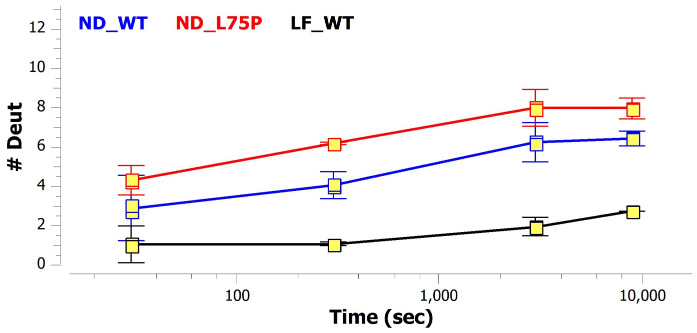

170-176: LRQRXAA (#98)

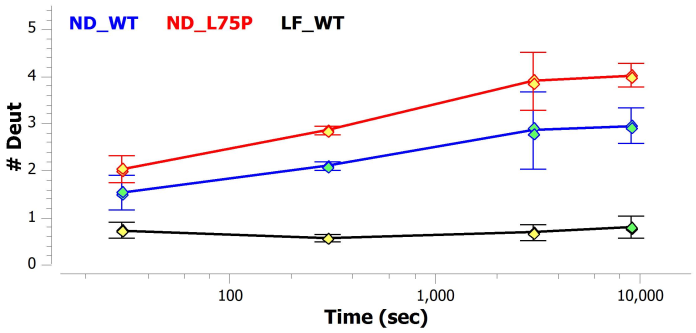

170-177: LRQRXAAR (#99)

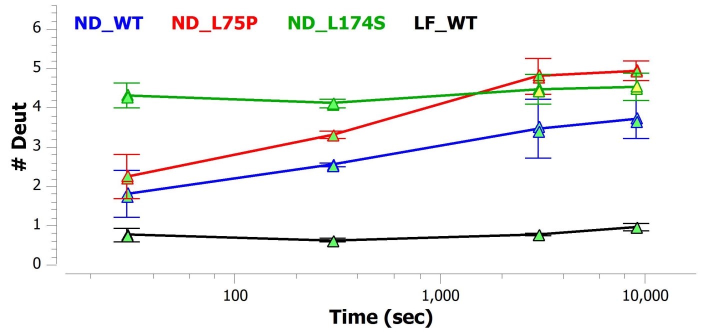

170-178: LRQRXAARL (#100)

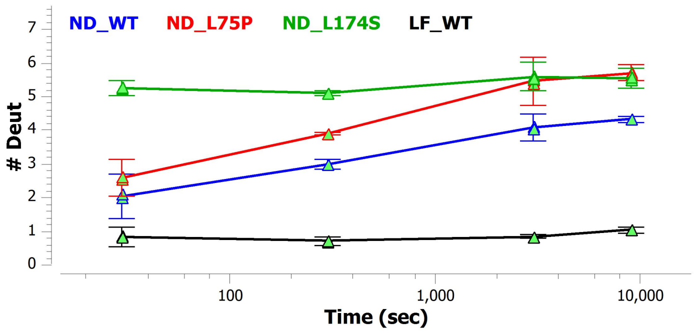

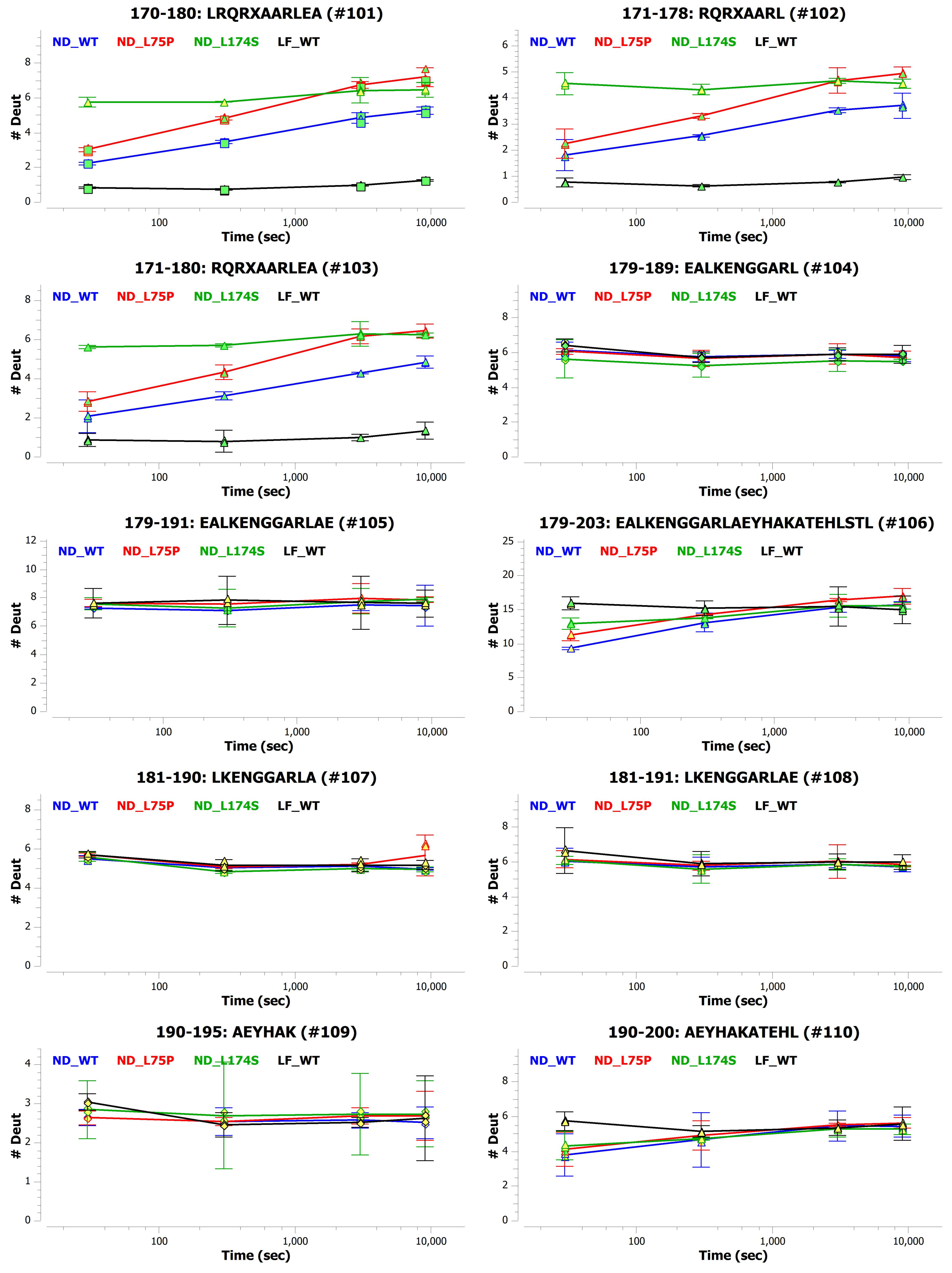
170-180: LRQRXAARLEA (#101)
171-178: RQRXAARL (#102)
171-180: RQRXAARLEA (#103)
179-189: EALKENGGARL (#104)
179-191: EALKENGGARLAE (#105)
179-203: EALKENGGARLAEYHAKATEHLSTL (#106)
181-190: LKENGGARLA (#107)
181-191: LKENGGARLAE (#108)
190-195: AEYHAK (#109)
190-200: AEYHAKATEHL (#110)
ND_WT
ND_L75P
ND_L174S
LF_WT
Deut
Time (sec)
100
1,000
10,000

190-201: AEYHAKATEHLS (#111)

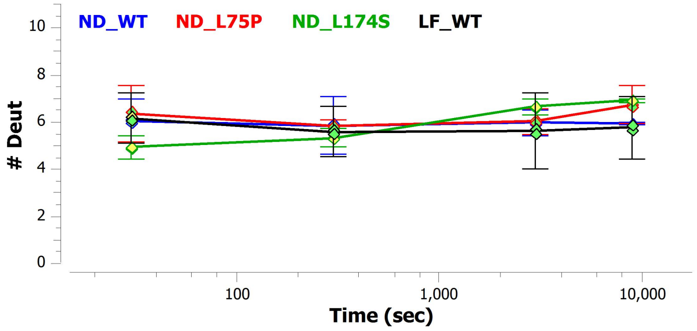

190-202: AEYHAKATEHLST (#112)

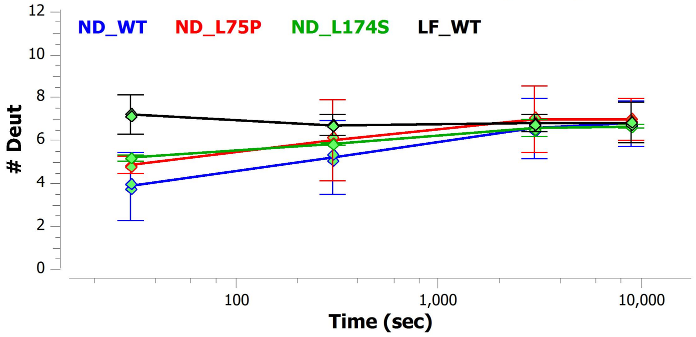

190-203: AEYHAKATEHLSTL (#113)

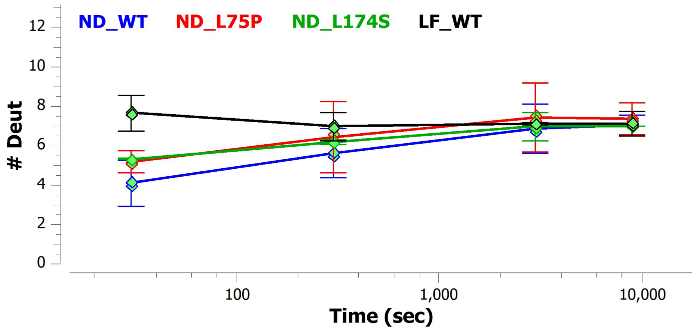

190-205: AEYHAKATEHLSTLSE (#114)

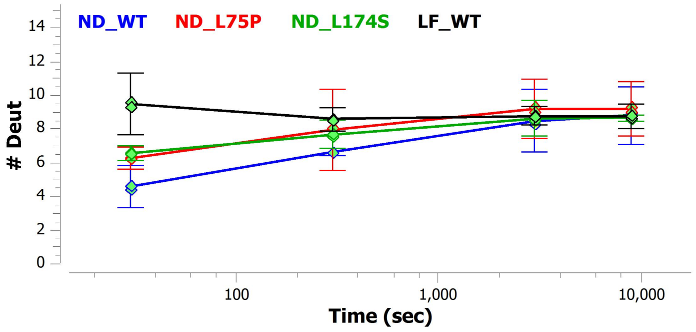

190-211: AEYHAKATEHLSTLSEKAKPAL (#115)

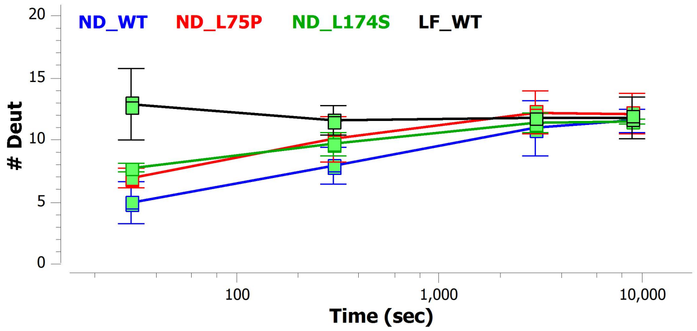

190-212: AEYHAKATEHLSTLSEKAKPALE (#116)

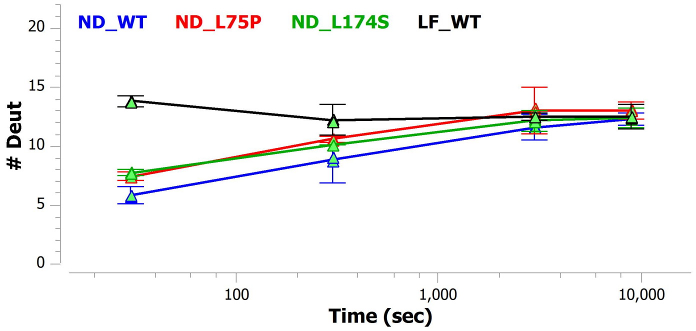

190-213: AEYHAKATEHLSTLSEKAKPALED (#117)

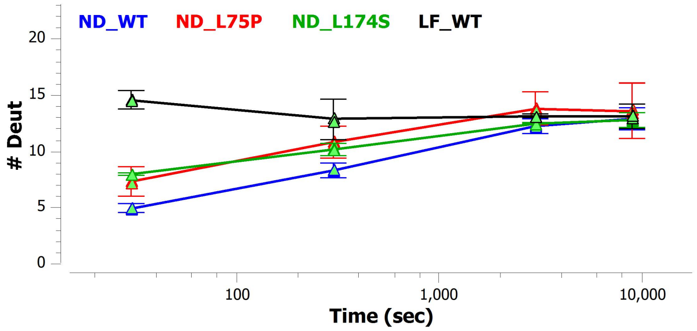

190-214: AEYHAKATEHLSTLSEKAKPALEDL (#118)

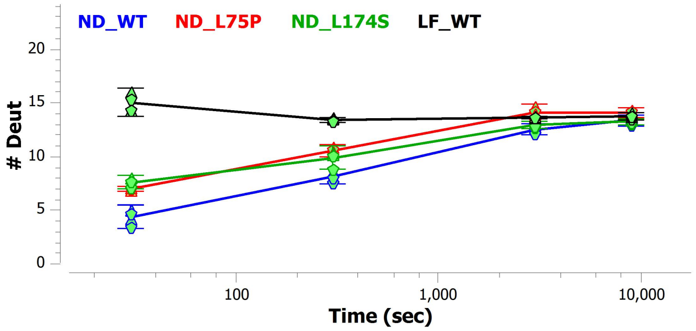

190-219: AEYHAKATEHLSTLSEKAKPALEDLRQGLL (#119)

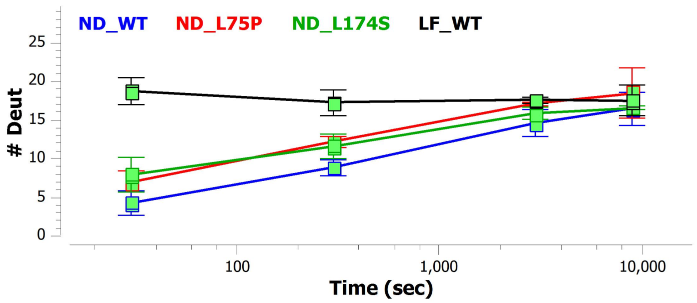

190-222: AEYHAKATEHLSTLSEKAKPALEDLRQGLLPVL (#120)

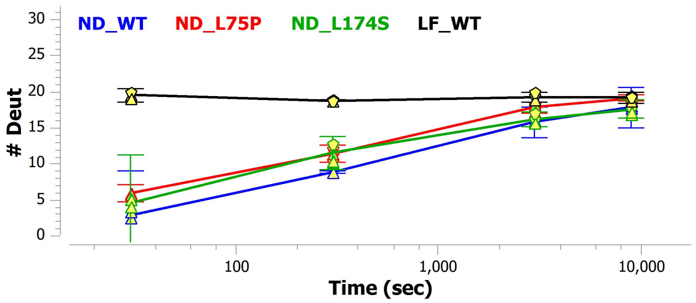

192-203: YHAKATEHLSTL (#121)

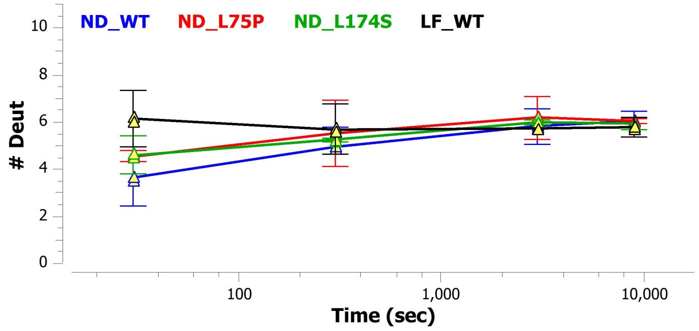

192-211: YHAKATEHLSTLSEKAKPAL (#122)

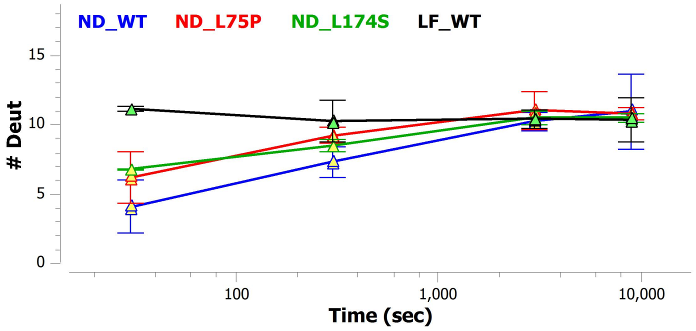

193-211: HAKATEHLSTLSEKAKPAL (#123)

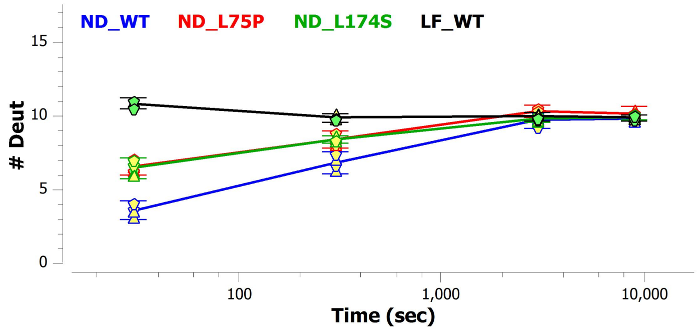

199-211: HLSTLSEKAKPAL (#124)

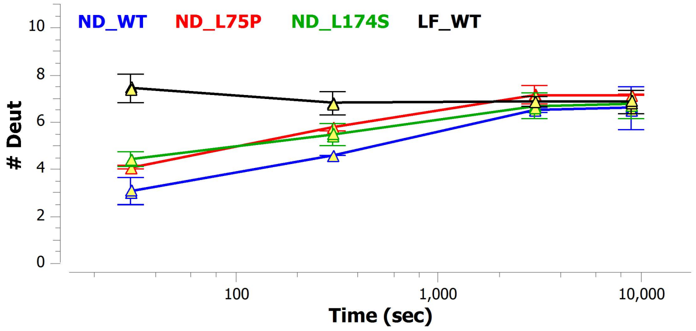

201-212: STLSEKAKPALE (#125)

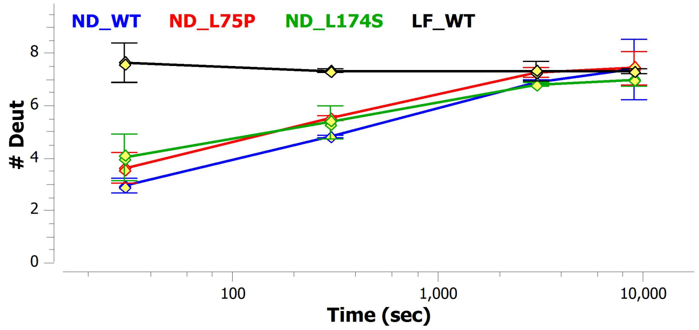

201-219: STLSEKAKPALEDLRQGLL (#126)

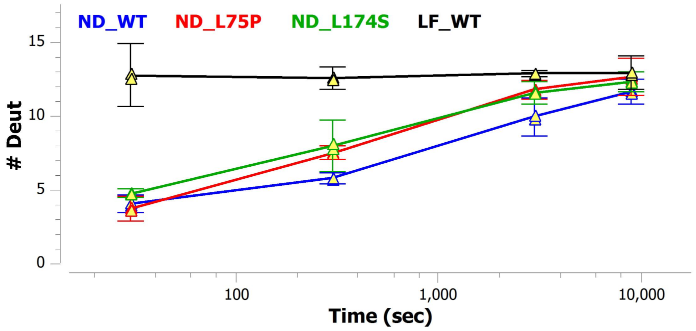

203-212: LSEKAKPALE (#127)

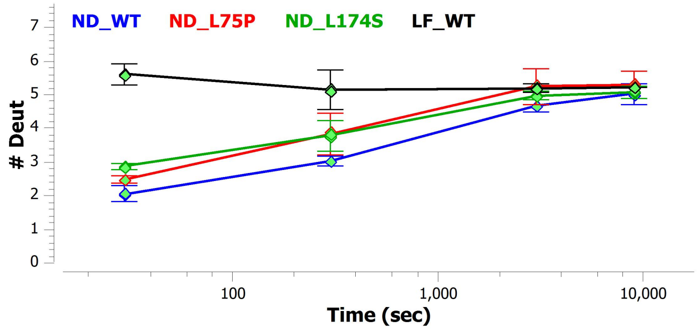

203-213: LSEKAKPALED (#128)

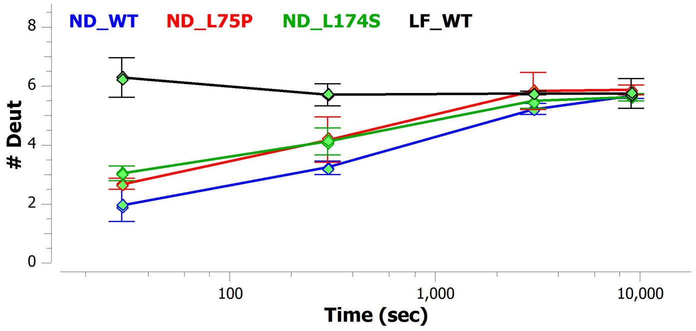

203-214: LSEKAKPALEDL (#129)

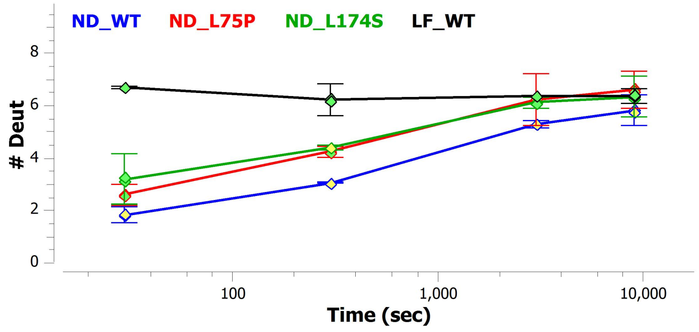

203-219: LSEKAKPALEDLRQGLL (#130)

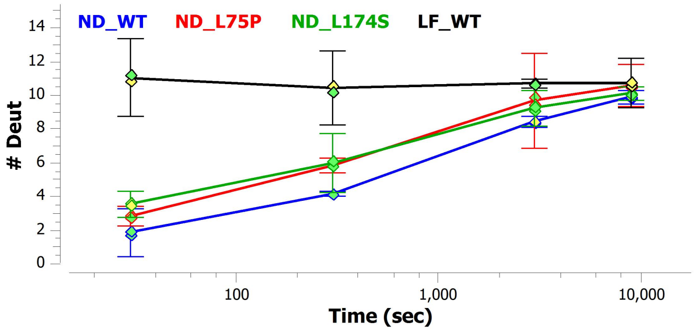

203-222: LSEKAKPALEDLRQGLLPVL (#131)

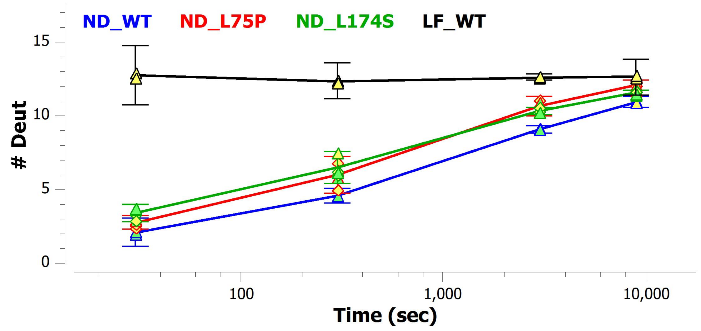

204-211: SEKAKPAL (#132)

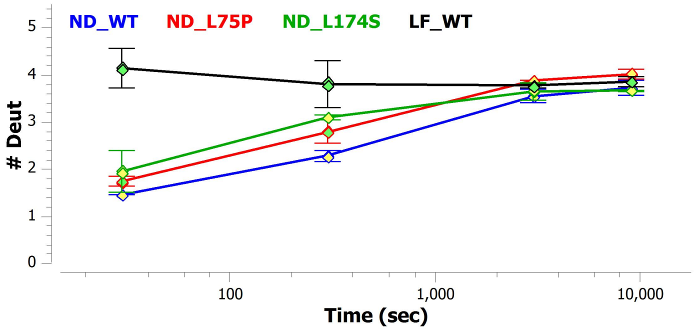

204-212: SEKAKPALE (#133)

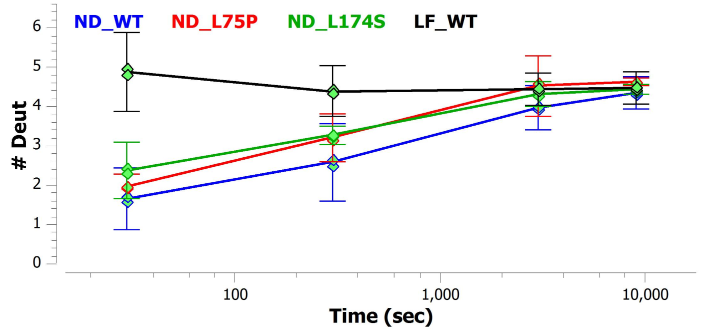

204-213: SEKAKPALED (#134)

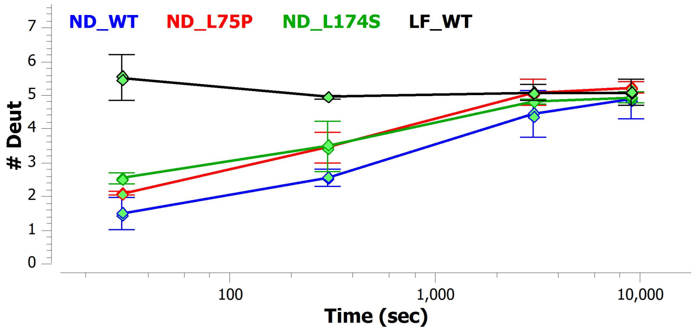

204-214: SEKAKPALEDL (#135)

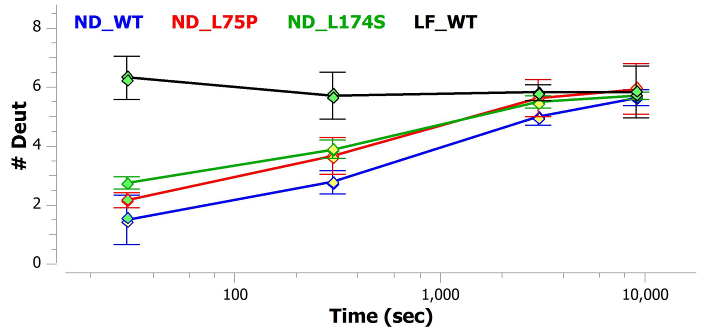

204-219: SEKAKPALEDLRQGLL (#136)

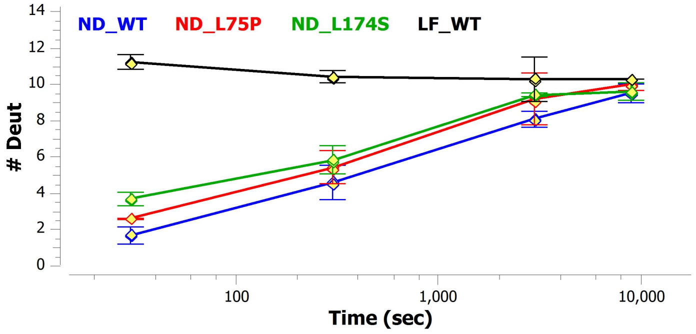

204-222: SEKAKPALEDLRQGLLPVL (#137)

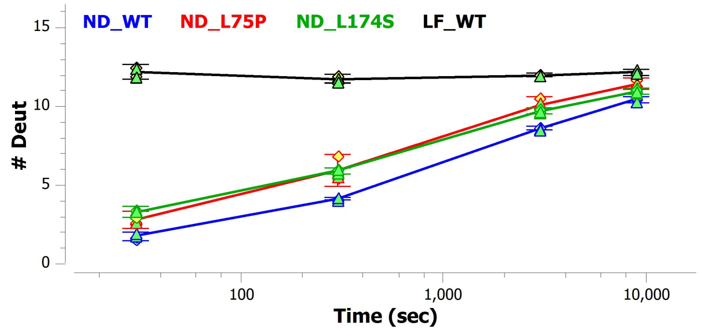

206-222: KAKPALEDLRQGLLPVL (#138)

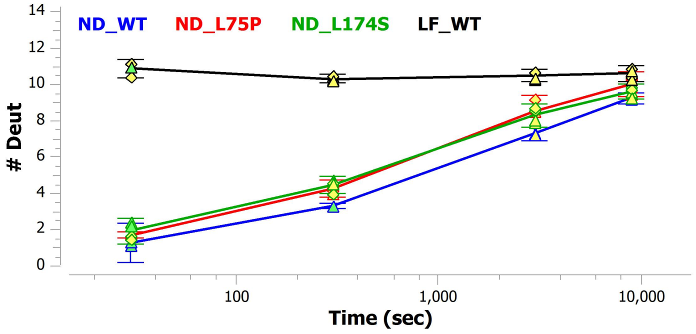

212-219: EDLRQGLL (#139)

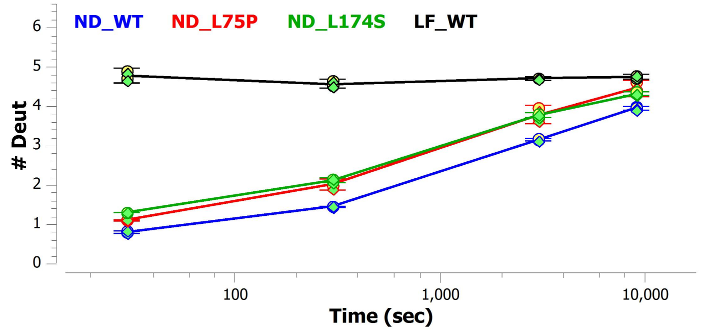

212-222: EDLRQGLLPVL (#140)

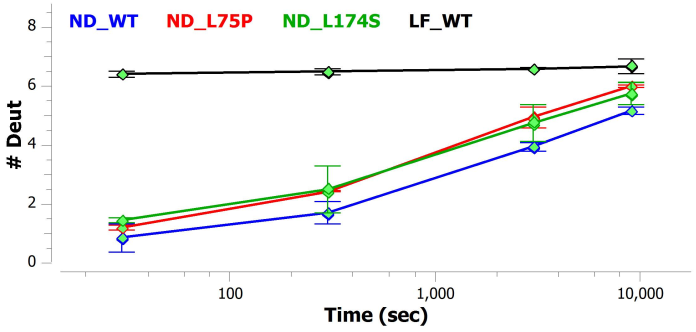

213-219: DLRQGLL (#141)

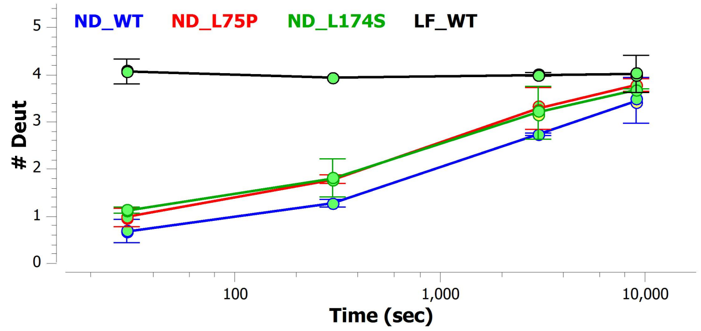

213-222: DLRQGLLPVL (#142)

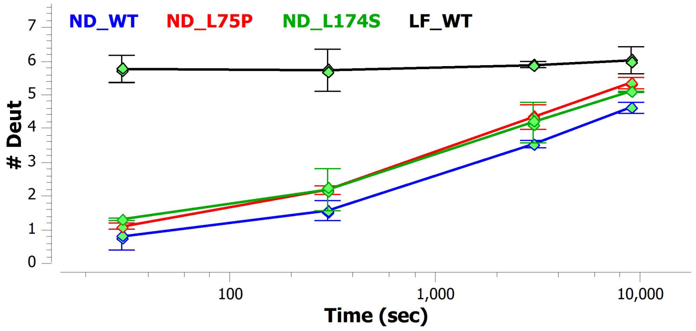

214-222: LRQGLLPVL (#143)

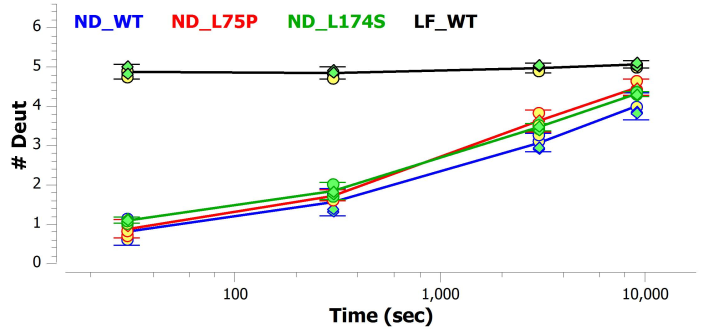

215-222: RQGLLPVL (#144)

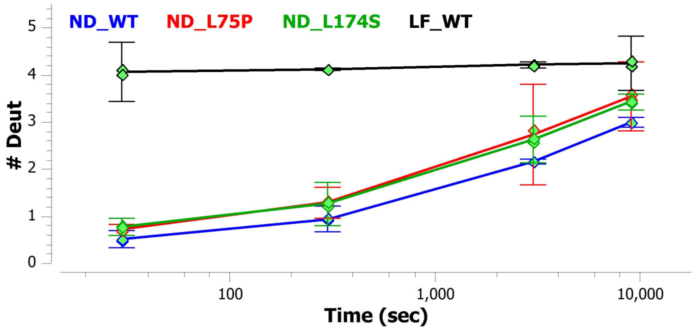

223-229: ESFKVSF (#145)

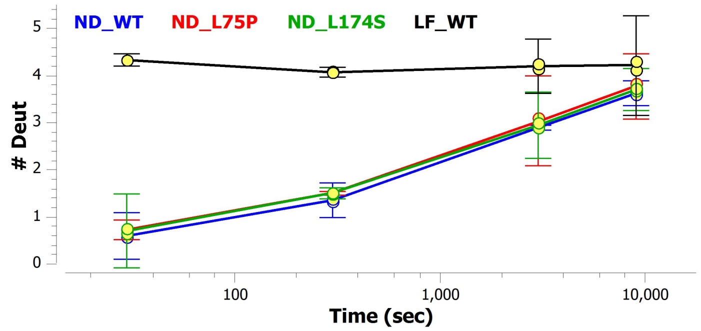

226-232: KVSFLSA (#146)

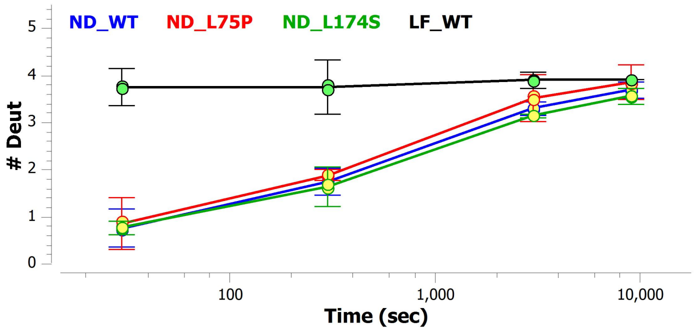

230-235: LSALEE (#147)

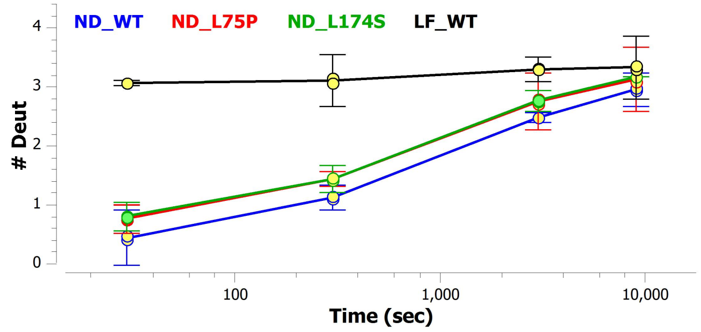

233-243: LEEYTKKLNTQ (#148)

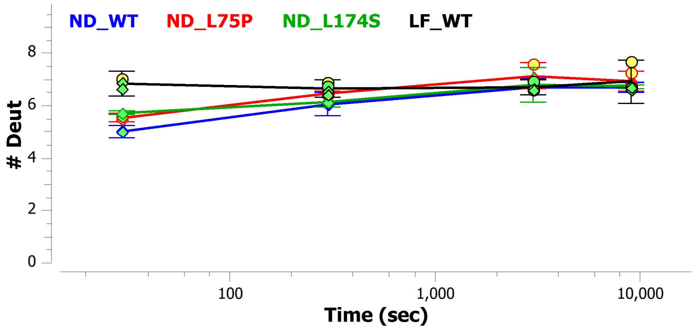

234-243: EEYTKKLNTQ (#149)

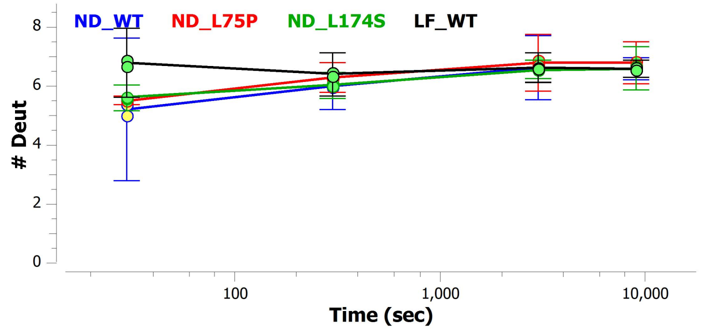

236-243: YTKKLNTQ (#150)

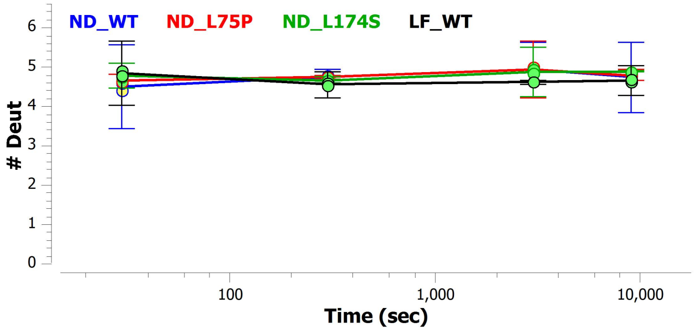

Supplement: Supplemental Data File 1 [file mmc2.pdf]
