# Supplementary material for: Structure dynamics of ApoA-I amyloidogenic variants in small HDL increase their ability to mediate cholesterol efflux
Source: J Lipid Res. 2020 Nov 24;62:100004. doi: 10.1194/jlr.RA120000920 (PMC7890215; doi:10.1194/jlr.RA120000920)
Supplement: Supplemental Data File 2 [file mmc3.pdf]

Supplementary data file S2

Protein State	Protein	Start	End	Sequence	Peptide Mass	RT (min)	Deut Time (sec)	maxD	Theor #D	#D	%D	Conf Interval (#D)	#Rep	Confidence	Stddev	p
ND_WT		17	28	VYVDVLKDSGRD	1364.6936	3.3178	0	10	0	0	0	n/a	2	High	0	
ND_WT		17	28	VYVDVLKDSGRD	1364.6936	3.319	30	10	0.164	1.423	7.117	0.579	2	High	0.064	0.0334232
ND_WT		17	28	VYVDVLKDSGRD	1364.6936	3.3171	300	10	1.45	2.323	11.615	0.065	2	High	0.007	0.0224088
ND_WT		17	28	VYVDVLKDSGRD	1364.6936	3.3161	3000	10	6.286	3.62	18.098	0.396	2	High	0.044	0.132062
ND_WT		17	28	VYVDVLKDSGRD	1364.6936	3.3134	9000	10	8.411	4.159	20.795	0.156	2	High	0.017	0.221157
ND_WT		17	46	VYVDVLKDSGRDYVSQFEGSALGKQLNLKL	3340.7667	5.3123	0	28	0	0	0	n/a	2	Medium	0	
ND_WT		17	46	VYVDVLKDSGRDYVSQFEGSALGKQLNLKL	3340.7667	5.3331	30	28	0.322	3.162	2.259	5.078	2	High	0.565	0.107189
ND_WT		17	46	VYVDVLKDSGRDYVSQFEGSALGKQLNLKL	3340.7667	5.298	300	28	2.951	7.826	5.59	0.924	2	Medium	0.103	0.0839254
ND_WT		17	46	VYVDVLKDSGRDYVSQFEGSALGKQLNLKL	3340.7667	5.2836	3000	28	16.007	13.291	9.494	1.036	2	Medium	0.115	0.0955085
ND_WT		17	46	VYVDVLKDSGRDYVSQFEGSALGKQLNLKL	3340.7667	5.2786	9000	28	23.684	14.709	10.507	1.044	2	High	0.116	0.25228
ND_WT		18	28	YVDVLKDSGRD	1265.6252	3.0655	0	9	0	0	0	n/a	2	High	0	
ND_WT		18	28	YVDVLKDSGRD	1265.6252	3.0433	30	9	0.163	1.468	8.153	0.351	2	High	0.039	0.00997418
ND_WT		18	28	YVDVLKDSGRD	1265.6252	3.0565	300	9	1.432	2.306	12.812	0.017	2	High	0.002	0.0211004
ND_WT		18	28	YVDVLKDSGRD	1265.6252	3.0528	3000	9	6.117	3.49	19.391	0.296	2	High	0.033	0.111086
ND_WT		18	28	YVDVLKDSGRD	1265.6252	3.0629	9000	9	7.986	3.92	21.778	0.073	2	High	0.008	0.189048
ND_WT		19	28	VDVLKDSGRD	1102.5619	2.317	0	8	0	0	0	n/a	2	High	0	
ND_WT		19	28	VDVLKDSGRD	1102.5619	2.3252	30	8	0.145	1.46	9.125	0.647	2	High	0.072	0.0120458
ND_WT		19	28	VDVLKDSGRD	1102.5619	2.3255	300	8	1.269	2.277	14.232	0.124	2	High	0.014	0.0230998
ND_WT		19	28	VDVLKDSGRD	1102.5619	2.3144	3000	8	5.286	3.357	20.983	0.333	2	High	0.037	0.064074
ND_WT		19	28	VDVLKDSGRD	1102.5619	2.3196	9000	8	6.991	3.761	23.506	0.208	2	High	0.023	0.101523
ND_WT		19	44	VDVLKDSGRDYVSQFEGSALGKQLNL	2837.4559	5.1893	0	24	0	0	0	n/a	2	Medium	0	
ND_WT		19	44	VDVLKDSGRDYVSQFEGSALGKQLNL	2837.4559	5.139	30	24	0.295	3.089	3.218	0.933	2	Medium	0.104	0.93663
ND_WT		19	44	VDVLKDSGRDYVSQFEGSALGKQLNL	2837.4559	5.1423	300	24	2.694	6.47	6.739	0.617	2	High	0.069	0.0541228
ND_WT		19	44	VDVLKDSGRDYVSQFEGSALGKQLNL	2837.4559	5.126	3000	24	14.365	11.731	12.22	0.471	2	High	0.052	0.0383277
ND_WT		19	44	VDVLKDSGRDYVSQFEGSALGKQLNL	2837.4559	5.0997	9000	24	20.902	13.012	13.554	0.979	2	High	0.109	0.0621467
ND_WT		20	28	DVLKDSGRD	1003.4934	2.1264	0	7	0	0	0	n/a	2	Medium	0	
ND_WT		20	28	DVLKDSGRD	1003.4934	2.3679	30	7	0.14	1.198	8.557	0.299	2	Medium	0.033	0.0117774
ND_WT		20	28	DVLKDSGRD	1003.4934	2.3453	300	7	1.219	2.093	14.953	0.275	2	Medium	0.031	0.00641933
ND_WT		20	28	DVLKDSGRD	1003.4934	2.3391	3000	7	4.883	3.015	21.536	0.01	2	Medium	0.001	0.0620542
ND_WT		20	28	DVLKDSGRD	1003.4934	2.3471	9000	7	6.203	3.412	24.37	0.247	2	Medium	0.028	0.0627528
ND_WT		29	37	YVSQFEGSA	986.4345	3.2536	0	7	0	0	0	n/a	2	High	0	
ND_WT		29	37	YVSQFEGSA	986.4345	3.2546	30	7	0.086	1.83	26.14	0.687	2	High	0.076	0.237
ND_WT		29	37	YVSQFEGSA	986.4345	3.2499	300	7	0.805	2.533	36.19	0.25	2	High	0.028	0.150875
ND_WT		29	37	YVSQFEGSA	986.4345	3.2756	3000	7	4.753	3.551	50.733	0.21	2	High	0.023	0.0109214
ND_WT		29	37	YVSQFEGSA	986.4345	3.2617	9000	7	6.655	3.703	52.904	0.309	2	High	0.034	0.515452
ND_WT		29	38	YVSQFEGSAL	1099.5186	4.2697	0	8	0	0	0	n/a	2	High	0	
ND_WT		29	38	YVSQFEGSAL	1099.5186	4.2624	30	8	0.09	2.3	21.724	0.132	4	High	0.083	0.000861199
ND_WT		29	38	YVSQFEGSAL	1099.5186	4.2574	300	8	0.845	3.26	30.534	0.028	4	High	0.018	0.0047627
ND_WT		29	38	YVSQFEGSAL	1099.5186	4.2568	3000	8	5.092	4.208	39.452	0.045	4	High	0.028	0.0032731
ND_WT		29	38	YVSQFEGSAL	1099.5186	4.2676	9000	8	7.366	4.37	41.04	0.065	4	High	0.041	0.221658
ND_WT		29	43	YVSQFEGSALGKQLN	1639.8206	3.9238	0	13	0	0	0	n/a	2	High	0	
ND_WT		29	43	YVSQFEGSALGKQLN	1639.8206	3.8997	30	13	0.131	3.601	13.849	1.334	2	High	0.149	0.036576
ND_WT		29	43	YVSQFEGSALGKQLN	1639.8206	3.8895	300	13	1.239	5.613	21.588	0.121	2	High	0.013	0.449064
ND_WT		29	43	YVSQFEGSALGKQLN	1639.8206	3.8878	3000	13	7.811	7.357	28.296	0.378	2	High	0.042	0.0169495
ND_WT		29	43	YVSQFEGSALGKQLN	1639.8206	3.8963	9000	13	11.753	7.618	29.3	0.402	2	High	0.045	0.333182
ND_WT		29	44	YVSQFEGSALGKQLNL	1752.9046	4.833	0	14	0	0	0	n/a	2	High	0	
ND_WT		29	44	YVSQFEGSALGKQLNL	1752.9046	4.7981	30	14	0.135	3.716	13.272	0.459	2	High	0.051	0.00652589
ND_WT		29	44	YVSQFEGSALGKQLNL	1752.9046	4.8143	300	14	1.285	5.781	20.648	0.24	2	High	0.027	0.0762203
ND_WT		29	44	YVSQFEGSALGKQLNL	1752.9046	4.8143	3000	14	8.184	7.834	27.979	0.502	2	High	0.056	0.179478
ND_WT		29	44	YVSQFEGSALGKQLNL	1752.9046	4.8168	9000	14	12.506	8.131	29.04	0.385	2	High	0.043	0.577192
ND_WT		29	46	YVSQFEGSALGKQLNLKL	1994.0837	4.833	0	16	0	0	0	n/a	2	High	0	
ND_WT		29	46	YVSQFEGSALGKQLNLKL	1994.0837	4.8177	30	16	0.143	4.373	13.667	1.683	2	High	0.187	0.0151495
ND_WT		29	46	YVSQFEGSALGKQLNLKL	1994.0837	4.8262	300	16	1.361	7.06	22.062	0.397	2	High	0.044	0.144309
ND_WT		29	46	YVSQFEGSALGKQLNLKL	1994.0837	4.8143	3000	16	8.826	9.5	29.688	0.424	2	High	0.047	0.0719669
ND_WT		29	46	YVSQFEGSALGKQLNLKL	1994.0837	4.8181	9000	16	13.868	9.78	30.562	0.362	2	High	0.04	0.856784
ND_WT		33	38	FEGSAL	622.2962	3.5886	0	4	0	0	0	n/a	2	Medium	0	
ND_WT		33	38	FEGSAL	622.2962	3.5844	30	4	0.053	1.732	43.292	0.521	2	High	0.058	0.0704477
ND_WT		33	38	FEGSAL	622.2962	3.5812	300	4	0.492	1.997	49.926	0.002	2	High	0	0.423348
ND_WT		33	38	FEGSAL	622.2962	3.5794	3000	4	2.714	2.116	52.903	0.181	2	High	0.02	0.933725
ND_WT		33	38	FEGSAL	622.2962	3.589	9000	4	3.676	2.069	51.732	0.125	2	High	0.014	0.233902
ND_WT		33	43	FEGSALGKQLN	1162.5982	3.2215	0	9	0	0	0	n/a	2	Medium	0	
ND_WT		33	43	FEGSALGKQLN	1162.5982	3.1748	30	9	0.094	3.05	16.944	0.657	2	Medium	0.073	0.0056305
ND_WT		33	43	FEGSALGKQLN	1162.5982	3.1903	300	9	0.886	4.19	23.276	0.453	2	Medium	0.05	0.015954
ND_WT		33	43	FEGSALGKQLN	1162.5982	3.1827	3000	9	5.433	5.008	27.822	0.443	2	Medium	0.049	0.901059
ND_WT		33	43	FEGSALGKQLN	1162.5982	3.1878	9000	9	8.062	5.057	28.095	0.441	2	Medium	0.049	0.889051
ND_WT		33	44	FEGSALGKQLNL	1275.6823	4.4284	0	10	0	0	0	n/a	2	Medium	0	
ND_WT		33	44	FEGSALGKQLNL	1275.6823	4.4187	30	10	0.099	2.975	14.874	1.239	2	Medium	0.138	0.0491268
ND_WT		33	44	FEGSALGKQLNL	1275.6823	4.3956	300	10	0.932	4.473	22.366	0.181	2	Medium	0.02	0.0063684
ND_WT		33	44	FEGSALGKQLNL	1275.6823	4.4084	3000	10	5.806	5.448	27.241	0.259	2	Medium	0.029	0.139937
ND_WT		33	44	FEGSALGKQLNL	1275.6823	4.4069	9000	10	8.815	5.417	27.085	0.883	2	Medium	0.098	0.128294
ND_WT		33	46	FEGSALGKQLNLKL	1516.8613	4.4743	0	12	0	0	0	n/a	2	High	0	
ND_WT		33	46	FEGSALGKQLNLKL	1516.8613	4.4755	30	12	0.106	3.674	15.309	0.676	2	High	0.075	0.00214256
ND_WT		33	46	FEGSALGKQLNLKL	1516.8613	4.4525	300	12	1.008	5.735	23.895	0.091	2	High	0.01	0.0274888
ND_WT		33	46	FEGSALGKQLNLKL	1516.8613	4.4471	3000	12	6.448	7.307	30.447	0.026	2	High	0.003	0.10211
ND_WT		33	46	FEGSALGKQLNLKL	1516.8613	4.4523	9000	12	10.177	7.34	30.583	0.636	2	High	0.071	0.915165

ND_WT		34	43	EGSALGKQLN	1015.5298	2.4444	0	8	0	0	0	n/a	2	High	0	
ND_WT		34	43	EGSALGKQLN	1015.5298	2.4332	30	8	0.08	2.467	15.42	1.268	2	Medium	0.141	0.066064
ND_WT		34	43	EGSALGKQLN	1015.5298	2.4279	300	8	0.757	3.576	22.349	0.003	2	Medium	0	0.0562337
ND_WT		34	43	EGSALGKQLN	1015.5298	2.4285	3000	8	4.685	4.353	27.209	0.144	2	Medium	0.016	0.414157
ND_WT		34	43	EGSALGKQLN	1015.5298	2.4497	9000	8	7.078	4.314	26.964	0.992	2	Medium	0.11	0.24752
ND_WT		34	44	EGSALGKQLNL	1128.6139	3.9514	0	9	0	0	0	n/a	2	Medium	0	
ND_WT		34	44	EGSALGKQLNL	1128.6139	3.9247	30	9	0.085	2.483	13.796	0.441	2	Medium	0.049	0.00552311
ND_WT		34	44	EGSALGKQLNL	1128.6139	3.9132	300	9	0.803	3.942	21.903	0.216	2	Medium	0.024	0.068047
ND_WT		34	44	EGSALGKQLNL	1128.6139	3.9376	3000	9	5.058	4.907	27.263	0.03	2	Medium	0.003	0.00164124
ND_WT		34	44	EGSALGKQLNL	1128.6139	3.9239	9000	9	7.831	5.101	28.338	0.257	2	Medium	0.029	0.0740867
ND_WT		34	46	EGSALGKQLNLKL	1369.7929	4.1481	0	11	0	0	0	n/a	2	High	0	
ND_WT		34	46	EGSALGKQLNLKL	1369.7929	4.1212	30	11	0.093	3.211	14.596	0.528	2	High	0.059	0.0110339
ND_WT		34	46	EGSALGKQLNLKL	1369.7929	4.1237	300	11	0.88	5.005	22.752	0.17	2	High	0.019	0.0574154
ND_WT		34	46	EGSALGKQLNLKL	1369.7929	4.1207	3000	11	5.7	6.445	29.297	0.149	2	High	0.017	0.0413708
ND_WT		34	46	EGSALGKQLNLKL	1369.7929	4.1279	9000	11	9.193	6.492	29.508	0.418	2	High	0.047	0.660319
ND_WT		36	46	SALGKQLNLKL	1183.7289	4.089	0	9	0	0	0	n/a	2	High	0	
ND_WT		36	46	SALGKQLNLKL	1183.7289	4.0742	30	9	0.058	2.404	8.904	1.01	2	High	0.112	0.0137882
ND_WT		36	46	SALGKQLNLKL	1183.7289	4.0641	300	9	0.557	4.126	15.281	1.046	2	High	0.116	0.0425068
ND_WT		36	46	SALGKQLNLKL	1183.7289	4.0788	3000	9	4.073	5.614	20.792	0.013	2	Medium	0.001	0.647514
ND_WT		36	46	SALGKQLNLKL	1183.7289	4.0694	9000	9	7.213	5.721	21.188	0.828	2	Medium	0.092	0.990285
ND_WT		38	44	LGKQLNL	784.4807	3.4489	0	5	0	0	0	n/a	2	Medium	0	
ND_WT		38	44	LGKQLNL	784.4807	3.4213	30	5	0.038	1.19	11.901	0.195	2	Medium	0.022	0.00320854
ND_WT		38	44	LGKQLNL	784.4807	3.4311	300	5	0.367	2.087	20.868	0.174	2	Medium	0.019	0.030798
ND_WT		38	44	LGKQLNL	784.4807	3.4314	3000	5	2.565	2.923	29.23	0.013	2	Medium	0.001	0.116438
ND_WT		38	44	LGKQLNL	784.4807	3.416	9000	5	4.246	2.975	29.751	0.278	2	Medium	0.031	0.29965
ND_WT		38	46	LGKQLNLKL	1025.6597	3.7951	0	7	0	0	0	n/a	2	High	0	
ND_WT		38	46	LGKQLNLKL	1025.6597	3.7649	30	7	0.046	1.858	13.273	0.828	2	High	0.092	0.0130541
ND_WT		38	46	LGKQLNLKL	1025.6597	3.7747	300	7	0.444	3.143	22.449	0.153	2	High	0.017	0.130054
ND_WT		38	46	LGKQLNLKL	1025.6597	3.7756	3000	7	3.208	4.2	29.999	0.148	2	High	0.016	0.0560394
ND_WT		38	46	LGKQLNLKL	1025.6597	3.7842	9000	7	5.608	4.237	30.264	0.684	2	High	0.076	0.722273
ND_WT		39	44	GKQLNL	671.3966	3.0655	0	4	0	0	0	n/a	2	Medium	0	
ND_WT		39	44	GKQLNL	671.3966	3.0488	30	4	0.027	0.95	23.739	0.372	2	Medium	0.041	0.0053026
ND_WT		39	44	GKQLNL	671.3966	3.039	300	4	0.263	1.683	42.082	0.224	2	Medium	0.025	0.039814
ND_WT		39	44	GKQLNL	671.3966	3.0429	3000	4	1.898	2.46	61.504	0.186	2	High	0.021	0.416369
ND_WT		39	44	GKQLNL	671.3966	3.0509	9000	4	3.283	2.501	62.527	0.093	2	Medium	0.01	0.44907
ND_WT		39	46	GKQLNLKL	912.5757	3.5565	0	6	0	0	0	n/a	2	High	0	
ND_WT		39	46	GKQLNLKL	912.5757	3.5561	30	6	0.035	1.527	25.452	0.662	2	High	0.074	0.00926108
ND_WT		39	46	GKQLNLKL	912.5757	3.5656	300	6	0.34	2.633	43.883	0.002	2	High	0	0.057154
ND_WT		39	46	GKQLNLKL	912.5757	3.5584	3000	6	2.541	3.65	60.841	0.205	2	High	0.023	0.932783
ND_WT		39	46	GKQLNLKL	912.5757	3.5733	9000	6	4.644	3.733	62.218	0.492	2	High	0.055	0.715153
ND_WT		45	50	KLLDNW	787.4228	4.5204	0	4	0	0	0	n/a	2	High	0	
ND_WT		45	50	KLLDNW	787.4228	4.4962	30	4	0.08	1.132	28.303	0.544	2	Medium	0.061	0.0174353
ND_WT		45	50	KLLDNW	787.4228	4.5042	300	4	0.674	1.725	43.115	0.058	2	Medium	0.007	0.00147044
ND_WT		45	50	KLLDNW	787.4228	4.5183	3000	4	2.557	2.19	54.753	0.298	2	Medium	0.033	0.858251
ND_WT		45	50	KLLDNW	787.4228	4.5247	9000	4	3.449	2.191	54.771	0.032	2	Medium	0.004	0.795436
ND_WT		45	51	KLLDNWD	902.4498	4.2122	0	5	0	0	0	n/a	2	Medium	0	
ND_WT		45	51	KLLDNWD	902.4498	4.2323	30	5	0.098	1.482	14.817	0.4	2	Medium	0.045	0.00242492
ND_WT		45	51	KLLDNWD	902.4498	4.1887	300	5	0.84	2.031	20.307	0.304	2	Medium	0.034	0.012832
ND_WT		45	51	KLLDNWD	902.4498	4.1845	3000	5	3.395	2.379	23.791	0.634	2	Medium	0.071	0.156147
ND_WT		45	51	KLLDNWD	902.4498	4.1943	9000	5	4.445	2.331	23.309	0.249	2	Medium	0.028	0.0270832
ND_WT		45	56	KLLDNWDSVTST	1377.6776	4.249	0	10	0	0	0	n/a	2	High	0	
ND_WT		45	56	KLLDNWDSVTST	1377.6776	4.2206	30	10	0.173	3.455	17.276	0.918	2	Medium	0.102	0.0136794
ND_WT		45	56	KLLDNWDSVTST	1377.6776	4.2464	300	10	1.499	4.593	22.967	0.571	2	Medium	0.064	0.0149987
ND_WT		45	56	KLLDNWDSVTST	1377.6776	4.2305	3000	10	6.315	5.597	27.987	0.958	2	Medium	0.107	0.880856
ND_WT		45	56	KLLDNWDSVTST	1377.6776	4.2572	9000	10	8.564	5.449	27.243	0.251	2	Medium	0.028	0.354308
ND_WT		47	56	LDNWDSVTST	1136.4986	3.9698	0	8	0	0	0	n/a	2	High	0	
ND_WT		47	56	LDNWDSVTST	1136.4986	3.9817	30	8	0.154	3.187	39.835	0.233	2	High	0.026	0.00618778
ND_WT		47	56	LDNWDSVTST	1136.4986	3.9418	300	8	1.317	4.163	52.041	0.257	2	High	0.029	0.00650635
ND_WT		47	56	LDNWDSVTST	1136.4986	3.9451	3000	8	5.25	4.661	58.269	0.362	2	High	0.04	0.556408
ND_WT		47	56	LDNWDSVTST	1136.4986	3.9498	9000	8	6.987	4.635	57.933	0.179	2	High	0.02	0.987437
ND_WT		50	56	WDSVTST	794.3446	3.0608	0	5	0	0	0	n/a	2	Medium	0	
ND_WT		50	56	WDSVTST	794.3446	3.0419	30	5	0.075	2.218	44.358	0.01	2	Medium	0.001	0.0194266
ND_WT		50	56	WDSVTST	794.3446	3.0618	300	5	0.659	2.652	53.049	0.083	2	Medium	0.009	0.0456155
ND_WT		50	56	WDSVTST	794.3446	3.0661	3000	5	2.92	2.942	58.836	0.154	2	Medium	0.017	0.107459
ND_WT		50	56	WDSVTST	794.3446	3.0753	9000	5	4.119	2.901	58.015	0.171	2	Medium	0.019	0.852413
ND_WT		51	56	DSVTST	608.2653	1.4986	0	4	0	0	0	n/a	2	High	0	
ND_WT		51	56	DSVTST	608.2653	1.4818	30	4	0.032	1.573	39.319	0.601	2	High	0.067	0.01703
ND_WT		51	56	DSVTST	608.2653	1.513	300	4	0.305	2.119	52.981	0.327	2	High	0.036	0.0164702
ND_WT		51	56	DSVTST	608.2653	1.4982	3000	4	1.933	2.432	60.806	0.01	2	High	0.001	0.0469579
ND_WT		51	56	DSVTST	608.2653	1.4952	9000	4	3.119	2.435	60.869	0.2	2	High	0.022	0.875205
ND_WT		51	57	DSVTSTF	755.3337	3.6161	0	5	0	0	0	n/a	2	High	0	
ND_WT		51	57	DSVTSTF	755.3337	3.5997	30	5	0.037	1.835	36.698	0.258	2	High	0.029	0.0046691
ND_WT		51	57	DSVTSTF	755.3337	3.585	300	5	0.356	2.462	49.244	0.196	2	High	0.022	0.00376435
ND_WT		51	57	DSVTSTF	755.3337	3.5792	3000	5	2.34	2.998	59.966	0.142	2	High	0.016	0.043902

**ND_WT**		51	57	DSVTSTF	755.3337	3.5716	9000	5	3.91	3.07	61.394	0.376		2	High	0.042	0.919792
**ND_WT**		57	70	FSKLREQLGPVTQE	1630.8679	3.1941	0	11	0	0	0	n/a		2	High	0	
**ND_WT**		57	70	FSKLREQLGPVTQE	1630.8679	3.1849	30	11	0.085	1.835	8.341	1.062		2	High	0.118	0.0438199
**ND_WT**		57	70	FSKLREQLGPVTQE	1630.8679	3.1865	300	11	0.814	3.676	16.708	0.403		2	Medium	0.045	0.477303
**ND_WT**		57	70	FSKLREQLGPVTQE	1630.8679	3.1838	3000	11	5.447	5.652	25.689	0.987		2	Medium	0.11	0.096542
**ND_WT**		57	70	FSKLREQLGPVTQE	1630.8679	3.1955	9000	11	8.858	5.966	27.119	0.044		2	Medium	0.005	0.301101
**ND_WT**		57	71	FSKLREQLGPVTQEF	1777.9363	4.1438	0	12	0	0	0	n/a		2	High	0	
**ND_WT**		57	71	FSKLREQLGPVTQEF	1777.9363	4.1109	30	12	0.093	1.854	7.724	1.304		2	High	0.145	0.0194828
**ND_WT**		57	71	FSKLREQLGPVTQEF	1777.9363	4.0963	300	12	0.892	3.821	15.919	0.267		2	High	0.03	0.331397
**ND_WT**		57	71	FSKLREQLGPVTQEF	1777.9363	4.1207	3000	12	6.004	6.071	25.296	0.604		2	High	0.067	0.0516166
**ND_WT**		57	71	FSKLREQLGPVTQEF	1777.9363	4.1201	9000	12	9.771	6.581	27.422	0.4		2	High	0.045	0.116122
**ND_WT**		58	70	SKLREQLGPVTQE	1483.7995	2.7834	0	10	0	0	0	n/a		2	Medium	0	
**ND_WT**		58	70	SKLREQLGPVTQE	1483.7995	2.7668	30	10	0.076	1.629	8.147	1.225		2	High	0.136	0.0352343
**ND_WT**		58	70	SKLREQLGPVTQE	1483.7995	2.7728	300	10	0.725	3.326	16.631	0.316		2	High	0.035	0.416155
**ND_WT**		58	70	SKLREQLGPVTQE	1483.7995	2.7556	3000	10	4.841	5.344	26.721	0.993		2	Medium	0.11	0.120703
**ND_WT**		58	70	SKLREQLGPVTQE	1483.7995	2.7599	9000	10	7.919	5.529	27.645	0.563		2	Medium	0.063	0.862087
**ND_WT**		58	71	SKLREQLGPVTQEF	1630.8679	3.8341	0	11	0	0	0	n/a		2	Medium	0	
**ND_WT**		58	71	SKLREQLGPVTQEF	1630.8679	3.8227	30	11	0.084	1.788	6.716	0.316		4	Medium	0.199	0.0013948
**ND_WT**		58	71	SKLREQLGPVTQEF	1630.8679	3.8295	300	11	0.803	3.659	13.867	0.13		4	Medium	0.082	0.584414
**ND_WT**		58	71	SKLREQLGPVTQEF	1630.8679	3.7858	3000	11	5.397	5.948	22.624	0.295		4	Medium	0.185	0.00371107
**ND_WT**		58	71	SKLREQLGPVTQEF	1630.8679	3.809	9000	11	8.832	6.378	24.206	0.172		4	Medium	0.108	0.00683474
**ND_WT**		61	71	REQLGPVTQEF	1302.6568	4.02	0	8	0	0	0	n/a		2	High	0	
**ND_WT**		61	71	REQLGPVTQEF	1302.6568	3.9581	30	8	0.062	1.454	9.09	0.54		2	High	0.06	0.00370614
**ND_WT**		61	71	REQLGPVTQEF	1302.6568	3.957	300	8	0.589	2.673	16.704	0.06		2	High	0.007	0.0463194
**ND_WT**		61	71	REQLGPVTQEF	1302.6568	3.973	3000	8	3.963	4.031	25.193	0.604		2	High	0.067	0.0896742
**ND_WT**		61	71	REQLGPVTQEF	1302.6568	3.9747	9000	8	6.444	4.273	26.706	0.328		2	High	0.037	0.0884735
**ND_WT**		72	91	WDNLEKETEGLRQEMSKDLE	2449.1431	5.1429	0	18	0	0	0	n/a		2	High	0	
**ND_WT**		72	91	WDNLEKETEGLRQEMSKDLE	2449.1431	5.1444	30	18	0.241	1.582	2.198	3.198		2	Medium	0.356	0.250696
**ND_WT**		72	91	WDNLEKETEGLRQEMSKDLE	2449.1431	5.146	300	18	2.187	5.602	7.781	0.025		2	Medium	0.003	2.70E-05
**ND_WT**		72	91	WDNLEKETEGLRQEMSKDLE	2449.1431	5.0906	3000	18	11.683	8.89	12.347	0.757		2	Medium	0.084	1.12E-05
**ND_WT**		72	91	WDNLEKETEGLRQEMSKDLE	2449.1431	5.0644	9000	18	16.731	10.762	14.947	0.235		2	Medium	0.026	3.11E-05
**ND_WT**		72	92	WDNLEKETEGLRQEMSKDLEE	2578.1857	5.1956	0	19	0	0	0	n/a		2	High	0	
**ND_WT**		72	92	WDNLEKETEGLRQEMSKDLEE	2578.1857	5.1634	30	19	0.261	2.11	3.702	0.698		2	High	0.078	0.261786
**ND_WT**		72	92	WDNLEKETEGLRQEMSKDLEE	2578.1857	5.1354	300	19	2.369	5.279	9.262	0.543		2	High	0.06	5.73E-05
**ND_WT**		72	92	WDNLEKETEGLRQEMSKDLEE	2578.1857	5.1328	3000	19	12.549	8.921	15.651	0.054		2	High	0.006	0.000250472
**ND_WT**		72	92	WDNLEKETEGLRQEMSKDLEE	2578.1857	5.1198	9000	19	17.729	10.123	17.76	0.301		2	High	0.034	2.86E-05
**ND_WT**		72	103	WDNLEKETEGLRQEMSKDLEEVKAKVQPYL[illegible]	3834.8622	5.8617	0	29	0	0	0	n/a		2	Medium	0	
**ND_WT**		72	103	WDNLEKETEGLRQEMSKDLEEVKAKVQPYL[illegible]	3834.8622	5.8943	30	29	0.4	1.714	1.478	0.863		2	High	0.096	0.0969785
**ND_WT**		72	103	WDNLEKETEGLRQEMSKDLEEVKAKVQPYL[illegible]	3834.8622	5.9098	300	29	3.492	3.317	2.86	0.426		2	High	0.047	0.0182037
**ND_WT**		72	103	WDNLEKETEGLRQEMSKDLEEVKAKVQPYL[illegible]	3834.8622	5.8766	3000	29	17.379	5.269	4.542	0.294		2	High	0.033	0.0342987
**ND_WT**		72	103	WDNLEKETEGLRQEMSKDLEEVKAKVQPYL[illegible]	3834.8622	5.914	9000	29	25.476	6.323	5.451	1.374		2	High	0.153	0.0364786
**ND_WT**		77	92	KETEGLRQEMSKDLEE	1920.9099	3.5224	0	14	0	0	0	n/a		2	Medium	0	
**ND_WT**		77	92	KETEGLRQEMSKDLEE	1920.9099	3.4364	30	14	0.173	2.426	8.665	0.52		2	High	0.058	0.110722
**ND_WT**		77	92	KETEGLRQEMSKDLEE	1920.9099	3.4521	300	14	1.615	3.968	14.17	0.09		2	High	0.01	0.0192636
**ND_WT**		77	92	KETEGLRQEMSKDLEE	1920.9099	3.4183	3000	14	9.321	6.706	23.949	1.009		2	High	0.112	0.0149295
**ND_WT**		77	92	KETEGLRQEMSKDLEE	1920.9099	3.4161	9000	14	13.171	7.437	26.561	0.524		2	High	0.058	0.001934
**ND_WT**		83	92	RQEMSKDLEE	1263.5765	2.2989	0	8	0	0	0	n/a		2	Medium	0	
**ND_WT**		83	92	RQEMSKDLEE	1263.5765	2.2699	30	8	0.114	1.166	7.285	0.148		2	Medium	0.016	0.00361857
**ND_WT**		83	92	RQEMSKDLEE	1263.5765	2.2853	300	8	1.052	1.993	12.459	0.324		2	Medium	0.036	0.00693483
**ND_WT**		83	92	RQEMSKDLEE	1263.5765	2.28	3000	8	5.659	3.374	21.086	0.11		2	Medium	0.012	4.90E-05
**ND_WT**		83	92	RQEMSKDLEE	1263.5765	2.2876	9000	8	7.634	3.91	24.439	0.465		2	Medium	0.052	0.00268484
**ND_WT**		87	92	SKDLEE	719.3337	1.6986	0	4	0	0	0	n/a		2	Medium	0	
**ND_WT**		87	92	SKDLEE	719.3337	1.6995	30	4	0.064	0.254	6.361	0.068		2	Medium	0.008	0.0072198
**ND_WT**		87	92	SKDLEE	719.3337	1.6941	300	4	0.579	0.66	16.498	0.035		2	Medium	0.004	0.000294053
**ND_WT**		87	92	SKDLEE	719.3337	1.7138	3000	4	2.821	1.385	34.632	0.308		2	Medium	0.034	0.00172663
**ND_WT**		87	92	SKDLEE	719.3337	1.7021	9000	4	3.749	1.559	38.975	0.602		2	Medium	0.067	0.0256131
**ND_WT**		90	103	LEEVKAKVQPYLDD	1645.8563	3.2673	0	11	0	0	0	n/a		2	High	0	
**ND_WT**		90	103	LEEVKAKVQPYLDD	1645.8563	3.239	30	11	0.159	0.868	3.945	0.456		2	High	0.051	0.0566528
**ND_WT**		90	103	LEEVKAKVQPYLDD	1645.8563	3.2473	300	11	1.305	1.469	6.677	0.763		2	High	0.085	0.0336713
**ND_WT**		90	103	LEEVKAKVQPYLDD	1645.8563	3.2434	3000	11	5.696	3.375	15.339	0.177		2	High	0.02	0.000490842
**ND_WT**		90	103	LEEVKAKVQPYLDD	1645.8563	3.2399	9000	11	8.744	4.382	19.917	0.708		2	High	0.079	0.00309691
**ND_WT**		91	103	EEVKAKVQPYLDD	1532.7722	2.9638	0	10	0	0	0	n/a		2	High	0	
**ND_WT**		91	103	EEVKAKVQPYLDD	1532.7722	2.9816	30	10	0.139	0.657	3.283	0.83		2	High	0.092	0.392253
**ND_WT**		91	103	EEVKAKVQPYLDD	1532.7722	2.9601	300	10	1.123	1.265	6.326	0.142		2	High	0.016	0.0366743
**ND_WT**		91	103	EEVKAKVQPYLDD	1532.7722	2.9487	3000	10	4.829	2.864	14.318	0.185		2	High	0.021	0.0292372
**ND_WT**		91	103	EEVKAKVQPYLDD	1532.7722	2.9563	9000	10	7.747	3.547	17.735	0.72		2	High	0.08	0.00492098
**ND_WT**		92	103	EVKAKVQPYLDD	1403.7296	2.8667	0	9	0	0	0	n/a		2	High	0	
**ND_WT**		92	103	EVKAKVQPYLDD	1403.7296	2.8389	30	9	0.136	0.569	3.161	0.868		2	High	0.097	0.399338
**ND_WT**		92	103	EVKAKVQPYLDD	1403.7296	2.8644	300	9	1.091	1.344	7.469	0.215		2	High	0.024	0.0560442
**ND_WT**		92	103	EVKAKVQPYLDD	1403.7296	2.881	3000	9	4.55	2.876	15.975	0.742		2	High	0.083	0.029983
**ND_WT**		92	103	EVKAKVQPYLDD	1403.7296	2.879	9000	9	7.122	3.57	19.831	0.507		2	High	0.056	0.00240023
**ND_WT**		93	103	VKAKVQPYLDD	1274.6871	2.7101	0	8	0	0	0	n/a		2	High	0	
**ND_WT**		93	103	VKAKVQPYLDD	1274.6871	2.7037	30	8	0.131	0.53	3.312	0.547		2	High	0.061	0.102263
**ND_WT**		93	103	VKAKVQPYLDD	1274.6871	2.7038	300	8	1.047	1.251	7.82	0.088		2	High	0.01	0.0133984
**ND_WT**		93	103	VKAKVQPYLDD	1274.6871	2.6974	3000	8	4.191	2.614	16.34	0.49		2	High	0.055	0.0234215
**ND_WT**		93	103	VKAKVQPYLDD	1274.6871	2.6998	9000	8	6.384	3.188	19.925	0.267		2	High	0.03	0.00615283
**ND_WT**		93	104	VKAKVQPYLDDF	1421.7555	4.2585	0	9	0	0	0	n/a		2	Medium	0	
**ND_WT**		93	104	VKAKVQPYLDDF	1421.7555	4.3555	30	9	0.144	0.336	1.869	0.392		2	High	0.044	0.150447

**ND_WT**		93	104	VKAKVQPYLDDF	1421.7555	4.3351	300	9	1.168	1.058	5.879	0.229	2	High	0.025	0.0274731
**ND_WT**		93	104	VKAKVQPYLDDF	1421.7555	4.3232	3000	9	4.915	2.579	14.329	0.344	2	High	0.038	0.0109728
**ND_WT**		93	104	VKAKVQPYLDDF	1421.7555	4.3308	9000	9	7.363	3.209	17.828	0.242	2	High	0.027	0.0108543
**ND_WT**		96	103	KVQPYLDD	976.4866	2.9039	0	5	0	0	0	n/a	2	Medium	0	
**ND_WT**		96	103	KVQPYLDD	976.4866	2.931	30	5	0.111	0.285	2.85	0.327	2	Medium	0.036	0.0379913
**ND_WT**		96	103	KVQPYLDD	976.4866	2.9233	300	5	0.848	0.683	6.825	0.011	2	Medium	0.001	0.0553945
**ND_WT**		96	103	KVQPYLDD	976.4866	2.9347	3000	5	2.803	1.259	12.585	0.328	2	Medium	0.036	0.0130004
**ND_WT**		96	103	KVQPYLDD	976.4866	2.9377	9000	5	4.053	1.451	14.509	0.09	2	Medium	0.01	0.0271345
**ND_WT**		104	111	FQKKWQEE	1121.5506	2.54	0	6	0	0	0	n/a	2	High	0	
**ND_WT**		104	111	FQKKWQEE	1121.5506	2.5411	30	6	0.058	0.294	4.905	0.241	2	High	0.027	0.501394
**ND_WT**		104	111	FQKKWQEE	1121.5506	2.5359	300	6	0.55	0.839	13.978	0.401	2	High	0.045	0.015744
**ND_WT**		104	111	FQKKWQEE	1121.5506	2.5297	3000	6	3.439	2.057	34.287	0.169	2	High	0.019	0.0068504
**ND_WT**		104	111	FQKKWQEE	1121.5506	2.5379	9000	6	5.306	2.48	41.332	0.206	2	High	0.023	0.0042668
**ND_WT**		113	124	ELYRQKVEPLRA	1500.8413	3.1804	0	9	0	0	0	n/a	2	High	0	
**ND_WT**		113	124	ELYRQKVEPLRA	1500.8413	3.1798	30	9	0.06	2.006	7.431	0.766	2	High	0.085	0.0111003
**ND_WT**		113	124	ELYRQKVEPLRA	1500.8413	3.1015	300	9	0.574	3.108	11.511	0.188	2	High	0.021	0.00486241
**ND_WT**		113	124	ELYRQKVEPLRA	1500.8413	3.092	3000	9	4.134	4.533	16.787	0.246	2	High	0.027	0.00519592
**ND_WT**		113	124	ELYRQKVEPLRA	1500.8413	3.0918	9000	9	7.172	4.918	18.216	0.215	2	High	0.024	0.0631542
**ND_WT**		113	126	ELYRQKVEPLRAEL	1742.9679	3.5243	0	11	0	0	0	n/a	2	High	0	
**ND_WT**		113	126	ELYRQKVEPLRAEL	1742.9679	3.5256	30	11	0.074	2.157	6.535	0.583	2	High	0.065	0.000547247
**ND_WT**		113	126	ELYRQKVEPLRAEL	1742.9679	3.5221	300	11	0.718	3.42	10.364	0.156	2	High	0.017	0.0218044
**ND_WT**		113	126	ELYRQKVEPLRAEL	1742.9679	3.5119	3000	11	5.145	5.246	15.896	0.451	2	Medium	0.05	0.0181132
**ND_WT**		113	126	ELYRQKVEPLRAEL	1742.9679	3.5205	9000	11	8.871	5.857	17.748	0.186	2	Medium	0.021	0.0103121
**ND_WT**		114	124	LYRQKVEPLRA	1371.7987	2.4079	0	8	0	0	0	n/a	2	High	0	
**ND_WT**		114	124	LYRQKVEPLRA	1371.7987	2.405	30	8	0.055	2.208	9.2	0.271	2	High	0.03	0.000289572
**ND_WT**		114	124	LYRQKVEPLRA	1371.7987	2.4239	300	8	0.529	2.886	12.025	0.166	2	High	0.019	0.00951987
**ND_WT**		114	124	LYRQKVEPLRA	1371.7987	2.4257	3000	8	3.763	4.108	17.116	0.176	2	High	0.02	0.00108524
**ND_WT**		114	124	LYRQKVEPLRA	1371.7987	2.4338	9000	8	6.42	4.511	18.796	0.338	2	High	0.038	0.0844203
**ND_WT**		114	125	LYRQKVEPLRAE	1500.8413	2.9962	0	9	0	0	0	n/a	2	High	0	
**ND_WT**		114	125	LYRQKVEPLRAE	1500.8413	2.9816	30	9	0.065	2.058	7.621	1.22	2	High	0.136	0.018867
**ND_WT**		114	125	LYRQKVEPLRAE	1500.8413	2.9839	300	9	0.628	3.121	11.559	0.26	2	High	0.029	0.00228284
**ND_WT**		114	125	LYRQKVEPLRAE	1500.8413	2.9786	3000	9	4.41	4.549	16.85	0.516	2	High	0.057	0.0254327
**ND_WT**		114	125	LYRQKVEPLRAE	1500.8413	2.9822	9000	9	7.376	4.938	18.287	0.141	2	High	0.016	0.0664529
**ND_WT**		114	126	LYRQKVEPLRAEL	1613.9253	3.3399	0	10	0	0	0	n/a	2	High	0	
**ND_WT**		114	126	LYRQKVEPLRAEL	1613.9253	3.3494	30	10	0.07	2.29	9.508	0.167	4	High	0.105	1.05E-07
**ND_WT**		114	126	LYRQKVEPLRAEL	1613.9253	3.3269	300	10	0.672	3.457	14.369	0.078	4	High	0.049	5.67E-09
**ND_WT**		114	126	LYRQKVEPLRAEL	1613.9253	3.3287	3000	10	4.773	5.116	21.289	0.113	4	High	0.071	6.59E-05
**ND_WT**		114	126	LYRQKVEPLRAEL	1613.9253	3.3341	9000	10	8.119	5.64	23.489	0.026	4	High	0.016	0.0247625
**ND_WT**		115	124	YRQKVEPLRA	1258.7146	2.1221	0	7	0	0	0	n/a	2	High	0	
**ND_WT**		115	124	YRQKVEPLRA	1258.7146	2.0579	30	7	0.048	2.032	9.675	0.493	2	High	0.055	0.0132555
**ND_WT**		115	124	YRQKVEPLRA	1258.7146	2.1049	300	7	0.46	2.648	12.609	0.085	2	High	0.009	0.00886208
**ND_WT**		115	124	YRQKVEPLRA	1258.7146	2.0956	3000	7	3.251	3.654	17.402	0.35	2	High	0.039	0.0276985
**ND_WT**		115	124	YRQKVEPLRA	1258.7146	2.1037	9000	7	5.537	3.924	18.686	0.097	2	High	0.011	0.0768171
**ND_WT**		115	126	YRQKVEPLRAEL	1500.8413	3.1618	0	9	0	0	0	n/a	2	High	0	
**ND_WT**		115	126	YRQKVEPLRAEL	1500.8413	3.1504	30	9	0.063	2.03	6.588	0.226	4	High	0.142	6.19E-06
**ND_WT**		115	126	YRQKVEPLRAEL	1500.8413	3.1009	300	9	0.603	3.069	9.965	0.08	4	High	0.051	7.78E-09
**ND_WT**		115	126	YRQKVEPLRAEL	1500.8413	3.1043	3000	9	4.262	4.478	14.539	0.106	4	High	0.067	2.90E-06
**ND_WT**		115	126	YRQKVEPLRAEL	1500.8413	3.1115	9000	9	7.235	4.892	15.865	0.07	4	High	0.044	0.000555289
**ND_WT**		115	147	YRQKVEPLRAELQEGARQKLHELQEKLSPLG	3901.081	4.5572	0	29	0	0	0	n/a	2	Medium	0	
**ND_WT**		115	147	YRQKVEPLRAELQEGARQKLHELQEKLSPLG	3901.081	4.5592	30	29	0.313	5.078	3.502	0.692	2	High	0.077	0.00563308
**ND_WT**		115	147	YRQKVEPLRAELQEGARQKLHELQEKLSPLG	3901.081	4.5105	300	29	2.883	9.657	6.66	0.347	2	High	0.039	0.00995529
**ND_WT**		115	147	YRQKVEPLRAELQEGARQKLHELQEKLSPLG	3901.081	4.5101	3000	29	16.5	13.69	9.441	0.947	2	High	0.105	0.00161758
**ND_WT**		115	147	YRQKVEPLRAELQEGARQKLHELQEKLSPLG	3901.081	4.5222	9000	29	24.896	15.286	10.542	2.402	2	High	0.267	0.0524239
**ND_WT**		115	158	YRQKVEPLRAELQEGARQKLHELQEKLSPLG	5179.7174	4.5203	0	40	0	0	0	n/a	2	Medium	0	
**ND_WT**		115	158	YRQKVEPLRAELQEGARQKLHELQEKLSPLG	5179.7174	4.5493	30	40	0.506	6.034	2.155	1.647	2	High	0.183	0.00663554
**ND_WT**		115	158	YRQKVEPLRAELQEGARQKLHELQEKLSPLG	5179.7174	4.4988	300	40	4.628	11.711	4.183	0.076	2	High	0.008	0.00713316
**ND_WT**		115	158	YRQKVEPLRAELQEGARQKLHELQEKLSPLG	5179.7174	4.4883	3000	40	24.825	17.781	6.35	0.949	2	High	0.106	0.0249609
**ND_WT**		115	158	YRQKVEPLRAELQEGARQKLHELQEKLSPLG	5179.7174	4.4975	9000	40	35.51	19.519	6.971	0.648	2	High	0.072	0.0294723
**ND_WT**		125	136	ELQEGARQKLHE	1436.7372	1.9467	0	10	0	0	0	n/a	2	Medium	0	
**ND_WT**		125	136	ELQEGARQKLHE	1436.7372	1.9451	30	10	0.157	2.593	12.963	0.325	2	High	0.036	0.00356174
**ND_WT**		125	136	ELQEGARQKLHE	1436.7372	1.9208	300	10	1.395	3.837	19.185	0.497	2	Medium	0.055	0.00180241
**ND_WT**		125	136	ELQEGARQKLHE	1436.7372	1.921	3000	10	6.71	4.787	23.937	0.967	2	Medium	0.108	0.0532615
**ND_WT**		125	136	ELQEGARQKLHE	1436.7372	1.9204	9000	10	9.173	4.854	24.269	0.869	2	Medium	0.097	0.025101
**ND_WT**		125	139	ELQEGARQKLHELQE	1806.9224	2.9776	0	13	0	0	0	n/a	2	High	0	
**ND_WT**		125	139	ELQEGARQKLHELQE	1806.9224	2.9925	30	13	0.181	2.559	9.842	0.371	2	High	0.041	0.00191191
**ND_WT**		125	139	ELQEGARQKLHELQE	1806.9224	2.9819	300	13	1.624	3.815	14.674	0.59	2	High	0.066	0.000922077
**ND_WT**		125	139	ELQEGARQKLHELQE	1806.9224	2.9539	3000	13	8.264	5.892	22.662	0.126	2	High	0.014	0.00510854
**ND_WT**		125	139	ELQEGARQKLHELQE	1806.9224	2.9505	9000	13	11.729	6.263	24.087	0.243	2	High	0.027	0.00443521
**ND_WT**		125	146	ELQEGARQKLHELQEKLSPLGE	2531.3344	4.3593	0	19	0	0	0	n/a	2	High	0	
**ND_WT**		125	146	ELQEGARQKLHELQEKLSPLGE	2531.3344	4.3298	30	19	0.23	4.496	7.888	0.396	2	Medium	0.044	0.000116965

**ND_WT**		125	146	ELQEGARQKLHELQEKLSPLGE	2531.3344	4.3056	300	19	2.098	6.795	11.921	0.709	2	High	0.079	1.56E-05
**ND_WT**		125	146	ELQEGARQKLHELQEKLSPLGE	2531.3344	4.2832	3000	19	11.371	9.345	16.394	0.67	2	High	0.075	0.000281623
**ND_WT**		125	146	ELQEGARQKLHELQEKLSPLGE	2531.3344	4.2701	9000	19	16.663	9.561	16.773	0.721	2	High	0.08	0.004279
**ND_WT**		125	148	ELQEGARQKLHELQEKLSPLGEEM	2791.4174	4.5711	0	21	0	0	0	n/a	2	High	0	
**ND_WT**		125	148	ELQEGARQKLHELQEKLSPLGEEM	2791.4174	4.5645	30	21	0.262	4.87	6.724	0.595	4	High	0.374	3.20E-07
**ND_WT**		125	148	ELQEGARQKLHELQEKLSPLGEEM	2791.4174	4.5526	300	21	2.393	7.431	10.278	0.401	4	High	0.252	3.75E-06
**ND_WT**		125	148	ELQEGARQKLHELQEKLSPLGEEM	2791.4174	4.5332	3000	21	12.937	10.297	14.285	0.179	4	High	0.113	4.04E-06
**ND_WT**		125	148	ELQEGARQKLHELQEKLSPLGEEM	2791.4174	4.5326	9000	21	18.634	10.939	15.206	0.15	4	High	0.094	0.0011102
**ND_WT**		125	158	ELQEGARQKLHELQEKLSPLGEEMRDRARAI	3939.0133	4.2996	0	31	0	0	0	n/a	2	High	0	
**ND_WT**		125	158	ELQEGARQKLHELQEKLSPLGEEMRDRARAI	3939.0133	4.3209	30	31	0.443	5.49	3.984	0.056	4	High	0.035	1.63E-05
**ND_WT**		125	158	ELQEGARQKLHELQEKLSPLGEEMRDRARAI	3939.0133	4.2985	300	31	4.025	9.188	6.663	0.323	4	High	0.203	3.93E-07
**ND_WT**		125	158	ELQEGARQKLHELQEKLSPLGEEMRDRARAI	3939.0133	4.2815	3000	31	20.563	13.253	9.611	0.285	4	High	0.179	0.00240528
**ND_WT**		125	158	ELQEGARQKLHELQEKLSPLGEEMRDRARAI	3939.0133	4.2858	9000	31	28.274	14.603	10.595	0.095	4	High	0.06	0.962005
**ND_WT**		125	159	ELQEGARQKLHELQEKLSPLGEEMRDRARAI	4052.0974	4.5297	0	32	0	0	0	n/a	2	Medium	0	
**ND_WT**		125	159	ELQEGARQKLHELQEKLSPLGEEMRDRARAI	4052.0974	4.4871	30	32	0.447	6.223	3.889	2.648	2	High	0.295	0.00353608
**ND_WT**		125	159	ELQEGARQKLHELQEKLSPLGEEMRDRARAI	4052.0974	4.4628	300	32	4.066	9.784	6.115	2.262	2	High	0.252	0.00699473
**ND_WT**		125	159	ELQEGARQKLHELQEKLSPLGEEMRDRARAI	4052.0974	4.4535	3000	32	20.902	14.133	8.833	0.699	2	High	0.078	0.357261
**ND_WT**		125	159	ELQEGARQKLHELQEKLSPLGEEMRDRARAI	4052.0974	4.4549	9000	32	28.986	15.831	9.894	0.775	2	High	0.086	0.0356857
**ND_WT**		126	158	LQEGARQKLHELQEKLSPLGEEMRDRARAH	3809.9707	4.364	0	30	0	0	0	n/a	2	High	0	
**ND_WT**		126	158	LQEGARQKLHELQEKLSPLGEEMRDRARAH	3809.9707	4.3464	30	30	0.437	5.449	3.632	0.903	2	High	0.1	0.00609459
**ND_WT**		126	158	LQEGARQKLHELQEKLSPLGEEMRDRARAH	3809.9707	4.3147	300	30	3.968	8.654	5.769	0.861	2	High	0.096	0.00672876
**ND_WT**		126	158	LQEGARQKLHELQEKLSPLGEEMRDRARAH	3809.9707	4.3271	3000	30	20.117	12.419	8.279	0.187	2	High	0.021	0.0356397
**ND_WT**		126	158	LQEGARQKLHELQEKLSPLGEEMRDRARAH	3809.9707	4.3283	9000	30	27.444	13.641	9.094	1.89	2	High	0.21	0.726539
**ND_WT**		127	139	QEGARQKLHELQE	1564.7958	2.4308	0	11	0	0	0	n/a	2	High	0	
**ND_WT**		127	139	QEGARQKLHELQE	1564.7958	2.4205	30	11	0.161	2.577	11.712	0.462	2	High	0.051	0.00134313
**ND_WT**		127	139	QEGARQKLHELQE	1564.7958	2.4021	300	11	1.44	3.842	17.463	0.862	2	High	0.096	0.0117799
**ND_WT**		127	139	QEGARQKLHELQE	1564.7958	2.4054	3000	11	7.073	4.922	22.371	0.827	2	High	0.092	0.0263543
**ND_WT**		127	139	QEGARQKLHELQE	1564.7958	2.4044	9000	11	9.916	5.255	23.886	0.452	2	High	0.05	0.0141444
**ND_WT**		127	146	QEGARQKLHELQEKLSPLGE	2289.2077	4.0888	0	17	0	0	0	n/a	2	High	0	
**ND_WT**		127	146	QEGARQKLHELQEKLSPLGE	2289.2077	4.1085	30	17	0.211	3.316	6.502	0.523	2	High	0.058	0.000403934
**ND_WT**		127	146	QEGARQKLHELQEKLSPLGE	2289.2077	4.0288	300	17	1.913	5.898	11.564	0.089	2	High	0.01	2.44E-05
**ND_WT**		127	146	QEGARQKLHELQEKLSPLGE	2289.2077	4.003	3000	17	10.181	8.529	16.724	0.459	2	High	0.051	0.00553382
**ND_WT**		127	146	QEGARQKLHELQEKLSPLGE	2289.2077	4.0024	9000	17	14.85	8.896	17.442	0.125	2	High	0.014	0.0152855
**ND_WT**		127	147	QEGARQKLHELQEKLSPLGEE	2418.2503	4.0729	0	18	0	0	0	n/a	2	High	0	
**ND_WT**		127	147	QEGARQKLHELQEKLSPLGEE	2418.2503	4.0624	30	18	0.231	3.994	8.186	0.668	4	High	0.42	0.000720754
**ND_WT**		127	147	QEGARQKLHELQEKLSPLGEE	2418.2503	4.0469	300	18	2.095	6.302	13.287	0.481	4	High	0.302	1.76E-05
**ND_WT**		127	147	QEGARQKLHELQEKLSPLGEE	2418.2503	4.0406	3000	18	11.048	8.495	17.857	0.455	4	High	0.286	0.000887483
**ND_WT**		127	147	QEGARQKLHELQEKLSPLGEE	2418.2503	4.0369	9000	18	15.847	8.924	18.709	0.426	4	Medium	0.268	0.0129804
**ND_WT**		127	154	QEGARQKLHELQEKLSPLGEEMRDRARA	3274.6953	4.1256	0	25	0	0	0	n/a	2	Medium	0	
**ND_WT**		127	154	QEGARQKLHELQEKLSPLGEEMRDRARA	3274.6953	4.0864	30	25	0.344	4.143	4.143		1	High	0	
**ND_WT**		127	154	QEGARQKLHELQEKLSPLGEEMRDRARA	3274.6953	4.0536	300	25	3.121	7.912	7.912	2.135	2	Medium	0.238	0.0101678
**ND_WT**		127	154	QEGARQKLHELQEKLSPLGEEMRDRARA	3274.6953	4.0697	3000	25	16.178	11.054	11.054	0.951	2	High	0.106	0.019937
**ND_WT**		127	154	QEGARQKLHELQEKLSPLGEEMRDRARA	3274.6953	4.0657	9000	25	22.604	12.025	12.025	2.392	2	High	0.266	0.0689359
**ND_WT**		127	158	QEGARQKLHELQEKLSPLGEEMRDRARAHV	3696.8867	4.1162	0	29	0	0	0	n/a	2	High	0	
**ND_WT**		127	158	QEGARQKLHELQEKLSPLGEEMRDRARAHV	3696.8867	4.1009	30	29	0.424	4.949	3.413	0.727	2	High	0.081	0.000704449
**ND_WT**		127	158	QEGARQKLHELQEKLSPLGEEMRDRARAHV	3696.8867	4.0752	300	29	3.84	8.357	5.763	0.477	2	High	0.053	0.00465219
**ND_WT**		127	158	QEGARQKLHELQEKLSPLGEEMRDRARAHV	3696.8867	4.0874	3000	29	19.373	11.982	8.263	0.64	2	High	0.071	0.021747
**ND_WT**		127	158	QEGARQKLHELQEKLSPLGEEMRDRARAHV	3696.8867	4.0694	9000	29	26.461	13.229	9.123	0.129	2	High	0.014	0.184773
**ND_WT**		127	159	QEGARQKLHELQEKLSPLGEEMRDRARAHV	3809.9707	4.3594	0	30	0	0	0	n/a	2	Medium	0	
**ND_WT**		127	159	QEGARQKLHELQEKLSPLGEEMRDRARAHV	3809.9707	4.3408	30	30	0.428	5.656	4.251	0.289	4	High	0.181	6.18E-05
**ND_WT**		127	159	QEGARQKLHELQEKLSPLGEEMRDRARAHV	3809.9707	4.33	300	30	3.881	8.582	6.447	0.446	4	High	0.28	6.88E-06
**ND_WT**		127	159	QEGARQKLHELQEKLSPLGEEMRDRARAHV	3809.9707	4.3258	3000	30	19.712	12.592	9.447	0.526	4	High	0.331	0.211325
**ND_WT**		127	159	QEGARQKLHELQEKLSPLGEEMRDRARAHV	3809.9707	4.3296	9000	30	27.172	13.827	10.385	0.386	4	High	0.242	0.110131
**ND_WT**		137	146	LQEKLSPLGE	1112.6077	3.4094	0	7	0	0	0	n/a	2	High	0	
**ND_WT**		137	146	LQEKLSPLGE	1112.6077	3.4129	30	7	0.063	1.603	11.452	0.192	2	High	0.021	0.00433704
**ND_WT**		137	146	LQEKLSPLGE	1112.6077	3.4048	300	7	0.601	2.522	18.012	0.27	2	High	0.03	0.00340758
**ND_WT**		137	146	LQEKLSPLGE	1112.6077	3.4013	3000	7	3.852	3.394	24.246	0.538	2	High	0.06	0.104665
**ND_WT**		137	146	LQEKLSPLGE	1112.6077	3.4209	9000	7	5.917	3.465	24.753	0.177	2	High	0.02	0.071189
**ND_WT**		137	147	LQEKLSPLGEE	1241.6503	3.4465	0	8	0	0	0	n/a	2	High	0	
**ND_WT**		137	147	LQEKLSPLGEE	1241.6503	3.4318	30	8	0.083	1.791	11.195	0.291	2	High	0.032	0.00560774
**ND_WT**		137	147	LQEKLSPLGEE	1241.6503	3.4397	300	8	0.783	2.77	17.315	0.146	2	High	0.016	0.000340093
**ND_WT**		137	147	LQEKLSPLGEE	1241.6503	3.4415	3000	8	4.718	3.782	23.64	0.307	2	High	0.034	0.0573727
**ND_WT**		137	147	LQEKLSPLGEE	1241.6503	3.4456	9000	8	6.915	3.858	24.11	0.019	2	High	0.002	0.167962
**ND_WT**		137	158	LQEKLSPLGEEMRDRARAHVDA	2520.2867	3.3317	0	19	0	0	0	n/a	2	High	0	
**ND_WT**		137	158	LQEKLSPLGEEMRDRARAHVDA	2520.2867	3.3253	30	19	0.276	2.938	4.119	0.47	4	High	0.295	0.000183479
**ND_WT**		137	158	LQEKLSPLGEEMRDRARAHVDA	2520.2867	3.3271	300	19	2.528	4.872	6.836	0.226	4	High	0.142	0.000290628
**ND_WT**		137	158	LQEKLSPLGEEMRDRARAHVDA	2520.2867	3.3129	3000	19	13.043	7.605	10.673	0.14	4	High	0.088	0.00194717

**ND_WT**		137	158	LQEKLSPLGEEMRDRARAHVDA	2520.2867	3.3237	9000	19	17.528	8.346	11.715	0.036	4	High	0.023	0.000310794
**ND_WT**		137	159	LQEKLSPLGEEMRDRARAHVDAL	2633.3708	3.864	0	20	0	0	0	n/a	2	High	0	
**ND_WT**		137	159	LQEKLSPLGEEMRDRARAHVDAL	2633.3708	3.8114	30	20	0.28	2.925	2.925	1.045	2	High	0.116	0.00146432
**ND_WT**		137	159	LQEKLSPLGEEMRDRARAHVDAL	2633.3708	3.785	300	20	2.569	5.172	5.172	0.326	2	High	0.036	0.0267558
**ND_WT**		137	159	LQEKLSPLGEEMRDRARAHVDAL	2633.3708	3.825	3000	20	13.382	7.693	7.693	0.804	2	High	0.09	0.0557229
**ND_WT**		137	159	LQEKLSPLGEEMRDRARAHVDAL	2633.3708	3.8142	9000	20	18.24	8.862	8.862	0.146	2	High	0.016	0.000594985
**ND_WT**		139	158	EKLSPLGEEMRDRARAHVDA	2279.1441	3.1987	0	17	0	0	0	n/a	2	High	0	
**ND_WT**		139	158	EKLSPLGEEMRDRARAHVDA	2279.1441	3.194	30	17	0.251	2.559	3.763	0.378	2	High	0.042	0.00885641
**ND_WT**		139	158	EKLSPLGEEMRDRARAHVDA	2279.1441	3.1647	300	17	2.292	4.443	6.534	0.014	2	Medium	0.002	0.00667338
**ND_WT**		139	158	EKLSPLGEEMRDRARAHVDA	2279.1441	3.1579	3000	17	11.614	7.141	10.502	0.529	2	Medium	0.059	0.0225633
**ND_WT**		139	158	EKLSPLGEEMRDRARAHVDA	2279.1441	3.1785	9000	17	15.576	7.721	11.354	0.122	2	Medium	0.014	0.0713418
**ND_WT**		140	146	KLSPLGE	742.4225	2.9034	0	4	0	0	0	n/a	2	Medium	0	
**ND_WT**		140	146	KLSPLGE	742.4225	2.9065	30	4	0.035	1.007	25.186	0.333	2	Medium	0.037	0.00173789
**ND_WT**		140	146	KLSPLGE	742.4225	2.9013	300	4	0.334	1.403	35.086	0.163	2	Medium	0.018	0.0207091
**ND_WT**		140	146	KLSPLGE	742.4225	2.9167	3000	4	2.158	1.831	45.787	0.481	2	Medium	0.054	0.924967
**ND_WT**		140	146	KLSPLGE	742.4225	2.9077	9000	4	3.363	1.791	44.781	0.002	2	Medium	0	0.177523
**ND_WT**		140	147	KLSPLGEE	871.4651	2.9915	0	5	0	0	0	n/a	2	High	0	
**ND_WT**		140	147	KLSPLGEE	871.4651	2.9773	30	5	0.055	1.224	12.242	0.469	2	Medium	0.052	0.00266031
**ND_WT**		140	147	KLSPLGEE	871.4651	2.9668	300	5	0.516	1.795	17.952	0.069	2	Medium	0.008	0.0120968
**ND_WT**		140	147	KLSPLGEE	871.4651	2.9976	3000	5	3.025	2.293	22.932	0.69	2	Medium	0.077	0.647108
**ND_WT**		140	147	KLSPLGEE	871.4651	2.9937	9000	5	4.361	2.283	22.828	0.124	2	Medium	0.014	0.637108
**ND_WT**		140	148	KLSPLGEEM	1002.5056	3.7126	0	6	0	0	0	n/a	2	Medium	0	
**ND_WT**		140	148	KLSPLGEEM	1002.5056	3.7547	30	6	0.067	1.132	9.436	0.033	2	Medium	0.004	0.0111067
**ND_WT**		140	148	KLSPLGEEM	1002.5056	3.7463	300	6	0.629	1.813	15.104	0.05	2	Medium	0.006	2.40E-05
**ND_WT**		140	148	KLSPLGEEM	1002.5056	3.7549	3000	6	3.723	2.531	21.094	0.17	2	Medium	0.019	0.136086
**ND_WT**		140	148	KLSPLGEEM	1002.5056	3.7416	9000	6	5.333	2.532	21.101	0.527	2	Medium	0.059	0.0603113
**ND_WT**		147	158	EMRDRARAHVDA	1425.6895	1.4553	0	10	0	0	0	n/a	2	Medium	0	
**ND_WT**		147	158	EMRDRARAHVDA	1425.6895	1.46	30	10	0.181	1.274	3.184	0.472	2	Medium	0.052	0.0582926
**ND_WT**		147	158	EMRDRARAHVDA	1425.6895	1.4328	300	10	1.632	2.277	5.692	0.792	2	Medium	0.088	0.0254908
**ND_WT**		147	158	EMRDRARAHVDA	1425.6895	1.4287	3000	10	7.626	3.499	8.746	0.282	2	Medium	0.031	0.000589474
**ND_WT**		147	158	EMRDRARAHVDA	1425.6895	1.4189	9000	10	9.641	4.533	11.332	0.094	2	Medium	0.01	0.00929246
**ND_WT**		147	159	EMRDRARAHVDAL	1538.7736	2.3169	0	11	0	0	0	n/a	2	Medium	0	
**ND_WT**		147	159	EMRDRARAHVDAL	1538.7736	2.312	30	11	0.185	1.084	2.859	0.192	4	Medium	0.121	0.107049
**ND_WT**		147	159	EMRDRARAHVDAL	1538.7736	2.3094	300	11	1.672	2.21	5.774	0.763	4	Medium	0.48	0.0134953
**ND_WT**		147	159	EMRDRARAHVDAL	1538.7736	2.3102	3000	11	7.965	3.212	8.499	0.102	4	Medium	0.064	1.50E-05
**ND_WT**		147	159	EMRDRARAHVDAL	1538.7736	2.318	9000	11	10.352	3.79	10.007	0.22	4	Medium	0.138	8.49E-06
**ND_WT**		147	169	EMRDRARAHVDALRTHLAPYSDE	2708.3201	3.116	0	20	0	0	0	n/a	2	High	0	
**ND_WT**		147	169	EMRDRARAHVDALRTHLAPYSDE	2708.3201	3.0979	30	20	0.353	3.176	3.971	1.433	2	High	0.16	0.0215964
**ND_WT**		147	169	EMRDRARAHVDALRTHLAPYSDE	2708.3201	3.1048	300	20	3.141	4.583	5.728	0.158	2	Medium	0.018	0.0128547
**ND_WT**		147	169	EMRDRARAHVDALRTHLAPYSDE	2708.3201	3.0969	3000	20	14.157	6.611	8.264	0.551	2	Medium	0.061	0.00463378
**ND_WT**		147	169	EMRDRARAHVDALRTHLAPYSDE	2708.3201	3.0861	9000	20	18.62	7.917	9.896	1.65	2	Medium	0.184	0.0229435
**ND_WT**		148	158	MRDRARAHVDA	1296.6469	1.4327	0	9	0	0	0	n/a	2	High	0	
**ND_WT**		148	158	MRDRARAHVDA	1296.6469	1.4115	30	9	0.173	1.028	2.855	0.408	2	High	0.045	0.0748754
**ND_WT**		148	158	MRDRARAHVDA	1296.6469	1.4287	300	9	1.552	1.442	4.005	0.358	2	High	0.04	0.00591666
**ND_WT**		148	158	MRDRARAHVDA	1296.6469	1.4193	3000	9	7.061	2.46	6.833	0.276	2	High	0.031	0.00854753
**ND_WT**		148	158	MRDRARAHVDA	1296.6469	1.4388	9000	9	8.723	2.795	7.764	0.129	2	High	0.014	0.00230771
**ND_WT**		148	159	MRDRARAHVDAL	1409.731	2.0479	0	10	0	0	0	n/a	2	High	0	
**ND_WT**		148	159	MRDRARAHVDAL	1409.731	2.0438	30	10	0.177	0.92	2.3	0.536	2	High	0.06	0.14481
**ND_WT**		148	159	MRDRARAHVDAL	1409.731	2.0362	300	10	1.593	1.424	3.56	0.302	2	High	0.034	0.0149848
**ND_WT**		148	159	MRDRARAHVDAL	1409.731	2.0258	3000	10	7.4	2.557	6.392	0.507	2	High	0.056	0.00261458
**ND_WT**		148	159	MRDRARAHVDAL	1409.731	2.0366	9000	10	9.434	2.989	7.473	0.528	2	High	0.059	0.00281806
**ND_WT**		148	169	MRDRARAHVDALRTHLAPYSDE	2579.2775	2.8758	0	19	0	0	0	n/a	2	Medium	0	
**ND_WT**		148	169	MRDRARAHVDALRTHLAPYSDE	2579.2775	2.8808	30	19	0.345	2.748	3.269	0.548	4	Medium	0.345	0.00042257
**ND_WT**		148	169	MRDRARAHVDALRTHLAPYSDE	2579.2775	2.8748	300	19	3.061	3.968	4.718	0.367	4	Medium	0.23	3.47E-06
**ND_WT**		148	169	MRDRARAHVDALRTHLAPYSDE	2579.2775	2.8576	3000	19	13.592	5.842	6.938	0.287	4	Medium	0.18	1.73E-05
**ND_WT**		148	169	MRDRARAHVDALRTHLAPYSDE	2579.2775	2.8655	9000	19	17.702	6.353	7.552	0.441	4	Medium	0.277	0.00331861
**ND_WT**		149	158	RDRARAHVDA	1165.6065	1.4415	0	8	0	0	0	n/a	2	Medium	0	
**ND_WT**		149	158	RDRARAHVDA	1165.6065	1.4253	30	8	0.136	0.862	3.593	0.751	2	Medium	0.084	0.909595
**ND_WT**		149	158	RDRARAHVDA	1165.6065	1.4462	300	8	1.24	1.281	5.336	0.124	2	Medium	0.014	0.00714943
**ND_WT**		149	158	RDRARAHVDA	1165.6065	1.4115	3000	8	6.085	2.248	9.367	0.069	2	Medium	0.008	0.00277134
**ND_WT**		149	158	RDRARAHVDA	1165.6065	1.4228	9000	8	7.723	2.541	10.587	0.03	2	Medium	0.003	0.0104103
**ND_WT**		155	169	HVDALRTHLAPYSDE	1722.8325	3.0655	0	12	0	0	0	n/a	2	Medium	0	
**ND_WT**		155	169	HVDALRTHLAPYSDE	1722.8325	3.0673	30	12	0.216	2.431	6.752	0.63	2	High	0.07	0.00293552
**ND_WT**		155	169	HVDALRTHLAPYSDE	1722.8325	3.0511	300	12	1.907	3.913	10.869	0.675	2	Medium	0.075	0.00572654
**ND_WT**		155	169	HVDALRTHLAPYSDE	1722.8325	3.0496	3000	12	8.293	4.433	12.314	0.599	2	Medium	0.067	0.0101581
**ND_WT**		155	169	HVDALRTHLAPYSDE	1722.8325	3.0504	9000	12	10.974	4.534	12.596	0.269	2	Medium	0.03	0.0058497
**ND_WT**		155	170	HVDALRTHLAPYSDEL	1835.9166	3.786	0	13	0	0	0	n/a	2	Medium	0	
**ND_WT**		155	170	HVDALRTHLAPYSDEL	1835.9166	3.7867	30	13	0.221	2.677	6.863	0.089	2	Medium	0.01	3.12E-05
**ND_WT**		155	170	HVDALRTHLAPYSDEL	1835.9166	3.7774	300	13	1.951	3.672	9.414	1.461	2	Medium	0.163	0.02085
**ND_WT**		155	170	HVDALRTHLAPYSDEL	1835.9166	3.7806	3000	13	8.657	4.68	12	0.929	2	Medium	0.103	0.00133225
**ND_WT**		155	170	HVDALRTHLAPYSDEL	1835.9166	3.7919	9000	13	11.716	5.063	12.981	0.251	2	Medium	0.028	0.000396119

**ND_WT**		159	168	LRTHLAPYSD	1171.5986	2.6689	0	7	0	0	0	n/a	2	Medium	0	
**ND_WT**		159	168	LRTHLAPYSD	1171.5986	2.639	30	7	0.131	1.904	13.597	0.113	2	Medium	0.013	0.000226751
**ND_WT**		159	168	LRTHLAPYSD	1171.5986	2.6434	300	7	1.143	2.275	16.253	0.147	2	Medium	0.016	0.00403226
**ND_WT**		159	168	LRTHLAPYSD	1171.5986	2.6571	3000	7	4.818	2.597	18.549	0.138	2	Medium	0.015	0.000805816
**ND_WT**		159	168	LRTHLAPYSD	1171.5986	2.6599	9000	7	6.467	2.671	19.081	0.509	2	Medium	0.057	0.0530886
**ND_WT**		159	169	LRTHLAPYSDE	1300.6412	2.784	0	8	0	0	0	n/a	2	High	0	
**ND_WT**		159	169	LRTHLAPYSDE	1300.6412	2.7242	30	8	0.163	1.853	11.579	0.519	2	High	0.058	0.00902292
**ND_WT**		159	169	LRTHLAPYSDE	1300.6412	2.7446	300	8	1.416	2.364	14.777	0.081	2	High	0.009	3.01E-05
**ND_WT**		159	169	LRTHLAPYSDE	1300.6412	2.7259	3000	8	5.777	2.748	17.174	0.352	2	High	0.039	0.0108422
**ND_WT**		159	169	LRTHLAPYSDE	1300.6412	2.7422	9000	8	7.467	2.83	17.691	0.356	2	High	0.04	0.00294589
**ND_WT**		159	170	LRTHLAPYSDEL	1413.7252	3.7079	0	9	0	0	0	n/a	2	High	0	
**ND_WT**		159	170	LRTHLAPYSDEL	1413.7252	3.6977	30	9	0.167	2.004	11.134	0.633	2	High	0.07	0.00882395
**ND_WT**		159	170	LRTHLAPYSDEL	1413.7252	3.6782	300	9	1.46	2.651	14.729	0.051	2	High	0.006	0.00023059
**ND_WT**		159	170	LRTHLAPYSDEL	1413.7252	3.6966	3000	9	6.14	3.204	17.803	0.523	2	High	0.058	0.0147307
**ND_WT**		159	170	LRTHLAPYSDEL	1413.7252	3.691	9000	9	8.21	3.304	18.356	0.309	2	High	0.034	0.000670023
**ND_WT**		159	173	LRTHLAPYSDELRQR	1853.986	3.3958	0	12	0	0	0	n/a	2	Medium	0	
**ND_WT**		159	173	LRTHLAPYSDELRQR	1853.986	3.3715	30	12	0.193	2.181	4.543	1.254	2	Medium	0.14	0.0149551
**ND_WT**		159	173	LRTHLAPYSDELRQR	1853.986	3.3639	300	12	1.705	3.379	7.039	0.62	2	Medium	0.069	0.0111593
**ND_WT**		159	173	LRTHLAPYSDELRQR	1853.986	3.3488	3000	12	7.827	4.725	9.844	0.447	2	Medium	0.05	0.000391382
**ND_WT**		159	173	LRTHLAPYSDELRQR	1853.986	3.3677	9000	12	10.913	4.837	10.078	0.268	2	Medium	0.03	0.00473828
**ND_WT**		159	174	LRTHLAPYSDELRQRL	1967.0701	3.5197	0	13	0	0	0	n/a	2	High	0	
**ND_WT**		159	174	LRTHLAPYSDELRQRL	1967.0701	3.5203	30	13	0.196	2.426	4.222	0.334	4	High	0.21	0.00020071
**ND_WT**		159	174	LRTHLAPYSDELRQRL	1967.0701	3.5135	300	13	1.738	3.667	6.382	0.335	4	High	0.211	6.18E-06
**ND_WT**		159	174	LRTHLAPYSDELRQRL	1967.0701	3.5054	3000	13	8.113	4.832	8.396	0.327	4	High	0.206	5.34E-07
**ND_WT**		159	174	LRTHLAPYSDELRQRL	1967.0701	3.5159	9000	13	11.55	5.053	8.773	0.281	4	High	0.176	7.58E-07
**ND_WT**		159	176	LRTHLAPYSDELRQRLAA	2109.1443	3.5657	0	15	0	0	0	n/a	2	High	0	
**ND_WT**		159	176	LRTHLAPYSDELRQRLAA	2109.1443	3.5001	30	15	0.222	3.601	6.002	0.316	2	Medium	0.035	0.00151691
**ND_WT**		159	176	LRTHLAPYSDELRQRLAA	2109.1443	3.4961	300	15	1.984	5.061	8.435	0.36	2	High	0.04	0.00203472
**ND_WT**		159	176	LRTHLAPYSDELRQRLAA	2109.1443	3.5031	3000	15	9.564	6.294	10.489	0.839	2	High	0.093	0.00435842
**ND_WT**		159	176	LRTHLAPYSDELRQRLAA	2109.1443	3.5125	9000	15	13.502	6.407	10.678	0.059	2	High	0.007	0.0200336
**ND_WT**		159	178	LRTHLAPYSDELRQRLAARL	2378.3295	4.0317	0	17	0	0	0	n/a	2	High	0	
**ND_WT**		159	178	LRTHLAPYSDELRQRLAARL	2378.3295	3.9886	30	17	0.234	2.819	3.04	0.735	4	Medium	0.462	0.00246996
**ND_WT**		159	178	LRTHLAPYSDELRQRLAARL	2378.3295	3.9718	300	17	2.096	5.162	5.547	0.392	4	High	0.246	5.96E-05
**ND_WT**		159	178	LRTHLAPYSDELRQRLAARL	2378.3295	3.9751	3000	17	10.407	6.875	7.396	0.374	4	High	0.235	5.31E-06
**ND_WT**		159	178	LRTHLAPYSDELRQRLAARL	2378.3295	3.9837	9000	17	15.052	7.144	7.689	0.307	4	High	0.193	9.90E-07
**ND_WT**		159	180	LRTHLAPYSDELRQRLAARLEA	2578.4092	4.0751	0	19	0	0	0	n/a	2	High	0	
**ND_WT**		159	180	LRTHLAPYSDELRQRLAARLEA	2578.4092	4.0665	30	19	0.257	2.376	3.126	2.716	2	High	0.302	0.0757511
**ND_WT**		159	180	LRTHLAPYSDELRQRLAARLEA	2578.4092	4.0395	300	19	2.317	5.3	6.973	0.736	2	High	0.082	0.000222271
**ND_WT**		159	180	LRTHLAPYSDELRQRLAARLEA	2578.4092	4.0269	3000	19	11.715	7.226	9.508	1.931	2	High	0.215	0.000619955
**ND_WT**		159	180	LRTHLAPYSDELRQRLAARLEA	2578.4092	4.0328	9000	19	16.914	7.682	10.108	1.326	2	High	0.148	5.17E-05
**ND_WT**		160	169	RTHLAPYSDE	1187.5571	2.3852	0	7	0	0	0	n/a	2	High	0	
**ND_WT**		160	169	RTHLAPYSDE	1187.5571	2.3654	30	7	0.156	1.59	11.357	0.647	2	High	0.072	0.0321723
**ND_WT**		160	169	RTHLAPYSDE	1187.5571	2.3815	300	7	1.347	2.064	14.743	0.097	2	Medium	0.011	5.66E-05
**ND_WT**		160	169	RTHLAPYSDE	1187.5571	2.3704	3000	7	5.264	2.429	17.353	0.387	2	High	0.043	0.00483062
**ND_WT**		160	169	RTHLAPYSDE	1187.5571	2.3744	9000	7	6.583	2.5	17.859	0.319	2	High	0.036	0.00171334
**ND_WT**		160	174	RTHLAPYSDELRQRL	1853.986	3.3958	0	12	0	0	0	n/a	2	Medium	0	
**ND_WT**		160	174	RTHLAPYSDELRQRL	1853.986	3.3715	30	12	0.189	2.181	4.543	1.254	2	Medium	0.14	0.0149718
**ND_WT**		160	174	RTHLAPYSDELRQRL	1853.986	3.4056	300	12	1.668	3.053	6.361	0.531	2	Medium	0.059	0.0106211
**ND_WT**		160	174	RTHLAPYSDELRQRL	1853.986	3.3502	3000	12	7.6	4.702	9.797	0.739	2	Medium	0.082	0.00289506
**ND_WT**		160	174	RTHLAPYSDELRQRL	1853.986	3.3677	9000	12	10.665	4.837	10.078	0.268	2	Medium	0.03	0.00473997
**ND_WT**		170	176	LRQRLAA	826.5137	1.8088	0	5	0	0	0	n/a	2	High	0	
**ND_WT**		170	176	LRQRLAA	826.5137	1.7818	30	5	0.05	1.15	11.497	0.277	2	High	0.031	0.00838812
**ND_WT**		170	176	LRQRLAA	826.5137	1.7954	300	5	0.472	1.577	15.767	0.067	2	High	0.007	4.83E-05
**ND_WT**		170	176	LRQRLAA	826.5137	1.7967	3000	5	3.008	2.143	21.429	0.615	2	High	0.068	0.014889
**ND_WT**		170	176	LRQRLAA	826.5137	1.8138	9000	5	4.492	2.215	22.15	0.278	2	High	0.031	0.000773486
**ND_WT**		170	177	LRQRLAAR	982.6148	2.2354	0	6	0	0	0	n/a	2	High	0	
**ND_WT**		170	177	LRQRLAAR	982.6148	2.2542	30	6	0.058	1.358	7.542	0.454	2	High	0.05	0.0185378
**ND_WT**		170	177	LRQRLAAR	982.6148	2.2347	300	6	0.55	1.913	10.626	0.026	2	High	0.003	1.52E-05
**ND_WT**		170	177	LRQRLAAR	982.6148	2.2433	3000	6	3.565	2.608	14.488	0.567	2	High	0.063	0.0137612
**ND_WT**		170	177	LRQRLAAR	982.6148	2.2225	9000	6	5.405	2.783	15.46	0.369	2	High	0.041	0.00643535
**ND_WT**		170	178	LRQRLAARL	1095.6989	2.5774	0	7	0	0	0	n/a	2	High	0	
**ND_WT**		170	178	LRQRLAARL	1095.6989	2.4899	30	7	0.061	1.536	7.314	0.484	2	High	0.054	0.0100658
**ND_WT**		170	178	LRQRLAARL	1095.6989	2.5049	300	7	0.583	2.249	10.711	0.102	2	High	0.011	4.50E-05
**ND_WT**		170	178	LRQRLAARL	1095.6989	2.498	3000	7	3.851	3.061	14.578	0.293	2	High	0.033	0.0049627
**ND_WT**		170	178	LRQRLAARL	1095.6989	2.4664	9000	7	6.042	3.252	15.487	0.07	2	High	0.008	1.02E-05
**ND_WT**		170	180	LRQRLAARLEA	1295.7786	2.5311	0	9	0	0	0	n/a	2	High	0	
**ND_WT**		170	180	LRQRLAARLEA	1295.7786	2.5053	30	9	0.085	1.683	5.453	0.048	4	High	0.03	4.20E-09

**ND_WT**		170	180	LRQRLAARLEA	1295.7786	2.5194	300	9	0.804	2.604	8.453	0.066	4	High	0.042	3.34E-08
**ND_WT**		170	180	LRQRLAARLEA	1295.7786	2.5124	3000	9	5.159	3.652	11.859	0.232	4	High	0.146	2.09E-05
**ND_WT**		170	180	LRQRLAARLEA	1295.7786	2.5124	9000	9	7.904	3.961	12.849	0.146	4	High	0.092	1.83E-06
**ND_WT**		171	178	RQRLAARL	982.6148	2.2354	0	6	0	0	0	n/a	2	High	0	
**ND_WT**		171	178	RQRLAARL	982.6148	2.2542	30	6	0.051	1.358	7.542	0.454	2	High	0.05	0.0185378
**ND_WT**		171	178	RQRLAARL	982.6148	2.2347	300	6	0.485	1.913	10.626	0.026	2	High	0.003	1.52E-05
**ND_WT**		171	178	RQRLAARL	982.6148	2.2133	3000	6	3.21	2.647	14.704	0.072	2	High	0.008	0.000388644
**ND_WT**		171	178	RQRLAARL	982.6148	2.2225	9000	6	5.088	2.783	15.46	0.369	2	High	0.041	0.00643535
**ND_WT**		171	180	RQRLAARLEA	1182.6945	2.2715	0	8	0	0	0	n/a	2	High	0	
**ND_WT**		171	180	RQRLAARLEA	1182.6945	2.257	30	8	0.074	1.556	6.483	0.623	2	High	0.069	0.0168142
**ND_WT**		171	180	RQRLAARLEA	1182.6945	2.2567	300	8	0.707	2.345	9.769	0.154	2	High	0.017	0.00482986
**ND_WT**		171	180	RQRLAARLEA	1182.6945	2.2728	3000	8	4.517	3.233	13.472	0.032	2	High	0.004	0.00104879
**ND_WT**		171	180	RQRLAARLEA	1182.6945	2.2431	9000	8	6.95	3.635	15.145	0.238	2	High	0.026	0.000250525
**ND_WT**		179	189	EALKENGGARL	1156.62	1.9997	0	9	0	0	0	n/a	2	Medium	0	
**ND_WT**		179	189	EALKENGGARL	1156.62	1.9922	30	9	0.121	4.607	38.602	0.367	4	Medium	0.231	0.143456
**ND_WT**		179	189	EALKENGGARL	1156.62	1.9884	300	9	1.106	4.323	36.311	0.21	4	Medium	0.132	0.721325
**ND_WT**		179	189	EALKENGGARL	1156.62	1.989	3000	9	5.692	4.426	37.156	0.183	4	Medium	0.115	0.895247
**ND_WT**		179	189	EALKENGGARL	1156.62	1.99	9000	9	7.965	4.355	36.622	0.221	4	Medium	0.139	0.31812
**ND_WT**		179	191	EALKENGGARLAE	1356.6997	2.2578	0	11	0	0	0	n/a	2	High	0	
**ND_WT**		179	191	EALKENGGARLAE	1356.6997	2.2375	30	11	0.142	5.469	24.858	0.047	2	High	0.005	0.156177
**ND_WT**		179	191	EALKENGGARLAE	1356.6997	2.2412	300	11	1.308	5.352	24.329	0.009	2	High	0.001	0.118038
**ND_WT**		179	191	EALKENGGARLAE	1356.6997	2.2383	3000	11	7.002	5.654	25.702	0.342	2	High	0.038	0.517819
**ND_WT**		179	191	EALKENGGARLAE	1356.6997	2.2458	9000	11	9.883	5.586	25.39	1.067	2	High	0.119	0.379696
**ND_WT**		179	203	EALKENGGARLAEYHAKATEHLSTL	2708.3882	3.2993	0	23	0	0	0	n/a	2	High	0	
**ND_WT**		179	203	EALKENGGARLAEYHAKATEHLSTL	2708.3882	3.2842	30	23	0.348	6.991	10.132	0.14	2	Medium	0.016	0.00498083
**ND_WT**		179	203	EALKENGGARLAEYHAKATEHLSTL	2708.3882	3.2654	300	23	3.083	9.82	14.231	1.038	2	Medium	0.116	0.0053607
**ND_WT**		179	203	EALKENGGARLAEYHAKATEHLSTL	2708.3882	3.2537	3000	23	14.85	11.555	16.747	0.588	2	High	0.065	0.774013
**ND_WT**		179	203	EALKENGGARLAEYHAKATEHLSTL	2708.3882	3.2812	9000	23	20.808	11.757	17.04	0.399	2	High	0.044	0.131965
**ND_WT**		181	190	LKENGGARLA	1027.5774	1.8956	0	8	0	0	0	n/a	2	Medium	0	
**ND_WT**		181	190	LKENGGARLA	1027.5774	1.8867	30	8	0.123	4.138	21.545	0.116	4	Medium	0.073	0.0326355
**ND_WT**		181	190	LKENGGARLA	1027.5774	1.8765	300	8	1.122	3.776	19.669	0.069	4	Medium	0.044	0.232393
**ND_WT**		181	190	LKENGGARLA	1027.5774	1.8716	3000	8	5.638	3.832	19.958	0.022	4	Medium	0.014	0.519753
**ND_WT**		181	190	LKENGGARLA	1027.5774	1.8831	9000	8	7.456	3.733	19.445	0.045	4	Medium	0.028	0.0673586
**ND_WT**		181	191	LKENGGARLAE	1156.62	1.9948	0	9	0	0	0	n/a	2	High	0	
**ND_WT**		181	191	LKENGGARLAE	1156.62	2.017	30	9	0.133	4.543	16.826	0.543	2	High	0.06	0.0568111
**ND_WT**		181	191	LKENGGARLAE	1156.62	1.9955	300	9	1.221	4.297	15.916	0.402	2	High	0.045	0.116356
**ND_WT**		181	191	LKENGGARLAE	1156.62	1.9903	3000	9	6.285	4.397	16.286	0.14	2	High	0.016	0.0981837
**ND_WT**		181	191	LKENGGARLAE	1156.62	1.9916	9000	9	8.412	4.301	15.931	0.218	2	High	0.024	0.02632
**ND_WT**		190	195	AEYHAK	717.3446	1.4189	0	4	0	0	0	n/a	2	Medium	0	
**ND_WT**		190	195	AEYHAK	717.3446	1.4004	30	4	0.08	1.982	24.78	0.157	2	Medium	0.017	0.00329267
**ND_WT**		190	195	AEYHAK	717.3446	1.4121	300	4	0.707	1.912	23.899	0.267	2	Medium	0.03	0.150149
**ND_WT**		190	195	AEYHAK	717.3446	1.3972	3000	4	2.992	1.932	24.153	0.15	2	Medium	0.017	0.121343
**ND_WT**		190	195	AEYHAK	717.3446	1.4003	9000	4	3.795	1.885	23.567	0.309	2	Medium	0.034	0.39586
**ND_WT**		190	200	AEYHAKATEHL	1268.6149	2.0307	0	9	0	0	0	n/a	2	High	0	
**ND_WT**		190	200	AEYHAKATEHL	1268.6149	2.0266	30	9	0.186	2.846	10.542	0.906	2	Medium	0.101	0.0118989
**ND_WT**		190	200	AEYHAKATEHL	1268.6149	2.0205	300	9	1.577	3.508	12.992	1.174	2	Medium	0.131	0.148752
**ND_WT**		190	200	AEYHAKATEHL	1268.6149	2.0351	3000	9	6.422	4.111	15.224	0.655	2	Medium	0.073	0.315696
**ND_WT**		190	200	AEYHAKATEHL	1268.6149	2.0203	9000	9	8.488	4.096	15.169	0.476	2	Medium	0.053	0.27851
**ND_WT**		190	201	AEYHAKATEHLS	1355.647	1.7246	0	10	0	0	0	n/a	2	High	0	
**ND_WT**		190	201	AEYHAKATEHLS	1355.647	1.7018	30	10	0.195	4.548	22.739	0.704	2	High	0.078	0.4499
**ND_WT**		190	201	AEYHAKATEHLS	1355.647	1.713	300	10	1.667	4.398	21.991	0.908	2	High	0.101	0.16909
**ND_WT**		190	201	AEYHAKATEHLS	1355.647	1.7162	3000	10	7.032	4.477	22.387	0.403	2	High	0.045	0.204185
**ND_WT**		190	201	AEYHAKATEHLS	1355.647	1.7192	9000	10	9.428	4.451	22.257	0.046	2	High	0.005	0.357672
**ND_WT**		190	202	AEYHAKATEHLST	1456.6947	1.8599	0	11	0	0	0	n/a	2	High	0	
**ND_WT**		190	202	AEYHAKATEHLST	1456.6947	1.827	30	11	0.203	2.911	13.234	1.19	2	High	0.132	0.00504149
**ND_WT**		190	202	AEYHAKATEHLST	1456.6947	1.8752	300	11	1.747	3.927	17.85	1.292	2	High	0.144	0.0417364
**ND_WT**		190	202	AEYHAKATEHLST	1456.6947	1.8539	3000	11	7.598	4.943	22.47	1.054	2	High	0.117	0.273974
**ND_WT**		190	202	AEYHAKATEHLST	1456.6947	1.92	9000	11	10.347	5.102	23.19	0.788	2	High	0.088	0.79233
**ND_WT**		190	203	AEYHAKATEHLSTL	1569.7787	2.7698	0	12	0	0	0	n/a	2	High	0	
**ND_WT**		190	203	AEYHAKATEHLSTL	1569.7787	2.7533	30	12	0.206	3.079	12.828	0.868	2	High	0.097	0.00146567
**ND_WT**		190	203	AEYHAKATEHLSTL	1569.7787	2.7781	300	12	1.775	4.206	17.525	0.935	2	High	0.104	0.0139605
**ND_WT**		190	203	AEYHAKATEHLSTL	1569.7787	2.7546	3000	12	7.848	5.156	21.484	0.94	2	High	0.105	0.246043
**ND_WT**		190	203	AEYHAKATEHLSTL	1569.7787	2.7614	9000	12	10.925	5.298	22.075	0.394	2	High	0.044	0.393575
**ND_WT**		190	205	AEYHAKATEHLSTLSE	1785.8533	2.563	0	14	0	0	0	n/a	2	High	0	
**ND_WT**		190	205	AEYHAKATEHLSTLSE	1785.8533	2.5438	30	14	0.232	3.435	12.266	0.921	2	High	0.102	0.00244131
**ND_WT**		190	205	AEYHAKATEHLSTLSE	1785.8533	2.5607	300	14	2.016	4.985	17.803	0.187	2	High	0.021	0.00798515
**ND_WT**		190	205	AEYHAKATEHLSTLSE	1785.8533	2.5614	3000	14	9.263	6.375	22.768	1.381	2	Medium	0.154	0.287638
**ND_WT**		190	205	AEYHAKATEHLSTLSE	1785.8533	2.5513	9000	14	12.858	6.61	23.609	1.285	2	High	0.143	0.807431
**ND_WT**		190	211	AEYHAKATEHLSTLSEKAKPAL	2394.2543	2.7009	0	19	0	0	0	n/a	2	High	0	
**ND_WT**		190	211	AEYHAKATEHLSTLSEKAKPAL	2394.2543	2.6607	30	19	0.275	3.71	4.882	1.235	2	High	0.137	0.0034664

ND_WT		190	211	AEYHAKATEHLSTLSEKAKPAL	2394.2543	2.702	300	19	2.434	5.947	7.825	1.129	2	High	0.126	0.00214835
ND_WT		190	211	AEYHAKATEHLSTLSEKAKPAL	2394.2543	2.7025	3000	19	12.095	8.226	10.823	1.687	2	High	0.188	0.133259
ND_WT		190	211	AEYHAKATEHLSTLSEKAKPAL	2394.2543	2.7116	9000	19	17.33	8.662	11.397	0.703	2	High	0.078	0.250619
ND_WT		190	212	AEYHAKATEHLSTLSEKAKPALE	2523.2969	2.5953	0	20	0	0	0	n/a	2	High	0	
ND_WT		190	212	AEYHAKATEHLSTLSEKAKPALE	2523.2969	2.5692	30	20	0.282	4.355	7.258	0.553	2	Medium	0.062	0.000335672
ND_WT		190	212	AEYHAKATEHLSTLSEKAKPALE	2523.2969	2.5434	300	20	2.5	6.655	11.091	1.523	2	High	0.169	0.00628353
ND_WT		190	212	AEYHAKATEHLSTLSEKAKPALE	2523.2969	2.5608	3000	20	12.586	8.712	14.519	0.835	2	High	0.093	0.0457465
ND_WT		190	212	AEYHAKATEHLSTLSEKAKPALE	2523.2969	2.5721	9000	20	18.199	9.228	15.381	0.366	2	High	0.041	0.259797
ND_WT		190	213	AEYHAKATEHLSTLSEKAKPALED	2638.3239	2.6228	0	21	0	0	0	n/a	2	High	0	
ND_WT		190	213	AEYHAKATEHLSTLSEKAKPALED	2638.3239	2.5794	30	21	0.335	3.73	5.921	0.311	2	High	0.035	0.000607628
ND_WT		190	213	AEYHAKATEHLSTLSEKAKPALED	2638.3239	2.5952	300	21	2.917	6.268	9.949	0.483	2	High	0.054	0.00937394
ND_WT		190	213	AEYHAKATEHLSTLSEKAKPALED	2638.3239	2.5778	3000	21	13.582	9.219	14.634	0.492	2	High	0.055	0.0285611
ND_WT		190	213	AEYHAKATEHLSTLSEKAKPALED	2638.3239	2.6105	9000	21	19.199	9.702	15.399	0.757	2	High	0.084	0.281161
ND_WT		190	214	AEYHAKATEHLSTLSEKAKPALEDL	2751.4079	3.4981	0	22	0	0	0	n/a	2	High	0	
ND_WT		190	214	AEYHAKATEHLSTLSEKAKPALEDL	2751.4079	3.4798	30	22	0.342	3.287	4.047	0.825	4	High	0.519	1.49E-06
ND_WT		190	214	AEYHAKATEHLSTLSEKAKPALEDL	2751.4079	3.4769	300	22	2.986	6.091	7.449	0.499	4	High	0.314	3.04E-05
ND_WT		190	214	AEYHAKATEHLSTLSEKAKPALEDL	2751.4079	3.4525	3000	22	14.093	9.391	11.444	0.386	4	High	0.243	0.00418446
ND_WT		190	214	AEYHAKATEHLSTLSEKAKPALEDL	2751.4079	3.47	9000	22	20.082	10.043	12.229	0.374	4	High	0.235	0.11678
ND_WT		190	219	AEYHAKATEHLSTLSEKAKPALEDLRQGLL	3318.7572	4.13	0	27	0	0	0	n/a	2	High	0	
ND_WT		190	219	AEYHAKATEHLSTLSEKAKPALEDLRQGLL	3318.7572	4.1047	30	27	0.377	3.212	2.974	1.154	2	High	0.128	0.000187516
ND_WT		190	219	AEYHAKATEHLSTLSEKAKPALEDLRQGLL	3318.7572	4.1226	300	27	3.326	6.612	6.123	0.765	2	High	0.085	0.00111382
ND_WT		190	219	AEYHAKATEHLSTLSEKAKPALEDLRQGLL	3318.7572	4.099	3000	27	16.525	10.965	10.153	1.345	2	High	0.15	0.024183
ND_WT		190	219	AEYHAKATEHLSTLSEKAKPALEDLRQGLL	3318.7572	4.0936	9000	27	24.236	12.354	11.439	1.588	2	High	0.177	0.0435611
ND_WT		190	222	AEYHAKATEHLSTLSEKAKPALEDLRQGLLPV	3627.9624	4.6571	0	29	0	0	0	n/a	2	Medium	0	
ND_WT		190	222	AEYHAKATEHLSTLSEKAKPALEDLRQGLLPV	3627.9624	4.628	30	29	0.381	2.235	2.569	4.499	2	Medium	0.501	0.00478046
ND_WT		190	222	AEYHAKATEHLSTLSEKAKPALEDLRQGLLPV	3627.9624	4.6085	300	29	3.366	6.602	7.588	0.116	2	Medium	0.013	3.62E-08
ND_WT		190	222	AEYHAKATEHLSTLSEKAKPALEDLRQGLLPV	3627.9624	4.5955	3000	29	16.898	11.84	13.61	1.571	2	Medium	0.175	0.000314508
ND_WT		190	222	AEYHAKATEHLSTLSEKAKPALEDLRQGLLPV	3627.9624	4.6225	9000	29	25.155	13.426	15.433	2.105	2	Medium	0.234	0.0353476
ND_WT		192	203	YHAKATEHLSTL	1369.699	2.609	0	10	0	0	0	n/a	2	Medium	0	
ND_WT		192	203	YHAKATEHLSTL	1369.699	2.5985	30	10	0.166	2.721	9.071	0.891	2	Medium	0.099	0.00274826
ND_WT		192	203	YHAKATEHLSTL	1369.699	2.5897	300	10	1.421	3.708	12.362	0.615	2	Medium	0.068	0.0218944
ND_WT		192	203	YHAKATEHLSTL	1369.699	2.5987	3000	10	6.328	4.37	14.566	0.563	2	Medium	0.063	0.387617
ND_WT		192	203	YHAKATEHLSTL	1369.699	2.6146	9000	10	9.01	4.549	15.163	0.305	2	Medium	0.034	0.0254625
ND_WT		192	211	YHAKATEHLSTLSEKAKPAL	2194.1746	2.5537	0	17	0	0	0	n/a	2	Medium	0	
ND_WT		192	211	YHAKATEHLSTLSEKAKPAL	2194.1746	2.5582	30	17	0.235	3.085	6.049	1.432	2	Medium	0.159	0.0124894
ND_WT		192	211	YHAKATEHLSTLSEKAKPAL	2194.1746	2.5644	300	17	2.08	5.49	10.764	0.837	2	Medium	0.093	0.00388876
ND_WT		192	211	YHAKATEHLSTLSEKAKPAL	2194.1746	2.5456	3000	17	10.575	7.697	15.092	0.511	2	Medium	0.057	0.158401
ND_WT		192	211	YHAKATEHLSTLSEKAKPAL	2194.1746	2.5554	9000	17	15.416	8.211	16.1	2.053	2	Medium	0.228	0.192125
ND_WT		193	211	HAKATEHLSTLSEKAKPAL	2031.1113	2.4172	0	16	0	0	0	n/a	2	Medium	0	
ND_WT		193	211	HAKATEHLSTLSEKAKPAL	2031.1113	2.4097	30	16	0.203	2.714	4.444	0.466	4	Medium	0.293	6.38E-07
ND_WT		193	211	HAKATEHLSTLSEKAKPAL	2031.1113	2.4113	300	16	1.796	5.152	8.537	0.563	4	Medium	0.354	0.000297724
ND_WT		193	211	HAKATEHLSTLSEKAKPAL	2031.1113	2.4125	3000	16	9.611	7.295	12.181	0.43	4	Medium	0.27	0.287416
ND_WT		193	211	HAKATEHLSTLSEKAKPAL	2031.1113	2.4272	9000	16	14.416	7.41	12.32	0.179	4	Medium	0.113	0.861706
ND_WT		199	211	HLSTLSEKAKPAL	1393.7929	2.5629	0	10	0	0	0	n/a	2	Medium	0	
ND_WT		199	211	HLSTLSEKAKPAL	1393.7929	2.5241	30	10	0.09	2.309	7.696	0.413	2	Medium	0.046	0.000223628
ND_WT		199	211	HLSTLSEKAKPAL	1393.7929	2.5656	300	10	0.857	3.433	11.444	0.01	2	Medium	0.001	0.0111907
ND_WT		199	211	HLSTLSEKAKPAL	1393.7929	2.5591	3000	10	5.673	4.901	16.335	0.074	2	Medium	0.008	0.0121055
ND_WT		199	211	HLSTLSEKAKPAL	1393.7929	2.587	9000	10	8.843	4.953	16.509	0.683	2	Medium	0.076	0.113757
ND_WT		201	212	STLSEKAKPALE	1272.6925	2.3166	0	9	0	0	0	n/a	2	Medium	0	
ND_WT		201	212	STLSEKAKPALE	1272.6925	2.3242	30	9	0.079	2.215	12.307	0.215	2	Medium	0.024	0.00260183
ND_WT		201	212	STLSEKAKPALE	1272.6925	2.3309	300	9	0.753	3.624	20.134	0.039	2	Medium	0.004	8.31E-06
ND_WT		201	212	STLSEKAKPALE	1272.6925	2.3303	3000	9	4.989	5.177	28.761	0.066	2	Medium	0.007	0.03282
ND_WT		201	212	STLSEKAKPALE	1272.6925	2.3317	9000	9	7.852	5.55	30.835	0.862	2	Medium	0.096	0.484085
ND_WT		201	219	STLSEKAKPALEDLRQGLL	2068.1528	4.442	0	16	0	0	0	n/a	2	Medium	0	
ND_WT		201	219	STLSEKAKPALEDLRQGLL	2068.1528	4.456	30	16	0.174	3.062	6.379	0.449	2	Medium	0.05	0.00731568
ND_WT		201	219	STLSEKAKPALEDLRQGLL	2068.1528	4.4046	300	16	1.579	4.349	9.06	0.291	2	Medium	0.032	0.00091921
ND_WT		201	219	STLSEKAKPALEDLRQGLL	2068.1528	4.4198	3000	16	8.928	7.482	15.588	0.963	2	Medium	0.107	0.0195548
ND_WT		201	219	STLSEKAKPALEDLRQGLL	2068.1528	4.438	9000	16	13.889	8.752	18.233	0.648	2	Medium	0.072	0.00898879
ND_WT		203	212	LSEKAKPALE	1084.6128	1.9512	0	7	0	0	0	n/a	2	High	0	
ND_WT		203	212	LSEKAKPALE	1084.6128	1.9258	30	7	0.067	1.545	11.037	0.169	2	High	0.019	0.000141764
ND_WT		203	212	LSEKAKPALE	1084.6128	1.95	300	7	0.635	2.277	16.264	0.102	2	Medium	0.011	0.0100158
ND_WT		203	212	LSEKAKPALE	1084.6128	1.9156	3000	7	4.129	3.504	25.028	0.125	2	High	0.014	0.00121396
ND_WT		203	212	LSEKAKPALE	1084.6128	1.9202	9000	7	6.334	3.773	26.948	0.233	2	High	0.026	0.0836345
ND_WT		203	213	LSEKAKPALED	1199.6398	2.0586	0	8	0	0	0	n/a	2	Medium	0	
ND_WT		203	213	LSEKAKPALED	1199.6398	2.0506	30	8	0.119	1.471	9.197	0.395	2	High	0.044	0.000358728
ND_WT		203	213	LSEKAKPALED	1199.6398	2.0479	300	8	1.052	2.433	15.208	0.167	2	Medium	0.019	0.000830396
ND_WT		203	213	LSEKAKPALED	1199.6398	2.0419	3000	8	5.125	3.925	24.531	0.154	2	High	0.017	0.0117717

**ND_WT**		203	213	LSEKAKPALED	1199.6398	2.0627	9000	8	7.334	4.256	26.6	0.075	2	High	0.008	0.279318
**ND_WT**		203	214	LSEKAKPALEDL	1312.7238	3.6483	0	9	0	0	0	n/a	2	Medium	0	
**ND_WT**		203	214	LSEKAKPALEDL	1312.7238	3.6479	30	9	0.126	1.382	7.676	0.224	2	Medium	0.025	0.00252089
**ND_WT**		203	214	LSEKAKPALEDL	1312.7238	3.6503	300	9	1.121	2.299	12.772	0.016	2	Medium	0.002	0.00933637
**ND_WT**		203	214	LSEKAKPALEDL	1312.7238	3.6383	3000	9	5.636	3.985	22.136	0.108	2	Medium	0.012	0.00665296
**ND_WT**		203	214	LSEKAKPALEDL	1312.7238	3.6451	9000	9	8.217	4.38	24.333	0.441	2	Medium	0.049	0.0261599
**ND_WT**		203	219	LSEKAKPALEDLRQGLL	1880.0731	4.2398	0	14	0	0	0	n/a	2	Medium	0	
**ND_WT**		203	219	LSEKAKPALEDLRQGLL	1880.0731	4.236	30	14	0.162	1.406	5.021	1.063	2	High	0.118	0.0015838
**ND_WT**		203	219	LSEKAKPALEDLRQGLL	1880.0731	4.2297	300	14	1.461	3.119	11.138	0.109	2	Medium	0.012	0.0170379
**ND_WT**		203	219	LSEKAKPALEDLRQGLL	1880.0731	4.2139	3000	14	8.068	6.339	22.639	0.247	2	Medium	0.027	0.000300877
**ND_WT**		203	219	LSEKAKPALEDLRQGLL	1880.0731	4.2149	9000	14	12.371	7.436	26.556	0.297	2	Medium	0.033	0.0745027
**ND_WT**		203	222	LSEKAKPALEDLRQGLLPVL	2189.2784	4.949	0	16	0	0	0	n/a	2	High	0	
**ND_WT**		203	222	LSEKAKPALEDLRQGLLPVL	2189.2784	4.9323	30	16	0.166	1.583	3.298	0.708	2	High	0.079	0.00211174
**ND_WT**		203	222	LSEKAKPALEDLRQGLLPVL	2189.2784	4.9389	300	16	1.501	3.419	7.123	0.371	2	High	0.041	0.00218576
**ND_WT**		203	222	LSEKAKPALEDLRQGLLPVL	2189.2784	4.9267	3000	16	8.441	6.844	14.259	0.188	2	High	0.021	0.000107912
**ND_WT**		203	222	LSEKAKPALEDLRQGLLPVL	2189.2784	4.9302	9000	16	13.29	8.225	17.136	0.277	2	Medium	0.031	0.0230365
**ND_WT**		204	211	SEKAKPAL	842.4862	1.8418	0	5	0	0	0	n/a	2	Medium	0	
**ND_WT**		204	211	SEKAKPAL	842.4862	1.8387	30	5	0.044	1.099	10.988	0.003	2	Medium	0	0.00777972
**ND_WT**		204	211	SEKAKPAL	842.4862	1.8328	300	5	0.418	1.711	17.11	0.086	2	Medium	0.01	0.0115472
**ND_WT**		204	211	SEKAKPAL	842.4862	1.8216	3000	5	2.832	2.668	26.675	0.108	2	Medium	0.012	0.0201403
**ND_WT**		204	211	SEKAKPAL	842.4862	1.8306	9000	5	4.473	2.791	27.908	0.114	2	Medium	0.013	0.0161403
**ND_WT**		204	212	SEKAKPALE	971.5288	1.5887	0	6	0	0	0	n/a	2	High	0	
**ND_WT**		204	212	SEKAKPALE	971.5288	1.5559	30	6	0.05	1.251	10.426	0.59	2	High	0.066	0.00132453
**ND_WT**		204	212	SEKAKPALE	971.5288	1.55	300	6	0.483	1.942	16.186	0.741	2	High	0.082	0.00506221
**ND_WT**		204	212	SEKAKPALE	971.5288	1.569	3000	6	3.323	2.98	24.834	0.432	2	High	0.048	0.0183457
**ND_WT**		204	212	SEKAKPALE	971.5288	1.5944	9000	6	5.341	3.254	27.119	0.309	2	High	0.034	0.0960817
**ND_WT**		204	213	SEKAKPALED	1086.5557	1.742	0	7	0	0	0	n/a	2	High	0	
**ND_WT**		204	213	SEKAKPALED	1086.5557	1.7382	30	7	0.103	1.118	7.985	0.347	2	High	0.039	0.000599584
**ND_WT**		204	213	SEKAKPALED	1086.5557	1.7529	300	7	0.901	1.92	13.712	0.201	2	High	0.022	0.0042275
**ND_WT**		204	213	SEKAKPALED	1086.5557	1.7387	3000	7	4.319	3.326	23.76	0.52	2	High	0.058	0.0323733
**ND_WT**		204	213	SEKAKPALED	1086.5557	1.7435	9000	7	6.341	3.669	26.211	0.448	2	High	0.05	0.0919414
**ND_WT**		204	214	SEKAKPALEDL	1199.6398	3.5059	0	8	0	0	0	n/a	2	High	0	
**ND_WT**		204	214	SEKAKPALEDL	1199.6398	3.476	30	8	0.11	1.13	7.066	0.617	2	Medium	0.069	0.000356276
**ND_WT**		204	214	SEKAKPALEDL	1199.6398	3.4631	300	8	0.97	2.08	13.003	0.283	2	Medium	0.032	0.00334927
**ND_WT**		204	214	SEKAKPALEDL	1199.6398	3.4657	3000	8	4.829	3.747	23.419	0.222	2	Medium	0.025	0.00151982
**ND_WT**		204	214	SEKAKPALEDL	1199.6398	3.4776	9000	8	7.224	4.235	26.469	0.202	2	Medium	0.023	0.199918
**ND_WT**		204	219	SEKAKPALEDLRQGLL	1766.989	4.1527	0	13	0	0	0	n/a	2	High	0	
**ND_WT**		204	219	SEKAKPALEDLRQGLL	1766.989	4.1569	30	13	0.145	1.275	4.906	0.374	2	Medium	0.042	3.90E-05
**ND_WT**		204	219	SEKAKPALEDLRQGLL	1766.989	4.1506	300	13	1.309	3.451	13.273	0.707	2	Medium	0.079	0.00290417
**ND_WT**		204	219	SEKAKPALEDLRQGLL	1766.989	4.1131	3000	13	7.262	6.084	23.398	0.321	2	Medium	0.036	0.0147144
**ND_WT**		204	219	SEKAKPALEDLRQGLL	1766.989	4.1216	9000	13	11.378	7.144	27.478	0.399	2	Medium	0.044	0.0351402
**ND_WT**		204	222	SEKAKPALEDLRQGLLPVL	2076.1943	4.9117	0	15	0	0	0	n/a	2	Medium	0	
**ND_WT**		204	222	SEKAKPALEDLRQGLLPVL	2076.1943	4.8992	30	15	0.149	1.324	3.63	0.194	4	Medium	0.122	8.03E-08
**ND_WT**		204	222	SEKAKPALEDLRQGLLPVL	2076.1943	4.879	300	15	1.35	3.098	8.596	0.065	4	Medium	0.041	3.86E-07
**ND_WT**		204	222	SEKAKPALEDLRQGLLPVL	2076.1943	4.8826	3000	15	7.635	6.468	17.994	0.089	4	Medium	0.056	1.03E-09
**ND_WT**		204	222	SEKAKPALEDLRQGLLPVL	2076.1943	4.8895	9000	15	12.297	7.834	21.802	0.135	4	Medium	0.085	1.96E-06
**ND_WT**		206	222	KAKPALEDLRQGLLPVL	1860.1197	4.8376	0	13	0	0	0	n/a	2	High	0	
**ND_WT**		206	222	KAKPALEDLRQGLLPVL	1860.1197	4.8606	30	13	0.127	0.954	2.445	0.824	2	High	0.092	7.55E-07
**ND_WT**		206	222	KAKPALEDLRQGLLPVL	1860.1197	4.8152	300	13	1.132	2.481	6.361	0.102	2	High	0.011	1.37E-06
**ND_WT**		206	222	KAKPALEDLRQGLLPVL	1860.1197	4.8115	3000	13	6.266	5.48	14.051	0.289	2	Medium	0.032	2.32E-05
**ND_WT**		206	222	KAKPALEDLRQGLLPVL	1860.1197	4.8139	9000	13	10.36	6.943	17.804	0.223	2	Medium	0.025	0.00206331
**ND_WT**		212	219	EDLRQGLL	942.5134	4.0568	0	6	0	0	0	n/a	2	Medium	0	
**ND_WT**		212	219	EDLRQGLL	942.5134	4.0478	30	6	0.042	0.609	7.631	0.032	4	Medium	0.02	2.35E-06
**ND_WT**		212	219	EDLRQGLL	942.5134	4.0507	300	6	0.408	1.091	13.654	0.018	4	Medium	0.011	1.10E-06
**ND_WT**		212	219	EDLRQGLL	942.5134	4.0522	3000	6	2.944	2.364	29.604	0.028	4	Medium	0.017	1.01E-10
**ND_WT**		212	219	EDLRQGLL	942.5134	4.0556	9000	6	5.037	2.968	37.171	0.04	4	Medium	0.025	2.06E-07
**ND_WT**		212	222	EDLRQGLLPVL	1251.7187	5.1779	0	8	0	0	0	n/a	2	High	0	
**ND_WT**		212	222	EDLRQGLLPVL	1251.7187	5.1757	30	8	0.046	0.641	4.009	0.348	2	High	0.039	0.00249119
**ND_WT**		212	222	EDLRQGLLPVL	1251.7187	5.1744	300	8	0.449	1.273	7.959	0.286	2	High	0.032	0.00198162
**ND_WT**		212	222	EDLRQGLLPVL	1251.7187	5.1772	3000	8	3.316	2.958	18.486	0.108	2	High	0.012	0.0023244
**ND_WT**		212	222	EDLRQGLLPVL	1251.7187	5.1745	9000	8	5.956	3.881	24.254	0.091	2	High	0.01	0.00159123
**ND_WT**		213	219	DLRQGLL	813.4709	4.1938	0	5	0	0	0	n/a	2	High	0	
**ND_WT**		213	219	DLRQGLL	813.4709	4.1964	30	5	0.035	0.518	10.354	0.193	2	High	0.021	7.15E-05
**ND_WT**		213	219	DLRQGLL	813.4709	4.1876	300	5	0.339	0.956	19.123	0.055	2	High	0.006	0.00137368
**ND_WT**		213	219	DLRQGLL	813.4709	4.168	3000	5	2.433	2.059	41.183	0.02	2	High	0.002	0.000100567
**ND_WT**		213	219	DLRQGLL	813.4709	4.1712	9000	5	4.154	2.582	51.65	0.363	2	Medium	0.04	0.00860196
**ND_WT**		213	222	DLRQGLLPVL	1122.6761	5.2978	0	7	0	0	0	n/a	2	High	0	
**ND_WT**		213	222	DLRQGLLPVL	1122.6761	5.2896	30	7	0.039	0.599	4.277	0.304	2	High	0.034	8.06E-05
**ND_WT**		213	222	DLRQGLLPVL	1122.6761	5.2851	300	7	0.38	1.17	8.356	0.223	2	High	0.025	0.00133929
**ND_WT**		213	222	DLRQGLLPVL	1122.6761	5.2829	3000	7	2.805	2.657	18.982	0.084	2	High	0.009	5.18E-05
**ND_WT**		213	222	DLRQGLLPVL	1122.6761	5.2824	9000	7	5.073	3.472	24.802	0.123	2	High	0.014	0.00503389

**ND_WT**	214	222	LRQGLLPVL	1007.6492	4.9702	0	6	0	0	0	n/a	2	Medium	0	
**ND_WT**	214	222	LRQGLLPVL	1007.6492	4.9595	30	6	0.034	0.617	7.927	0.268	4	Medium	0.168	1.47E-06
**ND_WT**	214	222	LRQGLLPVL	1007.6492	4.9638	300	6	0.328	1.171	15.209	0.257	4	Medium	0.161	6.77E-06
**ND_WT**	214	222	LRQGLLPVL	1007.6492	4.9639	3000	6	2.39	2.309	29.248	0.183	4	Medium	0.115	7.88E-06
**ND_WT**	214	222	LRQGLLPVL	1007.6492	4.9711	9000	6	4.273	3.008	38.066	0.263	4	Medium	0.165	0.00164572
**ND_WT**	215	222	RQGLLPVL	894.5651	4.7963	0	5	0	0	0	n/a	2	High	0	
**ND_WT**	215	222	RQGLLPVL	894.5651	4.7803	30	5	0.024	0.389	3.894	0.132	2	High	0.015	0.00479847
**ND_WT**	215	222	RQGLLPVL	894.5651	4.7741	300	5	0.23	0.714	7.135	0.209	2	High	0.023	0.0041094
**ND_WT**	215	222	RQGLLPVL	894.5651	4.7819	3000	5	1.748	1.619	16.193	0.041	2	High	0.005	2.14E-05
**ND_WT**	215	222	RQGLLPVL	894.5651	4.7818	9000	5	3.319	2.251	22.51	0.078	2	High	0.009	0.0193636
**ND_WT**	223	229	ESFKVSF	842.4174	4.5066	0	5	0	0	0	n/a	2	Medium	0	
**ND_WT**	223	229	ESFKVSF	842.4174	4.517	30	5	0.03	0.454	9.086	0.369	2	Medium	0.041	0.0039904
**ND_WT**	223	229	ESFKVSF	842.4174	4.4874	300	5	0.295	1.022	20.432	0.274	2	Medium	0.03	0.00339301
**ND_WT**	223	229	ESFKVSF	842.4174	4.5053	3000	5	2.211	2.168	43.353	0.043	2	Medium	0.005	0.0208136
**ND_WT**	223	229	ESFKVSF	842.4174	4.5041	9000	5	3.967	2.716	54.32	0.188	2	Medium	0.021	0.0753364
**ND_WT**	226	232	KVSFLSA	750.4276	3.8871	0	5	0	0	0	n/a	2	Medium	0	
**ND_WT**	226	232	KVSFLSA	750.4276	3.8618	30	5	0.041	0.575	11.506	0.296	2	Medium	0.033	0.000211681
**ND_WT**	226	232	KVSFLSA	750.4276	3.8501	300	5	0.393	1.313	26.25	0.211	2	Medium	0.023	0.00365466
**ND_WT**	226	232	KVSFLSA	750.4276	3.8508	3000	5	2.659	2.473	49.464	0.103	2	Medium	0.011	0.000979213
**ND_WT**	226	232	KVSFLSA	750.4276	3.8582	9000	5	4.246	2.77	55.394	0.125	2	Medium	0.014	0.0377594
**ND_WT**	230	235	LSALEE	660.333	3.0193	0	4	0	0	0	n/a	2	Medium	0	
**ND_WT**	230	235	LSALEE	660.333	3.0458	30	4	0.044	0.335	8.38	0.356	2	Medium	0.04	0.00847915
**ND_WT**	230	235	LSALEE	660.333	3.0127	300	4	0.414	0.845	21.135	0.155	2	Medium	0.017	0.00277405
**ND_WT**	230	235	LSALEE	660.333	3.013	3000	4	2.434	1.862	46.539	0.067	2	Medium	0.007	0.00402286
**ND_WT**	230	235	LSALEE	660.333	3.0192	9000	4	3.559	2.216	55.39	0.213	2	Medium	0.024	0.0310022
**ND_WT**	233	243	LEEYTKKLNTQ	1365.714	2.3257	0	9	0	0	0	n/a	2	High	0	
**ND_WT**	233	243	LEEYTKKLNTQ	1365.714	2.3114	30	9	0.072	3.771	20.948	0.174	2	Medium	0.019	0.00296727
**ND_WT**	233	243	LEEYTKKLNTQ	1365.714	2.3244	300	9	0.686	4.551	25.284	0.333	2	High	0.037	0.00922172
**ND_WT**	233	243	LEEYTKKLNTQ	1365.714	2.3264	3000	9	4.527	5.047	28.041	0.219	2	High	0.024	0.875256
**ND_WT**	233	243	LEEYTKKLNTQ	1365.714	2.3355	9000	9	7.358	5.024	27.908	0.136	2	High	0.015	0.417075
**ND_WT**	234	243	EEYTKKLNTQ	1252.6299	1.9054	0	8	0	0	0	n/a	2	Medium	0	
**ND_WT**	234	243	EEYTKKLNTQ	1252.6299	1.8932	30	8	0.052	3.904	48.804	1.822	2	Medium	0.203	0.0404313
**ND_WT**	234	243	EEYTKKLNTQ	1252.6299	1.8982	300	8	0.503	4.507	56.341	0.601	2	Medium	0.067	0.043926
**ND_WT**	234	243	EEYTKKLNTQ	1252.6299	1.8916	3000	8	3.661	4.969	62.114	0.82	2	Medium	0.091	0.908079
**ND_WT**	234	243	EEYTKKLNTQ	1252.6299	1.9116	9000	8	6.361	4.93	61.625	0.279	2	Medium	0.031	0.685489
**ND_WT**	236	243	YTKKLNTQ	994.5447	1.4091	0	6	0	0	0	n/a	2	Medium	0	
**ND_WT**	236	243	YTKKLNTQ	994.5447	1.4103	30	6	0.039	3.375	56.243	0.796	2	Medium	0.089	0.0869211
**ND_WT**	236	243	YTKKLNTQ	994.5447	1.4555	300	6	0.375	3.573	59.556	0.123	2	Medium	0.014	0.0396986
**ND_WT**	236	243	YTKKLNTQ	994.5447	1.4364	3000	6	2.705	3.703	61.72	0.528	2	Medium	0.059	0.107737
**ND_WT**	236	243	YTKKLNTQ	994.5447	1.4476	9000	6	4.666	3.561	59.354	0.671	2	Medium	0.075	0.401382
**ND_L75P**	17	28	VYVDVLKDSGRD	1364.6936	3.3173	0	10	0	0	0	n/a	2	High	0	
**ND_L75P**	17	28	VYVDVLKDSGRD	1364.6936	3.3239	30	10	0.164	1.867	9.336	0.11	2	High	0.012	0.0082187
**ND_L75P**	17	28	VYVDVLKDSGRD	1364.6936	3.324	300	10	1.45	2.845	14.227	0.956	2	High	0.106	0.511303
**ND_L75P**	17	28	VYVDVLKDSGRD	1364.6936	3.3204	3000	10	6.286	4.262	21.311	1.278	2	High	0.142	0.118796
**ND_L75P**	17	28	VYVDVLKDSGRD	1364.6936	3.3133	9000	10	8.411	4.761	23.806	0.635	2	High	0.071	0.0187222
**ND_L75P**	17	46	VYVDVLKDSGRDYVSQFEGSALGKQLNLKL	3340.7667	5.3313	0	28	0	0	0	n/a	2	Medium	0	
**ND_L75P**	17	46	VYVDVLKDSGRDYVSQFEGSALGKQLNLKL	3340.7667	5.3085	30	28	0.322	4.26	3.043	1.009	2	High	0.112	0.322671
**ND_L75P**	17	46	VYVDVLKDSGRDYVSQFEGSALGKQLNLKL	3340.7667	5.3085	300	28	2.951	7.797	5.569	1.781	2	High	0.198	0.0551494
**ND_L75P**	17	46	VYVDVLKDSGRDYVSQFEGSALGKQLNLKL	3340.7667	5.2687	3000	28	16.007	14.994	10.71	3.654	2	Medium	0.407	0.127337
**ND_L75P**	17	46	VYVDVLKDSGRDYVSQFEGSALGKQLNLKL	3340.7667	5.2779	9000	28	23.684	15.806	11.29	0.072	2	High	0.008	0.145787
**ND_L75P**	18	28	YVDVLKDSGRD	1265.6252	3.0611	0	9	0	0	0	n/a	2	High	0	
**ND_L75P**	18	28	YVDVLKDSGRD	1265.6252	3.0602	30	9	0.163	2.204	12.244	0.232	2	High	0.026	0.0520877
**ND_L75P**	18	28	YVDVLKDSGRD	1265.6252	3.0682	300	9	1.432	2.909	16.159	0.6	2	High	0.067	0.130785
**ND_L75P**	18	28	YVDVLKDSGRD	1265.6252	3.0446	3000	9	6.117	4.063	22.57	0.381	2	High	0.042	0.0412499
**ND_L75P**	18	28	YVDVLKDSGRD	1265.6252	3.059	9000	9	7.986	4.284	23.798	0.422	2	High	0.047	0.0386546
**ND_L75P**	19	28	VDVLKDSGRD	1102.5619	2.3197	0	8	0	0	0	n/a	2	High	0	
**ND_L75P**	19	28	VDVLKDSGRD	1102.5619	2.3326	30	8	0.145	2.194	13.715	0.821	2	High	0.091	0.493835
**ND_L75P**	19	28	VDVLKDSGRD	1102.5619	2.3407	300	8	1.269	2.883	18.02	0.608	2	High	0.068	0.0850185
**ND_L75P**	19	28	VDVLKDSGRD	1102.5619	2.2964	3000	8	5.286	3.907	24.419	0.8	2	High	0.089	0.093891
**ND_L75P**	19	28	VDVLKDSGRD	1102.5619	2.33	9000	8	6.991	4.157	25.98	0.37	2	High	0.041	0.023583
**ND_L75P**	19	44	VDVLKDSGRDYVSQFEGSALGKQLNL	2837.4559	5.1768	0	24	0	0	0	n/a	2	High	0	
**ND_L75P**	19	44	VDVLKDSGRDYVSQFEGSALGKQLNL	2837.4559	5.1496	30	24	0.295	3.548	3.696	1.334	2	Medium	0.148	0.305466
**ND_L75P**	19	44	VDVLKDSGRDYVSQFEGSALGKQLNL	2837.4559	5.1313	300	24	2.694	7.673	7.992	0.317	2	High	0.035	0.530544
**ND_L75P**	19	44	VDVLKDSGRDYVSQFEGSALGKQLNL	2837.4559	5.1136	3000	24	14.365	12.872	13.409	0.095	2	High	0.011	0.00629186
**ND_L75P**	19	44	VDVLKDSGRDYVSQFEGSALGKQLNL	2837.4559	5.1084	9000	24	20.902	13.886	14.465	0.938	2	High	0.104	0.130258
**ND_L75P**	20	28	DVLKDSGRD	1003.4934	2.3608	0	7	0	0	0	n/a	2	High	0	
**ND_L75P**	20	28	DVLKDSGRD	1003.4934	2.3082	30	7	0.14	1.944	13.887	0.271	2	High	0.03	0.220704

**ND_L75P**		20	28	DVLKDSGRD	1003.4934	2.3157	300	7	1.219	2.431	17.362	0.457	2	High	0.051	0.355755
**ND_L75P**		20	28	DVLKDSGRD	1003.4934	2.3277	3000	7	4.883	3.295	23.534	0.305	2	High	0.034	0.926979
**ND_L75P**		20	28	DVLKDSGRD	1003.4934	2.347	9000	7	6.203	3.561	25.436	0.354	2	High	0.039	0.358995
**ND_L75P**		29	37	YVSQFEGSA	986.4345	3.2575	0	7	0	0	0	n/a	2	High	0	
**ND_L75P**		29	37	YVSQFEGSA	986.4345	3.2571	30	7	0.086	2.192	31.318	0.547	2	High	0.061	0.0542811
**ND_L75P**		29	37	YVSQFEGSA	986.4345	3.2598	300	7	0.805	2.982	42.594	0.533	2	High	0.059	0.0108782
**ND_L75P**		29	37	YVSQFEGSA	986.4345	3.2627	3000	7	4.753	3.941	56.3	0.154	2	High	0.017	0.00108347
**ND_L75P**		29	37	YVSQFEGSA	986.4345	3.2608	9000	7	6.655	3.969	56.698	0.235	2	High	0.026	0.0989618
**ND_L75P**		29	38	YVSQFEGSAL	1099.5186	4.2645	0	8	0	0	0	n/a	2	High	0	
**ND_L75P**		29	38	YVSQFEGSAL	1099.5186	4.2646	30	8	0.09	2.826	26.444	0.097	4	High	0.061	0.0358834
**ND_L75P**		29	38	YVSQFEGSAL	1099.5186	4.2649	300	8	0.845	3.671	34.323	0.081	4	High	0.051	2.10E-05
**ND_L75P**		29	38	YVSQFEGSAL	1099.5186	4.264	3000	8	5.092	4.563	42.73	0.12	4	High	0.075	2.48E-05
**ND_L75P**		29	38	YVSQFEGSAL	1099.5186	4.2575	9000	8	7.366	4.56	42.761	0.087	4	High	0.054	0.00407945
**ND_L75P**		29	43	YVSQFEGSALGKQLN	1639.8206	3.9033	0	13	0	0	0	n/a	2	High	0	
**ND_L75P**		29	43	YVSQFEGSALGKQLN	1639.8206	3.9595	30	13	0.131	3.675	14.135	0.515	2	Medium	0.057	0.0164641
**ND_L75P**		29	43	YVSQFEGSALGKQLN	1639.8206	3.8899	300	13	1.239	6.07	23.347	0.687	2	Medium	0.077	0.0575477
**ND_L75P**		29	43	YVSQFEGSALGKQLN	1639.8206	3.8948	3000	13	7.811	7.857	30.221	1.55	2	Medium	0.172	0.0839117
**ND_L75P**		29	43	YVSQFEGSALGKQLN	1639.8206	3.8915	9000	13	11.753	7.908	30.415	1.037	2	Medium	0.115	0.0669251
**ND_L75P**		29	44	YVSQFEGSALGKQLNL	1752.9046	4.8252	0	14	0	0	0	n/a	2	High	0	
**ND_L75P**		29	44	YVSQFEGSALGKQLNL	1752.9046	4.8212	30	14	0.135	4.192	14.972	1.045	2	High	0.116	0.0621864
**ND_L75P**		29	44	YVSQFEGSALGKQLNL	1752.9046	4.8057	300	14	1.285	6.272	22.399	0.339	2	High	0.038	0.0191685
**ND_L75P**		29	44	YVSQFEGSALGKQLNL	1752.9046	4.8113	3000	14	8.184	8.347	29.81	0.877	2	High	0.098	0.0277316
**ND_L75P**		29	44	YVSQFEGSALGKQLNL	1752.9046	4.7991	9000	14	12.506	8.42	30.071	0.313	2	High	0.035	0.0601378
**ND_L75P**		29	46	YVSQFEGSALGKQLNLKL	1994.0837	4.8346	0	16	0	0	0	n/a	2	High	0	
**ND_L75P**		29	46	YVSQFEGSALGKQLNLKL	1994.0837	4.8014	30	16	0.143	5.17	16.156	0.489	2	High	0.054	0.0347529
**ND_L75P**		29	46	YVSQFEGSALGKQLNLKL	1994.0837	4.8031	300	16	1.361	7.652	23.914	0.774	2	High	0.086	0.0634687
**ND_L75P**		29	46	YVSQFEGSALGKQLNLKL	1994.0837	4.7955	3000	16	8.826	10.127	31.648	0.992	2	High	0.11	0.0540098
**ND_L75P**		29	46	YVSQFEGSALGKQLNLKL	1994.0837	4.8029	9000	16	13.868	10.11	31.592	1.069	2	High	0.119	0.0918329
**ND_L75P**		33	38	FEGSAL	622.2962	3.5678	0	4	0	0	0	n/a	2	Medium	0	
**ND_L75P**		33	38	FEGSAL	622.2962	3.5639	30	4	0.053	1.891	47.277	0.544	2	Medium	0.061	0.577507
**ND_L75P**		33	38	FEGSAL	622.2962	3.57	300	4	0.492	2.019	50.464	0.021	2	Medium	0.002	0.86022
**ND_L75P**		33	38	FEGSAL	622.2962	3.5757	3000	4	2.714	2.15	53.758	0.281	2	Medium	0.031	0.367981
**ND_L75P**		33	38	FEGSAL	622.2962	3.5728	9000	4	3.676	2.083	52.07	0.3	2	Medium	0.033	0.368388
**ND_L75P**		33	43	FEGSALGKQLN	1162.5982	3.2254	0	9	0	0	0	n/a	2	Medium	0	
**ND_L75P**		33	43	FEGSALGKQLN	1162.5982	3.172	30	9	0.094	3.607	20.038	0.418	2	Medium	0.046	0.0307262
**ND_L75P**		33	43	FEGSALGKQLN	1162.5982	3.186	300	9	0.886	4.425	24.584	0.046	2	Medium	0.005	0.12821
**ND_L75P**		33	43	FEGSALGKQLN	1162.5982	3.2306	3000	9	5.433	5.042	28.01	0.857	2	Medium	0.095	0.744241
**ND_L75P**		33	43	FEGSALGKQLN	1162.5982	3.2039	9000	9	8.062	5.06	28.112	0.177	2	Medium	0.02	0.864713
**ND_L75P**		33	44	FEGSALGKQLNL	1275.6823	4.4252	0	10	0	0	0	n/a	2	Medium	0	
**ND_L75P**		33	44	FEGSALGKQLNL	1275.6823	4.4123	30	10	0.099	3.294	16.47	1.348	2	Medium	0.15	0.0824091
**ND_L75P**		33	44	FEGSALGKQLNL	1275.6823	4.4087	300	10	0.932	4.616	23.082	0.507	2	Medium	0.056	0.109433
**ND_L75P**		33	44	FEGSALGKQLNL	1275.6823	4.4268	3000	10	5.806	5.461	27.306	0.513	2	Medium	0.057	0.11528
**ND_L75P**		33	44	FEGSALGKQLNL	1275.6823	4.4277	9000	10	8.815	5.332	26.66	0.923	2	Medium	0.103	0.087533
**ND_L75P**		33	46	FEGSALGKQLNLKL	1516.8613	4.471	0	12	0	0	0	n/a	2	High	0	
**ND_L75P**		33	46	FEGSALGKQLNLKL	1516.8613	4.4664	30	12	0.106	4.395	18.311	1.195	2	High	0.133	0.0295877
**ND_L75P**		33	46	FEGSALGKQLNLKL	1516.8613	4.4562	300	12	1.008	6.096	25.401	0.404	2	High	0.045	0.175134
**ND_L75P**		33	46	FEGSALGKQLNLKL	1516.8613	4.436	3000	12	6.448	7.742	32.259	0.403	2	High	0.045	0.0108061
**ND_L75P**		33	46	FEGSALGKQLNLKL	1516.8613	4.451	9000	12	10.177	7.531	31.38	0.937	2	High	0.104	0.278687
**ND_L75P**		34	43	EGSALGKQLN	1015.5298	2.4612	0	8	0	0	0	n/a	2	Medium	0	
**ND_L75P**		34	43	EGSALGKQLN	1015.5298	2.4576	30	8	0.08	2.463	15.393	0.27	2	Medium	0.03	0.00716083
**ND_L75P**		34	43	EGSALGKQLN	1015.5298	2.8443	300	8	0.757	3.384	21.149		1	Medium	0	
**ND_L75P**		34	43	EGSALGKQLN	1015.5298	2.4461	3000	8	4.685	4.467	27.918	0.226	2	Medium	0.025	0.23425
**ND_L75P**		34	43	EGSALGKQLN	1015.5298	2.4456	9000	8	7.078	4.556	28.473	0.774	2	Medium	0.086	0.495542
**ND_L75P**		34	44	EGSALGKQLNL	1128.6139	3.9172	0	9	0	0	0	n/a	2	Medium	0	
**ND_L75P**		34	44	EGSALGKQLNL	1128.6139	3.9253	30	9	0.085	2.782	15.455	0.127	2	Medium	0.014	0.0349845
**ND_L75P**		34	44	EGSALGKQLNL	1128.6139	3.9543	300	9	0.803	3.909	21.715	0.583	2	Medium	0.065	0.107809
**ND_L75P**		34	44	EGSALGKQLNL	1128.6139	3.9445	3000	9	5.058	5.195	28.859	0.756	2	Medium	0.084	0.0822601
**ND_L75P**		34	44	EGSALGKQLNL	1128.6139	3.9256	9000	9	7.831	5.107	28.37	0.428	2	Medium	0.048	0.0566522
**ND_L75P**		34	46	EGSALGKQLNLKL	1369.7929	4.141	0	11	0	0	0	n/a	2	High	0	
**ND_L75P**		34	46	EGSALGKQLNLKL	1369.7929	4.1397	30	11	0.093	3.638	16.535	0.628	2	High	0.07	0.015556
**ND_L75P**		34	46	EGSALGKQLNLKL	1369.7929	4.1329	300	11	0.88	5.245	23.841	0.492	2	High	0.055	0.266257
**ND_L75P**		34	46	EGSALGKQLNLKL	1369.7929	4.1334	3000	11	5.7	6.662	30.283	1.134	2	High	0.126	0.145207
**ND_L75P**		34	46	EGSALGKQLNLKL	1369.7929	4.1299	9000	11	9.193	6.601	30.003	0.706	2	High	0.079	0.244165
**ND_L75P**		36	46	SALGKQLNLKL	1183.7289	4.0773	0	9	0	0	0	n/a	2	Medium	0	
**ND_L75P**		36	46	SALGKQLNLKL	1183.7289	4.0774	30	9	0.058	2.85	10.556	0.849	2	Medium	0.094	0.0136323
**ND_L75P**		36	46	SALGKQLNLKL	1183.7289	4.0483	300	9	0.557	4.449	16.478	0.432	2	Medium	0.048	0.176648
**ND_L75P**		36	46	SALGKQLNLKL	1183.7289	4.066	3000	9	4.073	5.85	21.665	0.745	2	Medium	0.083	0.0959915
**ND_L75P**		36	46	SALGKQLNLKL	1183.7289	4.0627	9000	9	7.213	5.998	22.213	0.395	2	Medium	0.044	0.126002
**ND_L75P**		38	44	LGKQLNL	784.4807	3.4433	0	5	0	0	0	n/a	2	Medium	0	
**ND_L75P**		38	44	LGKQLNL	784.4807	3.4187	30	5	0.038	1.409	14.088	0.572	2	Medium	0.064	0.0371271

**ND_L75P**		38	44	LGKQLNL	784.4807	3.401	300	5	0.367	2.153	21.53	0.071		2	Medium	0.008	0.0262044
**ND_L75P**		38	44	LGKQLNL	784.4807	3.4301	3000	5	2.565	2.987	29.873	0.242		2	Medium	0.027	0.133663
**ND_L75P**		38	44	LGKQLNL	784.4807	3.408	9000	5	4.246	2.96	29.597	0.283		2	Medium	0.032	0.211432
**ND_L75P**		38	46	LGKQLNLKL	1025.6597	3.7746	0	7	0	0	0	n/a		2	Medium	0	
**ND_L75P**		38	46	LGKQLNLKL	1025.6597	3.8032	30	7	0.046	2.048	14.628	0.344		2	Medium	0.038	0.024704
**ND_L75P**		38	46	LGKQLNLKL	1025.6597	3.8087	300	7	0.444	3.26	23.284	0.437		2	Medium	0.049	0.876125
**ND_L75P**		38	46	LGKQLNLKL	1025.6597	3.7655	3000	7	3.208	4.371	31.224	0.462		2	Medium	0.051	0.0764685
**ND_L75P**		38	46	LGKQLNLKL	1025.6597	3.7832	9000	7	5.608	4.312	30.801	0.475		2	Medium	0.053	0.484038
**ND_L75P**		39	44	GKQLNL	671.3966	3.0564	0	4	0	0	0	n/a		2	Medium	0	
**ND_L75P**		39	44	GKQLNL	671.3966	3.0481	30	4	0.027	1.192	29.793	0.521		2	Medium	0.058	0.0273782
**ND_L75P**		39	44	GKQLNL	671.3966	3.0502	300	4	0.263	1.856	46.41	0.077		2	Medium	0.009	0.169493
**ND_L75P**		39	44	GKQLNL	671.3966	3.0592	3000	4	1.898	2.522	63.042	0.397		2	Medium	0.044	0.231639
**ND_L75P**		39	44	GKQLNL	671.3966	2.9911	9000	4	3.283	2.407	60.163	0.024		2	Medium	0.003	0.143798
**ND_L75P**		39	46	GKQLNLKL	912.5757	3.5679	0	6	0	0	0	n/a		2	Medium	0	
**ND_L75P**		39	46	GKQLNLKL	912.5757	3.556	30	6	0.035	1.928	32.141	0.673		2	Medium	0.075	0.0320118
**ND_L75P**		39	46	GKQLNLKL	912.5757	3.562	300	6	0.34	2.891	48.185	0.269		2	Medium	0.03	0.121972
**ND_L75P**		39	46	GKQLNLKL	912.5757	3.565	3000	6	2.541	3.824	63.738	0.405		2	Medium	0.045	0.0793582
**ND_L75P**		39	46	GKQLNLKL	912.5757	3.5633	9000	6	4.644	3.799	63.321	0.401		2	Medium	0.045	0.504435
**ND_L75P**		45	50	KLLDNW	787.4228	4.7751	0	4	0	0	0	n/a		2	Medium	0	
**ND_L75P**		45	50	KLLDNW	787.4228	4.5157	30	4	0.08	1.334	33.362	0.421		2	Medium	0.047	0.0127133
**ND_L75P**		45	50	KLLDNW	787.4228	4.5109	300	4	0.674	1.844	46.093	0.031		2	Medium	0.003	0.00916692
**ND_L75P**		45	50	KLLDNW	787.4228	4.5125	3000	4	2.557	2.246	56.14	0.407		2	Medium	0.045	0.307694
**ND_L75P**		45	50	KLLDNW	787.4228	4.5095	9000	4	3.449	2.2	55.012	0.251		2	Medium	0.028	0.645032
**ND_L75P**		45	51	KLLDNWD	902.4498	4.2231	0	5	0	0	0	n/a		2	Medium	0	
**ND_L75P**		45	51	KLLDNWD	902.4498	4.1948	30	5	0.098	1.714	17.138	0.374		2	Medium	0.042	0.00328735
**ND_L75P**		45	51	KLLDNWD	902.4498	4.1931	300	5	0.84	2.154	21.543	0.154		2	Medium	0.017	0.00578771
**ND_L75P**		45	51	KLLDNWD	902.4498	4.1895	3000	5	3.395	2.319	23.195	0.25		2	Medium	0.028	0.0196748
**ND_L75P**		45	51	KLLDNWD	902.4498	4.1873	9000	5	4.445	2.307	23.074	0.4		2	Medium	0.045	0.0681454
**ND_L75P**		45	56	KLLDNWDSVTST	1377.6776	4.2415	0	10	0	0	0	n/a		2	High	0	
**ND_L75P**		45	56	KLLDNWDSVTST	1377.6776	4.2241	30	10	0.173	4.017	20.083	0.471		2	Medium	0.052	0.00259823
**ND_L75P**		45	56	KLLDNWDSVTST	1377.6776	4.2341	300	10	1.499	4.917	24.584	0.561		2	Medium	0.062	0.0299922
**ND_L75P**		45	56	KLLDNWDSVTST	1377.6776	4.259	3000	10	6.315	5.665	28.324	0.003		2	Medium	0	0.347134
**ND_L75P**		45	56	KLLDNWDSVTST	1377.6776	4.1898	9000	10	8.564	5.758	28.789	0.786		2	Medium	0.087	0.217013
**ND_L75P**		47	56	LDNWDSVTST	1136.4986	3.9493	0	8	0	0	0	n/a		2	High	0	
**ND_L75P**		47	56	LDNWDSVTST	1136.4986	3.9566	30	8	0.154	3.798	47.479	0.175		2	High	0.02	0.00673753
**ND_L75P**		47	56	LDNWDSVTST	1136.4986	3.9556	300	8	1.317	4.458	55.725	0.529		2	High	0.059	0.26646
**ND_L75P**		47	56	LDNWDSVTST	1136.4986	3.955	3000	8	5.25	4.838	60.477	0.823		2	High	0.092	0.192581
**ND_L75P**		47	56	LDNWDSVTST	1136.4986	3.9385	9000	8	6.987	4.724	59.045	0.359		2	High	0.04	0.254577
**ND_L75P**		50	56	WDSVTST	794.3446	3.0657	0	5	0	0	0	n/a		2	Medium	0	
**ND_L75P**		50	56	WDSVTST	794.3446	3.0359	30	5	0.075	2.615	52.304	0.298		2	Medium	0.033	0.00584616
**ND_L75P**		50	56	WDSVTST	794.3446	3.061	300	5	0.659	2.891	57.812	0.231		2	Medium	0.026	0.325879
**ND_L75P**		50	56	WDSVTST	794.3446	3.0629	3000	5	2.92	3.071	61.418	0.204		2	Medium	0.023	0.0212049
**ND_L75P**		50	56	WDSVTST	794.3446	3.0558	9000	5	4.119	3.01	60.191	0.324		2	Medium	0.036	0.118114
**ND_L75P**		51	56	DSVTST	608.2653	1.5269	0	4	0	0	0	n/a		2	High	0	
**ND_L75P**		51	56	DSVTST	608.2653	1.4933	30	4	0.032	1.974	49.35	0.454		2	High	0.051	0.0166477
**ND_L75P**		51	56	DSVTST	608.2653	1.4775	300	4	0.305	2.333	58.313	0.077		2	High	0.009	0.205904
**ND_L75P**		51	56	DSVTST	608.2653	1.494	3000	4	1.933	2.547	63.687	0.423		2	High	0.047	0.194502
**ND_L75P**		51	56	DSVTST	608.2653	1.4934	9000	4	3.119	2.48	62	0.109		2	High	0.012	0.312536
**ND_L75P**		51	57	DSVTSTF	755.3337	3.6138	0	5	0	0	0	n/a		2	High	0	
**ND_L75P**		51	57	DSVTSTF	755.3337	3.599	30	5	0.037	2.364	47.283	0.616		2	Medium	0.069	0.0334953
**ND_L75P**		51	57	DSVTSTF	755.3337	3.5986	300	5	0.356	2.888	57.751	0.109		2	Medium	0.012	0.0960939
**ND_L75P**		51	57	DSVTSTF	755.3337	3.5981	3000	5	2.34	3.177	63.533	0.409		2	Medium	0.046	0.158856
**ND_L75P**		51	57	DSVTSTF	755.3337	3.5989	9000	5	3.91	3.104	62.076	0.258		2	Medium	0.029	0.316199
**ND_L75P**		57	70	FSKLREQLGPVTQE	1630.8679	3.1887	0	11	0	0	0	n/a		2	High	0	
**ND_L75P**		57	70	FSKLREQLGPVTQE	1630.8679	3.1771	30	11	0.085	4.723	21.47	1.17		2	High	0.13	0.0331237
**ND_L75P**		57	70	FSKLREQLGPVTQE	1630.8679	3.1652	300	11	0.814	6.139	27.904	0.29		2	High	0.032	0.00100877
**ND_L75P**		57	70	FSKLREQLGPVTQE	1630.8679	3.1607	3000	11	5.447	7.088	32.216	0.706		2	High	0.079	0.00192649
**ND_L75P**		57	70	FSKLREQLGPVTQE	1630.8679	3.1237	9000	11	8.858	7.055	32.07	0.506		2	High	0.056	0.00225009
**ND_L75P**		57	71	FSKLREQLGPVTQEF	1777.9363	4.1639	0	12	0	0	0	n/a		2	High	0	
**ND_L75P**		57	71	FSKLREQLGPVTQEF	1777.9363	4.1476	30	12	0.093	4.917	20.488	0.653		2	High	0.073	0.00190089
**ND_L75P**		57	71	FSKLREQLGPVTQEF	1777.9363	4.1406	300	12	0.892	6.552	27.298	0.805		2	High	0.09	0.00161218
**ND_L75P**		57	71	FSKLREQLGPVTQEF	1777.9363	4.1704	3000	12	6.004	7.543	31.43	0.453		2	High	0.05	0.0105202
**ND_L75P**		57	71	FSKLREQLGPVTQEF	1777.9363	4.1492	9000	12	9.771	7.511	31.296	1.209		2	High	0.135	0.010861
**ND_L75P**		58	70	SKLREQLGPVTQE	1483.7995	2.768	0	10	0	0	0	n/a		2	High	0	
**ND_L75P**		58	70	SKLREQLGPVTQE	1483.7995	2.7635	30	10	0.076	4.851	24.254	0.051		2	High	0.006	0.0123314
**ND_L75P**		58	70	SKLREQLGPVTQE	1483.7995	2.7574	300	10	0.725	5.731	28.655	0.124		2	High	0.014	0.0130214
**ND_L75P**		58	70	SKLREQLGPVTQE	1483.7995	2.7952	3000	10	4.841	6.247	31.236	0.537		2	High	0.06	0.0181992

**ND_L75P**		58	70	SKLREQLGPVTQE	1483.7995	2.7594	9000	10	7.919	6.291	31.456	0.188	2	High	0.021	0.0288641
**ND_L75P**		58	71	SKLREQLGPVTQEF	1630.8679	3.8804	0	11	0	0	0	n/a	2	Medium	0	
**ND_L75P**		58	71	SKLREQLGPVTQEF	1630.8679	3.8466	30	11	0.084	5.246	19.81	0.227	4	Medium	0.143	1.65E-05
**ND_L75P**		58	71	SKLREQLGPVTQEF	1630.8679	3.8429	300	11	0.803	6.287	23.889	0.203	4	Medium	0.128	1.16E-05
**ND_L75P**		58	71	SKLREQLGPVTQEF	1630.8679	3.8561	3000	11	5.397	7.111	26.95	0.291	4	Medium	0.183	0.000130636
**ND_L75P**		58	71	SKLREQLGPVTQEF	1630.8679	3.8151	9000	11	8.832	6.984	26.526	0.23	4	Medium	0.145	6.99E-05
**ND_L75P**		61	71	REQLGPVTQEF	1302.6568	3.9718	0	8	0	0	0	n/a	2	High	0	
**ND_L75P**		61	71	REQLGPVTQEF	1302.6568	3.9904	30	8	0.062	3.641	22.754	0.647	2	High	0.072	0.00420666
**ND_L75P**		61	71	REQLGPVTQEF	1302.6568	3.9855	300	8	0.589	4.38	27.375	0.62	2	High	0.069	0.00874452
**ND_L75P**		61	71	REQLGPVTQEF	1302.6568	3.9834	3000	8	3.963	4.891	30.571	0.748	2	High	0.083	0.031056
**ND_L75P**		61	71	REQLGPVTQEF	1302.6568	3.9645	9000	8	6.444	4.795	29.967	0.901	2	High	0.1	0.0173155
**ND_L75P**		72	91	WDNPEKETEGLRQEMSKDLE	2433.1118	4.6	0	17	0	0	0	n/a	2	High	0	
**ND_L75P**		72	91	WDNPEKETEGLRQEMSKDLE	2433.1118	4.5366	30	17	0.236	10.338	15.203	1.014	2	High	0.113	1.02E-06
**ND_L75P**		72	91	WDNPEKETEGLRQEMSKDLE	2433.1118	4.5659	300	17	2.136	10.109	14.866	0.852	2	High	0.095	0.000126874
**ND_L75P**		72	91	WDNPEKETEGLRQEMSKDLE	2433.1118	4.5555	3000	17	11.279	10.94	16.089	1.286	2	High	0.143	7.86E-05
**ND_L75P**		72	91	WDNPEKETEGLRQEMSKDLE	2433.1118	4.5642	9000	17	15.953	10.648	15.659	0.876	2	High	0.098	1.16E-05
**ND_L75P**		72	92	WDNPEKETEGLRQEMSKDLEE	2562.1544	4.7009	0	18	0	0	0	n/a	2	High	0	
**ND_L75P**		72	92	WDNPEKETEGLRQEMSKDLEE	2562.1544	4.6479	30	18	0.256	9.267	17.161	0.905	2	High	0.101	1.45E-06
**ND_L75P**		72	92	WDNPEKETEGLRQEMSKDLEE	2562.1544	4.6393	300	18	2.318	9.845	18.232	1.303	2	High	0.145	0.00259546
**ND_L75P**		72	92	WDNPEKETEGLRQEMSKDLEE	2562.1544	4.629	3000	18	12.146	11.46	21.223	0.889	2	High	0.099	4.71E-06
**ND_L75P**		72	92	WDNPEKETEGLRQEMSKDLEE	2562.1544	4.6031	9000	18	16.95	11.735	21.731	0.179	2	High	0.02	1.20E-05
**ND_L75P**		72	103	WDNPEKETEGLRQEMSKDLEEVKAKVQPYLI	3818.8309	5.6581	0	28	0	0	0	n/a	2	Medium	0	
**ND_L75P**		72	103	WDNPEKETEGLRQEMSKDLEEVKAKVQPYLI	3818.8309	5.5764	30	28	0.395	8.982	8.019	1.095	2	High	0.122	0.00510296
**ND_L75P**		72	103	WDNPEKETEGLRQEMSKDLEEVKAKVQPYLI	3818.8309	5.5837	300	28	3.441	10.46	9.339	0.095	2	High	0.011	0.00472838
**ND_L75P**		72	103	WDNPEKETEGLRQEMSKDLEEVKAKVQPYLI	3818.8309	5.5416	3000	28	16.975	13.38	11.946	0.211	2	High	0.023	0.00303321
**ND_L75P**		72	103	WDNPEKETEGLRQEMSKDLEEVKAKVQPYLI	3818.8309	5.5051	9000	28	24.697	15.132	13.511	1.539	2	High	0.171	0.00113683
**ND_L75P**		77	92	KETEGLRQEMSKDLEE	1920.9099	0	0	14	0	0	0	n/a	2	High	0	
**ND_L75P**		77	92	KETEGLRQEMSKDLEE	1920.9099	0	30	14	0.173							
**ND_L75P**		77	92	KETEGLRQEMSKDLEE	1920.9099	0	300	14	1.615							
**ND_L75P**		77	92	KETEGLRQEMSKDLEE	1920.9099	0	3000	14	9.321							
**ND_L75P**		77	92	KETEGLRQEMSKDLEE	1920.9099	0	9000	14	13.171							
**ND_L75P**		83	92	RQEMSKDLEE	1263.5765	2.2834	0	8	0	0	0	n/a	2	Medium	0	
**ND_L75P**		83	92	RQEMSKDLEE	1263.5765	2.246	30	8	0.114	3.733	23.332	0.27	2	Medium	0.03	0.00137605
**ND_L75P**		83	92	RQEMSKDLEE	1263.5765	2.2782	300	8	1.052	3.974	24.837	0.208	2	Medium	0.023	0.000270804
**ND_L75P**		83	92	RQEMSKDLEE	1263.5765	2.2797	3000	8	5.659	4.529	28.308	0.122	2	Medium	0.014	2.09E-05
**ND_L75P**		83	92	RQEMSKDLEE	1263.5765	2.273	9000	8	7.634	4.525	28.28	0.281	2	Medium	0.031	0.000174581
**ND_L75P**		87	92	SKDLEE	719.3337	1.6941	0	4	0	0	0	n/a	2	Medium	0	
**ND_L75P**		87	92	SKDLEE	719.3337	1.7147	30	4	0.064	1.101	27.536	0.593	2	Medium	0.066	0.0368238
**ND_L75P**		87	92	SKDLEE	719.3337	1.6868	300	4	0.579	1.523	38.087	0.51	2	Medium	0.057	0.0220221
**ND_L75P**		87	92	SKDLEE	719.3337	1.7007	3000	4	2.821	1.872	46.794	0.314	2	Medium	0.035	0.000698995
**ND_L75P**		87	92	SKDLEE	719.3337	1.6724	9000	4	3.749	1.859	46.473	0.49	2	Medium	0.055	0.011027
**ND_L75P**		90	103	LEEVKAKVQPYLDD	1645.8563	3.1913	0	11	0	0	0	n/a	2	Medium	0	
**ND_L75P**		90	103	LEEVKAKVQPYLDD	1645.8563	3.2542	30	11	0.159	2.463	11.197	0.726	2	High	0.081	0.0111629
**ND_L75P**		90	103	LEEVKAKVQPYLDD	1645.8563	3.2583	300	11	1.305	3.858	17.535	0.357	2	High	0.04	0.000214138
**ND_L75P**		90	103	LEEVKAKVQPYLDD	1645.8563	3.2537	3000	11	5.696	5.939	26.994	0.812	2	Medium	0.09	0.00454478
**ND_L75P**		90	103	LEEVKAKVQPYLDD	1645.8563	3.2437	9000	11	8.744	6.151	27.957	0.03	2	Medium	0.003	0.00680815
**ND_L75P**		91	103	EEVKAKVQPYLDD	1532.7722	2.9606	0	10	0	0	0	n/a	2	High	0	
**ND_L75P**		91	103	EEVKAKVQPYLDD	1532.7722	2.9883	30	10	0.139	2.284	11.422	0.361	2	High	0.04	0.00841288
**ND_L75P**		91	103	EEVKAKVQPYLDD	1532.7722	2.9779	300	10	1.123	3.361	16.806	0.69	2	High	0.077	0.000953384
**ND_L75P**		91	103	EEVKAKVQPYLDD	1532.7722	2.9624	3000	10	4.829	4.827	24.136	0.519	2	Medium	0.058	0.0011734
**ND_L75P**		91	103	EEVKAKVQPYLDD	1532.7722	2.9499	9000	10	7.747	5.327	26.635	0.721	2	Medium	0.08	0.000797778
**ND_L75P**		92	103	EVKAKVQPYLDD	1403.7296	2.8528	0	9	0	0	0	n/a	2	High	0	
**ND_L75P**		92	103	EVKAKVQPYLDD	1403.7296	2.8381	30	9	0.136	2.127	11.819	0.924	2	High	0.103	0.00530659
**ND_L75P**		92	103	EVKAKVQPYLDD	1403.7296	2.8095	300	9	1.091	3.443	19.13	0.288	2	High	0.032	0.0032635
**ND_L75P**		92	103	EVKAKVQPYLDD	1403.7296	2.8594	3000	9	4.55	4.758	26.432	1.108	2	High	0.123	0.0169189
**ND_L75P**		92	103	EVKAKVQPYLDD	1403.7296	2.8468	9000	9	7.122	4.993	27.737	1.129	2	High	0.126	0.00899037
**ND_L75P**		93	103	VKAKVQPYLDD	1274.6871	2.736	0	8	0	0	0	n/a	2	High	0	
**ND_L75P**		93	103	VKAKVQPYLDD	1274.6871	2.6787	30	8	0.131	1.919	11.993	0.488	2	High	0.054	0.00481693
**ND_L75P**		93	103	VKAKVQPYLDD	1274.6871	2.6939	300	8	1.047	2.916	18.225	0.211	2	High	0.023	0.00527554
**ND_L75P**		93	103	VKAKVQPYLDD	1274.6871	2.6929	3000	8	4.191	4.185	26.153	0.977	2	High	0.109	0.0190083
**ND_L75P**		93	103	VKAKVQPYLDD	1274.6871	2.6998	9000	8	6.384	4.348	27.172	0.474	2	High	0.053	0.000653829
**ND_L75P**		93	104	VKAKVQPYLDDF	1421.7555	4.3472	0	9	0	0	0	n/a	2	Medium	0	
**ND_L75P**		93	104	VKAKVQPYLDDF	1421.7555	4.3374	30	9	0.144	1.516	8.424	0.173	2	Medium	0.019	0.000345013
**ND_L75P**		93	104	VKAKVQPYLDDF	1421.7555	4.3384	300	9	1.168	2.651	14.726	0.255	2	Medium	0.028	0.00079812
**ND_L75P**		93	104	VKAKVQPYLDDF	1421.7555	4.3374	3000	9	4.915	4.241	23.561	0.918	2	Medium	0.102	0.00244176
**ND_L75P**		93	104	VKAKVQPYLDDF	1421.7555	4.3098	9000	9	7.363	4.372	24.289	0.693	2	Medium	0.077	0.00159342
**ND_L75P**		96	103	KVQPYLDD	976.4866	2.9485	0	5	0	0	0	n/a	2	Medium	0	
**ND_L75P**		96	103	KVQPYLDD	976.4866	2.9347	30	5	0.111	0.927	9.269	0.59	2	Medium	0.066	0.0277207
**ND_L75P**		96	103	KVQPYLDD	976.4866	2.9574	300	5	0.848	1.242	12.419	0.547	2	Medium	0.061	0.0197063
**ND_L75P**		96	103	KVQPYLDD	976.4866	2.9342	3000	5	2.803	1.849	18.494	0.092	2	Medium	0.01	0.00523372
**ND_L75P**		96	103	KVQPYLDD	976.4866	2.9054	9000	5	4.053	1.903	19.035	0.494	2	Medium	0.055	0.0162059

ND_L75P		104	111	FQKKWQEE	1121.5506	2.5295	0	6	0	0	0	n/a	2	High	0	
ND_L75P		104	111	FQKKWQEE	1121.5506	2.5064	30	6	0.058	0.416	6.933	0.768	2	High	0.086	0.180768
ND_L75P		104	111	FQKKWQEE	1121.5506	2.5331	300	6	0.55	0.927	15.446	0.244	2	High	0.027	0.00303068
ND_L75P		104	111	FQKKWQEE	1121.5506	2.5427	3000	6	3.439	2.313	38.555	0.176	2	High	0.02	0.00546588
ND_L75P		104	111	FQKKWQEE	1121.5506	2.5216	9000	6	5.306	2.788	46.464	0.47	2	High	0.052	0.00366821
ND_L75P		113	124	ELYRQKVEPLRA	1500.8413	3.1796	0	9	0	0	0	n/a	2	High	0	
ND_L75P		113	124	ELYRQKVEPLRA	1500.8413	3.1781	30	9	0.06	1.771	6.558	0.527	2	High	0.059	0.00553194
ND_L75P		113	124	ELYRQKVEPLRA	1500.8413	3.1653	300	9	0.574	2.839	10.514	0.32	2	High	0.036	0.00107481
ND_L75P		113	124	ELYRQKVEPLRA	1500.8413	3.1696	3000	9	4.134	4.564	16.905	1.057	2	High	0.118	0.0684387
ND_L75P		113	124	ELYRQKVEPLRA	1500.8413	3.1733	9000	9	7.172	5.042	18.674	0.685	2	High	0.076	0.0551819
ND_L75P		113	126	ELYRQKVEPLRAEL	1742.9679	3.5219	0	11	0	0	0	n/a	2	Medium	0	
ND_L75P		113	126	ELYRQKVEPLRAEL	1742.9679	3.5231	30	11	0.074	2.001	6.065	0.603	2	Medium	0.067	0.000571235
ND_L75P		113	126	ELYRQKVEPLRAEL	1742.9679	3.5251	300	11	0.718	3.103	9.403	0.586	2	Medium	0.065	0.00522674
ND_L75P		113	126	ELYRQKVEPLRAEL	1742.9679	3.5391	3000	11	5.145	5.147	15.598	0.273	2	Medium	0.03	0.00929133
ND_L75P		113	126	ELYRQKVEPLRAEL	1742.9679	3.5243	9000	11	8.871	5.98	18.123	1.837	2	Medium	0.204	0.121044
ND_L75P		114	124	LYRQKVEPLRA	1371.7987	2.3972	0	8	0	0	0	n/a	2	High	0	
ND_L75P		114	124	LYRQKVEPLRA	1371.7987	2.3792	30	8	0.055	1.838	7.659	0.138	2	Medium	0.015	0.000714415
ND_L75P		114	124	LYRQKVEPLRA	1371.7987	2.4492	300	8	0.529	2.521	10.504	0.536	2	Medium	0.06	0.00110929
ND_L75P		114	124	LYRQKVEPLRA	1371.7987	2.4678	3000	8	3.763	4.049	16.869	0.064	2	Medium	0.007	0.00411522
ND_L75P		114	124	LYRQKVEPLRA	1371.7987	2.4518	9000	8	6.42	4.727	19.697	0.99	2	Medium	0.11	0.287865
ND_L75P		114	125	LYRQKVEPLRAE	1500.8413	2.9837	0	9	0	0	0	n/a	2	Medium	0	
ND_L75P		114	125	LYRQKVEPLRAE	1500.8413	2.987	30	9	0.065	1.774	6.569	0.189	2	Medium	0.021	4.41E-05
ND_L75P		114	125	LYRQKVEPLRAE	1500.8413	2.9792	300	9	0.628	2.852	10.564	0.536	2	Medium	0.06	0.000686706
ND_L75P		114	125	LYRQKVEPLRAE	1500.8413	2.9734	3000	9	4.41	4.642	17.192	0.762	2	Medium	0.085	0.0484779
ND_L75P		114	125	LYRQKVEPLRAE	1500.8413	2.9404	9000	9	7.376	5.186	19.206	0.539	2	Medium	0.06	0.0886462
ND_L75P		114	126	LYRQKVEPLRAEL	1613.9253	3.3476	0	10	0	0	0	n/a	2	High	0	
ND_L75P		114	126	LYRQKVEPLRAEL	1613.9253	3.3454	30	10	0.07	2.084	8.637	0.146	4	High	0.092	1.52E-08
ND_L75P		114	126	LYRQKVEPLRAEL	1613.9253	3.3427	300	10	0.672	3.196	13.267	0.251	4	High	0.158	1.26E-05
ND_L75P		114	126	LYRQKVEPLRAEL	1613.9253	3.3483	3000	10	4.773	5.188	21.588	0.308	4	High	0.194	0.000669675
ND_L75P		114	126	LYRQKVEPLRAEL	1613.9253	3.347	9000	10	8.119	5.887	24.525	0.111	4	High	0.07	0.0879793
ND_L75P		115	124	YRQKVEPLRA	1258.7146	2.0982	0	7	0	0	0	n/a	2	High	0	
ND_L75P		115	124	YRQKVEPLRA	1258.7146	2.096	30	7	0.048	1.578	7.514	0.664	2	High	0.074	0.0145425
ND_L75P		115	124	YRQKVEPLRA	1258.7146	2.0942	300	7	0.46	2.317	11.031	0.489	2	High	0.054	0.00133796
ND_L75P		115	124	YRQKVEPLRA	1258.7146	2.092	3000	7	3.251	3.658	17.418	0.561	2	High	0.062	0.046661
ND_L75P		115	124	YRQKVEPLRA	1258.7146	2.1114	9000	7	5.537	4.029	19.184	0.328	2	High	0.036	0.0672244
ND_L75P		115	126	YRQKVEPLRAEL	1500.8413	3.1471	0	9	0	0	0	n/a	2	High	0	
ND_L75P		115	126	YRQKVEPLRAEL	1500.8413	3.2168	30	9	0.063	1.642	5.383	0.254	4	High	0.159	8.37E-06
ND_L75P		115	126	YRQKVEPLRAEL	1500.8413	3.1822	300	9	0.603	2.75	8.926	0.231	4	High	0.145	8.78E-06
ND_L75P		115	126	YRQKVEPLRAEL	1500.8413	3.1745	3000	9	4.262	4.477	14.535	0.258	4	High	0.162	0.0012601
ND_L75P		115	126	YRQKVEPLRAEL	1500.8413	3.1839	9000	9	7.235	4.958	16.105	0.17	4	High	0.107	0.0021764
ND_L75P		115	147	YRQKVEPLRAELQEGARQKLHELQEKLSPLG	3901.081	4.5586	0	29	0	0	0	n/a	2	High	0	
ND_L75P		115	147	YRQKVEPLRAELQEGARQKLHELQEKLSPLG	3901.081	4.582	30	29	0.313	3.778	2.606	0.201	2	High	0.022	0.00888886
ND_L75P		115	147	YRQKVEPLRAELQEGARQKLHELQEKLSPLG	3901.081	4.5083	300	29	2.883	8.534	5.886	2.264	2	High	0.252	0.0014591
ND_L75P		115	147	YRQKVEPLRAELQEGARQKLHELQEKLSPLG	3901.081	4.4799	3000	29	16.5	14.566	10.046	1.442	2	High	0.16	0.0135953
ND_L75P		115	147	YRQKVEPLRAELQEGARQKLHELQEKLSPLG	3901.081	4.5081	9000	29	24.896	16.202	11.174	1.697	2	High	0.189	0.31125
ND_L75P		115	158	YRQKVEPLRAELQEGARQKLHELQEKLSPLG	5179.7174	4.5539	0	40	0	0	0	n/a	2	High	0	
ND_L75P		115	158	YRQKVEPLRAELQEGARQKLHELQEKLSPLG	5179.7174	4.5794	30	40	0.506	4.653	1.662	1.845	2	High	0.205	0.00671973
ND_L75P		115	158	YRQKVEPLRAELQEGARQKLHELQEKLSPLG	5179.7174	4.4824	300	40	4.628	12.228	4.367	0.174	2	High	0.019	0.00622324
ND_L75P		115	158	YRQKVEPLRAELQEGARQKLHELQEKLSPLG	5179.7174	4.5001	3000	40	24.825	17.538	6.264	0.27	2	High	0.03	0.000817452
ND_L75P		115	158	YRQKVEPLRAELQEGARQKLHELQEKLSPLG	5179.7174	4.4822	9000	40	35.51	21.827	7.795	0.79	2	High	0.088	0.00681846
ND_L75P		125	136	ELQEGARQKLHE	1436.7372	1.9361	0	10	0	0	0	n/a	2	Medium	0	
ND_L75P		125	136	ELQEGARQKLHE	1436.7372	1.9264	30	10	0.157	2.333	11.667	0.025	2	Medium	0.003	0.000487237
ND_L75P		125	136	ELQEGARQKLHE	1436.7372	1.9318	300	10	1.395	3.403	17.017	0.171	2	Medium	0.019	0.00396682
ND_L75P		125	136	ELQEGARQKLHE	1436.7372	1.935	3000	10	6.71	4.914	24.569	0.379	2	Medium	0.042	0.013103
ND_L75P		125	136	ELQEGARQKLHE	1436.7372	1.919	9000	10	9.173	5.188	25.938	0.433	2	Medium	0.048	0.0605141
ND_L75P		125	139	ELQEGARQKLHELQE	1806.9224	2.9681	0	13	0	0	0	n/a	2	High	0	
ND_L75P		125	139	ELQEGARQKLHELQE	1806.9224	2.9629	30	13	0.181	2.729	10.497	0.432	2	High	0.048	0.00132896
ND_L75P		125	139	ELQEGARQKLHELQE	1806.9224	2.9604	300	13	1.624	4.096	15.755	0.623	2	High	0.069	0.00139061
ND_L75P		125	139	ELQEGARQKLHELQE	1806.9224	2.9501	3000	13	8.264	6.114	23.515	0.001	2	Medium	0	0.0186862
ND_L75P		125	139	ELQEGARQKLHELQE	1806.9224	2.938	9000	13	11.729	6.828	26.26	0.288	2	Medium	0.032	0.0180882
ND_L75P		125	146	ELQEGARQKLHELQEKLSPLGE	2531.3344	4.3472	0	19	0	0	0	n/a	2	High	0	
ND_L75P		125	146	ELQEGARQKLHELQEKLSPLGE	2531.3344	4.3627	30	19	0.23	3.683	6.461	1.11	2	Medium	0.123	2.80E-05
ND_L75P		125	146	ELQEGARQKLHELQEKLSPLGE	2531.3344	4.3046	300	19	2.098	5.94	10.42	0.81	2	Medium	0.09	5.97E-06
ND_L75P		125	146	ELQEGARQKLHELQEKLSPLGE	2531.3344	4.3078	3000	19	11.371	8.247	14.468	1.465	2	Medium	0.163	0.00343967
ND_L75P		125	146	ELQEGARQKLHELQEKLSPLGE	2531.3344	4.32	9000	19	16.663	8.693	15.25	1.785	2	Medium	0.199	0.00294515
ND_L75P		125	148	ELQEGARQKLHELQEKLSPLGEEM	2791.4174	4.5701	0	21	0	0	0	n/a	2	High	0	
ND_L75P		125	148	ELQEGARQKLHELQEKLSPLGEEM	2791.4174	4.5627	30	21	0.262	4.199	5.802	0.733	4	High	0.461	2.39E-07

**ND_L75P**		125	148	ELQEGARQKLHELQEKLSPLGEEM	2791.4174	4.5404	300	21	2.393	6.732	9.298	0.775	4	Medium	0.487	1.07E-05
**ND_L75P**		125	148	ELQEGARQKLHELQEKLSPLGEEM	2791.4174	4.5379	3000	21	12.937	10.467	14.573	0.561	4	High	0.353	0.00637223
**ND_L75P**		125	148	ELQEGARQKLHELQEKLSPLGEEM	2791.4174	4.5382	9000	21	18.634	11.433	15.907	0.326	4	High	0.205	0.708633
**ND_L75P**		125	158	ELQEGARQKLHELQEKLSPLGEEMRDRARAH	3939.0133	4.3172	0	31	0	0	0	n/a	2	High	0	
**ND_L75P**		125	158	ELQEGARQKLHELQEKLSPLGEEMRDRARAH	3939.0133	4.2757	30	31	0.443	5.554	4.039	0.447	4	High	0.281	4.05E-08
**ND_L75P**		125	158	ELQEGARQKLHELQEKLSPLGEEMRDRARAH	3939.0133	4.2749	300	31	4.025	8.658	6.324	1.316	4	High	0.827	0.0017859
**ND_L75P**		125	158	ELQEGARQKLHELQEKLSPLGEEMRDRARAH	3939.0133	4.2492	3000	31	20.563	14.759	10.685	1.609	4	High	1.011	0.186426
**ND_L75P**		125	158	ELQEGARQKLHELQEKLSPLGEEMRDRARAH	3939.0133	4.2594	9000	31	28.274	16.301	11.827	0.449	4	High	0.282	0.000119931
**ND_L75P**		125	159	ELQEGARQKLHELQEKLSPLGEEMRDRARAH	4052.0974	4.5079	0	32	0	0	0	n/a	2	High	0	
**ND_L75P**		125	159	ELQEGARQKLHELQEKLSPLGEEMRDRARAH	4052.0974	4.478	30	32	0.447	6.54	4.087	1.593	2	High	0.177	0.000749317
**ND_L75P**		125	159	ELQEGARQKLHELQEKLSPLGEEMRDRARAH	4052.0974	4.454	300	32	4.066	10.309	6.443	1.72	2	High	0.191	0.007918
**ND_L75P**		125	159	ELQEGARQKLHELQEKLSPLGEEMRDRARAH	4052.0974	4.4566	3000	32	20.902	15.857	9.91	1.575	2	High	0.175	0.0396914
**ND_L75P**		125	159	ELQEGARQKLHELQEKLSPLGEEMRDRARAH	4052.0974	4.4755	9000	32	28.986	16.795	10.497	0.254	2	High	0.028	0.0329896
**ND_L75P**		126	158	LQEGARQKLHELQEKLSPLGEEMRDRARAHV	3809.9707	4.3518	0	30	0	0	0	n/a	2	High	0	
**ND_L75P**		126	158	LQEGARQKLHELQEKLSPLGEEMRDRARAHV	3809.9707	4.3232	30	30	0.437	5.688	3.792	1.155	2	High	0.129	0.00383044
**ND_L75P**		126	158	LQEGARQKLHELQEKLSPLGEEMRDRARAHV	3809.9707	4.3215	300	30	3.968	8.822	5.881	1.668	2	High	0.186	0.00503689
**ND_L75P**		126	158	LQEGARQKLHELQEKLSPLGEEMRDRARAHV	3809.9707	4.3156	3000	30	20.117	13.926	9.284	0.414	2	High	0.046	0.00292117
**ND_L75P**		126	158	LQEGARQKLHELQEKLSPLGEEMRDRARAHV	3809.9707	4.3021	9000	30	27.444	15.891	10.594	1.647	2	High	0.183	0.00602692
**ND_L75P**		127	139	QEGARQKLHELQE	1564.7958	2.4201	0	11	0	0	0	n/a	2	High	0	
**ND_L75P**		127	139	QEGARQKLHELQE	1564.7958	2.4089	30	11	0.161	2.206	10.025	0.494	2	High	0.055	0.0015222
**ND_L75P**		127	139	QEGARQKLHELQE	1564.7958	2.421	300	11	1.44	3.394	15.429	0.434	2	High	0.048	0.000637247
**ND_L75P**		127	139	QEGARQKLHELQE	1564.7958	2.3836	3000	11	7.073	5.482	24.918	0.274	2	High	0.03	0.0774093
**ND_L75P**		127	139	QEGARQKLHELQE	1564.7958	2.3871	9000	11	9.916	5.941	27.006	0.345	2	High	0.038	0.0665386
**ND_L75P**		127	146	QEGARQKLHELQEKLSPLGE	2289.2077	4.1637	0	17	0	0	0	n/a	2	Medium	0	
**ND_L75P**		127	146	QEGARQKLHELQEKLSPLGE	2289.2077	4.0712	30	17	0.211	3.492	6.846	0.284	2	Medium	0.032	2.90E-05
**ND_L75P**		127	146	QEGARQKLHELQEKLSPLGE	2289.2077	4.0602	300	17	1.913	5.218	10.231	0.263	2	Medium	0.029	0.000623971
**ND_L75P**		127	146	QEGARQKLHELQEKLSPLGE	2289.2077	4.0097	3000	17	10.181	8.647	16.955	0.639	2	Medium	0.071	0.0161476
**ND_L75P**		127	146	QEGARQKLHELQEKLSPLGE	2289.2077	3.9909	9000	17	14.85	9.547	18.719	0.183	2	Medium	0.02	0.0113795
**ND_L75P**		127	147	QEGARQKLHELQEKLSPLGEE	2418.2503	4.0705	0	18	0	0	0	n/a	2	High	0	
**ND_L75P**		127	147	QEGARQKLHELQEKLSPLGEE	2418.2503	4.0475	30	18	0.231	3.394	6.903	0.466	4	High	0.293	0.000809703
**ND_L75P**		127	147	QEGARQKLHELQEKLSPLGEE	2418.2503	4.0378	300	18	2.095	5.717	11.889	0.279	4	High	0.175	3.89E-05
**ND_L75P**		127	147	QEGARQKLHELQEKLSPLGEE	2418.2503	4.0179	3000	18	11.048	8.753	18.393	0.533	4	High	0.335	0.00427043
**ND_L75P**		127	147	QEGARQKLHELQEKLSPLGEE	2418.2503	4.0123	9000	18	15.847	9.501	19.863	0.259	4	High	0.163	0.243303
**ND_L75P**		127	154	QEGARQKLHELQEKLSPLGEEMRDRARA	3274.6953	4.1826	0	25	0	0	0	n/a	2	Medium	0	
**ND_L75P**		127	154	QEGARQKLHELQEKLSPLGEEMRDRARA	3274.6953	4.0954	30	25	0.344	3.571	3.571	0.684	2	High	0.076	0.000391117
**ND_L75P**		127	154	QEGARQKLHELQEKLSPLGEEMRDRARA	3274.6953	4.0898	300	25	3.121	5.864	5.864	0.672	2	High	0.075	0.00107112
**ND_L75P**		127	154	QEGARQKLHELQEKLSPLGEEMRDRARA	3274.6953	4.0675	3000	25	16.178	11.375	11.375	0.145	2	Medium	0.016	0.00172274
**ND_L75P**		127	154	QEGARQKLHELQEKLSPLGEEMRDRARA	3274.6953	4.0538	9000	25	22.604	13.538	13.538	1.77	2	Medium	0.197	0.118391
**ND_L75P**		127	158	QEGARQKLHELQEKLSPLGEEMRDRARAHV	3696.8867	4.1828	0	29	0	0	0	n/a	2	High	0	
**ND_L75P**		127	158	QEGARQKLHELQEKLSPLGEEMRDRARAHV	3696.8867	4.0901	30	29	0.424	4.976	3.432	1.105	2	High	0.123	0.000365499
**ND_L75P**		127	158	QEGARQKLHELQEKLSPLGEEMRDRARAHV	3696.8867	4.087	300	29	3.84	8.41	5.8	1.122	2	High	0.125	0.00190576
**ND_L75P**		127	158	QEGARQKLHELQEKLSPLGEEMRDRARAHV	3696.8867	4.0648	3000	29	19.373	13.163	9.078	0.367	2	High	0.041	0.00594479
**ND_L75P**		127	158	QEGARQKLHELQEKLSPLGEEMRDRARAHV	3696.8867	4.0536	9000	29	26.461	15.068	10.392	0.851	2	High	0.095	0.00410108
**ND_L75P**		127	159	QEGARQKLHELQEKLSPLGEEMRDRARAHV	3809.9707	4.3655	0	30	0	0	0	n/a	2	High	0	
**ND_L75P**		127	159	QEGARQKLHELQEKLSPLGEEMRDRARAHV	3809.9707	4.3565	30	30	0.428	5.077	3.753	1.493	4	High	0.938	8.47E-05
**ND_L75P**		127	159	QEGARQKLHELQEKLSPLGEEMRDRARAHV	3809.9707	4.3368	300	30	3.881	8.063	6.074	1.555	4	Medium	0.977	0.00519572
**ND_L75P**		127	159	QEGARQKLHELQEKLSPLGEEMRDRARAHV	3809.9707	4.3155	3000	30	19.712	14.077	10.593	1.749	4	High	1.099	0.112348
**ND_L75P**		127	159	QEGARQKLHELQEKLSPLGEEMRDRARAHV	3809.9707	4.3239	9000	30	27.172	15.634	11.735	0.481	4	High	0.302	0.000215383
**ND_L75P**		137	146	LQEKLSPLGE	1112.6077	3.4152	0	7	0	0	0	n/a	2	High	0	
**ND_L75P**		137	146	LQEKLSPLGE	1112.6077	3.4079	30	7	0.063	1.372	9.799	0.408	2	High	0.045	0.00876045
**ND_L75P**		137	146	LQEKLSPLGE	1112.6077	3.3842	300	7	0.601	2.291	16.363	0.75	2	Medium	0.083	0.00980238
**ND_L75P**		137	146	LQEKLSPLGE	1112.6077	3.4131	3000	7	3.852	3.462	24.727	0.399	2	Medium	0.044	0.107204
**ND_L75P**		137	146	LQEKLSPLGE	1112.6077	3.4097	9000	7	5.917	3.579	25.562	0.422	2	Medium	0.047	0.461324
**ND_L75P**		137	147	LQEKLSPLGEE	1241.6503	3.4474	0	8	0	0	0	n/a	2	Medium	0	
**ND_L75P**		137	147	LQEKLSPLGEE	1241.6503	3.4526	30	8	0.083	1.496	9.351	0.524	2	Medium	0.058	0.00962695
**ND_L75P**		137	147	LQEKLSPLGEE	1241.6503	3.4451	300	8	0.783	2.498	15.613	0.336	2	Medium	0.037	0.00258409
**ND_L75P**		137	147	LQEKLSPLGEE	1241.6503	3.4527	3000	8	4.718	3.863	24.146	0.4	2	Medium	0.045	0.156478
**ND_L75P**		137	147	LQEKLSPLGEE	1241.6503	3.4473	9000	8	6.915	4.024	25.149	0.226	2	Medium	0.025	0.097562
**ND_L75P**		137	158	LQEKLSPLGEEMRDRARAHVDA	2520.2867	3.3362	0	19	0	0	0	n/a	2	High	0	
**ND_L75P**		137	158	LQEKLSPLGEEMRDRARAHVDA	2520.2867	3.3127	30	19	0.276	2.947	4.128	0.135	4	High	0.085	7.93E-09
**ND_L75P**		137	158	LQEKLSPLGEEMRDRARAHVDA	2520.2867	3.3075	300	19	2.528	5.119	7.152	0.647	4	High	0.406	0.0108505
**ND_L75P**		137	158	LQEKLSPLGEEMRDRARAHVDA	2520.2867	3.2998	3000	19	13.043	8.53	11.983	0.492	4	High	0.309	0.00319081
**ND_L75P**		137	158	LQEKLSPLGEEMRDRARAHVDA	2520.2867	3.3019	9000	19	17.528	9.493	13.338	0.344	4	High	0.216	0.000203998
**ND_L75P**		137	159	LQEKLSPLGEEMRDRARAHVDAL	2633.3708	3.8528	0	20	0	0	0	n/a	2	High	0	
**ND_L75P**		137	159	LQEKLSPLGEEMRDRARAHVDAL	2633.3708	3.7801	30	20	0.28	3.273	3.273	1.192	2	High	0.133	0.00277405
**ND_L75P**		137	159	LQEKLSPLGEEMRDRARAHVDAL	2633.3708	3.8168	300	20	2.569	5.057	5.057	0.225	2	High	0.025	0.03104
**ND_L75P**		137	159	LQEKLSPLGEEMRDRARAHVDAL	2633.3708	3.789	3000	20	13.382	9.248	9.248	0.256	2	Medium	0.029	0.015958
**ND_L75P**		137	159	LQEKLSPLGEEMRDRARAHVDAL	2633.3708	3.7792	9000	20	18.24	10.616	10.616	0.026	2	Medium	0.003	0.00266032
**ND_L75P**		139	158	EKLSPLGEEMRDRARAHVDA	2279.1441	3.1796	0	17	0	0	0	n/a	2	High	0	
**ND_L75P**		139	158	EKLSPLGEEMRDRARAHVDA	2279.1441	3.1758	30	17	0.251	3.438	5.056	0.736	2	Medium	0.082	0.00482867

**ND_L75P**		139	158	EKLSPLGEEMRDRARAHVDA	2279.1441	3.173	300	17	2.292	5.439	7.998	0.747		2	Medium	0.083	0.807641
**ND_L75P**		139	158	EKLSPLGEEMRDRARAHVDA	2279.1441	3.1345	3000	17	11.614	8.784	12.917	0.864		2	Medium	0.096	0.0110862
**ND_L75P**		139	158	EKLSPLGEEMRDRARAHVDA	2279.1441	3.1485	9000	17	15.576	9.348	13.748	0.617		2	Medium	0.069	0.00127572
**ND_L75P**		140	146	KLSPLGE	742.4225	2.8988	0	4	0	0	0	n/a		2	Medium	0	
**ND_L75P**		140	146	KLSPLGE	742.4225	2.8988	30	4	0.035	0.875	21.869	0.017		2	Medium	0.002	0.0166011
**ND_L75P**		140	146	KLSPLGE	742.4225	2.9076	300	4	0.334	1.253	31.321	0.254		2	Medium	0.028	0.00613246
**ND_L75P**		140	146	KLSPLGE	742.4225	2.9068	3000	4	2.158	1.845	46.128	0.088		2	Medium	0.01	0.748559
**ND_L75P**		140	146	KLSPLGE	742.4225	2.8893	9000	4	3.363	1.843	46.074	0.493		2	Medium	0.055	0.787502
**ND_L75P**		140	147	KLSPLGEE	871.4651	2.9837	0	5	0	0	0	n/a		2	High	0	
**ND_L75P**		140	147	KLSPLGEE	871.4651	2.9909	30	5	0.055	1.04	10.405	0.005		2	High	0.001	0.0202541
**ND_L75P**		140	147	KLSPLGEE	871.4651	2.9993	300	5	0.516	1.612	16.116	0.165		2	Medium	0.018	0.0013107
**ND_L75P**		140	147	KLSPLGEE	871.4651	2.9819	3000	5	3.025	2.329	23.291	0.022		2	Medium	0.002	0.318069
**ND_L75P**		140	147	KLSPLGEE	871.4651	2.979	9000	5	4.361	2.343	23.427	0.114		2	Medium	0.013	0.272676
**ND_L75P**		140	148	KLSPLGEEM	1002.5056	3.7793	0	6	0	0	0	n/a		2	Medium	0	
**ND_L75P**		140	148	KLSPLGEEM	1002.5056	3.7269	30	6	0.067	1.01	8.419	0.155		2	Medium	0.017	0.00410512
**ND_L75P**		140	148	KLSPLGEEM	1002.5056	3.7291	300	6	0.629	1.64	13.669	0.276		2	Medium	0.031	0.00846654
**ND_L75P**		140	148	KLSPLGEEM	1002.5056	3.7354	3000	6	3.723	2.639	21.993	0.158		2	Medium	0.018	0.230909
**ND_L75P**		140	148	KLSPLGEEM	1002.5056	3.7285	9000	6	5.333	2.69	22.417	0.676		2	Medium	0.075	0.25227
**ND_L75P**		147	158	EMRDRARAHVDA	1425.6895	1.4451	0	10	0	0	0	n/a		2	Medium	0	
**ND_L75P**		147	158	EMRDRARAHVDA	1425.6895	1.392	30	10	0.181	1.567	3.917	0.245		2	Medium	0.027	0.00248658
**ND_L75P**		147	158	EMRDRARAHVDA	1425.6895	1.4347	300	10	1.632	2.361	5.902	0.954		2	Medium	0.106	0.0317153
**ND_L75P**		147	158	EMRDRARAHVDA	1425.6895	1.4302	3000	10	7.626	4.102	10.254	0.945		2	Medium	0.105	0.0122466
**ND_L75P**		147	158	EMRDRARAHVDA	1425.6895	1.455	9000	10	9.641	4.708	11.769	0.037		2	Medium	0.004	0.0107463
**ND_L75P**		147	159	EMRDRARAHVDAL	1538.7736	2.3119	0	11	0	0	0	n/a		2	Medium	0	
**ND_L75P**		147	159	EMRDRARAHVDAL	1538.7736	2.2967	30	11	0.185	1.286	3.351	0.654		4	Medium	0.411	0.176493
**ND_L75P**		147	159	EMRDRARAHVDAL	1538.7736	2.3273	300	11	1.672	1.953	5.205	0.815		4	Medium	0.512	0.0364408
**ND_L75P**		147	159	EMRDRARAHVDAL	1538.7736	2.3074	3000	11	7.965	3.93	10.461	0.235		4	Medium	0.148	1.12E-06
**ND_L75P**		147	159	EMRDRARAHVDAL	1538.7736	2.3177	9000	11	10.352	4.517	12.007	0.312		4	Medium	0.196	1.30E-05
**ND_L75P**		147	169	EMRDRARAHVDALRTHLAPYSDE	2708.3201	3.0979	0	20	0	0	0	n/a		2	High	0	
**ND_L75P**		147	169	EMRDRARAHVDALRTHLAPYSDE	2708.3201	3.1052	30	20	0.353	3.853	4.817	0.381		2	High	0.042	0.000939893
**ND_L75P**		147	169	EMRDRARAHVDALRTHLAPYSDE	2708.3201	3.0868	300	20	3.141	6.482	8.103	0.694		2	Medium	0.077	0.000482794
**ND_L75P**		147	169	EMRDRARAHVDALRTHLAPYSDE	2708.3201	3.0915	3000	20	14.157	8.394	10.493	0.629		2	Medium	0.07	0.00175783
**ND_L75P**		147	169	EMRDRARAHVDALRTHLAPYSDE	2708.3201	3.0652	9000	20	18.62	9.794	12.243	0.257		2	High	0.029	0.000111352
**ND_L75P**		148	158	MRDRARAHVDA	1296.6469	1.4265	0	9	0	0	0	n/a		2	High	0	
**ND_L75P**		148	158	MRDRARAHVDA	1296.6469	1.4303	30	9	0.173	1.095	3.041	0.071		2	High	0.008	0.0125519
**ND_L75P**		148	158	MRDRARAHVDA	1296.6469	1.4262	300	9	1.552	1.782	4.95	0.441		2	High	0.049	0.00623349
**ND_L75P**		148	158	MRDRARAHVDA	1296.6469	1.432	3000	9	7.061	3.153	8.759	0.258		2	High	0.029	0.00419536
**ND_L75P**		148	158	MRDRARAHVDA	1296.6469	1.439	9000	9	8.723	3.65	10.138	0.338		2	High	0.038	0.000717364
**ND_L75P**		148	159	MRDRARAHVDAL	1409.731	2.0301	0	10	0	0	0	n/a		2	Medium	0	
**ND_L75P**		148	159	MRDRARAHVDAL	1409.731	2.0279	30	10	0.177	1.119	2.797	0.642		2	High	0.071	0.0400632
**ND_L75P**		148	159	MRDRARAHVDAL	1409.731	2.0208	300	10	1.593	1.791	4.477	0.447		2	High	0.05	0.0177061
**ND_L75P**		148	159	MRDRARAHVDAL	1409.731	2.0168	3000	10	7.4	3.308	8.27	0.738		2	Medium	0.082	0.00522282
**ND_L75P**		148	159	MRDRARAHVDAL	1409.731	2.0309	9000	10	9.434	3.889	9.723	0.39		2	High	0.043	0.00076528
**ND_L75P**		148	169	MRDRARAHVDALRTHLAPYSDE	2579.2775	2.874	0	19	0	0	0	n/a		2	High	0	
**ND_L75P**		148	169	MRDRARAHVDALRTHLAPYSDE	2579.2775	2.8616	30	19	0.345	3.614	4.755	0.917		2	High	0.102	0.000185115
**ND_L75P**		148	169	MRDRARAHVDALRTHLAPYSDE	2579.2775	2.8836	300	19	3.061	5.427	7.141	0.679		2	Medium	0.076	4.76E-06
**ND_L75P**		148	169	MRDRARAHVDALRTHLAPYSDE	2579.2775	2.8514	3000	19	13.592	7.652	10.068	1.355		2	Medium	0.151	1.34E-05
**ND_L75P**		148	169	MRDRARAHVDALRTHLAPYSDE	2579.2775	2.8664	9000	19	17.702	8.136	10.705	1.647		2	High	0.183	0.00112051
**ND_L75P**		149	158	RDRARAHVDA	1165.6065	1.4265	0	8	0	0	0	n/a		2	Medium	0	
**ND_L75P**		149	158	RDRARAHVDA	1165.6065	1.4205	30	8	0.136	1.032	4.3	0.386		2	Medium	0.043	0.117928
**ND_L75P**		149	158	RDRARAHVDA	1165.6065	1.4096	300	8	1.24	1.633	6.804	0.577		2	Medium	0.064	0.0340391
**ND_L75P**		149	158	RDRARAHVDA	1165.6065	1.4235	3000	8	6.085	2.878	11.993	0.58		2	Medium	0.065	0.0121461
**ND_L75P**		149	158	RDRARAHVDA	1165.6065	1.4112	9000	8	7.723	3.395	14.146	0.609		2	Medium	0.068	0.0114887
**ND_L75P**		155	169	HVDALRTHLAPYSDE	1722.8325	3.0518	0	12	0	0	0	n/a		2	High	0	
**ND_L75P**		155	169	HVDALRTHLAPYSDE	1722.8325	3.0879	30	12	0.216	3.106	8.629	0.512		2	Medium	0.057	0.000709274
**ND_L75P**		155	169	HVDALRTHLAPYSDE	1722.8325	3.0572	300	12	1.907	4.177	11.603	0.673		2	Medium	0.075	0.00511677
**ND_L75P**		155	169	HVDALRTHLAPYSDE	1722.8325	3.0239	3000	12	8.293	5.339	14.831	0.813		2	Medium	0.09	0.010833
**ND_L75P**		155	169	HVDALRTHLAPYSDE	1722.8325	3.0435	9000	12	10.974	5.292	14.699	0.547		2	Medium	0.061	0.00965203
**ND_L75P**		155	170	HVDALRTHLAPYSDEL	1835.9166	3.7747	0	13	0	0	0	n/a		2	Medium	0	
**ND_L75P**		155	170	HVDALRTHLAPYSDEL	1835.9166	3.7982	30	13	0.221	3.257	8.35	0.375		2	Medium	0.042	0.00558642
**ND_L75P**		155	170	HVDALRTHLAPYSDEL	1835.9166	3.7996	300	13	1.951	4.431	11.361	0.865		2	Medium	0.096	0.00502992
**ND_L75P**		155	170	HVDALRTHLAPYSDEL	1835.9166	3.7784	3000	13	8.657	5.861	15.029	0.548		2	Medium	0.061	0.00140289
**ND_L75P**		155	170	HVDALRTHLAPYSDEL	1835.9166	3.7999	9000	13	11.716	5.824	14.932	0.731		2	Medium	0.081	0.00848605
**ND_L75P**		159	168	LRTHLAPYSD	1171.5986	2.6535	0	7	0	0	0	n/a		2	Medium	0	
**ND_L75P**		159	168	LRTHLAPYSD	1171.5986	2.6407	30	7	0.131	2.292	16.372	0.055		2	Medium	0.006	0.00130795
**ND_L75P**		159	168	LRTHLAPYSD	1171.5986	2.6504	300	7	1.143	2.818	20.131	0.17		2	Medium	0.019	0.00178344
**ND_L75P**		159	168	LRTHLAPYSD	1171.5986	2.6419	3000	7	4.818	3.389	24.21	0.244		2	Medium	0.027	0.00246983
**ND_L75P**		159	168	LRTHLAPYSD	1171.5986	2.6598	9000	7	6.467	3.106	22.182	0.244		2	Medium	0.027	0.00472873
**ND_L75P**		159	169	LRTHLAPYSDE	1300.6412	2.8532	0	8	0	0	0	n/a		2	High	0	
**ND_L75P**		159	169	LRTHLAPYSDE	1300.6412	2.7135	30	8	0.163	2.4	15.002	0.339		2	High	0.038	0.000981557
**ND_L75P**		159	169	LRTHLAPYSDE	1300.6412	2.7214	300	8	1.416	3.026	18.91	0.251		2	High	0.028	0.00223172

ND_L75P		159	169	LRTHLAPYSDE	1300.6412	2.7241	3000	8	5.777	3.42	21.374	0.704	2	High	0.078	0.0164297
ND_L75P		159	169	LRTHLAPYSDE	1300.6412	2.7245	9000	8	7.467	3.342	20.889	0.113	2	High	0.013	0.0013489
ND_L75P		159	170	LRTHLAPYSDEL	1413.7252	3.7194	0	9	0	0	0	n/a	2	High	0	
ND_L75P		159	170	LRTHLAPYSDEL	1413.7252	3.6772	30	9	0.167	2.623	14.571	0.458	2	High	0.051	0.00137093
ND_L75P		159	170	LRTHLAPYSDEL	1413.7252	3.6756	300	9	1.46	3.422	19.01	0.273	2	High	0.03	0.00172618
ND_L75P		159	170	LRTHLAPYSDEL	1413.7252	3.6805	3000	9	6.14	4.051	22.504	0.68	2	High	0.076	0.0129721
ND_L75P		159	170	LRTHLAPYSDEL	1413.7252	3.6759	9000	9	8.21	3.978	22.099	0.215	2	High	0.024	0.000382018
ND_L75P		159	173	LRTHLAPYSDELRQR	1853.986	3.3232	0	12	0	0	0	n/a	2	Medium	0	
ND_L75P		159	173	LRTHLAPYSDELRQR	1853.986	3.3607	30	12	0.193	3.247	6.765	0.558	2	Medium	0.062	0.00111386
ND_L75P		159	173	LRTHLAPYSDELRQR	1853.986	3.3792	300	12	1.705	4.626	9.637	0.05	2	Medium	0.006	5.61E-06
ND_L75P		159	173	LRTHLAPYSDELRQR	1853.986	3.387	3000	12	7.827	6.002	12.505	0.687	2	Medium	0.076	0.000316904
ND_L75P		159	173	LRTHLAPYSDELRQR	1853.986	3.3878	9000	12	10.913	5.982	12.463	0.401	2	Medium	0.045	0.00506519
ND_L75P		159	174	LRTHLAPYSDELRQRL	1967.0701	3.5104	0	13	0	0	0	n/a	2	High	0	
ND_L75P		159	174	LRTHLAPYSDELRQRL	1967.0701	3.5063	30	13	0.196	3.247	5.648	0.338	4	Medium	0.212	3.61E-05
ND_L75P		159	174	LRTHLAPYSDELRQRL	1967.0701	3.5156	300	13	1.738	4.865	8.414	0.359	4	High	0.226	2.14E-06
ND_L75P		159	174	LRTHLAPYSDELRQRL	1967.0701	3.5044	3000	13	8.113	6.464	11.217	0.308	4	High	0.194	3.27E-08
ND_L75P		159	174	LRTHLAPYSDELRQRL	1967.0701	3.5022	9000	13	11.55	6.5	11.272	0.229	4	High	0.144	1.31E-08
ND_L75P		159	176	LRTHLAPYSDELRQRLAA	2109.1443	3.5402	0	15	0	0	0	n/a	2	High	0	
ND_L75P		159	176	LRTHLAPYSDELRQRLAA	2109.1443	3.5115	30	15	0.222	4.371	7.285	0.212	2	High	0.024	0.00282429
ND_L75P		159	176	LRTHLAPYSDELRQRLAA	2109.1443	3.5163	300	15	1.984	6.132	10.221	0.609	2	High	0.068	0.00463328
ND_L75P		159	176	LRTHLAPYSDELRQRLAA	2109.1443	3.5206	3000	15	9.564	7.857	13.096	1.343	2	High	0.15	0.00833843
ND_L75P		159	176	LRTHLAPYSDELRQRLAA	2109.1443	3.519	9000	15	13.502	7.886	13.143	1.158	2	High	0.129	0.00114007
ND_L75P		159	178	LRTHLAPYSDELRQRLAARL	2378.3295	4.0203	0	17	0	0	0	n/a	2	High	0	
ND_L75P		159	178	LRTHLAPYSDELRQRLAARL	2378.3295	3.9907	30	17	0.234	4.123	4.433	0.344	4	High	0.217	3.35E-06
ND_L75P		159	178	LRTHLAPYSDELRQRLAARL	2378.3295	3.981	300	17	2.096	6.689	7.203	0.371	4	Medium	0.233	1.90E-05
ND_L75P		159	178	LRTHLAPYSDELRQRLAARL	2378.3295	3.9823	3000	17	10.407	9.034	9.741	0.283	4	Medium	0.178	1.20E-07
ND_L75P		159	178	LRTHLAPYSDELRQRLAARL	2378.3295	3.9822	9000	17	15.052	9.182	9.901	0.24	4	High	0.151	1.26E-08
ND_L75P		159	180	LRTHLAPYSDELRQRLAARLEA	2578.4092	4.0864	0	19	0	0	0	n/a	2	Medium	0	
ND_L75P		159	180	LRTHLAPYSDELRQRLAARLEA	2578.4092	4.0378	30	19	0.257	4.141	5.449	1.616	2	High	0.18	0.0176905
ND_L75P		159	180	LRTHLAPYSDELRQRLAARLEA	2578.4092	4.0565	300	19	2.317	7.314	8.736	1.087	4	Medium	0.683	0.000302346
ND_L75P		159	180	LRTHLAPYSDELRQRLAARLEA	2578.4092	4.0264	3000	19	11.715	10.483	12.438	0.619	4	Medium	0.389	2.87E-07
ND_L75P		159	180	LRTHLAPYSDELRQRLAARLEA	2578.4092	3.9943	9000	19	16.914	10.664	13.115	0.466	3	Medium	0.188	4.28E-06
ND_L75P		160	169	RTHLAPYSDE	1187.5571	2.37	0	7	0	0	0	n/a	2	High	0	
ND_L75P		160	169	RTHLAPYSDE	1187.5571	2.3702	30	7	0.156	2.165	15.466	0.851	2	High	0.095	0.0269246
ND_L75P		160	169	RTHLAPYSDE	1187.5571	2.3795	300	7	1.347	2.626	18.755	0.066	2	High	0.007	6.63E-05
ND_L75P		160	169	RTHLAPYSDE	1187.5571	2.3851	3000	7	5.264	2.964	21.173	0.675	2	High	0.075	0.0129583
ND_L75P		160	169	RTHLAPYSDE	1187.5571	2.3833	9000	7	6.583	2.897	20.696	0.324	2	High	0.036	0.000825961
ND_L75P		160	174	RTHLAPYSDELRQRL	1853.986	3.3232	0	12	0	0	0	n/a	2	Medium	0	
ND_L75P		160	174	RTHLAPYSDELRQRL	1853.986	3.3607	30	12	0.189	3.247	6.765	0.558	2	Medium	0.062	0.00111471
ND_L75P		160	174	RTHLAPYSDELRQRL	1853.986	3.3792	300	12	1.668	4.626	9.637	0.05	2	Medium	0.006	5.61E-06
ND_L75P		160	174	RTHLAPYSDELRQRL	1853.986	3.387	3000	12	7.6	6.002	12.505	0.687	2	Medium	0.076	0.00132571
ND_L75P		160	174	RTHLAPYSDELRQRL	1853.986	3.3878	9000	12	10.665	5.982	12.463	0.401	2	Medium	0.045	0.00506646
ND_L75P		170	176	LRQRLAA	826.5137	1.7838	0	5	0	0	0	n/a	2	High	0	
ND_L75P		170	176	LRQRLAA	826.5137	1.7973	30	5	0.05	1.525	15.249	0.214	2	Medium	0.024	0.00151173
ND_L75P		170	176	LRQRLAA	826.5137	1.7911	300	5	0.472	2.141	21.414	0.061	2	Medium	0.007	1.61E-05
ND_L75P		170	176	LRQRLAA	826.5137	1.7816	3000	5	3.008	2.924	29.236	0.461	2	Medium	0.051	0.0057149
ND_L75P		170	176	LRQRLAA	826.5137	1.7786	9000	5	4.492	3.015	30.153	0.185	2	Medium	0.021	7.09E-05
ND_L75P		170	177	LRQRLAAR	982.6148	2.2315	0	6	0	0	0	n/a	2	High	0	
ND_L75P		170	177	LRQRLAAR	982.6148	2.2117	30	6	0.058	1.687	9.373	0.419	2	High	0.047	0.010286
ND_L75P		170	177	LRQRLAAR	982.6148	2.2123	300	6	0.55	2.485	13.804	0.071	2	High	0.008	0.000151331
ND_L75P		170	177	LRQRLAAR	982.6148	2.1788	3000	6	3.565	3.608	20.043	0.343	2	High	0.038	0.00537177
ND_L75P		170	177	LRQRLAAR	982.6148	2.2151	9000	6	5.405	3.71	20.612	0.182	2	High	0.02	0.00057512
ND_L75P		170	178	LRQRLAARL	1095.6989	2.5025	0	7	0	0	0	n/a	2	High	0	
ND_L75P		170	178	LRQRLAARL	1095.6989	2.4677	30	7	0.061	1.938	9.226	0.412	2	High	0.046	0.00335125
ND_L75P		170	178	LRQRLAARL	1095.6989	2.474	300	7	0.583	2.923	13.918	0.033	2	High	0.004	0.000482568
ND_L75P		170	178	LRQRLAARL	1095.6989	2.4717	3000	7	3.851	4.099	19.521	0.537	2	High	0.06	0.00733305
ND_L75P		170	178	LRQRLAARL	1095.6989	2.4749	9000	7	6.042	4.278	20.372	0.181	2	High	0.02	0.000588401
ND_L75P		170	180	LRQRLAARLEA	1295.7786	2.5228	0	9	0	0	0	n/a	2	High	0	
ND_L75P		170	180	LRQRLAARLEA	1295.7786	2.5139	30	9	0.085	2.271	7.375	0.09	4	Medium	0.057	2.64E-07
ND_L75P		170	180	LRQRLAARLEA	1295.7786	2.5134	300	9	0.804	3.62	11.742	0.064	4	Medium	0.04	3.72E-09
ND_L75P		170	180	LRQRLAARLEA	1295.7786	2.5165	3000	9	5.159	5.061	16.425	0.14	4	High	0.088	3.71E-07
ND_L75P		170	180	LRQRLAARLEA	1295.7786	2.5045	9000	9	7.904	5.402	17.578	0.407	4	High	0.256	4.52E-05
ND_L75P		171	178	RQRLAARL	982.6148	2.2315	0	6	0	0	0	n/a	2	High	0	
ND_L75P		171	178	RQRLAARL	982.6148	2.2117	30	6	0.051	1.687	9.373	0.419	2	High	0.047	0.010286
ND_L75P		171	178	RQRLAARL	982.6148	2.2123	300	6	0.485	2.485	13.804	0.071	2	High	0.008	0.000151331
ND_L75P		171	178	RQRLAARL	982.6148	2.241	3000	6	3.21	3.503	19.463	0.359	2	High	0.04	0.00584566
ND_L75P		171	178	RQRLAARL	982.6148	2.2216	9000	6	5.088	3.71	20.609	0.175	2	High	0.019	0.0004789

**ND_L75P**		171	180	RQRLAARLEA	1182.6945	2.2678	0	8	0	0	0	n/a		2	High	0	
**ND_L75P**		171	180	RQRLAARLEA	1182.6945	2.264	30	8	0.074	2.132	8.882	0.377		2	High	0.042	0.00131186
**ND_L75P**		171	180	RQRLAARLEA	1182.6945	2.2675	300	8	0.707	3.251	13.548	0.269		2	High	0.03	0.000617606
**ND_L75P**		171	180	RQRLAARLEA	1182.6945	2.268	3000	8	4.517	4.639	19.33	0.285		2	High	0.032	0.000870855
**ND_L75P**		171	180	RQRLAARLEA	1182.6945	2.2754	9000	8	6.95	4.833	20.137	0.28		2	High	0.031	8.72E-05
**ND_L75P**		179	189	EALKENGGARL	1156.62	1.9877	0	9	0	0	0	n/a		2	High	0	
**ND_L75P**		179	189	EALKENGGARL	1156.62	1.9824	30	9	0.121	4.56	25.331	0.109		2	High	0.012	0.03027
**ND_L75P**		179	189	EALKENGGARL	1156.62	1.9864	300	9	1.106	4.256	23.645	0.347		2	High	0.039	0.356839
**ND_L75P**		179	189	EALKENGGARL	1156.62	1.9854	3000	9	5.692	4.44	24.667	0.438		2	High	0.049	0.902972
**ND_L75P**		179	189	EALKENGGARL	1156.62	1.9977	9000	9	7.965	4.302	23.897	0.27		2	High	0.03	0.0768377
**ND_L75P**		179	191	EALKENGGARLAE	1356.6997	2.2496	0	11	0	0	0	n/a		2	Medium	0	
**ND_L75P**		179	191	EALKENGGARLAE	1356.6997	2.2316	30	11	0.142	5.74	17.394	0.19		2	Medium	0.021	0.699834
**ND_L75P**		179	191	EALKENGGARLAE	1356.6997	2.2282	300	11	1.308	5.68	17.211	0.21		2	Medium	0.023	0.284053
**ND_L75P**		179	191	EALKENGGARLAE	1356.6997	2.2261	3000	11	7.002	5.966	18.08	0.776		2	Medium	0.086	0.269522
**ND_L75P**		179	191	EALKENGGARLAE	1356.6997	2.1604	9000	11	9.883	5.907	17.9	0.14		2	Medium	0.016	0.15767
**ND_L75P**		179	203	EALKENGGARLAEYHAKATEHLSTL	2708.3882	3.3127	0	23	0	0	0	n/a		2	High	0	
**ND_L75P**		179	203	EALKENGGARLAEYHAKATEHLSTL	2708.3882	3.244	30	23	0.348	8.479	12.289	0.638		2	Medium	0.071	0.000467422
**ND_L75P**		179	203	EALKENGGARLAEYHAKATEHLSTL	2708.3882	3.2531	300	23	3.083	10.724	15.542	0.008		2	High	0.001	0.0610975
**ND_L75P**		179	203	EALKENGGARLAEYHAKATEHLSTL	2708.3882	3.2614	3000	23	14.85	12.32	17.854	0.145		2	High	0.016	0.149011
**ND_L75P**		179	203	EALKENGGARLAEYHAKATEHLSTL	2708.3882	3.2373	9000	23	20.808	12.732	18.452	0.84		2	High	0.093	0.0188104
**ND_L75P**		181	190	LKENGGARLA	1027.5774	1.8812	0	8	0	0	0	n/a		2	Medium	0	
**ND_L75P**		181	190	LKENGGARLA	1027.5774	1.8883	30	8	0.123	4.294	22.369	0.06		4	Medium	0.038	0.876568
**ND_L75P**		181	190	LKENGGARLA	1027.5774	1.8899	300	8	1.122	3.802	19.834	0.057		4	Medium	0.036	0.349387
**ND_L75P**		181	190	LKENGGARLA	1027.5774	1.8902	3000	8	5.638	3.916	20.394	0.068		4	Medium	0.043	0.74923
**ND_L75P**		181	190	LKENGGARLA	1027.5774	1.974	9000	8	7.456	4.246	21.674	0.784		4	Medium	0.493	0.234783
**ND_L75P**		181	191	LKENGGARLAE	1156.62	1.9876	0	9	0	0	0	n/a		2	High	0	
**ND_L75P**		181	191	LKENGGARLAE	1156.62	1.9836	30	9	0.133	4.626	17.134	0.37		2	High	0.041	0.095968
**ND_L75P**		181	191	LKENGGARLAE	1156.62	1.989	300	9	1.221	4.357	16.136	0.197		2	Medium	0.022	0.258392
**ND_L75P**		181	191	LKENGGARLAE	1156.62	1.9932	3000	9	6.285	4.525	16.76	0.731		2	Medium	0.081	0.930832
**ND_L75P**		181	191	LKENGGARLAE	1156.62	1.9847	9000	9	8.412	4.415	16.353	0.072		2	Medium	0.008	0.141525
**ND_L75P**		190	195	AEYHAK	717.3446	1.3949	0	4	0	0	0	n/a		2	Medium	0	
**ND_L75P**		190	195	AEYHAK	717.3446	1.4331	30	4	0.08	1.982	24.777	0.13		2	Medium	0.014	0.00323935
**ND_L75P**		190	195	AEYHAK	717.3446	1.4249	300	4	0.707	1.908	23.855	0.084		2	Medium	0.009	0.15644
**ND_L75P**		190	195	AEYHAK	717.3446	1.4198	3000	4	2.992	2.019	25.238	0.162		2	Medium	0.018	0.0253264
**ND_L75P**		190	195	AEYHAK	717.3446	1.3804	9000	4	3.795	2.017	25.214	0.464		2	Medium	0.052	0.60637
**ND_L75P**		190	200	AEYHAKATEHL	1268.6149	2.0575	0	9	0	0	0	n/a		2	High	0	
**ND_L75P**		190	200	AEYHAKATEHL	1268.6149	2.0114	30	9	0.186	3.1	11.482	0.747		2	High	0.083	0.00844545
**ND_L75P**		190	200	AEYHAKATEHL	1268.6149	2.0237	300	9	1.577	3.709	13.735	0.636		2	Medium	0.071	0.163206
**ND_L75P**		190	200	AEYHAKATEHL	1268.6149	2.0764	3000	9	6.422	4.152	15.376	0.062		2	Medium	0.007	0.125225
**ND_L75P**		190	200	AEYHAKATEHL	1268.6149	2.0299	9000	9	8.488	4.23	15.666	0.228		2	Medium	0.025	0.729405
**ND_L75P**		190	201	AEYHAKATEHLS	1355.647	1.707	0	10	0	0	0	n/a		2	High	0	
**ND_L75P**		190	201	AEYHAKATEHLS	1355.647	1.6883	30	10	0.195	4.769	23.843	0.883		2	High	0.098	0.272565
**ND_L75P**		190	201	AEYHAKATEHLS	1355.647	1.6995	300	10	1.667	4.373	21.867	0.198		2	High	0.022	0.201183
**ND_L75P**		190	201	AEYHAKATEHLS	1355.647	1.8833	3000	10	7.032	4.532	22.66	0.408		2	High	0.045	0.164294
**ND_L75P**		190	201	AEYHAKATEHLS	1355.647	2.0783	9000	10	9.428	5.052	25.259	0.61		2	High	0.068	0.0264063
**ND_L75P**		190	202	AEYHAKATEHLST	1456.6947	1.8856	0	11	0	0	0	n/a		2	High	0	
**ND_L75P**		190	202	AEYHAKATEHLST	1456.6947	1.8929	30	11	0.203	3.65	16.593	0.289		2	High	0.032	0.0075562
**ND_L75P**		190	202	AEYHAKATEHLST	1456.6947	1.89	300	11	1.747	4.528	20.581	1.41		2	High	0.157	0.114738
**ND_L75P**		190	202	AEYHAKATEHLST	1456.6947	1.8924	3000	11	7.598	5.237	23.806	1.154		2	High	0.128	0.384754
**ND_L75P**		190	202	AEYHAKATEHLST	1456.6947	1.9005	9000	11	10.347	5.241	23.824	0.722		2	High	0.08	0.285844
**ND_L75P**		190	203	AEYHAKATEHLSTL	1569.7787	2.7589	0	12	0	0	0	n/a		2	High	0	
**ND_L75P**		190	203	AEYHAKATEHLSTL	1569.7787	2.7559	30	12	0.206	3.874	16.142	0.42		2	High	0.047	0.00263152
**ND_L75P**		190	203	AEYHAKATEHLSTL	1569.7787	2.7617	300	12	1.775	4.844	20.183	1.368		2	High	0.152	0.133645
**ND_L75P**		190	203	AEYHAKATEHLSTL	1569.7787	2.7756	3000	12	7.848	5.579	23.244	1.329		2	High	0.148	0.259258
**ND_L75P**		190	203	AEYHAKATEHLSTL	1569.7787	2.7635	9000	12	10.925	5.53	23.043	0.604		2	High	0.067	0.104011
**ND_L75P**		190	205	AEYHAKATEHLSTLSE	1785.8533	2.5478	0	14	0	0	0	n/a		2	High	0	
**ND_L75P**		190	205	AEYHAKATEHLSTLSE	1785.8533	2.5397	30	14	0.232	4.728	16.886	0.49		2	High	0.055	0.0151078
**ND_L75P**		190	205	AEYHAKATEHLSTLSE	1785.8533	2.5637	300	14	2.016	5.959	21.282	1.809		2	High	0.201	0.161038
**ND_L75P**		190	205	AEYHAKATEHLSTLSE	1785.8533	2.5674	3000	14	9.263	6.893	24.617	1.33		2	High	0.148	0.183151
**ND_L75P**		190	205	AEYHAKATEHLSTLSE	1785.8533	2.5724	9000	14	12.858	6.886	24.593	1.21		2	High	0.135	0.15003
**ND_L75P**		190	211	AEYHAKATEHLSTLSEKAKPAL	2394.2543	2.6809	0	19	0	0	0	n/a		2	High	0	
**ND_L75P**		190	211	AEYHAKATEHLSTLSEKAKPAL	2394.2543	2.6775	30	19	0.275	5.186	6.823	0.585		2	High	0.065	0.0162359
**ND_L75P**		190	211	AEYHAKATEHLSTLSEKAKPAL	2394.2543	2.6424	300	19	2.434	7.541	9.922	1.349		2	High	0.15	0.0181096
**ND_L75P**		190	211	AEYHAKATEHLSTLSEKAKPAL	2394.2543	2.6491	3000	19	12.095	9.159	12.051	1.318		2	High	0.147	0.20533
**ND_L75P**		190	211	AEYHAKATEHLSTLSEKAKPAL	2394.2543	2.6609	9000	19	17.33	9.083	11.951	1.209		2	High	0.135	0.257116
**ND_L75P**		190	212	AEYHAKATEHLSTLSEKAKPALE	2523.2969	2.5891	0	20	0	0	0	n/a		2	High	0	
**ND_L75P**		190	212	AEYHAKATEHLSTLSEKAKPALE	2523.2969	2.5475	30	20	0.282	5.555	9.258	0.274		2	High	0.03	6.45E-05
**ND_L75P**		190	212	AEYHAKATEHLSTLSEKAKPALE	2523.2969	2.5493	300	20	2.5	7.947	13.245	0.176		2	High	0.02	0.0348096
**ND_L75P**		190	212	AEYHAKATEHLSTLSEKAKPALE	2523.2969	2.5572	3000	20	12.586	9.779	16.299	1.508		2	High	0.168	0.180029
**ND_L75P**		190	212	AEYHAKATEHLSTLSEKAKPALE	2523.2969	2.5606	9000	20	18.199	9.779	16.298	0.524		2	High	0.058	0.0392735

**ND_L75P**		190	213	AEYHAKATEHLSTLSEKAKPALED	2638.3239	2.612	0	21	0	0	0	n/a	2	High	0	
**ND_L75P**		190	213	AEYHAKATEHLSTLSEKAKPALED	2638.3239	2.6036	30	21	0.335	5.523	8.767	0.985	2	High	0.11	0.000814533
**ND_L75P**		190	213	AEYHAKATEHLSTLSEKAKPALED	2638.3239	2.5859	300	21	2.917	8.113	12.878	1.074	2	High	0.12	0.00889251
**ND_L75P**		190	213	AEYHAKATEHLSTLSEKAKPALED	2638.3239	2.5873	3000	21	13.582	10.365	16.452	1.114	2	High	0.124	0.109969
**ND_L75P**		190	213	AEYHAKATEHLSTLSEKAKPALED	2638.3239	2.6024	9000	21	19.199	10.217	16.218	1.818	2	High	0.202	0.184921
**ND_L75P**		190	214	AEYHAKATEHLSTLSEKAKPALEDL	2751.4079	3.4918	0	22	0	0	0	n/a	2	High	0	
**ND_L75P**		190	214	AEYHAKATEHLSTLSEKAKPALEDL	2751.4079	3.4836	30	22	0.342	5.263	6.379	0.149	4	Medium	0.094	0.0002354
**ND_L75P**		190	214	AEYHAKATEHLSTLSEKAKPALEDL	2751.4079	3.477	300	22	2.986	7.881	9.585	0.429	4	High	0.27	0.000109649
**ND_L75P**		190	214	AEYHAKATEHLSTLSEKAKPALEDL	2751.4079	3.4592	3000	22	14.093	10.567	12.864	0.592	4	High	0.372	0.198411
**ND_L75P**		190	214	AEYHAKATEHLSTLSEKAKPALEDL	2751.4079	3.4733	9000	22	20.082	10.58	12.849	0.324	4	High	0.203	0.0944872
**ND_L75P**		190	219	AEYHAKATEHLSTLSEKAKPALEDLRQGLL	3318.7572	4.1183	0	27	0	0	0	n/a	2	High	0	
**ND_L75P**		190	219	AEYHAKATEHLSTLSEKAKPALEDLRQGLL	3318.7572	4.0966	30	27	0.377	5.259	4.869	1.013	2	High	0.113	0.000355081
**ND_L75P**		190	219	AEYHAKATEHLSTLSEKAKPALEDLRQGLL	3318.7572	4.0757	300	27	3.326	9.155	8.477	0.542	2	High	0.06	0.00566589
**ND_L75P**		190	219	AEYHAKATEHLSTLSEKAKPALEDLRQGLL	3318.7572	4.0725	3000	27	16.525	12.849	11.897	0.193	2	High	0.021	0.0112305
**ND_L75P**		190	219	AEYHAKATEHLSTLSEKAKPALEDLRQGLL	3318.7572	4.0488	9000	27	24.236	13.827	12.803	2.442	2	High	0.272	0.118742
**ND_L75P**		190	222	AEYHAKATEHLSTLSEKAKPALEDLRQGLLPV	3627.9624	4.5862	0	29	0	0	0	n/a	2	Medium	0	
**ND_L75P**		190	222	AEYHAKATEHLSTLSEKAKPALEDLRQGLLPV	3627.9624	4.6332	30	29	0.381	4.451	5.116	0.866	2	High	0.096	4.54E-05
**ND_L75P**		190	222	AEYHAKATEHLSTLSEKAKPALEDLRQGLLPV	3627.9624	4.6404	300	29	3.366	8.62	7.97	0.906	4	Medium	0.57	0.00026131
**ND_L75P**		190	222	AEYHAKATEHLSTLSEKAKPALEDLRQGLLPV	3627.9624	4.6153	3000	29	16.898	13.396	12.307	0.608	4	Medium	0.382	0.00529737
**ND_L75P**		190	222	AEYHAKATEHLSTLSEKAKPALEDLRQGLLPV	3627.9624	4.6138	9000	29	25.155	14.356	13.213	0.333	4	Medium	0.209	0.830394
**ND_L75P**		192	203	YHAKATEHLSTL	1369.699	2.6074	0	10	0	0	0	n/a	2	Medium	0	
**ND_L75P**		192	203	YHAKATEHLSTL	1369.699	2.5907	30	10	0.166	3.42	11.399	0.172	2	Medium	0.019	0.0315131
**ND_L75P**		192	203	YHAKATEHLSTL	1369.699	2.603	300	10	1.421	4.144	13.814	1.047	2	High	0.116	0.328767
**ND_L75P**		192	203	YHAKATEHLSTL	1369.699	2.6018	3000	10	6.328	4.637	15.456	0.672	2	Medium	0.075	0.100833
**ND_L75P**		192	203	YHAKATEHLSTL	1369.699	2.6155	9000	10	9.01	4.532	15.107	0.085	2	High	0.009	0.0629361
**ND_L75P**		192	211	YHAKATEHLSTLSEKAKPAL	2194.1746	2.5571	0	17	0	0	0	n/a	2	Medium	0	
**ND_L75P**		192	211	YHAKATEHLSTLSEKAKPAL	2194.1746	2.5441	30	17	0.235	4.665	9.147	1.375	2	Medium	0.153	0.0171154
**ND_L75P**		192	211	YHAKATEHLSTLSEKAKPAL	2194.1746	2.5297	300	17	2.08	6.944	13.615	0.422	2	Medium	0.047	0.050289
**ND_L75P**		192	211	YHAKATEHLSTLSEKAKPAL	2194.1746	2.5341	3000	17	10.575	8.288	16.25	1.026	2	Medium	0.114	0.065497
**ND_L75P**		192	211	YHAKATEHLSTLSEKAKPAL	2194.1746	2.536	9000	17	15.416	8.131	15.943	0.344	2	Medium	0.038	0.157576
**ND_L75P**		193	211	HAKATEHLSTLSEKAKPAL	2031.1113	2.4179	0	16	0	0	0	n/a	2	Medium	0	
**ND_L75P**		193	211	HAKATEHLSTLSEKAKPAL	2031.1113	2.3759	30	16	0.203	4.931	8.184	0.448	4	Medium	0.282	5.99E-06
**ND_L75P**		193	211	HAKATEHLSTLSEKAKPAL	2031.1113	2.4087	300	16	1.796	6.306	10.487	0.429	4	Medium	0.27	0.00109579
**ND_L75P**		193	211	HAKATEHLSTLSEKAKPAL	2031.1113	2.4102	3000	16	9.611	7.774	11.84	0.322	3	Medium	0.13	0.0381921
**ND_L75P**		193	211	HAKATEHLSTLSEKAKPAL	2031.1113	2.4274	9000	16	14.416	7.635	11.71	0.402	3	Medium	0.162	0.136805
**ND_L75P**		199	211	HLSTLSEKAKPAL	1393.7929	2.5478	0	10	0	0	0	n/a	2	Medium	0	
**ND_L75P**		199	211	HLSTLSEKAKPAL	1393.7929	2.5476	30	10	0.09	3.064	10.213	0.046	2	Medium	0.005	0.00818348
**ND_L75P**		199	211	HLSTLSEKAKPAL	1393.7929	2.5306	300	10	0.857	4.346	14.488	0.123	2	Medium	0.014	0.0140765
**ND_L75P**		199	211	HLSTLSEKAKPAL	1393.7929	2.5323	3000	10	5.673	5.373	17.91	0.291	2	Medium	0.032	0.0288047
**ND_L75P**		199	211	HLSTLSEKAKPAL	1393.7929	2.529	9000	10	8.843	5.372	17.907	0.027	2	Medium	0.003	0.0843822
**ND_L75P**		201	212	STLSEKAKPALE	1272.6925	2.3379	0	9	0	0	0	n/a	2	Medium	0	
**ND_L75P**		201	212	STLSEKAKPALE	1272.6925	2.3081	30	9	0.079	2.718	15.099	0.441	2	Medium	0.049	0.000478062
**ND_L75P**		201	212	STLSEKAKPALE	1272.6925	2.3248	300	9	0.753	4.154	23.078	0.08	2	Medium	0.009	0.000189507
**ND_L75P**		201	212	STLSEKAKPALE	1272.6925	2.315	3000	9	4.989	5.456	30.311	0.147	2	Medium	0.016	0.353686
**ND_L75P**		201	212	STLSEKAKPALE	1272.6925	2.303	9000	9	7.852	5.582	31.011	0.471	2	Medium	0.052	0.213662
**ND_L75P**		201	219	STLSEKAKPALEDLRQGLL	2068.1528	4.4205	0	16	0	0	0	n/a	2	Medium	0	
**ND_L75P**		201	219	STLSEKAKPALEDLRQGLL	2068.1528	4.4431	30	16	0.174	2.818	5.87	0.621	2	Medium	0.069	0.00448685
**ND_L75P**		201	219	STLSEKAKPALEDLRQGLL	2068.1528	4.4356	300	16	1.579	5.658	11.787	0.353	2	Medium	0.039	0.000788597
**ND_L75P**		201	219	STLSEKAKPALEDLRQGLL	2068.1528	4.4141	3000	16	8.928	8.856	18.449	0.464	2	Medium	0.052	0.0182272
**ND_L75P**		201	219	STLSEKAKPALEDLRQGLL	2068.1528	4.4059	9000	16	13.889	9.507	19.805	0.923	2	Medium	0.103	0.164422
**ND_L75P**		203	212	LSEKAKPALE	1084.6128	1.9374	0	7	0	0	0	n/a	2	High	0	
**ND_L75P**		203	212	LSEKAKPALE	1084.6128	1.9435	30	7	0.067	1.86	13.289	0.088	2	High	0.01	0.00145174
**ND_L75P**		203	212	LSEKAKPALE	1084.6128	1.9206	300	7	0.635	2.882	20.584	0.461	2	High	0.051	0.00269944
**ND_L75P**		203	212	LSEKAKPALE	1084.6128	1.9301	3000	7	4.129	3.935	28.11	0.392	2	High	0.044	0.443952
**ND_L75P**		203	212	LSEKAKPALE	1084.6128	1.9494	9000	7	6.334	3.974	28.382	0.3	2	High	0.033	0.229558
**ND_L75P**		203	213	LSEKAKPALED	1199.6398	2.053	0	8	0	0	0	n/a	2	High	0	
**ND_L75P**		203	213	LSEKAKPALED	1199.6398	2.0446	30	8	0.119	2.01	12.564	0.149	2	High	0.017	0.00496767
**ND_L75P**		203	213	LSEKAKPALED	1199.6398	2.0451	300	8	1.052	3.134	19.585	0.581	2	High	0.065	0.00771048
**ND_L75P**		203	213	LSEKAKPALED	1199.6398	2.0506	3000	8	5.125	4.379	27.366	0.477	2	High	0.053	0.362274
**ND_L75P**		203	213	LSEKAKPALED	1199.6398	2.0803	9000	8	7.334	4.41	27.56	0.134	2	High	0.015	0.175727
**ND_L75P**		203	214	LSEKAKPALEDL	1312.7238	3.6276	0	9	0	0	0	n/a	2	Medium	0	
**ND_L75P**		203	214	LSEKAKPALEDL	1312.7238	3.6254	30	9	0.126	1.959	10.882	0.3	2	High	0.033	0.00442227
**ND_L75P**		203	214	LSEKAKPALEDL	1312.7238	3.6407	300	9	1.121	3.187	17.705	0.165	2	Medium	0.018	0.00651831
**ND_L75P**		203	214	LSEKAKPALEDL	1312.7238	3.647	3000	9	5.636	4.673	25.961	0.745	2	Medium	0.083	0.304348
**ND_L75P**		203	214	LSEKAKPALEDL	1312.7238	3.6213	9000	9	8.217	4.955	27.529	0.521	2	High	0.058	0.118261
**ND_L75P**		203	219	LSEKAKPALEDLRQGLL	1880.0731	4.2369	0	14	0	0	0	n/a	2	High	0	
**ND_L75P**		203	219	LSEKAKPALEDLRQGLL	1880.0731	4.2269	30	14	0.162	2.14	7.642	0.425	2	Medium	0.047	0.00946749

**ND_L75P**		203	219	LSEKAKPALEDLRQGLL	1880.0731	4.2121	300	14	1.461	4.381	15.646	0.307		2	High	0.034	0.0197138
**ND_L75P**		203	219	LSEKAKPALEDLRQGLL	1880.0731	4.2138	3000	14	8.068	7.269	25.959	2.115		2	High	0.235	0.135562
**ND_L75P**		203	219	LSEKAKPALEDLRQGLL	1880.0731	4.208	9000	14	12.371	7.944	28.373	0.912		2	High	0.102	0.526943
**ND_L75P**		203	222	LSEKAKPALEDLRQGLLPVL	2189.2784	4.9225	0	16	0	0	0	n/a		2	Medium	0	
**ND_L75P**		203	222	LSEKAKPALEDLRQGLLPVL	2189.2784	4.9524	30	16	0.166	2.074	5.309	0.342		4	Medium	0.215	5.07E-05
**ND_L75P**		203	222	LSEKAKPALEDLRQGLLPVL	2189.2784	4.9469	300	16	1.501	4.51	11.7	0.949		4	Medium	0.597	0.00032719
**ND_L75P**		203	222	LSEKAKPALEDLRQGLLPVL	2189.2784	4.9423	3000	16	8.441	8	20.864	0.478		4	Medium	0.301	0.00220296
**ND_L75P**		203	222	LSEKAKPALEDLRQGLLPVL	2189.2784	4.937	9000	16	13.29	9.067	23.663	0.267		4	Medium	0.168	0.0240732
**ND_L75P**		204	211	SEKAKPAL	842.4862	1.8446	0	5	0	0	0	n/a		2	High	0	
**ND_L75P**		204	211	SEKAKPAL	842.4862	1.8376	30	5	0.044	1.306	13.058	0.082		2	High	0.009	0.00517931
**ND_L75P**		204	211	SEKAKPAL	842.4862	1.826	300	5	0.418	2.099	20.992	0.189		2	High	0.021	0.00708245
**ND_L75P**		204	211	SEKAKPAL	842.4862	1.8144	3000	5	2.832	2.907	29.074	0.003		2	Medium	0	0.0216374
**ND_L75P**		204	211	SEKAKPAL	842.4862	1.7826	9000	5	4.473	3.016	30.161	0.077		2	Medium	0.009	0.00484518
**ND_L75P**		204	212	SEKAKPALE	971.5288	1.5865	0	6	0	0	0	n/a		2	High	0	
**ND_L75P**		204	212	SEKAKPALE	971.5288	1.5932	30	6	0.05	1.471	12.261	0.232		2	High	0.026	0.00966545
**ND_L75P**		204	212	SEKAKPALE	971.5288	1.5739	300	6	0.483	2.407	20.055	0.465		2	High	0.052	0.00356333
**ND_L75P**		204	212	SEKAKPALE	971.5288	1.5929	3000	6	3.323	3.393	28.277	0.569		2	High	0.063	0.371555
**ND_L75P**		204	212	SEKAKPALE	971.5288	1.5928	9000	6	5.341	3.476	28.97	0.064		2	High	0.007	0.117411
**ND_L75P**		204	213	SEKAKPALED	1086.5557	1.7388	0	7	0	0	0	n/a		2	High	0	
**ND_L75P**		204	213	SEKAKPALED	1086.5557	1.7367	30	7	0.103	1.573	11.239	0.041		2	High	0.005	0.00949493
**ND_L75P**		204	213	SEKAKPALED	1086.5557	1.7364	300	7	0.901	2.597	18.549	0.339		2	High	0.038	0.014012
**ND_L75P**		204	213	SEKAKPALED	1086.5557	1.7406	3000	7	4.319	3.812	27.23	0.281		2	High	0.031	0.914711
**ND_L75P**		204	213	SEKAKPALED	1086.5557	1.7384	9000	7	6.341	3.924	28.027	0.122		2	High	0.014	0.0937908
**ND_L75P**		204	214	SEKAKPALEDL	1199.6398	3.4988	0	8	0	0	0	n/a		2	High	0	
**ND_L75P**		204	214	SEKAKPALEDL	1199.6398	3.4853	30	8	0.11	1.628	10.176	0.197		2	High	0.022	0.00336476
**ND_L75P**		204	214	SEKAKPALEDL	1199.6398	3.5291	300	8	0.97	2.748	17.178	0.473		2	Medium	0.053	0.00190238
**ND_L75P**		204	214	SEKAKPALEDL	1199.6398	3.5102	3000	8	4.829	4.232	26.449	0.468		2	Medium	0.052	0.122707
**ND_L75P**		204	214	SEKAKPALEDL	1199.6398	3.4905	9000	8	7.224	4.452	27.828	0.645		2	Medium	0.072	0.387671
**ND_L75P**		204	219	SEKAKPALEDLRQGLL	1766.989	4.1639	0	13	0	0	0	n/a		2	High	0	
**ND_L75P**		204	219	SEKAKPALEDLRQGLL	1766.989	4.1512	30	13	0.145	1.978	7.61	0.025		2	Medium	0.003	0.00224714
**ND_L75P**		204	219	SEKAKPALEDLRQGLL	1766.989	4.1354	300	13	1.309	4.086	15.715	0.685		2	Medium	0.076	0.00319929
**ND_L75P**		204	219	SEKAKPALEDLRQGLL	1766.989	4.1347	3000	13	7.262	6.909	26.572	1.049		2	Medium	0.117	0.0183488
**ND_L75P**		204	219	SEKAKPALEDLRQGLL	1766.989	4.1389	9000	13	11.378	7.502	28.855	0.243		2	Medium	0.027	0.0580071
**ND_L75P**		204	222	SEKAKPALEDLRQGLLPVL	2076.1943	4.8922	0	15	0	0	0	n/a		2	Medium	0	
**ND_L75P**		204	222	SEKAKPALEDLRQGLLPVL	2076.1943	4.8737	30	15	0.149	2.102	5.759	0.397		4	Medium	0.249	1.44E-08
**ND_L75P**		204	222	SEKAKPALEDLRQGLLPVL	2076.1943	4.8825	300	15	1.35	4.462	12.495	0.771		4	Medium	0.485	0.000191439
**ND_L75P**		204	222	SEKAKPALEDLRQGLLPVL	2076.1943	4.8906	3000	15	7.635	7.574	21.087	0.431		4	Medium	0.271	0.00147016
**ND_L75P**		204	222	SEKAKPALEDLRQGLLPVL	2076.1943	4.8906	9000	15	12.297	8.58	23.876	0.252		4	Medium	0.159	0.00205886
**ND_L75P**		206	222	KAKPALEDLRQGLLPVL	1860.1197	4.8624	0	13	0	0	0	n/a		2	Medium	0	
**ND_L75P**		206	222	KAKPALEDLRQGLLPVL	1860.1197	4.847	30	13	0.127	1.289	4.088	0.139		4	Medium	0.087	1.24E-06
**ND_L75P**		206	222	KAKPALEDLRQGLLPVL	1860.1197	4.8222	300	13	1.132	3.225	10.324	0.354		4	Medium	0.223	1.68E-06
**ND_L75P**		206	222	KAKPALEDLRQGLLPVL	1860.1197	4.8374	3000	13	6.266	6.419	20.731	0.651		4	Medium	0.409	0.00304291
**ND_L75P**		206	222	KAKPALEDLRQGLLPVL	1860.1197	4.8347	9000	13	10.36	7.51	24.234	0.508		4	Medium	0.319	0.0627513
**ND_L75P**		212	219	EDLRQGLL	942.5134	4.0568	0	6	0	0	0	n/a		2	Medium	0	
**ND_L75P**		212	219	EDLRQGLL	942.5134	4.0577	30	6	0.042	0.835	10.467	0.018		4	Medium	0.011	6.01E-06
**ND_L75P**		212	219	EDLRQGLL	942.5134	4.0551	300	6	0.408	1.524	19.16	0.105		4	Medium	0.066	1.77E-08
**ND_L75P**		212	219	EDLRQGLL	942.5134	4.0589	3000	6	2.944	2.847	35.724	0.179		4	Medium	0.113	0.000968101
**ND_L75P**		212	219	EDLRQGLL	942.5134	4.0428	9000	6	5.037	3.348	42.058	0.155		4	Medium	0.097	0.0179597
**ND_L75P**		212	222	EDLRQGLLPVL	1251.7187	5.1778	0	8	0	0	0	n/a		2	High	0	
**ND_L75P**		212	222	EDLRQGLLPVL	1251.7187	5.1721	30	8	0.046	0.899	5.618	0.063		2	High	0.007	9.56E-06
**ND_L75P**		212	222	EDLRQGLLPVL	1251.7187	5.1661	300	8	0.449	1.827	11.416	0.028		2	High	0.003	0.000334816
**ND_L75P**		212	222	EDLRQGLLPVL	1251.7187	5.1538	3000	8	3.316	3.709	23.181	0.254		2	High	0.028	0.0100416
**ND_L75P**		212	222	EDLRQGLLPVL	1251.7187	5.1405	9000	8	5.956	4.495	28.093	0.035		2	High	0.004	0.0147936
**ND_L75P**		213	219	DLRQGLL	813.4709	4.1819	0	5	0	0	0	n/a		2	High	0	
**ND_L75P**		213	219	DLRQGLL	813.4709	4.1819	30	5	0.035	0.737	14.734	0.147		2	High	0.016	0.000118207
**ND_L75P**		213	219	DLRQGLL	813.4709	4.1674	300	5	0.339	1.335	26.69	0.071		2	High	0.008	0.00216819
**ND_L75P**		213	219	DLRQGLL	813.4709	4.1499	3000	5	2.433	2.468	49.363	0.333		2	High	0.037	0.0295838
**ND_L75P**		213	219	DLRQGLL	813.4709	4.1683	9000	5	4.154	2.835	56.693	0.099		2	High	0.011	0.0605207
**ND_L75P**		213	222	DLRQGLLPVL	1122.6761	5.2884	0	7	0	0	0	n/a		2	High	0	
**ND_L75P**		213	222	DLRQGLLPVL	1122.6761	5.2955	30	7	0.039	0.828	5.912	0.074		2	High	0.008	0.00233264
**ND_L75P**		213	222	DLRQGLLPVL	1122.6761	5.2807	300	7	0.38	1.63	11.642	0.104		2	High	0.012	0.00580789
**ND_L75P**		213	222	DLRQGLLPVL	1122.6761	5.284	3000	7	2.805	3.266	23.326	0.269		2	High	0.03	0.0082342
**ND_L75P**		213	222	DLRQGLLPVL	1122.6761	5.2668	9000	7	5.073	4.011	28.65	0.113		2	High	0.013	0.0149417
**ND_L75P**		214	222	LRQGLLPVL	1007.6492	4.9854	0	6	0	0	0	n/a		2	Medium	0	
**ND_L75P**		214	222	LRQGLLPVL	1007.6492	4.9749	30	6	0.034	0.663	7.923	0.174		4	Medium	0.11	2.00E-08
**ND_L75P**		214	222	LRQGLLPVL	1007.6492	4.9693	300	6	0.328	1.301	16.062	0.1		4	Medium	0.063	7.11E-09
**ND_L75P**		214	222	LRQGLLPVL	1007.6492	4.9705	3000	6	2.39	2.72	34.138	0.201		4	Medium	0.126	7.39E-05
**ND_L75P**		214	222	LRQGLLPVL	1007.6492	4.9759	9000	6	4.273	3.366	42.258	0.149		4	Medium	0.093	0.000946066
**ND_L75P**		215	222	RQGLLPVL	894.5651	4.8071	0	5	0	0	0	n/a		2	High	0	
**ND_L75P**		215	222	RQGLLPVL	894.5651	4.7443	30	5	0.024	0.541	5.409	0.083		2	High	0.009	0.00727985
**ND_L75P**		215	222	RQGLLPVL	894.5651	4.7812	300	5	0.23	0.968	9.68	0.253		2	High	0.028	0.00570612

**ND_L75P**		215	222	RQGLLPVL	894.5651	4.7774	3000	5	1.748	2.059	20.594	0.802	2	High	0.089	0.0356822
**ND_L75P**		215	222	RQGLLPVL	894.5651	4.7771	9000	5	3.319	2.661	26.61	0.55	2	High	0.061	0.0130274
**ND_L75P**		223	229	ESFKVSF	842.4174	4.4895	0	5	0	0	0	n/a	2	Medium	0	
**ND_L75P**		223	229	ESFKVSF	842.4174	4.491	30	5	0.03	0.553	11.062	0.16	2	Medium	0.018	0.00018319
**ND_L75P**		223	229	ESFKVSF	842.4174	4.507	300	5	0.295	1.128	22.567	0.027	2	Medium	0.003	0.000646674
**ND_L75P**		223	229	ESFKVSF	842.4174	4.4643	3000	5	2.211	2.269	45.382	0.714	2	Medium	0.079	0.0112562
**ND_L75P**		223	229	ESFKVSF	842.4174	4.4951	9000	5	3.967	2.825	56.501	0.512	2	Medium	0.057	0.0608472
**ND_L75P**		226	232	KVSFLSA	750.4276	3.8802	0	5	0	0	0	n/a	2	Medium	0	
**ND_L75P**		226	232	KVSFLSA	750.4276	3.8819	30	5	0.041	0.648	12.954	0.409	2	Medium	0.046	0.000655621
**ND_L75P**		226	232	KVSFLSA	750.4276	3.8716	300	5	0.393	1.414	28.279	0.09	2	Medium	0.01	0.0117988
**ND_L75P**		226	232	KVSFLSA	750.4276	3.8684	3000	5	2.659	2.643	52.852	0.367	2	Medium	0.041	0.0426919
**ND_L75P**		226	232	KVSFLSA	750.4276	3.8705	9000	5	4.246	2.887	57.733	0.275	2	Medium	0.031	0.269399
**ND_L75P**		230	235	LSALEE	660.333	3.0389	0	4	0	0	0	n/a	2	Medium	0	
**ND_L75P**		230	235	LSALEE	660.333	3.0517	30	4	0.044	0.572	14.293	0.179	2	Medium	0.02	0.0038185
**ND_L75P**		230	235	LSALEE	660.333	3.0299	300	4	0.414	1.082	27.045	0.091	2	Medium	0.01	0.00816898
**ND_L75P**		230	235	LSALEE	660.333	3.0334	3000	4	2.434	2.066	51.646	0.362	2	Medium	0.04	0.0210441
**ND_L75P**		230	235	LSALEE	660.333	3.0289	9000	4	3.559	2.35	58.754	0.41	2	Medium	0.046	0.0839963
**ND_L75P**		233	243	LEEYTKKLNTQ	1365.714	2.3356	0	9	0	0	0	n/a	2	Medium	0	
**ND_L75P**		233	243	LEEYTKKLNTQ	1365.714	2.3241	30	9	0.072	4.17	30.944	0.118	3	Medium	0.047	0.00380155
**ND_L75P**		233	243	LEEYTKKLNTQ	1365.714	2.3204	300	9	0.686	4.84	40.426	0.086	4	Medium	0.054	0.157923
**ND_L75P**		233	243	LEEYTKKLNTQ	1365.714	2.3245	3000	9	4.527	5.354	44.948	0.369	4	Medium	0.232	0.0653529
**ND_L75P**		233	243	LEEYTKKLNTQ	1365.714	2.3412	9000	9	7.358	5.207	43.619	0.268	4	Medium	0.168	0.995123
**ND_L75P**		234	243	EEYTKKLNTQ	1252.6299	1.904	0	8	0	0	0	n/a	2	High	0	
**ND_L75P**		234	243	EEYTKKLNTQ	1252.6299	1.8968	30	8	0.052	4.137	51.707	0.102	2	High	0.011	0.0433182
**ND_L75P**		234	243	EEYTKKLNTQ	1252.6299	1.8821	300	8	0.503	4.713	58.915	0.372	2	High	0.041	0.236762
**ND_L75P**		234	243	EEYTKKLNTQ	1252.6299	1.9205	3000	8	3.661	5.102	63.769	0.722	2	High	0.08	0.197118
**ND_L75P**		234	243	EEYTKKLNTQ	1252.6299	1.8849	9000	8	6.361	5.09	63.621	0.527	2	High	0.059	0.137603
**ND_L75P**		236	243	YTKKLNTQ	994.5447	1.4311	0	6	0	0	0	n/a	2	High	0	
**ND_L75P**		236	243	YTKKLNTQ	994.5447	1.3923	30	6	0.039	3.484	58.075	0.125	2	High	0.014	0.180841
**ND_L75P**		236	243	YTKKLNTQ	994.5447	1.4259	300	6	0.375	3.562	59.372	0.036	2	High	0.004	0.0792392
**ND_L75P**		236	243	YTKKLNTQ	994.5447	1.4211	3000	6	2.705	3.71	61.837	0.532	2	High	0.059	0.105565
**ND_L75P**		236	243	YTKKLNTQ	994.5447	1.4255	9000	6	4.666	3.598	59.97	0.113	2	Medium	0.013	0.0917944
**ND_L174S**		17	28	VYVDVLKDSGRD	1364.6936	3.3292	0	10	0	0	0	n/a	2	High	0	
**ND_L174S**		17	28	VYVDVLKDSGRD	1364.6936	3.3078	30	10	0.164	1.933	9.666	0.357	2	High	0.04	0.0950565
**ND_L174S**		17	28	VYVDVLKDSGRD	1364.6936	3.3221	300	10	1.45	2.739	13.694	0.517	2	High	0.057	0.542742
**ND_L174S**		17	28	VYVDVLKDSGRD	1364.6936	3.3177	3000	10	6.286	4.218	21.089	0.216	2	High	0.024	0.00493031
**ND_L174S**		17	28	VYVDVLKDSGRD	1364.6936	3.3035	9000	10	8.411	4.757	23.787	0.168	2	High	0.019	0.0243032
**ND_L174S**		17	46	VYVDVLKDSGRDYVSQFEGSALGKQLNLKL	3340.7667	5.3348	0	28	0	0	0	n/a	2	Medium	0	
**ND_L174S**		17	46	VYVDVLKDSGRDYVSQFEGSALGKQLNLKL	3340.7667	5.3077	30	28	0.322	3.93	2.807	4.209	2	High	0.468	0.224016
**ND_L174S**		17	46	VYVDVLKDSGRDYVSQFEGSALGKQLNLKL	3340.7667	5.2899	300	28	2.951	8.438	6.027	3.133	2	High	0.349	0.373724
**ND_L174S**		17	46	VYVDVLKDSGRDYVSQFEGSALGKQLNLKL	3340.7667	5.264	3000	28	16.007	14.433	10.309	2.613	2	Medium	0.291	0.150598
**ND_L174S**		17	46	VYVDVLKDSGRDYVSQFEGSALGKQLNLKL	3340.7667	5.2521	9000	28	23.684	15.831	11.308	0.005	2	High	0.001	0.141406
**ND_L174S**		18	28	YVDVLKDSGRD	1265.6252	3.0835	0	9	0	0	0	n/a	2	High	0	
**ND_L174S**		18	28	YVDVLKDSGRD	1265.6252	3.0058	30	9	0.163	2.099	11.658	0.814	2	High	0.091	0.98994
**ND_L174S**		18	28	YVDVLKDSGRD	1265.6252	3.0239	300	9	1.432	2.784	15.468	0.577	2	High	0.064	0.370399
**ND_L174S**		18	28	YVDVLKDSGRD	1265.6252	3.0284	3000	9	6.117	4.037	22.429	0.006	2	Medium	0.001	0.00298844
**ND_L174S**		18	28	YVDVLKDSGRD	1265.6252	3.0439	9000	9	7.986	4.391	24.392	0.575	2	Medium	0.064	0.0285416
**ND_L174S**		19	28	VDVLKDSGRD	1102.5619	2.362	0	8	0	0	0	n/a	2	High	0	
**ND_L174S**		19	28	VDVLKDSGRD	1102.5619	2.3264	30	8	0.145	2.006	12.535	0.227	2	High	0.025	0.135259
**ND_L174S**		19	28	VDVLKDSGRD	1102.5619	2.3422	300	8	1.269	2.612	16.324	0.378	2	High	0.042	0.329521
**ND_L174S**		19	28	VDVLKDSGRD	1102.5619	2.3414	3000	8	5.286	3.723	23.266	0.161	2	High	0.018	0.00736728
**ND_L174S**		19	28	VDVLKDSGRD	1102.5619	2.3253	9000	8	6.991	4.085	25.533	0.03	2	High	0.003	0.0950072
**ND_L174S**		19	44	VDVLKDSGRDYVSQFEGSALGKQLNL	2837.4559	5.2049	0	24	0	0	0	n/a	2	High	0	
**ND_L174S**		19	44	VDVLKDSGRDYVSQFEGSALGKQLNL	2837.4559	5.145	30	24	0.295	3.297	3.927	0.939	4	High	0.59	0.641732
**ND_L174S**		19	44	VDVLKDSGRDYVSQFEGSALGKQLNL	2837.4559	5.1369	300	24	2.694	6.921	8.41	0.35	4	High	0.22	0.115259
**ND_L174S**		19	44	VDVLKDSGRDYVSQFEGSALGKQLNL	2837.4559	5.1055	3000	24	14.365	12.664	15.325	1.278	4	High	0.803	0.264326
**ND_L174S**		19	44	VDVLKDSGRDYVSQFEGSALGKQLNL	2837.4559	5.111	9000	24	20.902	13.711	16.665	0.226	4	High	0.142	0.365895
**ND_L174S**		20	28	DVLKDSGRD	1003.4934	2.3545	0	7	0	0	0	n/a	2	High	0	
**ND_L174S**		20	28	DVLKDSGRD	1003.4934	2.3334	30	7	0.14	1.799	12.851	0.175	2	High	0.019	0.0184649
**ND_L174S**		20	28	DVLKDSGRD	1003.4934	2.3215	300	7	1.219	2.388	17.06	0.285	2	High	0.032	0.095349
**ND_L174S**		20	28	DVLKDSGRD	1003.4934	2.3285	3000	7	4.883	3.404	24.316	0.318	2	High	0.035	0.0926061
**ND_L174S**		20	28	DVLKDSGRD	1003.4934	2.3138	9000	7	6.203	3.691	26.367	0.013	2	High	0.001	0.281588
**ND_L174S**		29	37	YVSQFEGSA	986.4345	3.2622	0	7	0	0	0	n/a	2	High	0	
**ND_L174S**		29	37	YVSQFEGSA	986.4345	3.2439	30	7	0.086	2.291	32.723	0.399	2	High	0.044	0.0151929
**ND_L174S**		29	37	YVSQFEGSA	986.4345	3.2516	300	7	0.805	2.857	40.818	0.062	2	High	0.007	0.0586589
**ND_L174S**		29	37	YVSQFEGSA	986.4345	3.2563	3000	7	4.753	3.765	53.782	0.282	2	High	0.031	0.00726382
**ND_L174S**		29	37	YVSQFEGSA	986.4345	3.2541	9000	7	6.655	3.848	54.972	0.012	2	High	0.001	0.278898

**ND_L174S**		29	38	YVSQFEGSAL	1099.5186	4.2709	0	8	0	0	0	n/a	2	High	0	
**ND_L174S**		29	38	YVSQFEGSAL	1099.5186	4.2522	30	8	0.09	2.813	26.375	0.043	4	High	0.027	0.0480735
**ND_L174S**		29	38	YVSQFEGSAL	1099.5186	4.2675	300	8	0.845	3.462	32.414	0.04	4	High	0.025	0.000548172
**ND_L174S**		29	38	YVSQFEGSAL	1099.5186	4.271	3000	8	5.092	4.385	41.123	0.058	4	Medium	0.036	0.000360243
**ND_L174S**		29	38	YVSQFEGSAL	1099.5186	4.2651	9000	8	7.366	4.451	41.795	0.067	4	Medium	0.042	0.0336064
**ND_L174S**		29	43	YVSQFEGSALGKQLN	1639.8206	3.917	0	13	0	0	0	n/a	2	High	0	
**ND_L174S**		29	43	YVSQFEGSALGKQLN	1639.8206	3.8845	30	13	0.131	4.383	16.859	0.455	2	High	0.051	0.793187
**ND_L174S**		29	43	YVSQFEGSALGKQLN	1639.8206	3.8976	300	13	1.239	5.834	22.439	0.567	2	High	0.063	0.257859
**ND_L174S**		29	43	YVSQFEGSALGKQLN	1639.8206	3.892	3000	13	7.811	7.555	29.057	0.443	2	High	0.049	0.00973913
**ND_L174S**		29	43	YVSQFEGSALGKQLN	1639.8206	3.8938	9000	13	11.753	7.68	29.537	0.17	2	High	0.019	0.241111
**ND_L174S**		29	44	YVSQFEGSALGKQLNL	1752.9046	4.8354	0	14	0	0	0	n/a	2	High	0	
**ND_L174S**		29	44	YVSQFEGSALGKQLNL	1752.9046	4.8023	30	14	0.135	4.454	15.906	0.597	2	High	0.066	0.0421993
**ND_L174S**		29	44	YVSQFEGSALGKQLNL	1752.9046	4.8069	300	14	1.285	6.061	21.645	0.574	2	High	0.064	0.186056
**ND_L174S**		29	44	YVSQFEGSALGKQLNL	1752.9046	4.8055	3000	14	8.184	8.078	28.851	0.77	2	High	0.086	0.0500318
**ND_L174S**		29	44	YVSQFEGSALGKQLNL	1752.9046	4.8073	9000	14	12.506	8.207	29.309	0.017	2	High	0.002	0.535829
**ND_L174S**		29	46	YVSQFEGSALGKQLNLKL	1994.0837	4.9254	0	16	0	0	0	n/a	2	High	0	
**ND_L174S**		29	46	YVSQFEGSALGKQLNLKL	1994.0837	4.7982	30	16	0.143	5.303	16.571	0.732	2	High	0.081	0.0296494
**ND_L174S**		29	46	YVSQFEGSALGKQLNLKL	1994.0837	4.8069	300	16	1.361	7.306	22.831	0.84	2	High	0.094	0.913129
**ND_L174S**		29	46	YVSQFEGSALGKQLNLKL	1994.0837	4.8068	3000	16	8.826	9.737	30.428	0.769	2	High	0.086	0.0730783
**ND_L174S**		29	46	YVSQFEGSALGKQLNLKL	1994.0837	4.8061	9000	16	13.868	9.886	30.893	0.323	2	High	0.036	0.32095
**ND_L174S**		33	38	FEGSAL	622.2962	3.5761	0	4	0	0	0	n/a	2	High	0	
**ND_L174S**		33	38	FEGSAL	622.2962	3.5488	30	4	0.053	1.875	46.877	0.136	2	Medium	0.015	0.324664
**ND_L174S**		33	38	FEGSAL	622.2962	3.5665	300	4	0.492	1.956	48.893	0.065	2	Medium	0.007	0.120207
**ND_L174S**		33	38	FEGSAL	622.2962	3.571	3000	4	2.714	2.122	53.054	0.143	2	Medium	0.016	0.763067
**ND_L174S**		33	38	FEGSAL	622.2962	3.5655	9000	4	3.676	2.096	52.405	0.378	2	Medium	0.042	0.583435
**ND_L174S**		33	43	FEGSALGKQLN	1162.5982	3.221	0	9	0	0	0	n/a	2	Medium	0	
**ND_L174S**		33	43	FEGSALGKQLN	1162.5982	3.1859	30	9	0.094	3.231	17.948	0.04	2	Medium	0.004	0.0398598
**ND_L174S**		33	43	FEGSALGKQLN	1162.5982	3.2155	300	9	0.886	4.068	22.602	0.225	2	Medium	0.025	0.0218358
**ND_L174S**		33	43	FEGSALGKQLN	1162.5982	3.1971	3000	9	5.433	5.063	28.13	0.015	2	Medium	0.002	0.0411214
**ND_L174S**		33	43	FEGSALGKQLN	1162.5982	3.2041	9000	9	8.062	5.099	28.329	0.261	2	Medium	0.029	0.625657
**ND_L174S**		33	44	FEGSALGKQLNL	1275.6823	4.4325	0	10	0	0	0	n/a	2	Medium	0	
**ND_L174S**		33	44	FEGSALGKQLNL	1275.6823	4.4326	30	10	0.099	3.275	16.377	0.511	2	Medium	0.057	0.0102247
**ND_L174S**		33	44	FEGSALGKQLNL	1275.6823	4.4207	300	10	0.932	4.287	21.435	0.066	2	High	0.007	0.0140987
**ND_L174S**		33	44	FEGSALGKQLNL	1275.6823	4.42	3000	10	5.806	5.406	27.032	0.517	2	Medium	0.058	0.0864818
**ND_L174S**		33	44	FEGSALGKQLNL	1275.6823	4.4136	9000	10	8.815	5.315	26.576	1.026	2	Medium	0.114	0.0997173
**ND_L174S**		33	46	FEGSALGKQLNLKL	1516.8613	4.4833	0	12	0	0	0	n/a	2	High	0	
**ND_L174S**		33	46	FEGSALGKQLNLKL	1516.8613	4.447	30	12	0.106	4.322	18.008	0.417	2	High	0.046	0.00769042
**ND_L174S**		33	46	FEGSALGKQLNLKL	1516.8613	4.457	300	12	1.008	5.687	23.694	0.511	2	High	0.057	0.0145666
**ND_L174S**		33	46	FEGSALGKQLNLKL	1516.8613	4.4538	3000	12	6.448	7.333	30.555	0.082	2	High	0.009	0.0596814
**ND_L174S**		33	46	FEGSALGKQLNLKL	1516.8613	4.4523	9000	12	10.177	7.383	30.763	0.294	2	High	0.033	0.80144
**ND_L174S**		34	43	EGSALGKQLN	1015.5298	2.4615	0	8	0	0	0	n/a	2	Medium	0	
**ND_L174S**		34	43	EGSALGKQLN	1015.5298	2.4123	30	8	0.08	2.79	17.437	0.233	2	Medium	0.026	0.00822391
**ND_L174S**		34	43	EGSALGKQLN	1015.5298	2.4699	300	8	0.757	3.478	21.74	0.94	2	Medium	0.105	0.113369
**ND_L174S**		34	43	EGSALGKQLN	1015.5298	2.4486	3000	8	4.685	4.375	27.342	0.851	2	Medium	0.095	0.817607
**ND_L174S**		34	43	EGSALGKQLN	1015.5298	2.4583	9000	8	7.078	4.322	27.011		1	Medium	0	
**ND_L174S**		34	44	EGSALGKQLNL	1128.6139	3.9355	0	9	0	0	0	n/a	2	Medium	0	
**ND_L174S**		34	44	EGSALGKQLNL	1128.6139	3.9379	30	9	0.085	2.93	16.278	0.29	2	Medium	0.032	0.024848
**ND_L174S**		34	44	EGSALGKQLNL	1128.6139	3.9543	300	9	0.803	3.821	21.229	0.077	2	Medium	0.009	0.0561741
**ND_L174S**		34	44	EGSALGKQLNL	1128.6139	3.9199	3000	9	5.058	4.96	27.555	0.147	2	Medium	0.016	0.0214445
**ND_L174S**		34	44	EGSALGKQLNL	1128.6139	3.9136	9000	9	7.831	5.047	28.04	1.042	2	Medium	0.116	0.268847
**ND_L174S**		34	46	EGSALGKQLNLKL	1369.7929	4.142	0	11	0	0	0	n/a	2	High	0	
**ND_L174S**		34	46	EGSALGKQLNLKL	1369.7929	4.1206	30	11	0.093	3.614	16.429	0.343	2	High	0.038	0.0314157
**ND_L174S**		34	46	EGSALGKQLNLKL	1369.7929	4.1354	300	11	0.88	4.936	22.434	0.42	2	High	0.047	0.0193913
**ND_L174S**		34	46	EGSALGKQLNLKL	1369.7929	4.129	3000	11	5.7	6.438	29.264	0.637	2	High	0.071	0.19342
**ND_L174S**		34	46	EGSALGKQLNLKL	1369.7929	4.126	9000	11	9.193	6.486	29.482	0.037	2	High	0.004	0.694831
**ND_L174S**		36	46	SALGKQLNLKL	1183.7289	4.0734	0	9	0	0	0	n/a	2	High	0	
**ND_L174S**		36	46	SALGKQLNLKL	1183.7289	4.044	30	9	0.058	2.851	10.56	0.46	2	High	0.051	0.00236873
**ND_L174S**		36	46	SALGKQLNLKL	1183.7289	4.0625	300	9	0.557	4.211	15.598	1.276	2	High	0.142	0.0823623
**ND_L174S**		36	46	SALGKQLNLKL	1183.7289	4.0616	3000	9	4.073	5.562	20.6	0.299	2	High	0.033	0.471527
**ND_L174S**		36	46	SALGKQLNLKL	1183.7289	4.0568	9000	9	7.213	5.723	21.196	0.782	2	High	0.087	0.9753
**ND_L174S**		38	44	LGKQLNL	784.4807	3.4725	0	5	0	0	0	n/a	2	Medium	0	
**ND_L174S**		38	44	LGKQLNL	784.4807	3.3963	30	5	0.038	1.395	13.951	0.512	2	Medium	0.057	0.0263939
**ND_L174S**		38	44	LGKQLNL	784.4807	3.4401	300	5	0.367	2.073	20.725	0.051	2	Medium	0.006	0.00961135
**ND_L174S**		38	44	LGKQLNL	784.4807	3.4158	3000	5	2.565	2.85	28.502	0.165	2	Medium	0.018	0.124379
**ND_L174S**		38	44	LGKQLNL	784.4807	3.4021	9000	5	4.246	2.908	29.078	0.154	2	Medium	0.017	0.10149
**ND_L174S**		38	46	LGKQLNLKL	1025.6597	3.788	0	7	0	0	0	n/a	2	High	0	
**ND_L174S**		38	46	LGKQLNLKL	1025.6597	3.7698	30	7	0.046	2.061	14.724	0.34	2	High	0.038	0.0259304
**ND_L174S**		38	46	LGKQLNLKL	1025.6597	3.7782	300	7	0.444	3.083	22.02	0.169	2	High	0.019	0.0765653
**ND_L174S**		38	46	LGKQLNLKL	1025.6597	3.7792	3000	7	3.208	4.198	29.988	0.136	2	High	0.015	0.0537401
**ND_L174S**		38	46	LGKQLNLKL	1025.6597	3.7785	9000	7	5.608	4.26	30.427	0.078	2	Medium	0.009	0.920424

**ND_L174S**		39	44	GKQLNL	671.3966	3.0699	0	4	0	0	0	n/a	2	High	0	
**ND_L174S**		39	44	GKQLNL	671.3966	3.0347	30	4	0.027	1.18	29.49	0.107	2	Medium	0.012	0.0411087
**ND_L174S**		39	44	GKQLNL	671.3966	3.0503	300	4	0.263	1.755	43.879	0.189	2	Medium	0.021	0.172412
**ND_L174S**		39	44	GKQLNL	671.3966	3.043	3000	4	1.898	2.462	61.549	0.127	2	Medium	0.014	0.261249
**ND_L174S**		39	44	GKQLNL	671.3966	3.0294	9000	4	3.283	2.466	61.644	0.465	2	Medium	0.052	0.283069
**ND_L174S**		39	46	GKQLNLKL	912.5757	3.5761	0	6	0	0	0	n/a	2	Medium	0	
**ND_L174S**		39	46	GKQLNLKL	912.5757	3.5365	30	6	0.035	1.762	29.364	0.1	2	Medium	0.011	0.0320543
**ND_L174S**		39	46	GKQLNLKL	912.5757	3.5608	300	6	0.34	2.633	43.884	0.081	2	Medium	0.009	0.0343193
**ND_L174S**		39	46	GKQLNLKL	912.5757	3.5592	3000	6	2.541	3.652	60.875	0.284	2	Medium	0.032	0.894712
**ND_L174S**		39	46	GKQLNLKL	912.5757	3.5583	9000	6	4.644	3.69	61.495	0.025	2	Medium	0.003	0.348965
**ND_L174S**		45	50	KLLDNW	787.4228	4.8499	0	4	0	0	0	n/a	2	Medium	0	
**ND_L174S**		45	50	KLLDNW	787.4228	4.5002	30	4	0.08	1.093	27.321	0.294	2	Medium	0.033	0.00250152
**ND_L174S**		45	50	KLLDNW	787.4228	4.5117	300	4	0.674	1.565	39.12	0.113	2	Medium	0.013	0.000544101
**ND_L174S**		45	50	KLLDNW	787.4228	4.5124	3000	4	2.557	2.112	52.812	0.16	2	Medium	0.018	0.106638
**ND_L174S**		45	50	KLLDNW	787.4228	4.5093	9000	4	3.449	2.123	53.074	0.094	2	Medium	0.01	0.278022
**ND_L174S**		45	51	KLLDNWD	902.4498	4.2524	0	5	0	0	0	n/a	2	Medium	0	
**ND_L174S**		45	51	KLLDNWD	902.4498	4.1778	30	5	0.098	1.657	16.57	0.575	2	Medium	0.064	0.0122005
**ND_L174S**		45	51	KLLDNWD	902.4498	4.1942	300	5	0.84	1.939	19.391	0.126	2	Medium	0.014	0.00210292
**ND_L174S**		45	51	KLLDNWD	902.4498	4.1852	3000	5	3.395	2.314	23.141	0.589	2	Medium	0.066	0.0866661
**ND_L174S**		45	51	KLLDNWD	902.4498	4.1847	9000	5	4.445	2.225	22.253	0.76	2	Medium	0.085	0.11945
**ND_L174S**		45	56	KLLDNWDSVTST	1377.6776	4.2617	0	10	0	0	0	n/a	2	High	0	
**ND_L174S**		45	56	KLLDNWDSVTST	1377.6776	4.2253	30	10	0.173	3.319	16.593	0.228	2	Medium	0.025	0.000264622
**ND_L174S**		45	56	KLLDNWDSVTST	1377.6776	4.2598	300	10	1.499	4.228	21.141	0.292	2	Medium	0.033	0.000772207
**ND_L174S**		45	56	KLLDNWDSVTST	1377.6776	4.2834	3000	10	6.315	5.247	26.235	0.764	2	Medium	0.085	0.0572846
**ND_L174S**		45	56	KLLDNWDSVTST	1377.6776	4.1943	9000	10	8.564	5.358	26.789	0.052	2	Medium	0.006	0.225974
**ND_L174S**		47	56	LDNWDSVTST	1136.4986	3.9631	0	8	0	0	0	n/a	2	High	0	
**ND_L174S**		47	56	LDNWDSVTST	1136.4986	3.9356	30	8	0.154	3.195	39.944	0.154	2	High	0.017	0.00327739
**ND_L174S**		47	56	LDNWDSVTST	1136.4986	3.9506	300	8	1.317	3.84	48.004	0.052	2	High	0.006	0.0163908
**ND_L174S**		47	56	LDNWDSVTST	1136.4986	3.945	3000	8	5.25	4.621	57.757	0.141	2	High	0.016	0.357485
**ND_L174S**		47	56	LDNWDSVTST	1136.4986	3.9491	9000	8	6.987	4.595	57.435	0.01	2	High	0.001	0.523801
**ND_L174S**		50	56	WDSVTST	794.3446	3.0312	0	5	0	0	0	n/a	2	Medium	0	
**ND_L174S**		50	56	WDSVTST	794.3446	3.0386	30	5	0.075	2.072	41.448	0.061	2	Medium	0.007	0.013808
**ND_L174S**		50	56	WDSVTST	794.3446	3.0409	300	5	0.659	2.501	50.021	0.184	2	Medium	0.02	0.00769751
**ND_L174S**		50	56	WDSVTST	794.3446	3.0653	3000	5	2.92	2.857	57.147	0.319	2	Medium	0.035	0.338568
**ND_L174S**		50	56	WDSVTST	794.3446	3.0506	9000	5	4.119	2.849	56.971	0.066	2	Medium	0.007	0.410785
**ND_L174S**		51	56	DSVTST	608.2653	1.5022	0	4	0	0	0	n/a	2	High	0	
**ND_L174S**		51	56	DSVTST	608.2653	1.4874	30	4	0.032	1.599	39.984	0.117	2	High	0.013	0.00182748
**ND_L174S**		51	56	DSVTST	608.2653	1.4971	300	4	0.305	1.969	49.233	0.027	2	High	0.003	0.0306423
**ND_L174S**		51	56	DSVTST	608.2653	1.4934	3000	4	1.933	2.411	60.269	0.093	2	High	0.01	0.130619
**ND_L174S**		51	56	DSVTST	608.2653	1.4977	9000	4	3.119	2.417	60.421	0.057	2	High	0.006	0.755228
**ND_L174S**		51	57	DSVTSTF	755.3337	3.6176	0	5	0	0	0	n/a	2	High	0	
**ND_L174S**		51	57	DSVTSTF	755.3337	3.5919	30	5	0.037	1.764	35.278	0.046	2	High	0.005	0.000166577
**ND_L174S**		51	57	DSVTSTF	755.3337	3.6059	300	5	0.356	2.233	44.669	0.087	2	Medium	0.01	0.00966988
**ND_L174S**		51	57	DSVTSTF	755.3337	3.6015	3000	5	2.34	2.881	57.622	0.032	2	Medium	0.004	0.0254678
**ND_L174S**		51	57	DSVTSTF	755.3337	3.5973	9000	5	3.91	2.959	59.184	0.021	2	Medium	0.002	0.119361
**ND_L174S**		57	70	FSKLREQLGPVTQE	1630.8679	3.2118	0	11	0	0	0	n/a	2	High	0	
**ND_L174S**		57	70	FSKLREQLGPVTQE	1630.8679	3.2064	30	11	0.085	2.056	9.344	0.412	2	Medium	0.046	0.0166225
**ND_L174S**		57	70	FSKLREQLGPVTQE	1630.8679	3.1784	300	11	0.814	3.721	16.912	0.12	2	High	0.013	0.955311
**ND_L174S**		57	70	FSKLREQLGPVTQE	1630.8679	3.1946	3000	11	5.447	5.632	25.601	0.477	2	Medium	0.053	0.0503159
**ND_L174S**		57	70	FSKLREQLGPVTQE	1630.8679	3.2079	9000	11	8.858	5.913	26.876	0.573	2	Medium	0.064	0.779417
**ND_L174S**		57	71	FSKLREQLGPVTQEF	1777.9363	4.1422	0	12	0	0	0	n/a	2	High	0	
**ND_L174S**		57	71	FSKLREQLGPVTQEF	1777.9363	4.1728	30	12	0.093	2.184	9.1	1.416	2	High	0.158	0.0395037
**ND_L174S**		57	71	FSKLREQLGPVTQEF	1777.9363	4.1469	300	12	0.892	3.989	16.622	0.578	2	High	0.064	0.25601
**ND_L174S**		57	71	FSKLREQLGPVTQEF	1777.9363	4.1316	3000	12	6.004	6.291	26.214	0.131	2	High	0.015	0.00433462
**ND_L174S**		57	71	FSKLREQLGPVTQEF	1777.9363	4.1569	9000	12	9.771	6.747	28.112	0.48	2	High	0.053	0.0599057
**ND_L174S**		58	70	SKLREQLGPVTQE	1483.7995	2.7876	0	10	0	0	0	n/a	2	Medium	0	
**ND_L174S**		58	70	SKLREQLGPVTQE	1483.7995	2.7517	30	10	0.076	1.703	8.517	0.048	2	Medium	0.005	0.0383948
**ND_L174S**		58	70	SKLREQLGPVTQE	1483.7995	2.782	300	10	0.725	3.087	15.433	0.792	2	Medium	0.088	0.198173
**ND_L174S**		58	70	SKLREQLGPVTQE	1483.7995	2.7624	3000	10	4.841	5.365	26.827	0.457	2	Medium	0.051	0.0482018
**ND_L174S**		58	70	SKLREQLGPVTQE	1483.7995	2.7544	9000	10	7.919	5.655	28.273	0.33	2	Medium	0.037	0.223426
**ND_L174S**		58	71	SKLREQLGPVTQEF	1630.8679	3.9009	0	11	0	0	0	n/a	2	Medium	0	
**ND_L174S**		58	71	SKLREQLGPVTQEF	1630.8679	3.85	30	11	0.084	1.98	7.556	0.249	4	Medium	0.156	0.00103371
**ND_L174S**		58	71	SKLREQLGPVTQEF	1630.8679	3.867	300	11	0.803	3.651	13.812	0.133	4	Medium	0.084	0.695044
**ND_L174S**		58	71	SKLREQLGPVTQEF	1630.8679	3.8784	3000	11	5.397	5.8	21.929	0.165	4	Medium	0.104	0.000579578
**ND_L174S**		58	71	SKLREQLGPVTQEF	1630.8679	3.867	9000	11	8.832	6.342	23.948	0.179	4	Medium	0.113	0.0113396
**ND_L174S**		61	71	REQLGPVTQEF	1302.6568	4.0088	0	8	0	0	0	n/a	2	High	0	
**ND_L174S**		61	71	REQLGPVTQEF	1302.6568	3.9692	30	8	0.062	1.629	10.179	0.256	2	High	0.029	0.00733904

**ND_L174S**		61	71	REQLGPVTQEF	1302.6568	3.9872	300	8	0.589	2.591	16.192	0.453		2	High	0.05	0.0361458
**ND_L174S**		61	71	REQLGPVTQEF	1302.6568	3.983	3000	8	3.963	4.038	25.236	0.068		2	High	0.008	0.00264872
**ND_L174S**		61	71	REQLGPVTQEF	1302.6568	3.9816	9000	8	6.444	4.318	26.987	0.247		2	High	0.028	0.0829387
**ND_L174S**		72	91	WDNLEKETEGLRQEMSKDLE	2449.1431	5.1303	0	18	0	0	0	n/a		2	High	0	
**ND_L174S**		72	91	WDNLEKETEGLRQEMSKDLE	2449.1431	5.0787	30	18	0.241	3.156	4.383	0.174		2	Medium	0.019	0.001731
**ND_L174S**		72	91	WDNLEKETEGLRQEMSKDLE	2449.1431	5.0897	300	18	2.187	5.036	6.994	0.35		2	Medium	0.039	1.17E-05
**ND_L174S**		72	91	WDNLEKETEGLRQEMSKDLE	2449.1431	5.0748	3000	18	11.683	9.169	12.735	1.255		2	Medium	0.14	0.000209773
**ND_L174S**		72	91	WDNLEKETEGLRQEMSKDLE	2449.1431	5.0497	9000	18	16.731	11.371	15.792	0.32		2	Medium	0.036	1.30E-05
**ND_L174S**		72	92	WDNLEKETEGLRQEMSKDLEE	2578.1857	5.2104	0	19	0	0	0	n/a		2	High	0	
**ND_L174S**		72	92	WDNLEKETEGLRQEMSKDLEE	2578.1857	5.171	30	19	0.261	2.85	5	0.246		2	High	0.027	0.00502009
**ND_L174S**		72	92	WDNLEKETEGLRQEMSKDLEE	2578.1857	5.1697	300	19	2.369	4.866	8.537	0.436		2	High	0.048	2.16E-05
**ND_L174S**		72	92	WDNLEKETEGLRQEMSKDLEE	2578.1857	5.1499	3000	19	12.549	8.103	14.216	0.911		2	High	0.101	0.000210966
**ND_L174S**		72	92	WDNLEKETEGLRQEMSKDLEE	2578.1857	5.1409	9000	19	17.729	9.198	16.137	0.84		2	High	0.094	8.92E-05
**ND_L174S**		72	103	WDNLEKETEGLRQEMSKDLEEVKAKVQPYLD	3834.8622	5.9539	0	29	0	0	0	n/a		2	Medium	0	
**ND_L174S**		72	103	WDNLEKETEGLRQEMSKDLEEVKAKVQPYLD	3834.8622	5.8988	30	29	0.4	2.1	1.81	0.201		2	Medium	0.022	0.0888409
**ND_L174S**		72	103	WDNLEKETEGLRQEMSKDLEEVKAKVQPYLD	3834.8622	5.9383	300	29	3.492	2.784	2.4	0.535		2	Medium	0.06	0.0529613
**ND_L174S**		72	103	WDNLEKETEGLRQEMSKDLEEVKAKVQPYLD	3834.8622	5.9079	3000	29	17.379	4.792	4.131	1.421		2	Medium	0.158	0.130869
**ND_L174S**		72	103	WDNLEKETEGLRQEMSKDLEEVKAKVQPYLD	3834.8622	5.9015	9000	29	25.476	7.52	6.483	0.628		2	Medium	0.07	0.051952
**ND_L174S**		77	92	KETEGLRQEMSKDLEE	1920.9099	3.4736	0	14	0	0	0	n/a		2	High	0	
**ND_L174S**		77	92	KETEGLRQEMSKDLEE	1920.9099	3.4156	30	14	0.173	3.315	11.838	1.106		2	High	0.123	0.0480096
**ND_L174S**		77	92	KETEGLRQEMSKDLEE	1920.9099	3.4313	300	14	1.615	5.062	18.077	0.462		2	High	0.051	0.00119617
**ND_L174S**		77	92	KETEGLRQEMSKDLEE	1920.9099	3.4419	3000	14	9.321	7.162	25.577	0.167		2	High	0.019	0.000341539
**ND_L174S**		77	92	KETEGLRQEMSKDLEE	1920.9099	3.4234	9000	14	13.171	8.139	29.069	0.178		2	High	0.02	0.00879743
**ND_L174S**		83	92	RQEMSKDLEE	1263.5765	2.2584	0	8	0	0	0	n/a		2	Medium	0	
**ND_L174S**		83	92	RQEMSKDLEE	1263.5765	2.2676	30	8	0.114	1.497	9.357	0.047		2	Medium	0.005	0.00185221
**ND_L174S**		83	92	RQEMSKDLEE	1263.5765	2.2723	300	8	1.052	2.247	14.041	0.207		2	Medium	0.023	0.00097177
**ND_L174S**		83	92	RQEMSKDLEE	1263.5765	2.2842	3000	8	5.659	3.511	21.943	0.096		2	Medium	0.011	4.78E-05
**ND_L174S**		83	92	RQEMSKDLEE	1263.5765	2.2647	9000	8	7.634	3.911	24.446	0.043		2	Medium	0.005	0.00494976
**ND_L174S**		87	92	SKDLEE	719.3337	1.722	0	4	0	0	0	n/a		2	Medium	0	
**ND_L174S**		87	92	SKDLEE	719.3337	1.6974	30	4	0.064	0.488	12.196	0.094		2	Medium	0.011	0.0102433
**ND_L174S**		87	92	SKDLEE	719.3337	1.7143	300	4	0.579	0.862	21.539	0.114		2	Medium	0.013	0.00558887
**ND_L174S**		87	92	SKDLEE	719.3337	1.7101	3000	4	2.821	1.613	40.336	0.556		2	Medium	0.062	0.00669847
**ND_L174S**		87	92	SKDLEE	719.3337	1.7309	9000	4	3.749	1.838	45.943	0.25		2	Medium	0.028	0.000949843
**ND_L174S**		90	103	LEEVKAKVQPYLDD	1645.8563	3.2852	0	11	0	0	0	n/a		2	High	0	
**ND_L174S**		90	103	LEEVKAKVQPYLDD	1645.8563	3.2245	30	11	0.159	1.303	5.923	0.759		2	High	0.084	0.0406672
**ND_L174S**		90	103	LEEVKAKVQPYLDD	1645.8563	3.2425	300	11	1.305	2.143	9.741	0.932		2	High	0.104	0.0224766
**ND_L174S**		90	103	LEEVKAKVQPYLDD	1645.8563	3.255	3000	11	5.696	4.136	18.8	1.104		2	High	0.123	0.0163347
**ND_L174S**		90	103	LEEVKAKVQPYLDD	1645.8563	3.2439	9000	11	8.744	5.16	23.455	1.266		2	High	0.141	0.0125038
**ND_L174S**		91	103	EEVKAKVQPYLDD	1532.7722	2.9777	0	10	0	0	0	n/a		2	High	0	
**ND_L174S**		91	103	EEVKAKVQPYLDD	1532.7722	2.9537	30	10	0.139	0.996	4.981	0.394		2	High	0.044	0.0407748
**ND_L174S**		91	103	EEVKAKVQPYLDD	1532.7722	2.9567	300	10	1.123	1.628	8.14	0.365		2	High	0.041	0.00535449
**ND_L174S**		91	103	EEVKAKVQPYLDD	1532.7722	2.986	3000	10	4.829	3.037	15.185	0.323		2	High	0.036	0.0140878
**ND_L174S**		91	103	EEVKAKVQPYLDD	1532.7722	2.9405	9000	10	7.747	4.477	22.385	0.724		2	Medium	0.081	0.00157305
**ND_L174S**		92	103	EVKAKVQPYLDD	1403.7296	2.892	0	9	0	0	0	n/a		2	High	0	
**ND_L174S**		92	103	EVKAKVQPYLDD	1403.7296	2.823	30	9	0.136	1.177	6.539	0.199		2	High	0.022	0.0499014
**ND_L174S**		92	103	EVKAKVQPYLDD	1403.7296	2.8426	300	9	1.091	1.943	10.796	0.661		2	High	0.074	0.00529249
**ND_L174S**		92	103	EVKAKVQPYLDD	1403.7296	2.8443	3000	9	4.55	3.626	20.147	0.857		2	High	0.095	0.0201567
**ND_L174S**		92	103	EVKAKVQPYLDD	1403.7296	2.8304	9000	9	7.122	4.35	24.165	0.467		2	High	0.052	0.000740958
**ND_L174S**		93	103	VKAKVQPYLDD	1274.6871	2.7313	0	8	0	0	0	n/a		2	High	0	
**ND_L174S**		93	103	VKAKVQPYLDD	1274.6871	2.6757	30	8	0.131	1.069	6.679	0.162		2	High	0.018	0.00892491
**ND_L174S**		93	103	VKAKVQPYLDD	1274.6871	2.6994	300	8	1.047	1.79	11.189	0.381		2	High	0.042	0.0220695
**ND_L174S**		93	103	VKAKVQPYLDD	1274.6871	2.7004	3000	8	4.191	3.291	20.571	0.921		2	High	0.102	0.0284551
**ND_L174S**		93	103	VKAKVQPYLDD	1274.6871	2.6952	9000	8	6.384	3.86	24.126	0.613		2	High	0.068	0.00214594
**ND_L174S**		93	104	VKAKVQPYLDDF	1421.7555	4.3494	0	9	0	0	0	n/a		2	High	0	
**ND_L174S**		93	104	VKAKVQPYLDDF	1421.7555	4.3227	30	9	0.144	0.718	3.989	0.617		2	High	0.069	0.0966399
**ND_L174S**		93	104	VKAKVQPYLDDF	1421.7555	4.3361	300	9	1.168	1.51	8.39	1.333		2	High	0.148	0.104873
**ND_L174S**		93	104	VKAKVQPYLDDF	1421.7555	4.3377	3000	9	4.915	3.243	18.015	0.05		2	Medium	0.006	0.0196916
**ND_L174S**		93	104	VKAKVQPYLDDF	1421.7555	4.3289	9000	9	7.363	3.8	21.113	1.285		2	High	0.143	0.0217496
**ND_L174S**		96	103	KVQPYLDD	976.4866	2.9535	0	5	0	0	0	n/a		2	Medium	0	
**ND_L174S**		96	103	KVQPYLDD	976.4866	2.8688	30	5	0.111	0.816	8.164	0.26		2	Medium	0.029	0.00883532
**ND_L174S**		96	103	KVQPYLDD	976.4866	2.9152	300	5	0.848	1.062	10.617	0.078		2	Medium	0.009	0.00790886
**ND_L174S**		96	103	KVQPYLDD	976.4866	2.9167	3000	5	2.803	1.692	16.92	0.064		2	Medium	0.007	0.0103021
**ND_L174S**		96	103	KVQPYLDD	976.4866	2.8986	9000	5	4.053	1.891	18.91	0.028		2	Medium	0.003	0.0150007
**ND_L174S**		104	111	FQKKWQEE	1121.5506	2.535	0	6	0	0	0	n/a		2	High	0	
**ND_L174S**		104	111	FQKKWQEE	1121.5506	2.4889	30	6	0.058	0.459	7.648	0.076		2	High	0.008	0.10964
**ND_L174S**		104	111	FQKKWQEE	1121.5506	2.5185	300	6	0.55	1.07	17.834	0.222		2	High	0.025	0.00172173
**ND_L174S**		104	111	FQKKWQEE	1121.5506	2.5324	3000	6	3.439	2.351	39.175	0.43		2	High	0.048	0.0184284
**ND_L174S**		104	111	FQKKWQEE	1121.5506	2.5092	9000	6	5.306	2.802	46.708	0.129		2	High	0.014	0.00622885
**ND_L174S**		113	124	ELYRQKVEPLRA	1500.8413	3.1799	0	9	0	0	0	n/a		2	High	0	
**ND_L174S**		113	124	ELYRQKVEPLRA	1500.8413	3.1616	30	9	0.06	2.67	9.888	0.204		2	High	0.023	0.000303686
**ND_L174S**		113	124	ELYRQKVEPLRA	1500.8413	3.1771	300	9	0.574	3.328	12.325	0.011		2	High	0.001	0.0142952

**ND_L174S**		113	124	ELYRQKVEPLRA	1500.8413	3.1752	3000	9	4.134	4.712	17.452	0.093	2	High	0.01	0.000402565
**ND_L174S**		113	124	ELYRQKVEPLRA	1500.8413	3.1654	9000	9	7.172	5.048	18.696	0.398	2	High	0.044	0.0656495
**ND_L174S**		113	126	ELYRQKVEPLRAEL	1742.9679	3.5205	0	11	0	0	0	n/a	2	Medium	0	
**ND_L174S**		113	126	ELYRQKVEPLRAEL	1742.9679	3.4795	30	11	0.074	3.219	9.753	1.072	2	Medium	0.119	0.0113915
**ND_L174S**		113	126	ELYRQKVEPLRAEL	1742.9679	3.4949	300	11	0.718	4.035	12.229	0.032	2	Medium	0.004	0.0333877
**ND_L174S**		113	126	ELYRQKVEPLRAEL	1742.9679	3.5238	3000	11	5.145	5.55	16.818	0.22	2	Medium	0.024	0.0102363
**ND_L174S**		113	126	ELYRQKVEPLRAEL	1742.9679	3.4916	9000	11	8.871	6.201	18.79	0.871	2	Medium	0.097	0.0556993
**ND_L174S**		114	124	LYRQKVEPLRA	1371.7987	2.4249	0	8	0	0	0	n/a	2	High	0	
**ND_L174S**		114	124	LYRQKVEPLRA	1371.7987	2.4226	30	8	0.055	2.797	11.656	0.663	2	High	0.074	0.0139558
**ND_L174S**		114	124	LYRQKVEPLRA	1371.7987	2.4489	300	8	0.529	3.248	13.533		1	High	0	
**ND_L174S**		114	124	LYRQKVEPLRA	1371.7987	2.443	3000	8	3.763	4.337	18.072	0.371	2	Medium	0.041	0.0274514
**ND_L174S**		114	124	LYRQKVEPLRA	1371.7987	2.4465	9000	8	6.42	4.661	19.422	0.088	2	High	0.01	0.192136
**ND_L174S**		114	125	LYRQKVEPLRAE	1500.8413	2.9962	0	9	0	0	0	n/a	2	Medium	0	
**ND_L174S**		114	125	LYRQKVEPLRAE	1500.8413	2.9645	30	9	0.065	2.741	10.151	0.051	2	Medium	0.006	0.00165911
**ND_L174S**		114	125	LYRQKVEPLRAE	1500.8413	2.9788	300	9	0.628	3.394	12.571	0.312	2	Medium	0.035	0.00178881
**ND_L174S**		114	125	LYRQKVEPLRAE	1500.8413	2.9927	3000	9	4.41	4.749	17.59	0.391	2	Medium	0.044	0.0219286
**ND_L174S**		114	125	LYRQKVEPLRAE	1500.8413	2.9846	9000	9	7.376	5.071	18.781	1.086	2	Medium	0.121	0.0896489
**ND_L174S**		114	126	LYRQKVEPLRAEL	1613.9253	3.36	0	10	0	0	0	n/a	2	High	0	
**ND_L174S**		114	126	LYRQKVEPLRAEL	1613.9253	3.3385	30	10	0.07	2.955	12.232	0.233	4	High	0.147	8.19E-06
**ND_L174S**		114	126	LYRQKVEPLRAEL	1613.9253	3.3497	300	10	0.672	3.768	15.671	0.064	4	High	0.04	3.91E-08
**ND_L174S**		114	126	LYRQKVEPLRAEL	1613.9253	3.3485	3000	10	4.773	5.365	22.334	0.057	4	High	0.036	0.000918374
**ND_L174S**		114	126	LYRQKVEPLRAEL	1613.9253	3.3427	9000	10	8.119	5.828	24.272	0.043	4	High	0.027	0.0636518
**ND_L174S**		115	124	YRQKVEPLRA	1258.7146	2.1006	0	7	0	0	0	n/a	2	High	0	
**ND_L174S**		115	124	YRQKVEPLRA	1258.7146	2.0859	30	7	0.048	2.539	12.09	0.222	2	High	0.025	0.00651802
**ND_L174S**		115	124	YRQKVEPLRA	1258.7146	2.1041	300	7	0.46	2.903	13.823	0.194	2	High	0.022	0.00318767
**ND_L174S**		115	124	YRQKVEPLRA	1258.7146	2.103	3000	7	3.251	3.845	18.308	0.016	2	High	0.002	0.00122379
**ND_L174S**		115	124	YRQKVEPLRA	1258.7146	2.1262	9000	7	5.537	4.013	19.111	0.365	2	High	0.041	0.0572468
**ND_L174S**		115	126	YRQKVEPLRAEL	1500.8413	3.1965	0	9	0	0	0	n/a	2	High	0	
**ND_L174S**		115	126	YRQKVEPLRAEL	1500.8413	3.1417	30	9	0.063	2.651	8.6	0.042	4	High	0.026	2.57E-08
**ND_L174S**		115	126	YRQKVEPLRAEL	1500.8413	3.1202	300	9	0.603	3.285	10.665	0.094	4	High	0.059	1.55E-08
**ND_L174S**		115	126	YRQKVEPLRAEL	1500.8413	3.1739	3000	9	4.262	4.673	15.162	0.073	4	High	0.046	2.49E-06
**ND_L174S**		115	126	YRQKVEPLRAEL	1500.8413	3.1641	9000	9	7.235	5.01	16.253	0.097	4	High	0.061	0.00161675
**ND_L174S**		115	147	YRQKVEPLRAELQEGARQKLHELQEKLSPLG	3901.081	4.5529	0	29	0	0	0	n/a	2	High	0	
**ND_L174S**		115	147	YRQKVEPLRAELQEGARQKLHELQEKLSPLG	3901.081	4.5208	30	29	0.313	6.942	4.788	0.311	2	High	0.035	0.0111726
**ND_L174S**		115	147	YRQKVEPLRAELQEGARQKLHELQEKLSPLG	3901.081	4.4987	300	29	2.883	9.631	6.642	0.902	2	High	0.1	0.00241168
**ND_L174S**		115	147	YRQKVEPLRAELQEGARQKLHELQEKLSPLG	3901.081	4.4995	3000	29	16.5	13.482	9.298	2.689	2	High	0.299	0.0333299
**ND_L174S**		115	147	YRQKVEPLRAELQEGARQKLHELQEKLSPLG	3901.081	4.4627	9000	29	24.896	16.115	11.114	2.974	2	Medium	0.331	0.284256
**ND_L174S**		115	158	YRQKVEPLRAELQEGARQKLHELQEKLSPLG	5179.7174	4.5482	0	40	0	0	0	n/a	2	High	0	
**ND_L174S**		115	158	YRQKVEPLRAELQEGARQKLHELQEKLSPLG	5179.7174	4.5067	30	40	0.506	8.249	2.946	0.96	2	High	0.107	0.00429998
**ND_L174S**		115	158	YRQKVEPLRAELQEGARQKLHELQEKLSPLG	5179.7174	4.513	300	40	4.628	12.055	4.305	0.787	2	High	0.088	0.000319555
**ND_L174S**		115	158	YRQKVEPLRAELQEGARQKLHELQEKLSPLG	5179.7174	4.4889	3000	40	24.825	19.404	6.93	0.21	2	High	0.023	0.230269
**ND_L174S**		115	158	YRQKVEPLRAELQEGARQKLHELQEKLSPLG	5179.7174	4.4924	9000	40	35.51	21.202	7.572	0.011	2	High	0.001	0.0591666
**ND_L174S**		125	136	ELQEGARQKLHE	1436.7372	1.9466	0	10	0	0	0	n/a	2	High	0	
**ND_L174S**		125	136	ELQEGARQKLHE	1436.7372	1.8887	30	10	0.157	2.97	14.85	0.009	2	High	0.001	0.00152099
**ND_L174S**		125	136	ELQEGARQKLHE	1436.7372	1.9042	300	10	1.395	3.583	17.916	0.577	2	High	0.064	0.00213317
**ND_L174S**		125	136	ELQEGARQKLHE	1436.7372	1.9241	3000	10	6.71	4.644	23.22	0.112	2	High	0.012	0.0234791
**ND_L174S**		125	136	ELQEGARQKLHE	1436.7372	1.8592	9000	10	9.173	5.403	27.016	0.897	2	High	0.1	0.665026
**ND_L174S**		125	139	ELQEGARQKLHELQE	1806.9224	2.9761	0	13	0	0	0	n/a	2	Medium	0	
**ND_L174S**		125	139	ELQEGARQKLHELQE	1806.9224	2.94	30	13	0.181	4.038	15.531	1.038	2	High	0.116	0.0021463
**ND_L174S**		125	139	ELQEGARQKLHELQE	1806.9224	2.9381	300	13	1.624	4.98	19.152	0.838	2	High	0.093	0.00778875
**ND_L174S**		125	139	ELQEGARQKLHELQE	1806.9224	2.9293	3000	13	8.264	6.68	25.692	0.434	2	High	0.048	0.0352385
**ND_L174S**		125	139	ELQEGARQKLHELQE	1806.9224	2.9123	9000	13	11.729	7.184	27.629	0.179	2	High	0.02	0.677497
**ND_L174S**		125	146	ELQEGARQKLHELQEKLSPLGE	2531.3344	4.3449	0	19	0	0	0	n/a	2	High	0	
**ND_L174S**		125	146	ELQEGARQKLHELQEKLSPLGE	2531.3344	4.3221	30	19	0.23	4.723	6.531	1.069	4	Medium	0.672	1.33E-05
**ND_L174S**		125	146	ELQEGARQKLHELQEKLSPLGE	2531.3344	4.2967	300	19	2.098	6.935	9.744	1.72	4	Medium	1.081	0.00744842
**ND_L174S**		125	146	ELQEGARQKLHELQEKLSPLGE	2531.3344	4.279	3000	19	11.371	9.264	12.8	1.062	4	Medium	0.668	0.0295822
**ND_L174S**		125	146	ELQEGARQKLHELQEKLSPLGE	2531.3344	4.2837	9000	19	16.663	10.222	14.441	0.546	4	Medium	0.343	0.278448
**ND_L174S**		125	148	ELQEGARQKLHELQEKLSPLGEEM	2791.4174	4.5737	0	21	0	0	0	n/a	2	Medium	0	
**ND_L174S**		125	148	ELQEGARQKLHELQEKLSPLGEEM	2791.4174	4.5509	30	21	0.262	5.296	7.378	0.417	4	Medium	0.262	1.71E-06
**ND_L174S**		125	148	ELQEGARQKLHELQEKLSPLGEEM	2791.4174	4.5366	300	21	2.393	7.848	10.982	1.003	4	High	0.631	0.000277036
**ND_L174S**		125	148	ELQEGARQKLHELQEKLSPLGEEM	2791.4174	4.5325	3000	21	12.937	10.616	14.755	0.563	4	Medium	0.354	0.0104987
**ND_L174S**		125	148	ELQEGARQKLHELQEKLSPLGEEM	2791.4174	4.5301	9000	21	18.634	11.155	15.527	0.471	4	High	0.296	0.221276
**ND_L174S**		125	158	ELQEGARQKLHELQEKLSPLGEEMRDRARAI	3939.0133	4.3194	0	31	0	0	0	n/a	2	High	0	
**ND_L174S**		125	158	ELQEGARQKLHELQEKLSPLGEEMRDRARAI	3939.0133	4.2759	30	31	0.443	6.786	4.936	0.239	4	High	0.15	1.60E-06
**ND_L174S**		125	158	ELQEGARQKLHELQEKLSPLGEEMRDRARAI	3939.0133	4.2701	300	31	4.025	10.006	7.226	1.587	4	High	0.998	0.013109
**ND_L174S**		125	158	ELQEGARQKLHELQEKLSPLGEEMRDRARAI	3939.0133	4.2529	3000	31	20.563	14.895	10.826	1.217	4	High	0.765	0.0788029
**ND_L174S**		125	158	ELQEGARQKLHELQEKLSPLGEEMRDRARAI	3939.0133	4.2599	9000	31	28.274	16.1	11.682	0.089	4	High	0.056	0.000306957

ND_L174S		125	159	ELQEGARQKLHELQEKLSPLGEEMRDRARAI	4052.0974	4.5064	0	32	0	0	0	n/a	2	Medium	0	
ND_L174S		125	159	ELQEGARQKLHELQEKLSPLGEEMRDRARAI	4052.0974	4.4797	30	32	0.447	7.572	4.733	1.146	2	High	0.128	0.00134224
ND_L174S		125	159	ELQEGARQKLHELQEKLSPLGEEMRDRARAI	4052.0974	4.4636	300	32	4.066	10.868	6.793	1.612	2	High	0.179	0.0134504
ND_L174S		125	159	ELQEGARQKLHELQEKLSPLGEEMRDRARAI	4052.0974	4.4693	3000	32	20.902	15.837	9.898	2.78	2	High	0.309	0.0756366
ND_L174S		125	159	ELQEGARQKLHELQEKLSPLGEEMRDRARAI	4052.0974	4.4781	9000	32	28.986	16.997	10.623	0.128	2	High	0.014	0.0330625
ND_L174S		126	158	LQEGARQKLHELQEKLSPLGEEMRDRARAH	3809.9707	4.3725	0	30	0	0	0	n/a	2	High	0	
ND_L174S		126	158	LQEGARQKLHELQEKLSPLGEEMRDRARAH	3809.9707	4.3277	30	30	0.437	6.898	4.598	1.282	2	High	0.143	0.00441145
ND_L174S		126	158	LQEGARQKLHELQEKLSPLGEEMRDRARAH	3809.9707	4.2986	300	30	3.968	10.385	6.923	2.541	2	High	0.283	0.0745045
ND_L174S		126	158	LQEGARQKLHELQEKLSPLGEEMRDRARAH	3809.9707	4.3068	3000	30	20.117	14.474	9.649	1.289	2	High	0.143	0.0204658
ND_L174S		126	158	LQEGARQKLHELQEKLSPLGEEMRDRARAH	3809.9707	4.3225	9000	30	27.444	15.437	10.291	0.638	2	High	0.071	0.013668
ND_L174S		127	139	QEGARQKLHELQE	1564.7958	2.4432	0	11	0	0	0	n/a	2	High	0	
ND_L174S		127	139	QEGARQKLHELQE	1564.7958	2.3992	30	11	0.161	3.324	15.108	0.059	2	High	0.007	0.00258762
ND_L174S		127	139	QEGARQKLHELQE	1564.7958	2.4037	300	11	1.44	3.909	17.768	0.314	2	High	0.035	0.00040181
ND_L174S		127	139	QEGARQKLHELQE	1564.7958	2.3868	3000	11	7.073	5.467	24.851	0.237	2	High	0.026	0.0717336
ND_L174S		127	139	QEGARQKLHELQE	1564.7958	2.3762	9000	11	9.916	5.753	26.149	0.112	2	High	0.012	0.173219
ND_L174S		127	146	QEGARQKLHELQEKLSPLGE	2289.2077	4.0826	0	17	0	0	0	n/a	2	High	0	
ND_L174S		127	146	QEGARQKLHELQEKLSPLGE	2289.2077	4.0273	30	17	0.211	4.828	9.468	0.193	2	High	0.021	0.000110127
ND_L174S		127	146	QEGARQKLHELQEKLSPLGE	2289.2077	4.0555	300	17	1.913	5.721	11.218	0.078	2	Medium	0.009	3.75E-05
ND_L174S		127	146	QEGARQKLHELQEKLSPLGE	2289.2077	4.0251	3000	17	10.181	7.991	15.668	0.756	2	Medium	0.084	0.00734049
ND_L174S		127	146	QEGARQKLHELQEKLSPLGE	2289.2077	3.998	9000	17	14.85	9.262	18.162	0.32	2	High	0.036	0.76406
ND_L174S		127	147	QEGARQKLHELQEKLSPLGEE	2418.2503	4.1217	0	18	0	0	0	n/a	2	High	0	
ND_L174S		127	147	QEGARQKLHELQEKLSPLGEE	2418.2503	4.0341	30	18	0.231	4.418	8.893	1.117	4	High	0.702	0.000407507
ND_L174S		127	147	QEGARQKLHELQEKLSPLGEE	2418.2503	4.0267	300	18	2.095	6.308	13.3	0.724	4	Medium	0.455	4.44E-05
ND_L174S		127	147	QEGARQKLHELQEKLSPLGEE	2418.2503	4.0182	3000	18	11.048	8.772	18.317	0.136	4	High	0.086	0.00502696
ND_L174S		127	147	QEGARQKLHELQEKLSPLGEE	2418.2503	3.9969	9000	18	15.847	9.392	19.639	0.297	4	High	0.187	0.0905431
ND_L174S		127	154	QEGARQKLHELQEKLSPLGEEMRDRARA	3274.6953	4.1239	0	25	0	0	0	n/a	2	High	0	
ND_L174S		127	154	QEGARQKLHELQEKLSPLGEEMRDRARA	3274.6953	4.0876	30	25	0.344	4.509	4.509	1.251	2	Medium	0.139	0.000420493
ND_L174S		127	154	QEGARQKLHELQEKLSPLGEEMRDRARA	3274.6953	4.0998	300	25	3.121	7.283	7.283	1.355	2	Medium	0.151	0.00147146
ND_L174S		127	154	QEGARQKLHELQEKLSPLGEEMRDRARA	3274.6953	4.0666	3000	25	16.178	12.632	12.632	2.405	2	High	0.268	0.94263
ND_L174S		127	154	QEGARQKLHELQEKLSPLGEEMRDRARA	3274.6953	4.0663	9000	25	22.604	13.351	13.351	0.918	2	High	0.102	0.141377
ND_L174S		127	158	QEGARQKLHELQEKLSPLGEEMRDRARAHV	3696.8867	4.1146	0	29	0	0	0	n/a	2	High	0	
ND_L174S		127	158	QEGARQKLHELQEKLSPLGEEMRDRARAHV	3696.8867	4.09	30	29	0.424	6.178	4.261	0.385	2	High	0.043	0.00464222
ND_L174S		127	158	QEGARQKLHELQEKLSPLGEEMRDRARAHV	3696.8867	4.0845	300	29	3.84	8.709	6.007	0.672	2	High	0.075	0.00261191
ND_L174S		127	158	QEGARQKLHELQEKLSPLGEEMRDRARAHV	3696.8867	4.0702	3000	29	19.373	13.27	9.152	0.989	2	High	0.11	0.0590849
ND_L174S		127	158	QEGARQKLHELQEKLSPLGEEMRDRARAHV	3696.8867	4.0582	9000	29	26.461	14.82	10.221	0.221	2	High	0.025	0.00857772
ND_L174S		127	159	QEGARQKLHELQEKLSPLGEEMRDRARAHV	3809.9707	4.3703	0	30	0	0	0	n/a	2	High	0	
ND_L174S		127	159	QEGARQKLHELQEKLSPLGEEMRDRARAHV	3809.9707	4.3341	30	30	0.428	6.682	5.033	0.476	4	High	0.299	3.02E-05
ND_L174S		127	159	QEGARQKLHELQEKLSPLGEEMRDRARAHV	3809.9707	4.3078	300	30	3.881	10.459	7.864	0.442	4	High	0.278	0.00250761
ND_L174S		127	159	QEGARQKLHELQEKLSPLGEEMRDRARAHV	3809.9707	4.3171	3000	30	19.712	14.421	10.874	1.33	4	High	0.836	0.0327084
ND_L174S		127	159	QEGARQKLHELQEKLSPLGEEMRDRARAHV	3809.9707	4.3302	9000	30	27.172	15.57	11.688	0.27	4	High	0.17	2.88E-06
ND_L174S		137	146	LQEKLSPLGE	1112.6077	3.4214	0	7	0	0	0	n/a	2	High	0	
ND_L174S		137	146	LQEKLSPLGE	1112.6077	3.3928	30	7	0.063	1.778	12.699	0.246	2	High	0.027	0.00626198
ND_L174S		137	146	LQEKLSPLGE	1112.6077	3.4281	300	7	0.601	2.275	16.252	0.235	2	High	0.026	0.00313584
ND_L174S		137	146	LQEKLSPLGE	1112.6077	3.4087	3000	7	3.852	3.212	22.945	0.13	2	Medium	0.014	0.00495097
ND_L174S		137	146	LQEKLSPLGE	1112.6077	3.3448	9000	7	5.917	3.465	24.752	0.897	2	Medium	0.1	0.465848
ND_L174S		137	147	LQEKLSPLGEE	1241.6503	3.4557	0	8	0	0	0	n/a	2	Medium	0	
ND_L174S		137	147	LQEKLSPLGEE	1241.6503	3.4258	30	8	0.083	1.984	12.398	0.269	2	Medium	0.03	0.00556493
ND_L174S		137	147	LQEKLSPLGEE	1241.6503	3.4449	300	8	0.783	2.606	16.288	0.21	2	Medium	0.023	0.00039579
ND_L174S		137	147	LQEKLSPLGEE	1241.6503	3.4004	3000	8	4.718	3.759	23.492	0.252	2	Medium	0.028	0.0332017
ND_L174S		137	147	LQEKLSPLGEE	1241.6503	3.3895	9000	8	6.915	3.853	24.082	0.25	2	Medium	0.028	0.104091
ND_L174S		137	158	LQEKLSPLGEEMRDRARAHVDA	2520.2867	3.3349	0	19	0	0	0	n/a	2	High	0	
ND_L174S		137	158	LQEKLSPLGEEMRDRARAHVDA	2520.2867	3.3031	30	19	0.276	3.862	5.419	0.037	4	High	0.023	1.64E-06
ND_L174S		137	158	LQEKLSPLGEEMRDRARAHVDA	2520.2867	3.3173	300	19	2.528	5.431	7.61	0.22	4	High	0.138	0.00515227
ND_L174S		137	158	LQEKLSPLGEEMRDRARAHVDA	2520.2867	3.3221	3000	19	13.043	8.278	11.702	0.804	4	High	0.505	0.0232147
ND_L174S		137	158	LQEKLSPLGEEMRDRARAHVDA	2520.2867	3.3014	9000	19	17.528	9.389	13.194	0.108	4	High	0.068	7.46E-08
ND_L174S		137	159	LQEKLSPLGEEMRDRARAHVDAL	2633.3708	3.8295	0	20	0	0	0	n/a	2	High	0	
ND_L174S		137	159	LQEKLSPLGEEMRDRARAHVDAL	2633.3708	3.801	30	20	0.28	4.266	4.266	1.165	2	High	0.13	0.00617096
ND_L174S		137	159	LQEKLSPLGEEMRDRARAHVDAL	2633.3708	3.8176	300	20	2.569	5.853	5.853	0.388	2	High	0.043	0.146858
ND_L174S		137	159	LQEKLSPLGEEMRDRARAHVDAL	2633.3708	3.8053	3000	20	13.382	9.399	9.399	1.073	2	Medium	0.119	0.00305043
ND_L174S		137	159	LQEKLSPLGEEMRDRARAHVDAL	2633.3708	3.8437	9000	20	18.24	9.923	9.923	1.111	2	Medium	0.124	0.026912
ND_L174S		139	158	EKLSPLGEEMRDRARAHVDA	2279.1441	3.1981	0	17	0	0	0	n/a	2	Medium	0	
ND_L174S		139	158	EKLSPLGEEMRDRARAHVDA	2279.1441	3.1706	30	17	0.251	3.537	5.201	0.123	2	Medium	0.014	0.0288951
ND_L174S		139	158	EKLSPLGEEMRDRARAHVDA	2279.1441	3.1388	300	17	2.292	5.821	8.56	0.088	2	Medium	0.01	0.00274187
ND_L174S		139	158	EKLSPLGEEMRDRARAHVDA	2279.1441	3.1726	3000	17	11.614	8.263	12.152	0.539	2	Medium	0.06	0.00376978
ND_L174S		139	158	EKLSPLGEEMRDRARAHVDA	2279.1441	3.1592	9000	17	15.576	8.934	13.139	0.283	2	Medium	0.032	0.00782457
ND_L174S		140	146	KLSPLGE	742.4225	2.9013	0	4	0	0	0	n/a	2	Medium	0	
ND_L174S		140	146	KLSPLGE	742.4225	2.8474	30	4	0.035	1.093	27.326	0.258	2	Medium	0.029	0.00298301
ND_L174S		140	146	KLSPLGE	742.4225	2.8896	300	4	0.334	1.372	34.301	0.177	2	Medium	0.02	0.0166245
ND_L174S		140	146	KLSPLGE	742.4225	2.8975	3000	4	2.158	1.829	45.722	0.015	2	Medium	0.002	0.785833
ND_L174S		140	146	KLSPLGE	742.4225	2.8825	9000	4	3.363	1.806	45.138	0.138	2	Medium	0.015	0.261575

ND_L174S		140	147	KLSPLGEE	871.4651	3.0038	0	5	0	0	0	n/a	2	Medium	0	
ND_L174S		140	147	KLSPLGEE	871.4651	2.9857	30	5	0.055	1.329	13.286	0.027	2	Medium	0.003	0.0252383
ND_L174S		140	147	KLSPLGEE	871.4651	3.0013	300	5	0.516	1.692	16.924	0.038	2	Medium	0.004	0.0149977
ND_L174S		140	147	KLSPLGEE	871.4651	3.008	3000	5	3.025	2.263	22.629	0.266	2	Medium	0.03	0.908754
ND_L174S		140	147	KLSPLGEE	871.4651	2.9946	9000	5	4.361	2.254	22.535	0.039	2	Medium	0.004	0.953192
ND_L174S		140	148	KLSPLGEEM	1002.5056	3.788	0	6	0	0	0	n/a	2	Medium	0	
ND_L174S		140	148	KLSPLGEEM	1002.5056	3.7258	30	6	0.067	1.291	10.758	0.423	2	Medium	0.047	0.000838424
ND_L174S		140	148	KLSPLGEEM	1002.5056	3.7741	300	6	0.629	1.761	14.673	0.474	2	Medium	0.053	0.0188852
ND_L174S		140	148	KLSPLGEEM	1002.5056	3.7978	3000	6	3.723	2.616	21.802	0.077	2	Medium	0.009	0.210571
ND_L174S		140	148	KLSPLGEEM	1002.5056	3.713	9000	6	5.333	2.53	21.082	0.41	2	Medium	0.046	0.0331402
ND_L174S		147	158	EMRDRARAHVDA	1425.6895	1.438	0	10	0	0	0	n/a	2	High	0	
ND_L174S		147	158	EMRDRARAHVDA	1425.6895	1.4461	30	10	0.181	1.871	4.678	0.351	2	High	0.039	0.00354814
ND_L174S		147	158	EMRDRARAHVDA	1425.6895	1.4328	300	10	1.632	2.682	6.706	0.206	2	Medium	0.023	0.000218261
ND_L174S		147	158	EMRDRARAHVDA	1425.6895	1.4399	3000	10	7.626	4.101	10.253	0.589	2	Medium	0.066	0.00268271
ND_L174S		147	158	EMRDRARAHVDA	1425.6895	1.3871	9000	10	9.641	4.866	12.165	0.56	2	Medium	0.062	0.000798529
ND_L174S		147	159	EMRDRARAHVDAL	1538.7736	2.3307	0	11	0	0	0	n/a	2	Medium	0	
ND_L174S		147	159	EMRDRARAHVDAL	1538.7736	2.312	30	11	0.185	1.747	4.488	0.714	4	Medium	0.449	0.0310022
ND_L174S		147	159	EMRDRARAHVDAL	1538.7736	2.3185	300	11	1.672	2.613	6.972	0.352	4	Medium	0.221	5.60E-05
ND_L174S		147	159	EMRDRARAHVDAL	1538.7736	2.2981	3000	11	7.965	4.367	11.569	0.104	4	Medium	0.065	7.34E-07
ND_L174S		147	159	EMRDRARAHVDAL	1538.7736	2.3058	9000	11	10.352	4.989	13.215	0.093	4	Medium	0.058	8.05E-07
ND_L174S		147	169	EMRDRARAHVDALRTHLAPYSDE	2708.3201	3.134	0	20	0	0	0	n/a	2	High	0	
ND_L174S		147	169	EMRDRARAHVDALRTHLAPYSDE	2708.3201	3.0897	30	20	0.353	4.646	5.807	0.08	2	Medium	0.009	0.00720786
ND_L174S		147	169	EMRDRARAHVDALRTHLAPYSDE	2708.3201	3.0987	300	20	3.141	5.983	7.479	1.197	2	High	0.133	0.00134417
ND_L174S		147	169	EMRDRARAHVDALRTHLAPYSDE	2708.3201	3.0838	3000	20	14.157	8.875	11.093	0.187	2	High	0.021	0.00835215
ND_L174S		147	169	EMRDRARAHVDALRTHLAPYSDE	2708.3201	3.0651	9000	20	18.62	9.705	12.132	0.547	2	High	0.061	0.000380112
ND_L174S		148	158	MRDRARAHVDA	1296.6469	1.4181	0	9	0	0	0	n/a	2	High	0	
ND_L174S		148	158	MRDRARAHVDA	1296.6469	1.4141	30	9	0.173	1.501	4.168	0.228	2	High	0.025	0.00198782
ND_L174S		148	158	MRDRARAHVDA	1296.6469	1.4045	300	9	1.552	2.047	5.685	0.553	2	High	0.062	0.00920278
ND_L174S		148	158	MRDRARAHVDA	1296.6469	1.4303	3000	9	7.061	3.282	9.117	0.051	2	High	0.006	2.86E-05
ND_L174S		148	158	MRDRARAHVDA	1296.6469	1.436	9000	9	8.723	3.714	10.315	0.129	2	High	0.014	0.000758909
ND_L174S		148	159	MRDRARAHVDAL	1409.731	2.0508	0	10	0	0	0	n/a	2	Medium	0	
ND_L174S		148	159	MRDRARAHVDAL	1409.731	2.0322	30	10	0.177	1.638	4.095	0.392	2	Medium	0.044	0.00398239
ND_L174S		148	159	MRDRARAHVDAL	1409.731	2.0354	300	10	1.593	2.185	5.463	0.508	2	Medium	0.056	0.0150866
ND_L174S		148	159	MRDRARAHVDAL	1409.731	2.0454	3000	10	7.4	3.48	8.7	0.285	2	Medium	0.032	0.000365526
ND_L174S		148	159	MRDRARAHVDAL	1409.731	2.0417	9000	10	9.434	4.025	10.063	0.691	2	Medium	0.077	0.00182519
ND_L174S		148	169	MRDRARAHVDALRTHLAPYSDE	2579.2775	2.8921	0	19	0	0	0	n/a	2	High	0	
ND_L174S		148	169	MRDRARAHVDALRTHLAPYSDE	2579.2775	2.869	30	19	0.345	4.269	5.618	0.645	2	High	0.072	0.000124414
ND_L174S		148	169	MRDRARAHVDALRTHLAPYSDE	2579.2775	2.8744	300	19	3.061	5.363	7.056	0.678	2	High	0.075	5.07E-06
ND_L174S		148	169	MRDRARAHVDALRTHLAPYSDE	2579.2775	2.8742	3000	19	13.592	7.177	9.443	0.056	2	High	0.006	9.20E-05
ND_L174S		148	169	MRDRARAHVDALRTHLAPYSDE	2579.2775	2.8576	9000	19	17.702	7.957	10.469	0.449	2	High	0.05	0.0036953
ND_L174S		149	158	RDRARAHVDA	1165.6065	1.4268	0	8	0	0	0	n/a	2	Medium	0	
ND_L174S		149	158	RDRARAHVDA	1165.6065	1.4483	30	8	0.136	1.422	5.924	0.648	2	Medium	0.072	0.0584953
ND_L174S		149	158	RDRARAHVDA	1165.6065	1.4176	300	8	1.24	1.85	7.707	0.547	2	Medium	0.061	0.0254553
ND_L174S		149	158	RDRARAHVDA	1165.6065	1.4111	3000	8	6.085	2.994	12.473	0.243	2	Medium	0.027	0.000497711
ND_L174S		149	158	RDRARAHVDA	1165.6065	1.4111	9000	8	7.723	3.432	14.298	0.378	2	Medium	0.042	0.00272573
ND_L174S		155	169	HVDALRTHLAPYSDE	1722.8325	3.0653	0	12	0	0	0	n/a	2	Medium	0	
ND_L174S		155	169	HVDALRTHLAPYSDE	1722.8325	3.0386	30	12	0.216	4.062	11.284	0.09	2	Medium	0.01	0.00528016
ND_L174S		155	169	HVDALRTHLAPYSDE	1722.8325	3.0647	300	12	1.907	5.234	14.539	0.756	2	Medium	0.084	0.00485087
ND_L174S		155	169	HVDALRTHLAPYSDE	1722.8325	3.0405	3000	12	8.293	5.034	13.982	1.074	2	Medium	0.12	0.0162317
ND_L174S		155	169	HVDALRTHLAPYSDE	1722.8325	3.036	9000	12	10.974	5.167	14.352	0.214	2	Medium	0.024	0.00314342
ND_L174S		155	170	HVDALRTHLAPYSDEL	1835.9166	3.7833	0	13	0	0	0	n/a	2	Medium	0	
ND_L174S		155	170	HVDALRTHLAPYSDEL	1835.9166	3.7672	30	13	0.221	4.699	12.049	0.28	2	Medium	0.031	0.00177821
ND_L174S		155	170	HVDALRTHLAPYSDEL	1835.9166	3.7862	300	13	1.951	4.813	12.341	0.195	2	Medium	0.022	0.000268013
ND_L174S		155	170	HVDALRTHLAPYSDEL	1835.9166	3.8196	3000	13	8.657	5.63	14.436	0.481	2	Medium	0.054	0.00221992
ND_L174S		155	170	HVDALRTHLAPYSDEL	1835.9166	3.7969	9000	13	11.716	5.75	14.745	0.101	2	Medium	0.011	6.76E-05
ND_L174S		159	168	LRTHLAPYSD	1171.5986	2.6683	0	7	0	0	0	n/a	2	Medium	0	
ND_L174S		159	168	LRTHLAPYSD	1171.5986	2.6379	30	7	0.131	2.669	19.066	0.032	2	Medium	0.004	0.00231609
ND_L174S		159	168	LRTHLAPYSD	1171.5986	2.6666	300	7	1.143	2.682	19.156	0.059	2	Medium	0.007	0.00790009
ND_L174S		159	168	LRTHLAPYSD	1171.5986	2.6678	3000	7	4.818	2.886	20.611	0.161	2	Medium	0.018	0.000803909
ND_L174S		159	168	LRTHLAPYSD	1171.5986	2.6496	9000	7	6.467	2.893	20.666	0.013	2	Medium	0.001	0.0398747
ND_L174S		159	169	LRTHLAPYSDE	1300.6412	2.8277	0	8	0	0	0	n/a	2	High	0	
ND_L174S		159	169	LRTHLAPYSDE	1300.6412	2.7029	30	8	0.163	2.719	16.992	0.076	2	High	0.008	0.0018454
ND_L174S		159	169	LRTHLAPYSDE	1300.6412	2.7177	300	8	1.416	2.829	17.682	0.145	2	High	0.016	0.000252232
ND_L174S		159	169	LRTHLAPYSDE	1300.6412	2.7253	3000	8	5.777	3.132	19.576	0.339	2	High	0.038	0.00798967
ND_L174S		159	169	LRTHLAPYSDE	1300.6412	2.7161	9000	8	7.467	3.119	19.493	0.039	2	High	0.004	0.00732405
ND_L174S		159	170	LRTHLAPYSDEL	1413.7252	3.7141	0	9	0	0	0	n/a	2	High	0	
ND_L174S		159	170	LRTHLAPYSDEL	1413.7252	3.6586	30	9	0.167	3.101	17.225	0.022	2	High	0.002	0.00585632

**ND_L174S**		159	170	LRTHLAPYSDEL	1413.7252	3.6721	300	9	1.46	3.287	18.264	0.177	2	High	0.02	0.000282357
**ND_L174S**		159	170	LRTHLAPYSDEL	1413.7252	3.6755	3000	9	6.14	3.696	20.532	0.446	2	High	0.05	0.00984763
**ND_L174S**		159	170	LRTHLAPYSDEL	1413.7252	3.6739	9000	9	8.21	3.686	20.476	0.074	2	High	0.008	0.00542629
**ND_L174S**		159	173	LRTHLAPYSDELRQR	1853.986	2.7988	0	12	0	0	0	n/a	2	Medium	0	
**ND_L174S**		159	173	LRTHLAPYSDELRQR	1853.986	2.7853	30	12	0.193	5.789	12.061	1.273	2	Medium	0.142	0.00213407
**ND_L174S**		159	173	LRTHLAPYSDELRQR	1853.986	2.7664	300	12	1.705	5.853	12.193	1.106	2	Medium	0.123	0.0106781
**ND_L174S**		159	173	LRTHLAPYSDELRQR	1853.986	2.7861	3000	12	7.827	5.707	11.89	0.294	2	Medium	0.033	0.000864139
**ND_L174S**		159	173	LRTHLAPYSDELRQR	1853.986	2.7898	9000	12	10.913	5.633	11.736	1.169	2	Medium	0.13	0.0162979
**ND_L174S**		159	174	LRTHLAPYSDELRQRS	1941.0181	0	0	13	0	0	0	n/a	2	High	0	
**ND_L174S**		159	174	LRTHLAPYSDELRQRS	1941.0181	0	30	13	0.212							
**ND_L174S**		159	174	LRTHLAPYSDELRQRS	1941.0181	0	300	13	1.88							
**ND_L174S**		159	174	LRTHLAPYSDELRQRS	1941.0181	0	3000	13	8.681							
**ND_L174S**		159	174	LRTHLAPYSDELRQRS	1941.0181	0	9000	13	11.91							
**ND_L174S**		159	176	LRTHLAPYSDELRQRSAA	2083.0923	0	0	15	0	0	0	n/a	2	High	0	
**ND_L174S**		159	176	LRTHLAPYSDELRQRSAA	2083.0923	0	30	15	0.24							
**ND_L174S**		159	176	LRTHLAPYSDELRQRSAA	2083.0923	0	300	15	2.148							
**ND_L174S**		159	176	LRTHLAPYSDELRQRSAA	2083.0923	0	3000	15	10.204							
**ND_L174S**		159	176	LRTHLAPYSDELRQRSAA	2083.0923	0	9000	15	13.882							
**ND_L174S**		159	178	LRTHLAPYSDELRQRSAARL	2352.2775	0	0	17	0	0	0	n/a	2	High	0	
**ND_L174S**		159	178	LRTHLAPYSDELRQRSAARL	2352.2775	0	30	17	0.252							
**ND_L174S**		159	178	LRTHLAPYSDELRQRSAARL	2352.2775	0	300	17	2.259							
**ND_L174S**		159	178	LRTHLAPYSDELRQRSAARL	2352.2775	0	3000	17	11.048							
**ND_L174S**		159	178	LRTHLAPYSDELRQRSAARL	2352.2775	0	9000	17	15.432							
**ND_L174S**		159	180	LRTHLAPYSDELRQRSAARLEA	2552.3572	3.0883	0	19	0	0	0	n/a	2	High	0	
**ND_L174S**		159	180	LRTHLAPYSDELRQRSAARLEA	2552.3572	3.0929	30	19	0.276	7.904	10.401	1.615	2	High	0.18	0.00785771
**ND_L174S**		159	180	LRTHLAPYSDELRQRSAARLEA	2552.3572	3.1185	300	19	2.48	8.598	11.313	0.569	2	High	0.063	3.49E-06
**ND_L174S**		159	180	LRTHLAPYSDELRQRSAARLEA	2552.3572	3.1167	3000	19	12.355	9.7	12.763	0.748	2	High	0.083	5.35E-07
**ND_L174S**		159	180	LRTHLAPYSDELRQRSAARLEA	2552.3572	3.1097	9000	19	17.294	9.698	12.76	0.581	2	High	0.065	4.83E-05
**ND_L174S**		160	169	RTHLAPYSDE	1187.5571	2.3931	0	7	0	0	0	n/a	2	High	0	
**ND_L174S**		160	169	RTHLAPYSDE	1187.5571	2.3643	30	7	0.156	2.454	17.529	0.035	2	High	0.004	4.98E-06
**ND_L174S**		160	169	RTHLAPYSDE	1187.5571	2.3842	300	7	1.347	2.471	17.648	0.04	2	High	0.004	0.000540113
**ND_L174S**		160	169	RTHLAPYSDE	1187.5571	2.3753	3000	7	5.264	2.759	19.706	0.321	2	High	0.036	0.00163363
**ND_L174S**		160	169	RTHLAPYSDE	1187.5571	2.3714	9000	7	6.583	2.751	19.651	0.325	2	Medium	0.036	0.00106202
**ND_L174S**		160	174	RTHLAPYSDELRQRS	1827.934	0	0	12	0	0	0	n/a	2	High	0	
**ND_L174S**		160	174	RTHLAPYSDELRQRS	1827.934	0	30	12	0.205							
**ND_L174S**		160	174	RTHLAPYSDELRQRS	1827.934	0	300	12	1.81							
**ND_L174S**		160	174	RTHLAPYSDELRQRS	1827.934	0	3000	12	8.168							
**ND_L174S**		160	174	RTHLAPYSDELRQRS	1827.934	0	9000	12	11.026							
**ND_L174S**		170	176	LRQRSAA	800.4617	0	0	5	0	0	0	n/a	2	High	0	
**ND_L174S**		170	176	LRQRSAA	800.4617	0	30	5	0.068							
**ND_L174S**		170	176	LRQRSAA	800.4617	0	300	5	0.635							
**ND_L174S**		170	176	LRQRSAA	800.4617	0	3000	5	3.649							
**ND_L174S**		170	176	LRQRSAA	800.4617	0	9000	5	4.872							
**ND_L174S**		170	177	LRQRSAAR	956.5628	1.6615	0	6	0	0	0	n/a	2	High	0	
**ND_L174S**		170	177	LRQRSAAR	956.5628	1.6254	30	6	0.076	3.234	17.969	0.243	2	Medium	0.027	0.000424476
**ND_L174S**		170	177	LRQRSAAR	956.5628	1.6325	300	6	0.713	3.086	17.146	0.085	2	High	0.009	0.000267749
**ND_L174S**		170	177	LRQRSAAR	956.5628	1.6319	3000	6	4.205	3.36	18.666	0.284	2	Medium	0.032	0.00471935
**ND_L174S**		170	177	LRQRSAAR	956.5628	1.6405	9000	6	5.785	3.404	18.91	0.249	2	Medium	0.028	0.00199131
**ND_L174S**		170	178	LRQRSAARL	1069.6469	2.3492	0	7	0	0	0	n/a	2	Medium	0	
**ND_L174S**		170	178	LRQRSAARL	1069.6469	1.626	30	7	0.079	3.942	18.773	0.167	2	High	0.019	7.52E-05
**ND_L174S**		170	178	LRQRSAARL	1069.6469	1.6464	300	7	0.746	3.839	18.281	0.058	2	High	0.006	3.33E-05
**ND_L174S**		170	178	LRQRSAARL	1069.6469	1.6473	3000	7	4.492	4.191	19.958	0.314	2	High	0.035	0.00368022
**ND_L174S**		170	178	LRQRSAARL	1069.6469	1.6435	9000	7	6.422	4.163	19.825	0.223	2	High	0.025	0.0012757
**ND_L174S**		170	180	LRQRSAARLEA	1269.7266	2.1677	0	9	0	0	0	n/a	2	Medium	0	
**ND_L174S**		170	180	LRQRSAARLEA	1269.7266	1.7609	30	9	0.103	4.324	16.015	0.222	2	Medium	0.025	6.47E-06
**ND_L174S**		170	180	LRQRSAARLEA	1269.7266	1.7991	300	9	0.968	4.34	16.074	0.013	2	Medium	0.001	3.08E-08
**ND_L174S**		170	180	LRQRSAARLEA	1269.7266	1.7928	3000	9	5.8	4.831	17.893	0.543	2	Medium	0.06	0.00295539
**ND_L174S**		170	180	LRQRSAARLEA	1269.7266	1.7656	9000	9	8.284	4.855	17.982	0.319	2	Medium	0.035	0.000315659
**ND_L174S**		171	178	RQRSAARL	956.5628	2.1627	0	6	0	0	0	n/a	2	Medium	0	
**ND_L174S**		171	178	RQRSAARL	956.5628	2.1118	30	6	0.069	3.413	18.964	0.312	2	Medium	0.035	0.00123687
**ND_L174S**		171	178	RQRSAARL	956.5628	2.1473	300	6	0.649	3.239	17.995	0.153	2	Medium	0.017	0.00163025
**ND_L174S**		171	178	RQRSAARL	956.5628	2.1414	3000	6	3.85	3.491	19.393	0.066	2	Medium	0.007	0.000168124
**ND_L174S**		171	178	RQRSAARL	956.5628	2.1502	9000	6	5.468	3.423	19.015	0.13	2	Medium	0.014	0.000111432
**ND_L174S**		171	180	RQRSAARLEA	1156.6425	2.4753	0	8	0	0	0	n/a	2	High	0	
**ND_L174S**		171	180	RQRSAARLEA	1156.6425	2.4521	30	8	0.093	4.226	17.609	0.072	2	High	0.008	0.00163652
**ND_L174S**		171	180	RQRSAARLEA	1156.6425	2.4711	300	8	0.87	4.291	17.878	0.071	2	High	0.008	0.00442836
**ND_L174S**		171	180	RQRSAARLEA	1156.6425	2.4728	3000	8	5.158	4.726	19.694	0.459	2	High	0.051	0.00342652
**ND_L174S**		171	180	RQRSAARLEA	1156.6425	2.4658	9000	8	7.33	4.687	19.527	0.082	2	High	0.009	0.00239611
**ND_L174S**		179	189	EALKENGGARL	1156.62	2.2336	0	9	0	0	0	n/a	2	High	0	
**ND_L174S**		179	189	EALKENGGARL	1156.62	1.994	30	9	0.121	4.236	23.532	0.831	2	Medium	0.093	0.0471118
**ND_L174S**		179	189	EALKENGGARL	1156.62	1.9905	300	9	1.106	3.956	21.978	0.507	2	High	0.056	0.0407311

**ND_L174S**		179	189	EALKENGGARL	1156.62	2.0019	3000	9	5.692	4.168	23.156	0.465	2	Medium	0.052	0.0381654
**ND_L174S**		179	189	EALKENGGARL	1156.62	1.9775	9000	9	7.965	4.13	22.946	0.1	2	Medium	0.011	0.0493916
**ND_L174S**		179	191	EALKENGGARLAE	1356.6997	2.2533	0	11	0	0	0	n/a	2	Medium	0	
**ND_L174S**		179	191	EALKENGGARLAE	1356.6997	2.2209	30	11	0.142	5.694	17.254	0.335	2	High	0.037	0.848088
**ND_L174S**		179	191	EALKENGGARLAE	1356.6997	2.231	300	11	1.308	5.462	16.551	0.989	2	High	0.11	0.0861414
**ND_L174S**		179	191	EALKENGGARLAE	1356.6997	2.2345	3000	11	7.002	5.827	17.657	0.651	2	High	0.073	0.639466
**ND_L174S**		179	191	EALKENGGARLAE	1356.6997	2.2319	9000	11	9.883	5.918	17.935	0.097	2	High	0.011	0.154717
**ND_L174S**		179	203	EALKENGGARLAEYHAKATEHLSTL	2708.3882	3.3035	0	23	0	0	0	n/a	2	High	0	
**ND_L174S**		179	203	EALKENGGARLAEYHAKATEHLSTL	2708.3882	3.2204	30	23	0.348	9.74	14.115	0.629	2	Medium	0.07	0.00113994
**ND_L174S**		179	203	EALKENGGARLAEYHAKATEHLSTL	2708.3882	3.2593	300	23	3.083	10.337	14.981	0.048	2	High	0.005	0.0381833
**ND_L174S**		179	203	EALKENGGARLAEYHAKATEHLSTL	2708.3882	3.2588	3000	23	14.85	11.732	17.002	1.259	2	High	0.14	0.633416
**ND_L174S**		179	203	EALKENGGARLAEYHAKATEHLSTL	2708.3882	3.2489	9000	23	20.808	11.686	16.936	0.36	2	High	0.04	0.159491
**ND_L174S**		181	190	LKENGGARLA	1027.5774	1.8968	0	8	0	0	0	n/a	2	Medium	0	
**ND_L174S**		181	190	LKENGGARLA	1027.5774	1.8966	30	8	0.123	4.199	21.855	0.166	4	Medium	0.104	0.233886
**ND_L174S**		181	190	LKENGGARLA	1027.5774	1.9247	300	8	1.122	3.637	18.919	0.058	4	Medium	0.036	0.0333387
**ND_L174S**		181	190	LKENGGARLA	1027.5774	1.9202	3000	8	5.638	3.764	19.623	0.089	4	Medium	0.056	0.208336
**ND_L174S**		181	190	LKENGGARLA	1027.5774	1.9192	9000	8	7.456	3.712	19.301	0.094	4	Medium	0.059	0.0437074
**ND_L174S**		181	191	LKENGGARLAE	1156.62	2.0104	0	9	0	0	0	n/a	2	High	0	
**ND_L174S**		181	191	LKENGGARLAE	1156.62	1.9768	30	9	0.133	4.581	16.965	0.181	2	Medium	0.02	0.105536
**ND_L174S**		181	191	LKENGGARLAE	1156.62	1.9788	300	9	1.221	4.203	15.566	0.601	2	Medium	0.067	0.0641516
**ND_L174S**		181	191	LKENGGARLAE	1156.62	1.9883	3000	9	6.285	4.402	16.303	0.25	2	Medium	0.028	0.0796527
**ND_L174S**		181	191	LKENGGARLAE	1156.62	1.9817	9000	9	8.412	4.323	16.01	0.031	2	Medium	0.003	0.0791894
**ND_L174S**		190	195	AEYHAK	717.3446	1.3713	0	4	0	0	0	n/a	2	Medium	0	
**ND_L174S**		190	195	AEYHAK	717.3446	1.399	30	4	0.08	2.137	26.714	0.558	2	Medium	0.062	0.16112
**ND_L174S**		190	195	AEYHAK	717.3446	1.379	300	4	0.707	2.021	25.265	1.022	2	Medium	0.114	0.263054
**ND_L174S**		190	195	AEYHAK	717.3446	1.4088	3000	4	2.992	2.052	25.646	0.783	2	Medium	0.087	0.228809
**ND_L174S**		190	195	AEYHAK	717.3446	1.3963	9000	4	3.795	2.055	25.689	0.631	2	Medium	0.07	0.412484
**ND_L174S**		190	200	AEYHAKATEHL	1268.6149	2.0725	0	9	0	0	0	n/a	2	Medium	0	
**ND_L174S**		190	200	AEYHAKATEHL	1268.6149	1.9961	30	9	0.186	3.243	12.011	0.614	2	Medium	0.068	0.00521169
**ND_L174S**		190	200	AEYHAKATEHL	1268.6149	2.0531	300	9	1.577	3.548	13.139	0.051	2	Medium	0.006	0.0308902
**ND_L174S**		190	200	AEYHAKATEHL	1268.6149	2.0631	3000	9	6.422	3.977	14.731	0.369	2	Medium	0.041	0.353976
**ND_L174S**		190	200	AEYHAKATEHL	1268.6149	2.062	9000	9	8.488	3.971	14.708	0.231	2	Medium	0.026	0.130614
**ND_L174S**		190	201	AEYHAKATEHLS	1355.647	1.7083	0	10	0	0	0	n/a	2	Medium	0	
**ND_L174S**		190	201	AEYHAKATEHLS	1355.647	2.0822	30	10	0.195	3.702	18.511	0.367	2	Medium	0.041	0.0196228
**ND_L174S**		190	201	AEYHAKATEHLS	1355.647	2.0952	300	10	1.667	4.001	20.006	0.304	2	Medium	0.034	0.168661
**ND_L174S**		190	201	AEYHAKATEHLS	1355.647	2.0581	3000	10	7.032	4.988	24.938	0.248	2	Medium	0.028	0.0696165
**ND_L174S**		190	201	AEYHAKATEHLS	1355.647	2.0539	9000	10	9.428	5.193	25.967	0.053	2	Medium	0.006	0.05683
**ND_L174S**		190	202	AEYHAKATEHLST	1456.6947	1.8253	0	11	0	0	0	n/a	2	High	0	
**ND_L174S**		190	202	AEYHAKATEHLST	1456.6947	1.8744	30	11	0.203	3.903	17.74	0.111	2	High	0.012	0.01992
**ND_L174S**		190	202	AEYHAKATEHLST	1456.6947	1.8977	300	11	1.747	4.397	19.988	0.032	2	High	0.004	0.0281566
**ND_L174S**		190	202	AEYHAKATEHLST	1456.6947	1.8975	3000	11	7.598	4.937	22.441	0.311	2	High	0.035	0.0378088
**ND_L174S**		190	202	AEYHAKATEHLST	1456.6947	1.8915	9000	11	10.347	5.003	22.74	0.056	2	High	0.006	0.264626
**ND_L174S**		190	203	AEYHAKATEHLSTL	1569.7787	2.7648	0	12	0	0	0	n/a	2	High	0	
**ND_L174S**		190	203	AEYHAKATEHLSTL	1569.7787	2.7405	30	12	0.206	4.009	16.705	0.024	2	High	0.003	0.0189236
**ND_L174S**		190	203	AEYHAKATEHLSTL	1569.7787	2.7646	300	12	1.775	4.632	19.302	0.092	2	High	0.01	0.0407306
**ND_L174S**		190	203	AEYHAKATEHLSTL	1569.7787	2.7646	3000	12	7.848	5.244	21.849	0.55	2	High	0.061	0.274679
**ND_L174S**		190	203	AEYHAKATEHLSTL	1569.7787	2.7618	9000	12	10.925	5.261	21.92	0.017	2	High	0.002	0.251336
**ND_L174S**		190	205	AEYHAKATEHLSTLSE	1785.8533	2.5301	0	14	0	0	0	n/a	2	High	0	
**ND_L174S**		190	205	AEYHAKATEHLSTLSE	1785.8533	2.5487	30	14	0.232	4.944	17.656	0.328	2	High	0.036	0.0236068
**ND_L174S**		190	205	AEYHAKATEHLSTLSE	1785.8533	2.5017	300	14	2.016	5.762	20.578	0.632	2	High	0.07	0.0101031
**ND_L174S**		190	205	AEYHAKATEHLSTLSE	1785.8533	2.5679	3000	14	9.263	6.485	23.161	0.806	2	High	0.09	0.338899
**ND_L174S**		190	205	AEYHAKATEHLSTLSE	1785.8533	2.5571	9000	14	12.858	6.488	23.173	0.142	2	High	0.016	0.259754
**ND_L174S**		190	211	AEYHAKATEHLSTLSEKAKPAL	2394.2543	2.7222	0	19	0	0	0	n/a	2	High	0	
**ND_L174S**		190	211	AEYHAKATEHLSTLSEKAKPAL	2394.2543	2.629	30	19	0.275	5.812	7.648	0.243	2	High	0.027	0.026326
**ND_L174S**		190	211	AEYHAKATEHLSTLSEKAKPAL	2394.2543	2.6385	300	19	2.434	7.252	9.542	0.729	2	High	0.081	0.0050048
**ND_L174S**		190	211	AEYHAKATEHLSTLSEKAKPAL	2394.2543	2.6547	3000	19	12.095	8.544	11.241	0.572	2	High	0.064	0.0927271
**ND_L174S**		190	211	AEYHAKATEHLSTLSEKAKPAL	2394.2543	2.6456	9000	19	17.33	8.614	11.335	0.117	2	High	0.013	0.238099
**ND_L174S**		190	212	AEYHAKATEHLSTLSEKAKPALE	2523.2969	2.5946	0	20	0	0	0	n/a	2	High	0	
**ND_L174S**		190	212	AEYHAKATEHLSTLSEKAKPALE	2523.2969	2.5522	30	20	0.282	5.822	9.703	0.17	2	High	0.019	0.000393748
**ND_L174S**		190	212	AEYHAKATEHLSTLSEKAKPALE	2523.2969	2.559	300	20	2.5	7.619	12.698	0.001	2	High	0	0.0317429
**ND_L174S**		190	212	AEYHAKATEHLSTLSEKAKPALE	2523.2969	2.5638	3000	20	12.586	9.136	15.227	0.668	2	High	0.074	0.100098
**ND_L174S**		190	212	AEYHAKATEHLSTLSEKAKPALE	2523.2969	2.5564	9000	20	18.199	9.305	15.508	0.655	2	High	0.073	0.614917
**ND_L174S**		190	213	AEYHAKATEHLSTLSEKAKPALED	2638.3239	2.6177	0	21	0	0	0	n/a	2	High	0	
**ND_L174S**		190	213	AEYHAKATEHLSTLSEKAKPALED	2638.3239	2.5906	30	21	0.335	5.999	9.523	0.076	2	High	0.008	0.00566574
**ND_L174S**		190	213	AEYHAKATEHLSTLSEKAKPALED	2638.3239	2.6115	300	21	2.917	7.651	12.145	0.426	2	High	0.047	0.0205536
**ND_L174S**		190	213	AEYHAKATEHLSTLSEKAKPALED	2638.3239	2.6008	3000	21	13.582	9.396	14.915	0.013	2	High	0.001	0.0111782
**ND_L174S**		190	213	AEYHAKATEHLSTLSEKAKPALED	2638.3239	2.5984	9000	21	19.199	9.615	15.263	0.511	2	High	0.057	0.13005
**ND_L174S**		190	214	AEYHAKATEHLSTLSEKAKPALEDL	2751.4079	3.5052	0	22	0	0	0	n/a	2	High	0	
**ND_L174S**		190	214	AEYHAKATEHLSTLSEKAKPALEDL	2751.4079	3.4652	30	22	0.342	5.701	6.962	0.471	4	Medium	0.296	5.14E-05
**ND_L174S**		190	214	AEYHAKATEHLSTLSEKAKPALEDL	2751.4079	3.5061	300	22	2.986	7.407	9.07	0.818	4	Medium	0.514	0.00133526

**ND_L174S**		190	214	AEYHAKATEHLSTLSEKAKPALEDL	2751.4079	3.4765	3000	22	14.093	9.719	11.824	0.264	4	High	0.166	0.00436384
**ND_L174S**		190	214	AEYHAKATEHLSTLSEKAKPALEDL	2751.4079	3.4742	9000	22	20.082	9.937	12.076	0.191	4	High	0.12	0.0143073
**ND_L174S**		190	219	AEYHAKATEHLSTLSEKAKPALEDLRQGLL	3318.7572	4.1375	0	27	0	0	0	n/a	2	High	0	
**ND_L174S**		190	219	AEYHAKATEHLSTLSEKAKPALEDLRQGLL	3318.7572	4.0685	30	27	0.377	5.935	5.495	1.659	2	High	0.185	0.000574164
**ND_L174S**		190	219	AEYHAKATEHLSTLSEKAKPALEDLRQGLL	3318.7572	4.0908	300	27	3.326	8.682	8.039	1.203	2	High	0.134	0.00104773
**ND_L174S**		190	219	AEYHAKATEHLSTLSEKAKPALEDLRQGLL	3318.7572	4.0882	3000	27	16.525	11.874	10.995	0.617	2	High	0.069	0.00802359
**ND_L174S**		190	219	AEYHAKATEHLSTLSEKAKPALEDLRQGLL	3318.7572	4.0802	9000	27	24.236	12.44	11.519	0.163	2	High	0.018	0.0986743
**ND_L174S**		190	222	AEYHAKATEHLSTLSEKAKPALEDLRQGLLPV	3627.9624	4.6312	0	29	0	0	0	n/a	2	Medium	0	
**ND_L174S**		190	222	AEYHAKATEHLSTLSEKAKPALEDLRQGLLPV	3627.9624	4.6588	30	29	0.381	3.497	4.019	4.997	2	Medium	0.556	0.00812654
**ND_L174S**		190	222	AEYHAKATEHLSTLSEKAKPALEDLRQGLLPV	3627.9624	4.6288	300	29	3.366	8.655	7.745	1.72	4	Medium	1.081	0.00200632
**ND_L174S**		190	222	AEYHAKATEHLSTLSEKAKPALEDLRQGLLPV	3627.9624	4.6152	3000	29	16.898	12.184	11.128	0.76	4	Medium	0.478	0.000397273
**ND_L174S**		190	222	AEYHAKATEHLSTLSEKAKPALEDLRQGLLPV	3627.9624	4.6256	9000	29	25.155	13.209	12.044	0.967	4	Medium	0.608	0.0227586
**ND_L174S**		192	203	YHAKATEHLSTL	1369.699	2.6176	0	10	0	0	0	n/a	2	Medium	0	
**ND_L174S**		192	203	YHAKATEHLSTL	1369.699	2.599	30	10	0.166	3.456	11.521	0.598	2	Medium	0.067	0.00893727
**ND_L174S**		192	203	YHAKATEHLSTL	1369.699	2.6236	300	10	1.421	3.933	13.111	0.069	2	Medium	0.008	0.11122
**ND_L174S**		192	203	YHAKATEHLSTL	1369.699	2.6154	3000	10	6.328	4.497	14.991	0.06	2	Medium	0.007	0.013512
**ND_L174S**		192	203	YHAKATEHLSTL	1369.699	2.5974	9000	10	9.01	4.459	14.865	0.207	2	Medium	0.023	0.0678731
**ND_L174S**		192	211	YHAKATEHLSTLSEKAKPAL	2194.1746	2.5761	0	17	0	0	0	n/a	2	Medium	0	
**ND_L174S**		192	211	YHAKATEHLSTLSEKAKPAL	2194.1746	2.5303	30	17	0.235	5.099	9.998	0.047	2	Medium	0.005	0.0009665
**ND_L174S**		192	211	YHAKATEHLSTLSEKAKPAL	2194.1746	2.5615	300	17	2.08	6.374	12.499	0.32	2	Medium	0.036	0.0312132
**ND_L174S**		192	211	YHAKATEHLSTLSEKAKPAL	2194.1746	2.5489	3000	17	10.575	7.882	15.454	0.345	2	Medium	0.038	0.352354
**ND_L174S**		192	211	YHAKATEHLSTLSEKAKPAL	2194.1746	2.5377	9000	17	15.416	7.883	15.457	0.252	2	Medium	0.028	0.55799
**ND_L174S**		193	211	HAKATEHLSTLSEKAKPAL	2031.1113	2.4387	0	16	0	0	0	n/a	2	Medium	0	
**ND_L174S**		193	211	HAKATEHLSTLSEKAKPAL	2031.1113	2.3889	30	16	0.203	4.852	7.969	0.546	4	Medium	0.343	2.85E-05
**ND_L174S**		193	211	HAKATEHLSTLSEKAKPAL	2031.1113	2.4139	300	16	1.796	6.31	10.549	0.21	4	Medium	0.132	3.14E-05
**ND_L174S**		193	211	HAKATEHLSTLSEKAKPAL	2031.1113	2.4439	3000	16	9.611	7.364	12.244	0.147	4	Medium	0.092	0.231794
**ND_L174S**		193	211	HAKATEHLSTLSEKAKPAL	2031.1113	2.4023	9000	16	14.416	7.448	12.4	0.135	4	Medium	0.085	0.726505
**ND_L174S**		199	211	HLSTLSEKAKPAL	1393.7929	2.5717	0	10	0	0	0	n/a	2	Medium	0	
**ND_L174S**		199	211	HLSTLSEKAKPAL	1393.7929	2.5276	30	10	0.09	3.323	11.076	0.237	2	Medium	0.026	0.00162821
**ND_L174S**		199	211	HLSTLSEKAKPAL	1393.7929	2.5417	300	10	0.857	4.098	13.662	0.355	2	Medium	0.039	0.00165996
**ND_L174S**		199	211	HLSTLSEKAKPAL	1393.7929	2.5517	3000	10	5.673	5.017	16.724	0.411	2	Medium	0.046	0.107491
**ND_L174S**		199	211	HLSTLSEKAKPAL	1393.7929	2.536	9000	10	8.843	5.072	16.908	0.439	2	Medium	0.049	0.221915
**ND_L174S**		201	212	STLSEKAKPALE	1272.6925	2.3345	0	9	0	0	0	n/a	2	High	0	
**ND_L174S**		201	212	STLSEKAKPALE	1272.6925	2.2998	30	9	0.079	3.031	16.842	0.662	2	Medium	0.074	0.000756112
**ND_L174S**		201	212	STLSEKAKPALE	1272.6925	2.3143	300	9	0.753	4.031	22.392	0.474	2	Medium	0.053	0.0151083
**ND_L174S**		201	212	STLSEKAKPALE	1272.6925	2.3224	3000	9	4.989	5.115	28.417	0.055	2	Medium	0.006	0.0293188
**ND_L174S**		201	212	STLSEKAKPALE	1272.6925	2.2961	9000	9	7.852	5.253	29.181	0.179	2	Medium	0.02	0.0197697
**ND_L174S**		201	219	STLSEKAKPALEDLRQGLL	2068.1528	4.4372	0	16	0	0	0	n/a	2	Medium	0	
**ND_L174S**		201	219	STLSEKAKPALEDLRQGLL	2068.1528	4.4251	30	16	0.174	3.581	7.46	0.223	2	Medium	0.025	0.0117952
**ND_L174S**		201	219	STLSEKAKPALEDLRQGLL	2068.1528	4.43	300	16	1.579	6.005	12.51	1.338	2	Medium	0.149	0.00664801
**ND_L174S**		201	219	STLSEKAKPALEDLRQGLL	2068.1528	4.4204	3000	16	8.928	8.718	18.162	0.567	2	Medium	0.063	0.022078
**ND_L174S**		201	219	STLSEKAKPALEDLRQGLL	2068.1528	4.4293	9000	16	13.889	9.266	19.303	0.478	2	Medium	0.053	0.0443552
**ND_L174S**		203	212	LSEKAKPALE	1084.6128	1.9712	0	7	0	0	0	n/a	2	High	0	
**ND_L174S**		203	212	LSEKAKPALE	1084.6128	1.9248	30	7	0.067	2.154	15.385	0.069	2	High	0.008	0.00263985
**ND_L174S**		203	212	LSEKAKPALE	1084.6128	1.9405	300	7	0.635	2.835	20.247	0.35	2	High	0.039	0.00248758
**ND_L174S**		203	212	LSEKAKPALE	1084.6128	1.9444	3000	7	4.129	3.729	26.638	0.093	2	High	0.01	0.00465116
**ND_L174S**		203	212	LSEKAKPALE	1084.6128	1.9371	9000	7	6.334	3.81	27.217	0.135	2	High	0.015	0.0666257
**ND_L174S**		203	213	LSEKAKPALED	1199.6398	2.0709	0	8	0	0	0	n/a	2	High	0	
**ND_L174S**		203	213	LSEKAKPALED	1199.6398	2.0368	30	8	0.119	2.288	14.299	0.18	2	High	0.02	0.00429612
**ND_L174S**		203	213	LSEKAKPALED	1199.6398	2.0532	300	8	1.052	3.089	19.306	0.347	2	High	0.039	0.001052
**ND_L174S**		203	213	LSEKAKPALED	1199.6398	2.062	3000	8	5.125	4.123	25.767	0.19	2	High	0.021	0.0381763
**ND_L174S**		203	213	LSEKAKPALED	1199.6398	2.0515	9000	8	7.334	4.234	26.463	0.091	2	High	0.01	0.205221
**ND_L174S**		203	214	LSEKAKPALEDL	1312.7238	3.6176	0	9	0	0	0	n/a	2	High	0	
**ND_L174S**		203	214	LSEKAKPALEDL	1312.7238	3.5984	30	9	0.126	2.409	13.385	0.711	2	High	0.079	0.0134089
**ND_L174S**		203	214	LSEKAKPALEDL	1312.7238	3.6219	300	9	1.121	3.312	18.398	0.06	2	Medium	0.007	0.0146163
**ND_L174S**		203	214	LSEKAKPALEDL	1312.7238	3.6222	3000	9	5.636	4.598	25.546	0.168	2	High	0.019	0.0445482
**ND_L174S**		203	214	LSEKAKPALEDL	1312.7238	3.6306	9000	9	8.217	4.752	26.398	0.582	2	Medium	0.065	0.571967
**ND_L174S**		203	219	LSEKAKPALEDLRQGLL	1880.0731	4.2432	0	14	0	0	0	n/a	2	High	0	
**ND_L174S**		203	219	LSEKAKPALEDLRQGLL	1880.0731	4.2112	30	14	0.162	2.671	9.538	0.584	2	Medium	0.065	0.00759059
**ND_L174S**		203	219	LSEKAKPALEDLRQGLL	1880.0731	4.2238	300	14	1.461	4.498	16.064	1.328	2	High	0.148	0.00306879
**ND_L174S**		203	219	LSEKAKPALEDLRQGLL	1880.0731	4.2225	3000	14	8.068	6.928	24.744	0.797	2	High	0.089	0.0272315
**ND_L174S**		203	219	LSEKAKPALEDLRQGLL	1880.0731	4.2258	9000	14	12.371	7.588	27.101	0.288	2	High	0.032	0.104725
**ND_L174S**		203	222	LSEKAKPALEDLRQGLLPVL	2189.2784	4.9497	0	16	0	0	0	n/a	2	Medium	0	
**ND_L174S**		203	222	LSEKAKPALEDLRQGLLPVL	2189.2784	4.9341	30	16	0.166	2.568	6.58	0.428	4	Medium	0.269	1.35E-05
**ND_L174S**		203	222	LSEKAKPALEDLRQGLLPVL	2189.2784	4.9294	300	16	1.501	4.868	12.542	0.803	4	Medium	0.505	0.000206005
**ND_L174S**		203	222	LSEKAKPALEDLRQGLLPVL	2189.2784	4.9335	3000	16	8.441	7.769	20.269	0.175	4	Medium	0.11	4.62E-05
**ND_L174S**		203	222	LSEKAKPALEDLRQGLLPVL	2189.2784	4.9312	9000	16	13.29	8.696	22.683	0.144	4	Medium	0.091	0.0134995

ND_L174S		204	211	SEKAKPAL	842.4862	1.9708	0	5	0	0	0	n/a	2	Medium	0	
ND_L174S		204	211	SEKAKPAL	842.4862	1.8913	30	5	0.044	1.467	14.67	0.335	2	Medium	0.037	0.000495233
ND_L174S		204	211	SEKAKPAL	842.4862	1.9164	300	5	0.418	2.324	23.243	0.032	2	Medium	0.004	0.0336297
ND_L174S		204	211	SEKAKPAL	842.4862	1.9537	3000	5	2.832	2.739	27.387	0.143	2	Medium	0.016	0.066183
ND_L174S		204	211	SEKAKPAL	842.4862	1.9588	9000	5	4.473	2.754	27.542	0.01	2	Medium	0.001	0.0250907
ND_L174S		204	212	SEKAKPALE	971.5288	1.6024	0	6	0	0	0	n/a	2	High	0	
ND_L174S		204	212	SEKAKPALE	971.5288	1.5752	30	6	0.05	1.776	14.799	0.538	2	High	0.06	0.00245076
ND_L174S		204	212	SEKAKPALE	971.5288	1.5837	300	6	0.483	2.455	20.454	0.17	2	High	0.019	0.0157453
ND_L174S		204	212	SEKAKPALE	971.5288	1.5998	3000	6	3.323	3.239	26.995	0.24	2	High	0.027	0.108715
ND_L174S		204	212	SEKAKPALE	971.5288	1.5912	9000	6	5.341	3.327	27.725	0.099	2	High	0.011	0.419554
ND_L174S		204	213	SEKAKPALED	1086.5557	1.759	0	7	0	0	0	n/a	2	High	0	
ND_L174S		204	213	SEKAKPALED	1086.5557	1.7327	30	7	0.103	1.902	13.588	0.112	2	High	0.013	0.00812724
ND_L174S		204	213	SEKAKPALED	1086.5557	1.7543	300	7	0.901	2.618	18.703	0.555	2	High	0.062	0.0246329
ND_L174S		204	213	SEKAKPALED	1086.5557	1.7527	3000	7	4.319	3.61	25.787	0.059	2	High	0.007	0.0306765
ND_L174S		204	213	SEKAKPALED	1086.5557	1.7497	9000	7	6.341	3.708	26.485	0.116	2	High	0.013	0.0979
ND_L174S		204	214	SEKAKPALEDL	1199.6398	3.516	0	8	0	0	0	n/a	2	High	0	
ND_L174S		204	214	SEKAKPALEDL	1199.6398	3.4921	30	8	0.11	2.064	12.898	0.154	2	High	0.017	0.0057404
ND_L174S		204	214	SEKAKPALEDL	1199.6398	3.4987	300	8	0.97	2.916	18.225	0.229	2	Medium	0.025	0.00953964
ND_L174S		204	214	SEKAKPALEDL	1199.6398	3.5044	3000	8	4.829	4.122	25.762	0.151	2	Medium	0.017	0.00965261
ND_L174S		204	214	SEKAKPALEDL	1199.6398	3.4899	9000	8	7.224	4.278	26.738	0.094	2	Medium	0.01	0.306223
ND_L174S		204	219	SEKAKPALEDLRQGLL	1766.989	4.165	0	13	0	0	0	n/a	2	High	0	
ND_L174S		204	219	SEKAKPALEDLRQGLL	1766.989	4.1371	30	13	0.145	2.772	10.662	0.266	2	Medium	0.03	3.93E-05
ND_L174S		204	219	SEKAKPALEDLRQGLL	1766.989	4.1558	300	13	1.309	4.384	16.861	0.576	2	Medium	0.064	0.00192403
ND_L174S		204	219	SEKAKPALEDLRQGLL	1766.989	4.0553	3000	13	7.262	7.086	27.253	0.093	2	Medium	0.01	0.0680366
ND_L174S		204	219	SEKAKPALEDLRQGLL	1766.989	4.1425	9000	13	11.378	7.199	27.687	0.354	2	Medium	0.039	0.0344416
ND_L174S		204	222	SEKAKPALEDLRQGLLPVL	2076.1943	4.9131	0	15	0	0	0	n/a	2	High	0	
ND_L174S		204	222	SEKAKPALEDLRQGLLPVL	2076.1943	4.8745	30	15	0.149	2.48	6.845	0.254	4	Medium	0.159	2.95E-08
ND_L174S		204	222	SEKAKPALEDLRQGLLPVL	2076.1943	4.8938	300	15	1.35	4.433	12.315	0.146	4	High	0.092	8.69E-09
ND_L174S		204	222	SEKAKPALEDLRQGLLPVL	2076.1943	4.8906	3000	15	7.635	7.301	20.308	0.13	4	High	0.081	4.99E-07
ND_L174S		204	222	SEKAKPALEDLRQGLLPVL	2076.1943	4.886	9000	15	12.297	8.239	22.917	0.123	4	High	0.077	1.87E-05
ND_L174S		206	222	KAKPALEDLRQGLLPVL	1860.1197	4.8419	0	13	0	0	0	n/a	2	Medium	0	
ND_L174S		206	222	KAKPALEDLRQGLLPVL	1860.1197	4.8718	30	13	0.127	1.462	4.511	0.526	4	Medium	0.33	1.75E-07
ND_L174S		206	222	KAKPALEDLRQGLLPVL	1860.1197	4.8364	300	13	1.132	3.353	10.668	0.364	4	Medium	0.229	2.40E-06
ND_L174S		206	222	KAKPALEDLRQGLLPVL	1860.1197	4.8205	3000	13	6.266	6.245	20.181	0.476	4	Medium	0.299	0.00036501
ND_L174S		206	222	KAKPALEDLRQGLLPVL	1860.1197	4.8313	9000	13	10.36	7.219	23.222	0.306	4	Medium	0.193	0.00214343
ND_L174S		212	219	EDLRQGLL	942.5134	4.0527	0	6	0	0	0	n/a	2	Medium	0	
ND_L174S		212	219	EDLRQGLL	942.5134	4.0298	30	6	0.042	0.98	12.26	0.006	4	Medium	0.004	9.33E-06
ND_L174S		212	219	EDLRQGLL	942.5134	4.0494	300	6	0.408	1.601	20.041	0.053	4	Medium	0.033	1.50E-08
ND_L174S		212	219	EDLRQGLL	942.5134	4.0479	3000	6	2.944	2.835	35.514	0.05	4	Medium	0.031	3.88E-07
ND_L174S		212	219	EDLRQGLL	942.5134	4.0413	9000	6	5.037	3.248	40.686	0.045	4	Medium	0.028	7.30E-06
ND_L174S		212	222	EDLRQGLLPVL	1251.7187	5.1901	0	8	0	0	0	n/a	2	High	0	
ND_L174S		212	222	EDLRQGLLPVL	1251.7187	5.1605	30	8	0.046	1.087	6.792	0.058	2	High	0.006	1.56E-05
ND_L174S		212	222	EDLRQGLLPVL	1251.7187	5.1543	300	8	0.449	1.87	11.689	0.596	2	High	0.066	0.00872922
ND_L174S		212	222	EDLRQGLLPVL	1251.7187	5.1808	3000	8	3.316	3.556	22.228	0.473	2	High	0.053	0.0168516
ND_L174S		212	222	EDLRQGLLPVL	1251.7187	5.1686	9000	8	5.956	4.309	26.928	0.278	2	High	0.031	0.00277285
ND_L174S		213	219	DLRQGLL	813.4709	4.1926	0	5	0	0	0	n/a	2	High	0	
ND_L174S		213	219	DLRQGLL	813.4709	4.1613	30	5	0.035	0.85	17.006	0.055	2	High	0.006	0.00214396
ND_L174S		213	219	DLRQGLL	813.4709	4.1789	300	5	0.339	1.353	27.067	0.302	2	High	0.034	0.00941384
ND_L174S		213	219	DLRQGLL	813.4709	4.1676	3000	5	2.433	2.395	47.902	0.426	2	Medium	0.047	0.0339771
ND_L174S		213	219	DLRQGLL	813.4709	4.1669	9000	5	4.154	2.756	55.114	0.03	2	High	0.003	0.0557719
ND_L174S		213	222	DLRQGLLPVL	1122.6761	5.2992	0	7	0	0	0	n/a	2	High	0	
ND_L174S		213	222	DLRQGLLPVL	1122.6761	5.284	30	7	0.039	0.979	6.994	0.025	2	High	0.003	0.00407288
ND_L174S		213	222	DLRQGLLPVL	1122.6761	5.2978	300	7	0.38	1.65	11.786	0.466	2	High	0.052	0.000377512
ND_L174S		213	222	DLRQGLLPVL	1122.6761	5.2907	3000	7	2.805	3.141	22.434	0.445	2	High	0.05	0.0155296
ND_L174S		213	222	DLRQGLLPVL	1122.6761	5.2891	9000	7	5.073	3.827	27.333	0.014	2	High	0.002	0.0214997
ND_L174S		214	222	LRQGLLPVL	1007.6492	4.9853	0	6	0	0	0	n/a	2	Medium	0	
ND_L174S		214	222	LRQGLLPVL	1007.6492	4.9416	30	6	0.034	0.826	10.235	0.059	4	Medium	0.037	5.45E-07
ND_L174S		214	222	LRQGLLPVL	1007.6492	4.9582	300	6	0.328	1.388	17.449	0.159	4	High	0.1	9.54E-08
ND_L174S		214	222	LRQGLLPVL	1007.6492	4.9559	3000	6	2.39	2.603	32.571	0.064	4	Medium	0.04	5.28E-07
ND_L174S		214	222	LRQGLLPVL	1007.6492	4.952	9000	6	4.273	3.245	40.642	0.043	4	Medium	0.027	2.54E-06
ND_L174S		215	222	RQGLLPVL	894.5651	4.824	0	5	0	0	0	n/a	2	High	0	
ND_L174S		215	222	RQGLLPVL	894.5651	4.7645	30	5	0.024	0.588	5.883	0.132	2	High	0.015	0.0052328
ND_L174S		215	222	RQGLLPVL	894.5651	4.7823	300	5	0.23	0.95	9.505	0.349	2	High	0.039	0.00798097
ND_L174S		215	222	RQGLLPVL	894.5651	4.7822	3000	5	1.748	1.972	19.721	0.368	2	High	0.041	0.0135457
ND_L174S		215	222	RQGLLPVL	894.5651	4.7737	9000	5	3.319	2.576	25.762	0.125	2	High	0.014	0.0242761
ND_L174S		223	229	ESFKVSF	842.4174	4.5065	0	5	0	0	0	n/a	2	Medium	0	
ND_L174S		223	229	ESFKVSF	842.4174	4.4729	30	5	0.03	0.53	10.597	0.592	2	Medium	0.066	0.00914631

**ND_L174S**		223	229	ESFKVSF	842.4174	4.4935	300	5	0.295	1.13	22.591	0.089	2	Medium	0.01	2.61E-05
**ND_L174S**		223	229	ESFKVSF	842.4174	4.498	3000	5	2.211	2.212	44.231	0.524	2	Medium	0.058	0.00378932
**ND_L174S**		223	229	ESFKVSF	842.4174	4.4963	9000	5	3.967	2.776	55.522	0.33	2	Medium	0.037	0.0678169
**ND_L174S**		226	232	KVSFLSA	750.4276	3.8985	0	5	0	0	0	n/a	2	Medium	0	
**ND_L174S**		226	232	KVSFLSA	750.4276	3.8687	30	5	0.041	0.582	11.641	0.104	2	Medium	0.012	0.00231091
**ND_L174S**		226	232	KVSFLSA	750.4276	3.887	300	5	0.393	1.239	24.774	0.31	2	Medium	0.035	0.00128024
**ND_L174S**		226	232	KVSFLSA	750.4276	3.8854	3000	5	2.659	2.363	47.265	0.029	2	Medium	0.003	0.00764135
**ND_L174S**		226	232	KVSFLSA	750.4276	3.8858	9000	5	4.246	2.671	53.421	0.128	2	Medium	0.014	0.0240019
**ND_L174S**		230	235	LSALEE	660.333	3.0377	0	4	0	0	0	n/a	2	Medium	0	
**ND_L174S**		230	235	LSALEE	660.333	3.036	30	4	0.044	0.603	15.069	0.182	2	Medium	0.02	0.0040042
**ND_L174S**		230	235	LSALEE	660.333	3.0448	300	4	0.414	1.081	27.028	0.177	2	Medium	0.02	0.00259857
**ND_L174S**		230	235	LSALEE	660.333	3.0286	3000	4	2.434	2.074	51.845	0.133	2	High	0.015	0.00183796
**ND_L174S**		230	235	LSALEE	660.333	3.0151	9000	4	3.559	2.378	59.446	0.003	2	Medium	0	0.164081
**ND_L174S**		233	243	LEEYTKKLNTQ	1365.714	2.3362	0	9	0	0	0	n/a	2	High	0	
**ND_L174S**		233	243	LEEYTKKLNTQ	1365.714	2.3111	30	9	0.072	4.303	23.908	0.046	2	High	0.005	0.00949198
**ND_L174S**		233	243	LEEYTKKLNTQ	1365.714	2.3296	300	9	0.686	4.617	25.65	0.137	2	High	0.015	0.0181728
**ND_L174S**		233	243	LEEYTKKLNTQ	1365.714	2.3338	3000	9	4.527	5.101	28.34	0.477	2	High	0.053	0.423735
**ND_L174S**		233	243	LEEYTKKLNTQ	1365.714	2.3232	9000	9	7.358	5.05	28.055	0.057	2	High	0.006	0.480798
**ND_L174S**		234	243	EEYTKKLNTQ	1252.6299	1.9199	0	8	0	0	0	n/a	2	High	0	
**ND_L174S**		234	243	EEYTKKLNTQ	1252.6299	1.8874	30	8	0.052	4.21	52.63	0.337	2	High	0.037	0.0282642
**ND_L174S**		234	243	EEYTKKLNTQ	1252.6299	1.9031	300	8	0.503	4.531	56.633	0.329	2	High	0.037	0.0481932
**ND_L174S**		234	243	EEYTKKLNTQ	1252.6299	1.9068	3000	8	3.661	4.925	61.569	0.22	2	High	0.025	0.447083
**ND_L174S**		234	243	EEYTKKLNTQ	1252.6299	1.8952	9000	8	6.361	4.95	61.873	0.548	2	High	0.061	0.904371
**ND_L174S**		236	243	YTKKLNTQ	994.5447	1.4164	0	6	0	0	0	n/a	2	Medium	0	
**ND_L174S**		236	243	YTKKLNTQ	994.5447	1.4341	30	6	0.039	3.588	59.798	0.244	2	High	0.027	0.479733
**ND_L174S**		236	243	YTKKLNTQ	994.5447	1.4315	300	6	0.375	3.501	58.343	0.062	2	High	0.007	0.13013
**ND_L174S**		236	243	YTKKLNTQ	994.5447	1.4326	3000	6	2.705	3.663	61.051	0.475	2	High	0.053	0.115941
**ND_L174S**		236	243	YTKKLNTQ	994.5447	1.4313	9000	6	4.666	3.664	61.069	0.008	2	High	0.001	0.0793305
**LF_WT**		17	28	VYVDVLKDSGRD	1364.6936	3.3152	0	10	0	0	0	n/a	2	High	0	
**LF_WT**		17	28	VYVDVLKDSGRD	1364.6936	3.3301	30	10	0.164	2.064	10.32	0.154	2	High	0.017	
**LF_WT**		17	28	VYVDVLKDSGRD	1364.6936	3.3081	300	10	1.45	2.774	13.871	0.272	2	High	0.03	
**LF_WT**		17	28	VYVDVLKDSGRD	1364.6936	3.3096	3000	10	6.286	3.743	18.715	0.124	2	High	0.014	
**LF_WT**		17	28	VYVDVLKDSGRD	1364.6936	3.3027	9000	10	8.411	4.244	21.222	0.447	2	High	0.05	
**LF_WT**		17	46	VYVDVLKDSGRDYVSQFEGSALGKQLNLKL	3340.7667	5.3135	0	28	0	0	0	n/a	2	High	0	
**LF_WT**		17	46	VYVDVLKDSGRDYVSQFEGSALGKQLNLKL	3340.7667	5.3119	30	28	0.322	4.672	3.337	3.113	2	Medium	0.346	
**LF_WT**		17	46	VYVDVLKDSGRDYVSQFEGSALGKQLNLKL	3340.7667	5.3114	300	28	2.951	8.794	6.281	2.315	2	High	0.258	
**LF_WT**		17	46	VYVDVLKDSGRDYVSQFEGSALGKQLNLKL	3340.7667	5.2827	3000	28	16.007	13.645	9.747	0.527	2	High	0.059	
**LF_WT**		17	46	VYVDVLKDSGRDYVSQFEGSALGKQLNLKL	3340.7667	5.2787	9000	28	23.684	15.066	10.761	2.203	2	High	0.245	
**LF_WT**		18	28	YVDVLKDSGRD	1265.6252	3.0536	0	9	0	0	0	n/a	2	High	0	
**LF_WT**		18	28	YVDVLKDSGRD	1265.6252	3.0496	30	9	0.163	2.099	11.664	0.163	2	High	0.018	
**LF_WT**		18	28	YVDVLKDSGRD	1265.6252	3.036	300	9	1.432	2.719	15.103	0.183	2	High	0.02	
**LF_WT**		18	28	YVDVLKDSGRD	1265.6252	3.0432	3000	9	6.117	3.618	20.099	0.034	2	High	0.004	
**LF_WT**		18	28	YVDVLKDSGRD	1265.6252	3.0407	9000	9	7.986	4.036	22.42	0.469	2	High	0.052	
**LF_WT**		19	28	VDVLKDSGRD	1102.5619	2.3293	0	8	0	0	0	n/a	2	High	0	
**LF_WT**		19	28	VDVLKDSGRD	1102.5619	2.3274	30	8	0.145	2.129	13.309	0.463	2	High	0.052	
**LF_WT**		19	28	VDVLKDSGRD	1102.5619	2.2756	300	8	1.269	2.662	16.64	0.326	2	High	0.036	
**LF_WT**		19	28	VDVLKDSGRD	1102.5619	2.3167	3000	8	5.286	3.521	22.007	0.146	2	High	0.016	
**LF_WT**		19	28	VDVLKDSGRD	1102.5619	2.3186	9000	8	6.991	3.886	24.286	0.389	2	High	0.043	
**LF_WT**		19	44	VDVLKDSGRDYVSQFEGSALGKQLNL	2837.4559	5.1873	0	24	0	0	0	n/a	2	High	0	
**LF_WT**		19	44	VDVLKDSGRDYVSQFEGSALGKQLNL	2837.4559	5.1694	30	24	0.295	3.055	3.678	1.26	4	Medium	0.792	
**LF_WT**		19	44	VDVLKDSGRDYVSQFEGSALGKQLNL	2837.4559	5.1397	300	24	2.694	8.034	9.79	1.624	4	Medium	1.021	
**LF_WT**		19	44	VDVLKDSGRDYVSQFEGSALGKQLNL	2837.4559	5.1346	3000	24	14.365	12.11	14.684	0.356	4	High	0.223	
**LF_WT**		19	44	VDVLKDSGRDYVSQFEGSALGKQLNL	2837.4559	5.1102	9000	24	20.902	13.516	16.394	0.564	4	Medium	0.355	
**LF_WT**		20	28	DVLKDSGRD	1003.4934	2.3403	0	7	0	0	0	n/a	2	High	0	
**LF_WT**		20	28	DVLKDSGRD	1003.4934	2.3008	30	7	0.14	1.997	14.266	0.083	2	High	0.009	
**LF_WT**		20	28	DVLKDSGRD	1003.4934	2.299	300	7	1.219	2.484	17.743	0.288	2	High	0.032	
**LF_WT**		20	28	DVLKDSGRD	1003.4934	2.3028	3000	7	4.883	3.291	23.507	0.345	2	High	0.038	
**LF_WT**		20	28	DVLKDSGRD	1003.4934	2.3073	9000	7	6.203	3.616	25.826	0.457	2	High	0.051	
**LF_WT**		29	37	YVSQFEGSA	986.4345	3.2566	0	7	0	0	0	n/a	2	High	0	
**LF_WT**		29	37	YVSQFEGSA	986.4345	3.2545	30	7	0.086	1.946	27.798	0.358	2	High	0.04	
**LF_WT**		29	37	YVSQFEGSA	986.4345	3.1637	300	7	0.805	2.386	34.088	0.579	2	Medium	0.064	
**LF_WT**		29	37	YVSQFEGSA	986.4345	3.2496	3000	7	4.753	3.336	47.66	0.18	2	High	0.02	
**LF_WT**		29	37	YVSQFEGSA	986.4345	3.2477	9000	7	6.655	3.748	53.538	0.6	2	High	0.067	
**LF_WT**		29	38	YVSQFEGSAL	1099.5186	4.2681	0	8	0	0	0	n/a	2	High	0	
**LF_WT**		29	38	YVSQFEGSAL	1099.5186	4.278	30	8	0.09	2.675	25.239	0.142	4	High	0.089	
**LF_WT**		29	38	YVSQFEGSAL	1099.5186	4.2562	300	8	0.845	2.989	28.208	0.124	4	Medium	0.078	
**LF_WT**		29	38	YVSQFEGSAL	1099.5186	4.2551	3000	8	5.092	3.924	37	0.127	4	Medium	0.08	
**LF_WT**		29	38	YVSQFEGSAL	1099.5186	4.2734	9000	8	7.366	4.3	40.528	0.141	4	High	0.088	
**LF_WT**		29	43	YVSQFEGSALGKQLN	1639.8206	3.8996	0	13	0	0	0	n/a	2	High	0	
**LF_WT**		29	43	YVSQFEGSALGKQLN	1639.8206	3.9331	30	13	0.131	4.405	16.944	0.792	2	High	0.088	
**LF_WT**		29	43	YVSQFEGSALGKQLN	1639.8206	3.8846	300	13	1.239	5.695	21.902	0.894	2	High	0.099	

**LF_WT**		29	43	YVSQFEGSALGKQLN	1639.8206	3.8855	3000	13	7.811	7.05	27.115	0.328	2	Medium	0.036	
**LF_WT**		29	43	YVSQFEGSALGKQLN	1639.8206	3.8824	9000	13	11.753	7.499	28.841	0.953	2	Medium	0.106	
**LF_WT**		29	44	YVSQFEGSALGKQLNL	1752.9046	4.8236	0	14	0	0	0	n/a	2	High	0	
**LF_WT**		29	44	YVSQFEGSALGKQLNL	1752.9046	4.8096	30	14	0.135	4.929	17.605	0.201	2	High	0.022	
**LF_WT**		29	44	YVSQFEGSALGKQLNL	1752.9046	4.8039	300	14	1.285	5.945	21.231	0.426	2	High	0.047	
**LF_WT**		29	44	YVSQFEGSALGKQLNL	1752.9046	4.7802	3000	14	8.184	7.718	27.566	0.522	2	High	0.058	
**LF_WT**		29	44	YVSQFEGSALGKQLNL	1752.9046	4.7857	9000	14	12.506	8.167	29.169	0.56	2	High	0.062	
**LF_WT**		29	46	YVSQFEGSALGKQLNLKL	1994.0837	4.9294	0	16	0	0	0	n/a	2	High	0	
**LF_WT**		29	46	YVSQFEGSALGKQLNLKL	1994.0837	4.8282	30	16	0.143	6.004	18.764	1.094	2	High	0.122	
**LF_WT**		29	46	YVSQFEGSALGKQLNLKL	1994.0837	4.7936	300	16	1.361	7.294	22.794	0.896	2	High	0.1	
**LF_WT**		29	46	YVSQFEGSALGKQLNLKL	1994.0837	4.7955	3000	16	8.826	9.221	28.815	0.051	2	High	0.006	
**LF_WT**		29	46	YVSQFEGSALGKQLNLKL	1994.0837	4.7933	9000	16	13.868	9.763	30.509	0.929	2	High	0.103	
**LF_WT**		33	38	FEGSAL	622.2962	3.5619	0	4	0	0	0	n/a	2	High	0	
**LF_WT**		33	38	FEGSAL	622.2962	3.5577	30	4	0.053	1.926	48.161	0.393	2	Medium	0.044	
**LF_WT**		33	38	FEGSAL	622.2962	3.5474	300	4	0.492	2.015	50.384	0.183	2	Medium	0.02	
**LF_WT**		33	38	FEGSAL	622.2962	3.5548	3000	4	2.714	2.118	52.941	0.07	2	Medium	0.008	
**LF_WT**		33	38	FEGSAL	622.2962	3.555	9000	4	3.676	2.121	53.02	0.293	2	Medium	0.033	
**LF_WT**		33	43	FEGSALGKQLN	1162.5982	3.2238	0	9	0	0	0	n/a	2	Medium	0	
**LF_WT**		33	43	FEGSALGKQLN	1162.5982	3.1947	30	9	0.094	4.003	22.236	0.628	2	Medium	0.07	
**LF_WT**		33	43	FEGSALGKQLN	1162.5982	3.1884	300	9	0.886	4.617	25.648	0.508	2	Medium	0.057	
**LF_WT**		33	43	FEGSALGKQLN	1162.5982	3.1566	3000	9	5.433	5.013	27.852	0.056	2	Medium	0.006	
**LF_WT**		33	43	FEGSALGKQLN	1162.5982	3.1958	9000	9	8.062	5.041	28.008	1.107	2	Medium	0.123	
**LF_WT**		33	44	FEGSALGKQLNL	1275.6823	4.4234	0	10	0	0	0	n/a	2	High	0	
**LF_WT**		33	44	FEGSALGKQLNL	1275.6823	4.4273	30	10	0.099	4.01	20.048	0.284	2	Medium	0.032	
**LF_WT**		33	44	FEGSALGKQLNL	1275.6823	4.3785	300	10	0.932	4.782	23.908	0.225	2	Medium	0.025	
**LF_WT**		33	44	FEGSALGKQLNL	1275.6823	4.4123	3000	10	5.806	5.718	28.588	0.892	2	Medium	0.099	
**LF_WT**		33	44	FEGSALGKQLNL	1275.6823	4.3958	9000	10	8.815	5.649	28.244	0.549	2	High	0.061	
**LF_WT**		33	46	FEGSALGKQLNLKL	1516.8613	4.4612	0	12	0	0	0	n/a	2	High	0	
**LF_WT**		33	46	FEGSALGKQLNLKL	1516.8613	4.4323	30	12	0.106	5.272	21.966	0.65	2	High	0.072	
**LF_WT**		33	46	FEGSALGKQLNLKL	1516.8613	4.4342	300	12	1.008	6.183	25.763	0.342	2	High	0.038	
**LF_WT**		33	46	FEGSALGKQLNLKL	1516.8613	4.4414	3000	12	6.448	7.2	29.998	0.23	2	High	0.026	
**LF_WT**		33	46	FEGSALGKQLNLKL	1516.8613	4.4415	9000	12	10.177	7.353	30.638	1.183	2	High	0.132	
**LF_WT**		34	43	EGSALGKQLN	1015.5298	2.4362	0	8	0	0	0	n/a	2	Medium	0	
**LF_WT**		34	43	EGSALGKQLN	1015.5298	2.4369	30	8	0.08	3.406	21.288	0.089	2	Medium	0.01	
**LF_WT**		34	43	EGSALGKQLN	1015.5298	2.4161	300	8	0.757	3.82	23.877	0.276	2	Medium	0.031	
**LF_WT**		34	43	EGSALGKQLN	1015.5298	2.4353	3000	8	4.685	4.395	27.47	0.421	2	Medium	0.047	
**LF_WT**		34	43	EGSALGKQLN	1015.5298	2.4344	9000	8	7.078	4.495	28.094	0.209	2	Medium	0.023	
**LF_WT**		34	44	EGSALGKQLNL	1128.6139	3.9225	0	9	0	0	0	n/a	2	Medium	0	
**LF_WT**		34	44	EGSALGKQLNL	1128.6139	3.9251	30	9	0.085	3.556	19.753	0.645	2	Medium	0.072	
**LF_WT**		34	44	EGSALGKQLNL	1128.6139	3.9189	300	9	0.803	4.082	22.679	0.35	2	Medium	0.039	
**LF_WT**		34	44	EGSALGKQLNL	1128.6139	3.952	3000	9	5.058	4.744	26.353	0.046	2	Medium	0.005	
**LF_WT**		34	44	EGSALGKQLNL	1128.6139	3.9489	9000	9	7.831	4.889	27.159	0.514	2	Medium	0.057	
**LF_WT**		34	46	EGSALGKQLNLKL	1369.7929	4.1334	0	11	0	0	0	n/a	2	High	0	
**LF_WT**		34	46	EGSALGKQLNLKL	1369.7929	4.1255	30	11	0.093	4.566	20.754	0.97	2	High	0.108	
**LF_WT**		34	46	EGSALGKQLNLKL	1369.7929	4.1139	300	11	0.88	5.331	24.232	0.52	2	High	0.058	
**LF_WT**		34	46	EGSALGKQLNLKL	1369.7929	4.1199	3000	11	5.7	6.279	28.54	0.02	2	High	0.002	
**LF_WT**		34	46	EGSALGKQLNLKL	1369.7929	4.1184	9000	11	9.193	6.449	29.313	0.913	2	High	0.102	
**LF_WT**		36	46	SALGKQLNLKL	1183.7289	4.0669	0	9	0	0	0	n/a	2	High	0	
**LF_WT**		36	46	SALGKQLNLKL	1183.7289	4.0356	30	9	0.058	3.898	14.435	0.456	2	Medium	0.051	
**LF_WT**		36	46	SALGKQLNLKL	1183.7289	4.0509	300	9	0.557	4.648	17.216	0.912	2	Medium	0.101	
**LF_WT**		36	46	SALGKQLNLKL	1183.7289	4.0594	3000	9	4.073	5.596	20.724	0.376	2	Medium	0.042	
**LF_WT**		36	46	SALGKQLNLKL	1183.7289	4.0579	9000	9	7.213	5.72	21.183	0.938	2	Medium	0.104	
**LF_WT**		38	44	LGKQLNL	784.4807	3.4237	0	5	0	0	0	n/a	2	Medium	0	
**LF_WT**		38	44	LGKQLNL	784.4807	3.4151	30	5	0.038	1.8	18.004	0.278	2	Medium	0.031	
**LF_WT**		38	44	LGKQLNL	784.4807	3.41	300	5	0.367	2.213	22.134	0.093	2	Medium	0.01	
**LF_WT**		38	44	LGKQLNL	784.4807	3.4257	3000	5	2.565	2.905	29.054	0.048	2	Medium	0.005	
**LF_WT**		38	44	LGKQLNL	784.4807	3.3996	9000	5	4.246	3.024	30.236	0.339	2	Medium	0.038	
**LF_WT**		38	46	LGKQLNLKL	1025.6597	3.7689	0	7	0	0	0	n/a	2	High	0	
**LF_WT**		38	46	LGKQLNLKL	1025.6597	3.7656	30	7	0.046	2.635	18.823	0.674	2	High	0.075	
**LF_WT**		38	46	LGKQLNLKL	1025.6597	3.7599	300	7	0.444	3.268	23.344	0.416	2	High	0.046	
**LF_WT**		38	46	LGKQLNLKL	1025.6597	3.7633	3000	7	3.208	4.135	29.537	0.138	2	High	0.015	
**LF_WT**		38	46	LGKQLNLKL	1025.6597	3.7601	9000	7	5.608	4.265	30.463	0.52	2	High	0.058	
**LF_WT**		39	44	GKQLNL	671.3966	3.0498	0	4	0	0	0	n/a	2	High	0	
**LF_WT**		39	44	GKQLNL	671.3966	3.0407	30	4	0.027	1.527	38.179	0.385	2	High	0.043	
**LF_WT**		39	44	GKQLNL	671.3966	3.0333	300	4	0.263	1.803	45.078	0.219	2	Medium	0.024	
**LF_WT**		39	44	GKQLNL	671.3966	3.0378	3000	4	1.898	2.442	61.044	0.053	2	Medium	0.006	
**LF_WT**		39	44	GKQLNL	671.3966	3.0366	9000	4	3.283	2.535	63.386	0.381	2	Medium	0.042	
**LF_WT**		39	46	GKQLNLKL	912.5757	3.5529	0	6	0	0	0	n/a	2	Medium	0	
**LF_WT**		39	46	GKQLNLKL	912.5757	3.5423	30	6	0.035	2.337	38.954	0.459	2	High	0.051	
**LF_WT**		39	46	GKQLNLKL	912.5757	3.5383	300	6	0.34	2.819	46.981	0.213	2	High	0.024	

**LF_WT**		39	46	GKQLNLKL	912.5757	3.5369	3000	6	2.541	3.649	60.808	0.164	2	High	0.018	
**LF_WT**		39	46	GKQLNLKL	912.5757	3.5382	9000	6	4.644	3.757	62.614	0.523	2	High	0.058	
**LF_WT**		45	50	KLLDNW	787.4228	4.9085	0	4	0	0	0	n/a	2	Medium	0	
**LF_WT**		45	50	KLLDNW	787.4228	4.5073	30	4	0.08	2.096	52.405	0.167	2	Medium	0.019	
**LF_WT**		45	50	KLLDNW	787.4228	4.4963	300	4	0.674	2.111	52.776	0.094	2	Medium	0.01	
**LF_WT**		45	50	KLLDNW	787.4228	4.4983	3000	4	2.557	2.185	54.622	0.016	2	Medium	0.002	
**LF_WT**		45	50	KLLDNW	787.4228	4.4972	9000	4	3.449	2.181	54.532	0.367	2	Medium	0.041	
**LF_WT**		45	51	KLLDNWD	902.4498	4.2065	0	5	0	0	0	n/a	2	High	0	
**LF_WT**		45	51	KLLDNWD	902.4498	4.1748	30	5	0.098	2.441	24.408	0.301	2	Medium	0.034	
**LF_WT**		45	51	KLLDNWD	902.4498	4.1606	300	5	0.84	2.399	23.985	0.173	2	Medium	0.019	
**LF_WT**		45	51	KLLDNWD	902.4498	4.1854	3000	5	3.395	2.534	25.342	0.285	2	Medium	0.032	
**LF_WT**		45	51	KLLDNWD	902.4498	4.1916	9000	5	4.445	2.514	25.144	0.145	2	Medium	0.016	
**LF_WT**		45	56	KLLDNWDSVTST	1377.6776	4.2438	0	10	0	0	0	n/a	2	High	0	
**LF_WT**		45	56	KLLDNWDSVTST	1377.6776	4.2215	30	10	0.173	5.428	27.14	0.286	2	Medium	0.032	
**LF_WT**		45	56	KLLDNWDSVTST	1377.6776	4.2314	300	10	1.499	5.379	26.896	0.261	2	Medium	0.029	
**LF_WT**		45	56	KLLDNWDSVTST	1377.6776	4.2437	3000	10	6.315	5.612	28.061	0.407	2	Medium	0.045	
**LF_WT**		45	56	KLLDNWDSVTST	1377.6776	4.2435	9000	10	8.564	5.573	27.863	1.019	2	Medium	0.113	
**LF_WT**		47	56	LDNWDSVTST	1136.4986	3.954	0	8	0	0	0	n/a	2	High	0	
**LF_WT**		47	56	LDNWDSVTST	1136.4986	3.9517	30	8	0.154	4.813	60.156	0.032	2	High	0.004	
**LF_WT**		47	56	LDNWDSVTST	1136.4986	3.9368	300	8	1.317	4.539	56.733	0.281	2	High	0.031	
**LF_WT**		47	56	LDNWDSVTST	1136.4986	3.9479	3000	8	5.25	4.638	57.975	0.12	2	High	0.013	
**LF_WT**		47	56	LDNWDSVTST	1136.4986	3.9449	9000	8	6.987	4.635	57.943	0.559	2	High	0.062	
**LF_WT**		50	56	WDSVTST	794.3446	3.0478	0	5	0	0	0	n/a	2	Medium	0	
**LF_WT**		50	56	WDSVTST	794.3446	3.0434	30	5	0.075	3.105	62.092	0.347	2	Medium	0.039	
**LF_WT**		50	56	WDSVTST	794.3446	3.0516	300	5	0.659	2.855	57.099	0.262	2	Medium	0.029	
**LF_WT**		50	56	WDSVTST	794.3446	3.0611	3000	5	2.92	2.897	57.939	0.12	2	Medium	0.013	
**LF_WT**		50	56	WDSVTST	794.3446	3.0575	9000	5	4.119	2.893	57.852	0.428	2	Medium	0.048	
**LF_WT**		51	56	DSVTST	608.2653	1.4857	0	4	0	0	0	n/a	2	High	0	
**LF_WT**		51	56	DSVTST	608.2653	1.5215	30	4	0.032	2.539	63.473	0.213	2	High	0.024	
**LF_WT**		51	56	DSVTST	608.2653	1.4716	300	4	0.305	2.39	59.749	0.271	2	High	0.03	
**LF_WT**		51	56	DSVTST	608.2653	1.4786	3000	4	1.933	2.443	61.067	0.019	2	High	0.002	
**LF_WT**		51	56	DSVTST	608.2653	1.478	9000	4	3.119	2.429	60.716	0.371	2	High	0.041	
**LF_WT**		51	57	DSVTSTF	755.3337	3.6026	0	5	0	0	0	n/a	2	High	0	
**LF_WT**		51	57	DSVTSTF	755.3337	3.5933	30	5	0.037	3.211	64.218	0.08	2	High	0.009	
**LF_WT**		51	57	DSVTSTF	755.3337	3.5893	300	5	0.356	2.989	59.77	0.27	2	High	0.03	
**LF_WT**		51	57	DSVTSTF	755.3337	3.5891	3000	5	2.34	3.069	61.371	0.129	2	High	0.014	
**LF_WT**		51	57	DSVTSTF	755.3337	3.5843	9000	5	3.91	3.066	61.311	0.261	2	High	0.029	
**LF_WT**		57	70	FSKLREQLGPVTQE	1630.8679	3.1865	0	11	0	0	0	n/a	2	High	0	
**LF_WT**		57	70	FSKLREQLGPVTQE	1630.8679	3.1808	30	11	0.085	3.008	13.673	0.088	2	Medium	0.01	
**LF_WT**		57	70	FSKLREQLGPVTQE	1630.8679	3.1998	300	11	0.814	3.718	16.9	0.47	2	Medium	0.052	
**LF_WT**		57	70	FSKLREQLGPVTQE	1630.8679	3.1712	3000	11	5.447	5.341	24.277	0.64	2	Medium	0.071	
**LF_WT**		57	70	FSKLREQLGPVTQE	1630.8679	3.1995	9000	11	8.858	5.894	26.791	0.476	2	Medium	0.053	
**LF_WT**		57	71	FSKLREQLGPVTQEF	1777.9363	4.1481	0	12	0	0	0	n/a	2	High	0	
**LF_WT**		57	71	FSKLREQLGPVTQEF	1777.9363	4.1419	30	12	0.093	3.064	12.765	0.742	2	High	0.083	
**LF_WT**		57	71	FSKLREQLGPVTQEF	1777.9363	4.143	300	12	0.892	3.89	16.208	0.555	2	High	0.062	
**LF_WT**		57	71	FSKLREQLGPVTQEF	1777.9363	4.1375	3000	12	6.004	5.497	22.905	0.038	2	High	0.004	
**LF_WT**		57	71	FSKLREQLGPVTQEF	1777.9363	4.1336	9000	12	9.771	6.263	26.097	0.991	2	High	0.11	
**LF_WT**		58	70	SKLREQLGPVTQE	1483.7995	2.757	0	10	0	0	0	n/a	2	High	0	
**LF_WT**		58	70	SKLREQLGPVTQE	1483.7995	2.7886	30	10	0.076	2.478	12.392	0.614	2	High	0.068	
**LF_WT**		58	70	SKLREQLGPVTQE	1483.7995	2.7701	300	10	0.725	3.251	16.257	0.766	2	High	0.085	
**LF_WT**		58	70	SKLREQLGPVTQE	1483.7995	2.7361	3000	10	4.841	4.944	24.722	0.078	2	High	0.009	
**LF_WT**		58	70	SKLREQLGPVTQE	1483.7995	2.7723	9000	10	7.919	5.542	27.71	0.634	2	High	0.071	
**LF_WT**		58	71	SKLREQLGPVTQEF	1630.8679	3.7752	0	11	0	0	0	n/a	2	Medium	0	
**LF_WT**		58	71	SKLREQLGPVTQEF	1630.8679	3.7611	30	11	0.084	2.816	10.649	0.068	4	Medium	0.043	
**LF_WT**		58	71	SKLREQLGPVTQEF	1630.8679	3.7574	300	11	0.803	3.632	13.74	0.052	4	Medium	0.032	
**LF_WT**		58	71	SKLREQLGPVTQEF	1630.8679	3.7595	3000	11	5.397	5.264	19.97	0.075	4	Medium	0.047	
**LF_WT**		58	71	SKLREQLGPVTQEF	1630.8679	3.7588	9000	11	8.832	6.017	22.845	0.218	4	Medium	0.137	
**LF_WT**		61	71	REQLGPVTQEF	1302.6568	4.0059	0	8	0	0	0	n/a	2	High	0	
**LF_WT**		61	71	REQLGPVTQEF	1302.6568	3.9528	30	8	0.062	2.422	15.139	0.455	2	High	0.051	
**LF_WT**		61	71	REQLGPVTQEF	1302.6568	3.9724	300	8	0.589	2.883	18.018	0.245	2	High	0.027	
**LF_WT**		61	71	REQLGPVTQEF	1302.6568	3.9745	3000	8	3.963	3.699	23.119	0.03	2	High	0.003	
**LF_WT**		61	71	REQLGPVTQEF	1302.6568	3.9678	9000	8	6.444	4.038	25.235	0.65	2	High	0.072	
**LF_WT**		72	91	WDNLEKETEGLRQEMSKDLE	2449.1431	5.1444	0	18	0	0	0	n/a	2	High	0	
**LF_WT**		72	91	WDNLEKETEGLRQEMSKDLE	2449.1431	5.1287	30	18	0.241	2.108	4.831	0.324	4	High	0.204	
**LF_WT**		72	91	WDNLEKETEGLRQEMSKDLE	2449.1431	5.1181	300	18	2.187	3.582	8.286	0.15	4	High	0.094	
**LF_WT**		72	91	WDNLEKETEGLRQEMSKDLE	2449.1431	5.1056	3000	18	11.683	5.84	13.474	0.3	4	High	0.188	
**LF_WT**		72	91	WDNLEKETEGLRQEMSKDLE	2449.1431	5.0946	9000	18	16.731	7.191	16.586	0.321	4	High	0.202	

LF_WT		72	92	WDNLEKETEGLRQEMSKDLEE	2578.1857	5.1911	0	19	0	0	0	n/a	2	High	0	
LF_WT		72	92	WDNLEKETEGLRQEMSKDLEE	2578.1857	5.1647	30	19	0.261	2.245	4.869	0.279	4	High	0.175	
LF_WT		72	92	WDNLEKETEGLRQEMSKDLEE	2578.1857	5.1861	300	19	2.369	3.327	7.295	0.139	4	High	0.087	
LF_WT		72	92	WDNLEKETEGLRQEMSKDLEE	2578.1857	5.1459	3000	19	12.549	6.059	13.202	0.444	4	Medium	0.279	
LF_WT		72	92	WDNLEKETEGLRQEMSKDLEE	2578.1857	5.1506	9000	19	17.729	7.236	15.861	0.287	4	Medium	0.18	
LF_WT		72	103	WDNLEKETEGLRQEMSKDLEEVKAKVQPYLI	3834.8622	5.9076	0	29	0	0	0	n/a	2	Medium	0	
LF_WT		72	103	WDNLEKETEGLRQEMSKDLEEVKAKVQPYLI	3834.8622	5.9274	30	29	0.4	0.702	0.605	2.549	2	High	0.284	
LF_WT		72	103	WDNLEKETEGLRQEMSKDLEEVKAKVQPYLI	3834.8622	5.9245	300	29	3.492	2.389	2.06	0.85	2	High	0.095	
LF_WT		72	103	WDNLEKETEGLRQEMSKDLEEVKAKVQPYLI	3834.8622	5.9152	3000	29	17.379	4.425	3.814	0.886	2	High	0.099	
LF_WT		72	103	WDNLEKETEGLRQEMSKDLEEVKAKVQPYLI	3834.8622	5.8594	9000	29	25.476	7.107	6.127	0.908	2	High	0.101	
LF_WT		77	92	KETEGLRQEMSKDLEE	1920.9099	3.4546	0	14	0	0	0	n/a	2	High	0	
LF_WT		77	92	KETEGLRQEMSKDLEE	1920.9099	3.4212	30	14	0.173	2.774	9.907	1.104	2	High	0.123	
LF_WT		77	92	KETEGLRQEMSKDLEE	1920.9099	3.4255	300	14	1.615	3.371	12.039	0.335	2	High	0.037	
LF_WT		77	92	KETEGLRQEMSKDLEE	1920.9099	3.4426	3000	14	9.321	4.457	15.919	0.272	2	High	0.03	
LF_WT		77	92	KETEGLRQEMSKDLEE	1920.9099	3.4321	9000	14	13.171	5.429	19.388	0.719	2	High	0.08	
LF_WT		83	92	RQEMSKDLEE	1263.5765	2.2752	0	8	0	0	0	n/a	2	Medium	0	
LF_WT		83	92	RQEMSKDLEE	1263.5765	2.2881	30	8	0.114	0.859	5.371	0.103	2	Medium	0.011	
LF_WT		83	92	RQEMSKDLEE	1263.5765	2.2745	300	8	1.052	0.935	5.846	0.116	2	Medium	0.013	
LF_WT		83	92	RQEMSKDLEE	1263.5765	2.2632	3000	8	5.659	1.586	9.91	0.114	2	Medium	0.013	
LF_WT		83	92	RQEMSKDLEE	1263.5765	2.2781	9000	8	7.634	2.094	13.085	0.237	2	Medium	0.026	
LF_WT		87	92	SKDLEE	719.3337	1.6989	0	4	0	0	0	n/a	2	High	0	
LF_WT		87	92	SKDLEE	719.3337	1.702	30	4	0.064	0.385	9.616	0.095	2	High	0.011	
LF_WT		87	92	SKDLEE	719.3337	1.6841	300	4	0.579	0.405	10.133	0.04	2	High	0.004	
LF_WT		87	92	SKDLEE	719.3337	1.6975	3000	4	2.821	0.562	14.04	0.308	2	High	0.034	
LF_WT		87	92	SKDLEE	719.3337	1.6946	9000	4	3.749	0.766	19.145	0.177	2	High	0.02	
LF_WT		90	103	LEEVKAKVQPYLDD	1645.8563	3.2372	0	11	0	0	0	n/a	2	High	0	
LF_WT		90	103	LEEVKAKVQPYLDD	1645.8563	3.2202	30	11	0.159	0.641	2.916	0.23	2	High	0.026	
LF_WT		90	103	LEEVKAKVQPYLDD	1645.8563	3.2556	300	11	1.305	0.768	3.491	0.273	2	High	0.03	
LF_WT		90	103	LEEVKAKVQPYLDD	1645.8563	3.2725	3000	11	5.696	1.667	7.578	0.26	2	High	0.029	
LF_WT		90	103	LEEVKAKVQPYLDD	1645.8563	3.2262	9000	11	8.744	2.734	12.426	0.481	2	High	0.054	
LF_WT		91	103	EEVKAKVQPYLDD	1532.7722	2.9623	0	10	0	0	0	n/a	2	High	0	
LF_WT		91	103	EEVKAKVQPYLDD	1532.7722	2.9759	30	10	0.139	0.565	2.824	0.063	2	Medium	0.007	
LF_WT		91	103	EEVKAKVQPYLDD	1532.7722	2.9957	300	10	1.123	0.716	3.581	0.536	2	High	0.06	
LF_WT		91	103	EEVKAKVQPYLDD	1532.7722	2.9628	3000	10	4.829	1.895	9.476	0.748	2	Medium	0.083	
LF_WT		91	103	EEVKAKVQPYLDD	1532.7722	2.9552	9000	10	7.747	2.424	12.12	0.592	2	Medium	0.066	
LF_WT		92	103	EVKAKVQPYLDD	1403.7296	2.8881	0	9	0	0	0	n/a	2	High	0	
LF_WT		92	103	EVKAKVQPYLDD	1403.7296	2.8543	30	9	0.136	0.663	3.682	0.687	2	High	0.076	
LF_WT		92	103	EVKAKVQPYLDD	1403.7296	2.8037	300	9	1.091	0.958	5.325	0.621	2	High	0.069	
LF_WT		92	103	EVKAKVQPYLDD	1403.7296	2.8216	3000	9	4.55	1.888	10.491	0.163	2	High	0.018	
LF_WT		92	103	EVKAKVQPYLDD	1403.7296	2.7998	9000	9	7.122	2.446	13.588	0.464	2	High	0.052	
LF_WT		93	103	VKAKVQPYLDD	1274.6871	2.719	0	8	0	0	0	n/a	2	High	0	
LF_WT		93	103	VKAKVQPYLDD	1274.6871	2.6562	30	8	0.131	0.713	4.454	0.259	2	High	0.029	
LF_WT		93	103	VKAKVQPYLDD	1274.6871	2.68	300	8	1.047	0.929	5.804	0.005	2	High	0.001	
LF_WT		93	103	VKAKVQPYLDD	1274.6871	2.684	3000	8	4.191	1.77	11.063	0.1	2	High	0.011	
LF_WT		93	103	VKAKVQPYLDD	1274.6871	2.6884	9000	8	6.384	2.301	14.384	0.465	2	High	0.052	
LF_WT		93	104	VKAKVQPYLDDF	1421.7555	4.3294	0	9	0	0	0	n/a	2	High	0	
LF_WT		93	104	VKAKVQPYLDDF	1421.7555	4.3562	30	9	0.144	0.441	2.451	0.142	2	High	0.016	
LF_WT		93	104	VKAKVQPYLDDF	1421.7555	4.3012	300	9	1.168	0.901	5.004	0.146	2	High	0.016	
LF_WT		93	104	VKAKVQPYLDDF	1421.7555	4.3515	3000	9	4.915	1.747	9.703	0.613	2	High	0.068	
LF_WT		93	104	VKAKVQPYLDDF	1421.7555	4.3133	9000	9	7.363	2.306	12.81	0.526	2	High	0.059	
LF_WT		96	103	KVQPYLDD	976.4866	2.9582	0	5	0	0	0	n/a	2	High	0	
LF_WT		96	103	KVQPYLDD	976.4866	2.9384	30	5	0.111	0.462	4.621	0.31	2	High	0.034	
LF_WT		96	103	KVQPYLDD	976.4866	2.9271	300	5	0.848	0.5	5.001	0.205	2	High	0.023	
LF_WT		96	103	KVQPYLDD	976.4866	2.9308	3000	5	2.803	0.939	9.393	0.242	2	High	0.027	
LF_WT		96	103	KVQPYLDD	976.4866	2.9319	9000	5	4.053	1.239	12.385	0.218	2	High	0.024	
LF_WT		104	111	FQKKWQEE	1121.5506	2.5319	0	6	0	0	0	n/a	2	High	0	
LF_WT		104	111	FQKKWQEE	1121.5506	2.4878	30	6	0.058	0.258	4.295	0.475	2	High	0.053	
LF_WT		104	111	FQKKWQEE	1121.5506	2.4796	300	6	0.55	0.436	7.265	0.242	2	Medium	0.027	
LF_WT		104	111	FQKKWQEE	1121.5506	2.5116	3000	6	3.439	1.246	20.771	0.041	2	High	0.005	
LF_WT		104	111	FQKKWQEE	1121.5506	2.5452	9000	6	5.306	1.809	30.149	0.323	2	High	0.036	
LF_WT		113	124	ELYRQKVEPLRA	1500.8413	3.1637	0	9	0	0	0	n/a	2	High	0	
LF_WT		113	124	ELYRQKVEPLRA	1500.8413	3.1224	30	9	0.06	4.888	18.105	0.125	2	High	0.014	
LF_WT		113	124	ELYRQKVEPLRA	1500.8413	3.15	300	9	0.574	5.027	18.617	0.488	2	High	0.054	
LF_WT		113	124	ELYRQKVEPLRA	1500.8413	3.1609	3000	9	4.134	5.306	19.651	0.107	2	High	0.012	
LF_WT		113	124	ELYRQKVEPLRA	1500.8413	3.1574	9000	9	7.172	5.366	19.875	0.736	2	High	0.082	
LF_WT		113	126	ELYRQKVEPLRAEL	1742.9679	3.5034	0	11	0	0	0	n/a	2	Medium	0	
LF_WT		113	126	ELYRQKVEPLRAEL	1742.9679	3.5016	30	11	0.074	5.208	15.782	0.447	2	Medium	0.05	

**LF_WT**		113	126	ELYRQKVEPLRAEL	1742.9679	3.4877	300	11	0.718	5.672	17.188	1.099	2	Medium	0.122	
**LF_WT**		113	126	ELYRQKVEPLRAEL	1742.9679	3.5002	3000	11	5.145	6.442	19.523	0.036	2	Medium	0.004	
**LF_WT**		113	126	ELYRQKVEPLRAEL	1742.9679	3.461	9000	11	8.871	6.648	20.146	0.422	2	Medium	0.047	
**LF_WT**		114	124	LYRQKVEPLRA	1371.7987	2.3928	0	8	0	0	0	n/a	2	High	0	
**LF_WT**		114	124	LYRQKVEPLRA	1371.7987	2.4318	30	8	0.055	3.951	16.46	0.254	2	High	0.028	
**LF_WT**		114	124	LYRQKVEPLRA	1371.7987	2.4464	300	8	0.529	4.276	17.817	0.505	2	High	0.056	
**LF_WT**		114	124	LYRQKVEPLRA	1371.7987	2.4391	3000	8	3.763	4.691	19.547	0.156	2	High	0.017	
**LF_WT**		114	124	LYRQKVEPLRA	1371.7987	2.4205	9000	8	6.42	4.877	20.321	0.87	2	Medium	0.097	
**LF_WT**		114	125	LYRQKVEPLRAE	1500.8413	2.6779	0	9	0	0	0	n/a	2	Medium	0	
**LF_WT**		114	125	LYRQKVEPLRAE	1500.8413	3.1249	30	9	0.065	4.945	18.313	0.169	2	High	0.019	
**LF_WT**		114	125	LYRQKVEPLRAE	1500.8413	3.15	300	9	0.628	5.087	18.839	0.488	2	High	0.054	
**LF_WT**		114	125	LYRQKVEPLRAE	1500.8413	3.1609	3000	9	4.41	5.366	19.873	0.107	2	High	0.012	
**LF_WT**		114	125	LYRQKVEPLRAE	1500.8413	3.1574	9000	9	7.376	5.426	20.097	0.736	2	High	0.082	
**LF_WT**		114	126	LYRQKVEPLRAEL	1613.9253	3.3283	0	10	0	0	0	n/a	2	High	0	
**LF_WT**		114	126	LYRQKVEPLRAEL	1613.9253	3.3154	30	10	0.07	5.225	21.732	0.099	4	Medium	0.062	
**LF_WT**		114	126	LYRQKVEPLRAEL	1613.9253	3.3295	300	10	0.672	5.566	23.166	0.097	4	High	0.061	
**LF_WT**		114	126	LYRQKVEPLRAEL	1613.9253	3.313	3000	10	4.773	6.034	25.099	0.196	4	Medium	0.123	
**LF_WT**		114	126	LYRQKVEPLRAEL	1613.9253	3.2916	9000	10	8.119	6.239	25.878	0.456	4	Medium	0.287	
**LF_WT**		115	124	YRQKVEPLRA	1258.7146	2.0872	0	7	0	0	0	n/a	2	High	0	
**LF_WT**		115	124	YRQKVEPLRA	1258.7146	2.0877	30	7	0.048	3.794	18.065	0.044	2	High	0.005	
**LF_WT**		115	124	YRQKVEPLRA	1258.7146	2.0743	300	7	0.46	4.003	19.064	0.354	2	High	0.039	
**LF_WT**		115	124	YRQKVEPLRA	1258.7146	2.0791	3000	7	3.251	4.244	20.211	0.04	2	High	0.004	
**LF_WT**		115	124	YRQKVEPLRA	1258.7146	2.0786	9000	7	5.537	4.293	20.444	0.617	2	High	0.069	
**LF_WT**		115	126	YRQKVEPLRAEL	1500.8413	3.1129	0	9	0	0	0	n/a	2	High	0	
**LF_WT**		115	126	YRQKVEPLRAEL	1500.8413	3.1008	30	9	0.063	4.847	15.726	0.079	4	High	0.049	
**LF_WT**		115	126	YRQKVEPLRAEL	1500.8413	3.119	300	9	0.603	4.992	16.193	0.096	4	High	0.06	
**LF_WT**		115	126	YRQKVEPLRAEL	1500.8413	3.1575	3000	9	4.262	5.264	17.079	0.08	4	High	0.05	
**LF_WT**		115	126	YRQKVEPLRAEL	1500.8413	3.1549	9000	9	7.235	5.319	17.261	0.138	4	High	0.087	
**LF_WT**		115	147	YRQKVEPLRAELQEGARQKLHELQEKLSPLG	3901.081	4.5416	0	29	0	0	0	n/a	2	High	0	
**LF_WT**		115	147	YRQKVEPLRAELQEGARQKLHELQEKLSPLG	3901.081	4.5048	30	29	0.313	16.155	11.141	2.329	2	High	0.259	
**LF_WT**		115	147	YRQKVEPLRAELQEGARQKLHELQEKLSPLG	3901.081	4.5128	300	29	2.883	15.87	10.945	1.66	2	High	0.185	
**LF_WT**		115	147	YRQKVEPLRAELQEGARQKLHELQEKLSPLG	3901.081	4.5199	3000	29	16.5	16.302	11.243	0.794	2	High	0.088	
**LF_WT**		115	147	YRQKVEPLRAELQEGARQKLHELQEKLSPLG	3901.081	4.5073	9000	29	24.896	16.593	11.443	2.954	2	High	0.329	
**LF_WT**		115	158	YRQKVEPLRAELQEGARQKLHELQEKLSPLG	5179.7174	4.5296	0	40	0	0	0	n/a	2	High	0	
**LF_WT**		115	158	YRQKVEPLRAELQEGARQKLHELQEKLSPLG	5179.7174	4.4602	30	40	0.506	17.874	6.384	0.125	2	High	0.014	
**LF_WT**		115	158	YRQKVEPLRAELQEGARQKLHELQEKLSPLG	5179.7174	4.495	300	40	4.628	17.768	6.346	0.913	2	High	0.102	
**LF_WT**		115	158	YRQKVEPLRAELQEGARQKLHELQEKLSPLG	5179.7174	4.4907	3000	40	24.825	19.44	6.943	0.152	2	High	0.017	
**LF_WT**		115	158	YRQKVEPLRAELQEGARQKLHELQEKLSPLG	5179.7174	4.4731	9000	40	35.51	20.279	7.242	1.097	2	High	0.122	
**LF_WT**		125	136	ELQEGARQKLHE	1436.7372	1.9401	0	10	0	0	0	n/a	2	Medium	0	
**LF_WT**		125	136	ELQEGARQKLHE	1436.7372	1.9194	30	10	0.157	5.296	26.479	0.082	2	Medium	0.009	
**LF_WT**		125	136	ELQEGARQKLHE	1436.7372	1.8616	300	10	1.395	5.135	25.676	0.415	2	Medium	0.046	
**LF_WT**		125	136	ELQEGARQKLHE	1436.7372	1.8621	3000	10	6.71	5.322	26.608	0.439	2	Medium	0.049	
**LF_WT**		125	136	ELQEGARQKLHE	1436.7372	1.848	9000	10	9.173	5.447	27.236	0.626	2	Medium	0.07	
**LF_WT**		125	139	ELQEGARQKLHELQE	1806.9224	2.9609	0	13	0	0	0	n/a	2	High	0	
**LF_WT**		125	139	ELQEGARQKLHELQE	1806.9224	2.9631	30	13	0.181	6.734	25.9	0.77	2	High	0.086	
**LF_WT**		125	139	ELQEGARQKLHELQE	1806.9224	2.9025	300	13	1.624	6.876	26.446	0.374	2	High	0.042	
**LF_WT**		125	139	ELQEGARQKLHELQE	1806.9224	2.932	3000	13	8.264	6.927	26.641	0.304	2	High	0.034	
**LF_WT**		125	139	ELQEGARQKLHELQE	1806.9224	2.9045	9000	13	11.729	7.165	27.56	0.408	2	High	0.045	
**LF_WT**		125	146	ELQEGARQKLHELQEKLSPLGE	2531.3344	4.3371	0	19	0	0	0	n/a	2	High	0	
**LF_WT**		125	146	ELQEGARQKLHELQEKLSPLGE	2531.3344	4.3041	30	19	0.23	10.745	14.989	0.797	4	High	0.501	
**LF_WT**		125	146	ELQEGARQKLHELQEKLSPLGE	2531.3344	4.2815	300	19	2.098	10.206	14.263	0.375	4	High	0.236	
**LF_WT**		125	146	ELQEGARQKLHELQEKLSPLGE	2531.3344	4.2817	3000	19	11.371	10.517	14.77	0.256	4	High	0.161	
**LF_WT**		125	146	ELQEGARQKLHELQEKLSPLGE	2531.3344	4.2892	9000	19	16.663	10.486	14.78	0.443	4	Medium	0.278	
**LF_WT**		125	148	ELQEGARQKLHELQEKLSPLGEEM	2791.4174	4.5589	0	21	0	0	0	n/a	2	High	0	
**LF_WT**		125	148	ELQEGARQKLHELQEKLSPLGEEM	2791.4174	4.5182	30	21	0.262	12.407	17.226	0.713	4	High	0.448	
**LF_WT**		125	148	ELQEGARQKLHELQEKLSPLGEEM	2791.4174	4.5229	300	21	2.393	11.379	15.855	0.549	4	High	0.345	
**LF_WT**		125	148	ELQEGARQKLHELQEKLSPLGEEM	2791.4174	4.5268	3000	21	12.937	11.533	16.035	0.171	4	Medium	0.107	
**LF_WT**		125	148	ELQEGARQKLHELQEKLSPLGEEM	2791.4174	4.5403	9000	21	18.634	11.386	15.833	0.184	4	High	0.116	
**LF_WT**		125	158	ELQEGARQKLHELQEKLSPLGEEMRDRARAI	3939.0133	4.3038	0	31	0	0	0	n/a	2	High	0	
**LF_WT**		125	158	ELQEGARQKLHELQEKLSPLGEEMRDRARAI	3939.0133	4.2565	30	31	0.443	12.849	9.323	0.49	4	High	0.308	
**LF_WT**		125	158	ELQEGARQKLHELQEKLSPLGEEMRDRARAI	3939.0133	4.2568	300	31	4.025	12.528	9.087	0.318	4	High	0.2	
**LF_WT**		125	158	ELQEGARQKLHELQEKLSPLGEEMRDRARAI	3939.0133	4.2608	3000	31	20.563	13.897	10.081	0.13	4	High	0.082	
**LF_WT**		125	158	ELQEGARQKLHELQEKLSPLGEEMRDRARAI	3939.0133	4.2569	9000	31	28.274	14.598	10.59	0.316	4	High	0.198	
**LF_WT**		125	159	ELQEGARQKLHELQEKLSPLGEEMRDRARAI	4052.0974	4.5187	0	32	0	0	0	n/a	2	High	0	
**LF_WT**		125	159	ELQEGARQKLHELQEKLSPLGEEMRDRARAI	4052.0974	4.4705	30	32	0.447	12.984	8.115	1.578	2	High	0.176	
**LF_WT**		125	159	ELQEGARQKLHELQEKLSPLGEEMRDRARAI	4052.0974	4.476	300	32	4.066	12.692	7.932	1.976	2	High	0.22	
**LF_WT**		125	159	ELQEGARQKLHELQEKLSPLGEEMRDRARAI	4052.0974	4.4844	3000	32	20.902	14.05	8.781	0.227	2	High	0.025	
**LF_WT**		125	159	ELQEGARQKLHELQEKLSPLGEEMRDRARAI	4052.0974	4.4769	9000	32	28.986	14.853	9.283	1.474	2	High	0.164	
**LF_WT**		126	158	LQEGARQKLHELQEKLSPLGEEMRDRARAH	3809.9707	4.3801	0	30	0	0	0	n/a	2	High	0	
**LF_WT**		126	158	LQEGARQKLHELQEKLSPLGEEMRDRARAH	3809.9707	4.3218	30	30	0.437	11.436	7.624	2.101	2	High	0.234	
**LF_WT**		126	158	LQEGARQKLHELQEKLSPLGEEMRDRARAH	3809.9707	4.3176	300	30	3.968	11.357	7.571	1.501	2	High	0.167	

**LF_WT**		126	158	LQEGARQKLHELQEKLSPLGEEMRDRARAH	3809.9707	4.3159	3000	30	20.117	12.865	8.576	0.513		2	High	0.057
**LF_WT**		126	158	LQEGARQKLHELQEKLSPLGEEMRDRARAH	3809.9707	4.3171	9000	30	27.444	13.567	9.045	1.336		2	High	0.149
**LF_WT**		127	139	QEGARQKLHELQE	1564.7958	2.4052	0	11	0	0	0	n/a		2	High	0
**LF_WT**		127	139	QEGARQKLHELQE	1564.7958	2.4039	30	11	0.161	5.704	25.927	0.226		2	High	0.025
**LF_WT**		127	139	QEGARQKLHELQE	1564.7958	2.3639	300	11	1.44	5.652	25.691	0.314		2	Medium	0.035
**LF_WT**		127	139	QEGARQKLHELQE	1564.7958	2.3831	3000	11	7.073	5.629	25.588	0.41		2	Medium	0.046
**LF_WT**		127	139	QEGARQKLHELQE	1564.7958	2.3759	9000	11	9.916	5.806	26.392	0.24		2	High	0.027
**LF_WT**		127	146	QEGARQKLHELQEKLSPLGE	2289.2077	4.0348	0	17	0	0	0	n/a		2	High	0
**LF_WT**		127	146	QEGARQKLHELQEKLSPLGE	2289.2077	4.0585	30	17	0.211	9.404	18.439	0.287		2	Medium	0.032
**LF_WT**		127	146	QEGARQKLHELQEKLSPLGE	2289.2077	4.0029	300	17	1.913	9.052	17.748	0.116		2	High	0.013
**LF_WT**		127	146	QEGARQKLHELQEKLSPLGE	2289.2077	4.0164	3000	17	10.181	9.21	18.059	0.454		2	Medium	0.051
**LF_WT**		127	146	QEGARQKLHELQEKLSPLGE	2289.2077	4.0073	9000	17	14.85	9.251	18.14	0.268		2	High	0.03
**LF_WT**		127	147	QEGARQKLHELQEKLSPLGEE	2418.2503	4.0693	0	18	0	0	0	n/a		2	High	0
**LF_WT**		127	147	QEGARQKLHELQEKLSPLGEE	2418.2503	4.039	30	18	0.231	9.617	20.683	1.717		4	Medium	1.079
**LF_WT**		127	147	QEGARQKLHELQEKLSPLGEE	2418.2503	3.9857	300	18	2.095	9.527	19.706	0.611		4	Medium	0.384
**LF_WT**		127	147	QEGARQKLHELQEKLSPLGEE	2418.2503	3.995	3000	18	11.048	9.764	20.383	0.48		4	Medium	0.301
**LF_WT**		127	147	QEGARQKLHELQEKLSPLGEE	2418.2503	3.9951	9000	18	15.847	9.619	20.043	0.059		4	Medium	0.037
**LF_WT**		127	154	QEGARQKLHELQEKLSPLGEEMRDRARA	3274.6953	4.0969	0	25	0	0	0	n/a		2	Medium	0
**LF_WT**		127	154	QEGARQKLHELQEKLSPLGEEMRDRARA	3274.6953	4.0511	30	25	0.344	11.547	11.547	1.024		2	Medium	0.114
**LF_WT**		127	154	QEGARQKLHELQEKLSPLGEEMRDRARA	3274.6953	4.0648	300	25	3.121	11.303	11.303	1.089		2	Medium	0.121
**LF_WT**		127	154	QEGARQKLHELQEKLSPLGEEMRDRARA	3274.6953	4.0518	3000	25	16.178	12.649	12.649	0.268		2	Medium	0.03
**LF_WT**		127	154	QEGARQKLHELQEKLSPLGEEMRDRARA	3274.6953	4.0528	9000	25	22.604	13.054	13.054	1.22		2	Medium	0.136
**LF_WT**		127	158	QEGARQKLHELQEKLSPLGEEMRDRARAHV	3696.8867	4.1335	0	29	0	0	0	n/a		2	High	0
**LF_WT**		127	158	QEGARQKLHELQEKLSPLGEEMRDRARAHV	3696.8867	4.0586	30	29	0.424	11.444	7.892	1.117		2	High	0.124
**LF_WT**		127	158	QEGARQKLHELQEKLSPLGEEMRDRARAHV	3696.8867	4.0597	300	29	3.84	11.213	7.733	0.984		2	High	0.109
**LF_WT**		127	158	QEGARQKLHELQEKLSPLGEEMRDRARAHV	3696.8867	4.0556	3000	29	19.373	12.642	8.719	0.298		2	High	0.033
**LF_WT**		127	158	QEGARQKLHELQEKLSPLGEEMRDRARAHV	3696.8867	4.0528	9000	29	26.461	13.368	9.22	0.574		2	High	0.064
**LF_WT**		127	159	QEGARQKLHELQEKLSPLGEEMRDRARAHV	3809.9707	4.3523	0	30	0	0	0	n/a		2	High	0
**LF_WT**		127	159	QEGARQKLHELQEKLSPLGEEMRDRARAHV	3809.9707	4.3085	30	30	0.428	11.666	8.769	0.869		4	High	0.546
**LF_WT**		127	159	QEGARQKLHELQEKLSPLGEEMRDRARAHV	3809.9707	4.3246	300	30	3.881	11.37	8.532	0.352		4	High	0.221
**LF_WT**		127	159	QEGARQKLHELQEKLSPLGEEMRDRARAHV	3809.9707	4.3204	3000	30	19.712	12.854	9.638	0.099		4	High	0.062
**LF_WT**		127	159	QEGARQKLHELQEKLSPLGEEMRDRARAHV	3809.9707	4.321	9000	30	27.172	13.556	10.165	0.202		4	High	0.127
**LF_WT**		137	146	LQEKLSPLGE	1112.6077	3.4104	0	7	0	0	0	n/a		2	High	0
**LF_WT**		137	146	LQEKLSPLGE	1112.6077	3.4186	30	7	0.063	3.665	26.177	0.017		2	High	0.002
**LF_WT**		137	146	LQEKLSPLGE	1112.6077	3.3659	300	7	0.601	3.432	24.518	0.405		2	High	0.045
**LF_WT**		137	146	LQEKLSPLGE	1112.6077	3.3985	3000	7	3.852	3.588	25.626	0.201		2	High	0.022
**LF_WT**		137	146	LQEKLSPLGE	1112.6077	3.3842	9000	7	5.917	3.542	25.303	0.206		2	High	0.023
**LF_WT**		137	147	LQEKLSPLGEE	1241.6503	3.4355	0	8	0	0	0	n/a		2	Medium	0
**LF_WT**		137	147	LQEKLSPLGEE	1241.6503	3.3564	30	8	0.083	4.142	25.888	0.031		2	Medium	0.004
**LF_WT**		137	147	LQEKLSPLGEE	1241.6503	3.3715	300	8	0.783	3.826	23.913	0.173		2	Medium	0.019
**LF_WT**		137	147	LQEKLSPLGEE	1241.6503	3.4211	3000	8	4.718	3.976	24.849	0.1		2	Medium	0.011
**LF_WT**		137	147	LQEKLSPLGEE	1241.6503	3.4136	9000	8	6.915	3.938	24.611	0.278		2	Medium	0.031
**LF_WT**		137	158	LQEKLSPLGEEMRDRARAHVDA	2520.2867	3.315	0	19	0	0	0	n/a		2	High	0
**LF_WT**		137	158	LQEKLSPLGEEMRDRARAHVDA	2520.2867	3.3031	30	19	0.276	5.758	8.078	0.107		4	High	0.067
**LF_WT**		137	158	LQEKLSPLGEEMRDRARAHVDA	2520.2867	3.2855	300	19	2.528	6.068	8.554	0.363		4	High	0.228
**LF_WT**		137	158	LQEKLSPLGEEMRDRARAHVDA	2520.2867	3.2896	3000	19	13.043	7.194	10.097	0.03		4	High	0.019
**LF_WT**		137	158	LQEKLSPLGEEMRDRARAHVDA	2520.2867	3.2916	9000	19	17.528	7.809	10.957	0.118		4	High	0.074
**LF_WT**		137	159	LQEKLSPLGEEMRDRARAHVDAL	2633.3708	3.8225	0	20	0	0	0	n/a		2	Medium	0
**LF_WT**		137	159	LQEKLSPLGEEMRDRARAHVDAL	2633.3708	3.8179	30	20	0.28	5.927	5.927	0.899		2	High	0.1
**LF_WT**		137	159	LQEKLSPLGEEMRDRARAHVDAL	2633.3708	3.7852	300	20	2.569	6.072	6.072	0.855		2	Medium	0.095
**LF_WT**		137	159	LQEKLSPLGEEMRDRARAHVDAL	2633.3708	3.818	3000	20	13.382	7.29	7.29	0.939		2	Medium	0.105
**LF_WT**		137	159	LQEKLSPLGEEMRDRARAHVDAL	2633.3708	3.7906	9000	20	18.24	8.093	8.093	0.169		2	Medium	0.019
**LF_WT**		139	158	EKLSPLGEEMRDRARAHVDA	2279.1441	3.1679	0	17	0	0	0	n/a		2	Medium	0
**LF_WT**		139	158	EKLSPLGEEMRDRARAHVDA	2279.1441	3.1605	30	17	0.251	5.164	7.595	1.015		2	Medium	0.113
**LF_WT**		139	158	EKLSPLGEEMRDRARAHVDA	2279.1441	3.1592	300	17	2.292	5.42	7.971	0.142		2	Medium	0.016
**LF_WT**		139	158	EKLSPLGEEMRDRARAHVDA	2279.1441	3.1673	3000	17	11.614	6.673	9.813	0.284		2	Medium	0.032
**LF_WT**		139	158	EKLSPLGEEMRDRARAHVDA	2279.1441	3.1476	9000	17	15.576	7.3	10.735	0.672		2	Medium	0.075
**LF_WT**		140	146	KLSPLGE	742.4225	2.9112	0	4	0	0	0	n/a		2	Medium	0
**LF_WT**		140	146	KLSPLGE	742.4225	2.9083	30	4	0.035	2.015	50.375	0.384		2	Medium	0.043
**LF_WT**		140	146	KLSPLGE	742.4225	2.8882	300	4	0.334	1.788	44.704	0.355		2	Medium	0.04
**LF_WT**		140	146	KLSPLGE	742.4225	2.8947	3000	4	2.158	1.836	45.906	0.269		2	Medium	0.03
**LF_WT**		140	146	KLSPLGE	742.4225	2.875	9000	4	3.363	1.829	45.736	0.139		2	Medium	0.016
**LF_WT**		140	147	KLSPLGEE	871.4651	2.9826	0	5	0	0	0	n/a		2	High	0
**LF_WT**		140	147	KLSPLGEE	871.4651	2.9719	30	5	0.055	2.429	24.289	0.563		2	Medium	0.063
**LF_WT**		140	147	KLSPLGEE	871.4651	2.9699	300	5	0.516	2.214	22.139	0.196		2	Medium	0.022
**LF_WT**		140	147	KLSPLGEE	871.4651	2.9824	3000	5	3.025	2.257	22.568	0.504		2	Medium	0.056
**LF_WT**		140	147	KLSPLGEE	871.4651	2.9682	9000	5	4.361	2.256	22.565	0.525		2	Medium	0.058
**LF_WT**		140	148	KLSPLGEEM	1002.5056	3.7469	0	6	0	0	0	n/a		2	Medium	0
**LF_WT**		140	148	KLSPLGEEM	1002.5056	3.7224	30	6	0.067	2.896	24.135	0.41		2	Medium	0.046
**LF_WT**		140	148	KLSPLGEEM	1002.5056	3.6925	300	6	0.629	2.938	24.485	0.049		2	Medium	0.005

**LF_WT**		140	148	KLSPLGEEM	1002.5056	3.7444	3000	6	3.723	2.799	23.325	0.81	2	Medium	0.09	
**LF_WT**		140	148	KLSPLGEEM	1002.5056	3.7246	9000	6	5.333	2.805	23.372	0.229	2	Medium	0.025	
**LF_WT**		147	158	EMRDRARAHVDA	1425.6895	1.4499	0	10	0	0	0	n/a	2	Medium	0	
**LF_WT**		147	158	EMRDRARAHVDA	1425.6895	1.4264	30	10	0.181	1.033	2.582	0.219	2	Medium	0.024	
**LF_WT**		147	158	EMRDRARAHVDA	1425.6895	1.457	300	10	1.632	1.141	2.852	0.203	2	Medium	0.023	
**LF_WT**		147	158	EMRDRARAHVDA	1425.6895	1.4644	3000	10	7.626	2.043	5.107	0.321	2	Medium	0.036	
**LF_WT**		147	158	EMRDRARAHVDA	1425.6895	1.4336	9000	10	9.641	2.636	6.59	0.466	2	Medium	0.052	
**LF_WT**		147	159	EMRDRARAHVDAL	1538.7736	2.3086	0	11	0	0	0	n/a	2	Medium	0	
**LF_WT**		147	159	EMRDRARAHVDAL	1538.7736	2.3001	30	11	0.185	0.924	2.412	0.187	4	Medium	0.118	
**LF_WT**		147	159	EMRDRARAHVDAL	1538.7736	2.287	300	11	1.672	1.077	2.817	0.251	4	Medium	0.158	
**LF_WT**		147	159	EMRDRARAHVDAL	1538.7736	2.3092	3000	11	7.965	1.915	5.05	0.19	4	Medium	0.12	
**LF_WT**		147	159	EMRDRARAHVDAL	1538.7736	2.2816	9000	11	10.352	2.479	6.539	0.182	4	Medium	0.114	
**LF_WT**		147	169	EMRDRARAHVDALRTHLAPYSDE	2708.3201	3.1003	0	20	0	0	0	n/a	2	High	0	
**LF_WT**		147	169	EMRDRARAHVDALRTHLAPYSDE	2708.3201	3.1093	30	20	0.353	1.402	1.752	0.55	2	High	0.061	
**LF_WT**		147	169	EMRDRARAHVDALRTHLAPYSDE	2708.3201	3.0838	300	20	3.141	1.859	2.324	0.866	2	High	0.096	
**LF_WT**		147	169	EMRDRARAHVDALRTHLAPYSDE	2708.3201	3.1086	3000	20	14.157	3.266	4.082	1.141	2	High	0.127	
**LF_WT**		147	169	EMRDRARAHVDALRTHLAPYSDE	2708.3201	3.0756	9000	20	18.62	5.079	6.348	0.353	2	Medium	0.039	
**LF_WT**		148	158	MRDRARAHVDA	1296.6469	1.4284	0	9	0	0	0	n/a	2	Medium	0	
**LF_WT**		148	158	MRDRARAHVDA	1296.6469	1.4313	30	9	0.173	0.839	2.331	0.159	2	High	0.018	
**LF_WT**		148	158	MRDRARAHVDA	1296.6469	1.4267	300	9	1.552	0.831	2.309	0.229	2	High	0.026	
**LF_WT**		148	158	MRDRARAHVDA	1296.6469	1.4375	3000	9	7.061	1.404	3.899	0.069	2	High	0.008	
**LF_WT**		148	158	MRDRARAHVDA	1296.6469	1.436	9000	9	8.723	1.903	5.287	0.235	2	High	0.026	
**LF_WT**		148	159	MRDRARAHVDAL	1409.731	2.0388	0	10	0	0	0	n/a	2	High	0	
**LF_WT**		148	159	MRDRARAHVDAL	1409.731	2.0236	30	10	0.177	0.787	1.967	0.485	2	High	0.054	
**LF_WT**		148	159	MRDRARAHVDAL	1409.731	2.0223	300	10	1.593	0.733	1.831	0.078	2	Medium	0.009	
**LF_WT**		148	159	MRDRARAHVDAL	1409.731	2.0218	3000	10	7.4	1.298	3.246	0.354	2	Medium	0.039	
**LF_WT**		148	159	MRDRARAHVDAL	1409.731	2.0166	9000	10	9.434	1.911	4.777	0.479	2	Medium	0.053	
**LF_WT**		148	169	MRDRARAHVDALRTHLAPYSDE	2579.2775	2.8821	0	19	0	0	0	n/a	2	Medium	0	
**LF_WT**		148	169	MRDRARAHVDALRTHLAPYSDE	2579.2775	2.9072	30	19	0.345	1.08	1.314	0.521	4	Medium	0.327	
**LF_WT**		148	169	MRDRARAHVDALRTHLAPYSDE	2579.2775	2.889	300	19	3.061	1.405	1.686	0.333	4	Medium	0.209	
**LF_WT**		148	169	MRDRARAHVDALRTHLAPYSDE	2579.2775	2.896	3000	19	13.592	2.475	2.966	0.521	4	Medium	0.328	
**LF_WT**		148	169	MRDRARAHVDALRTHLAPYSDE	2579.2775	2.8682	9000	19	17.702	3.878	4.803	1.141	3	Medium	0.459	
**LF_WT**		149	158	RDRARAHVDA	1165.6065	1.4341	0	8	0	0	0	n/a	2	Medium	0	
**LF_WT**		149	158	RDRARAHVDA	1165.6065	1.3783	30	8	0.136	0.871	3.627	0.012	2	Medium	0.001	
**LF_WT**		149	158	RDRARAHVDA	1165.6065	1.3855	300	8	1.24	0.804	3.349	0.037	2	Medium	0.004	
**LF_WT**		149	158	RDRARAHVDA	1165.6065	1.3921	3000	8	6.085	1.388	5.784	0.164	2	Medium	0.018	
**LF_WT**		149	158	RDRARAHVDA	1165.6065	1.3945	9000	8	7.723	1.818	7.577	0.181	2	Medium	0.02	
**LF_WT**		155	169	HVDALRTHLAPYSDE	1722.8325	3.0439	0	12	0	0	0	n/a	2	Medium	0	
**LF_WT**		155	169	HVDALRTHLAPYSDE	1722.8325	3.046	30	12	0.216	0.989	2.748	0.447	2	Medium	0.05	
**LF_WT**		155	169	HVDALRTHLAPYSDE	1722.8325	3.0255	300	12	1.907	0.987	2.743	0.216	2	Medium	0.024	
**LF_WT**		155	169	HVDALRTHLAPYSDE	1722.8325	3.0443	3000	12	8.293	1.846	5.128	0.081	2	Medium	0.009	
**LF_WT**		155	169	HVDALRTHLAPYSDE	1722.8325	3.0482	9000	12	10.974	2.552	7.09	0.036	2	Medium	0.004	
**LF_WT**		155	170	HVDALRTHLAPYSDEL	1835.9166	3.8144	0	13	0	0	0	n/a	2	Medium	0	
**LF_WT**		155	170	HVDALRTHLAPYSDEL	1835.9166	3.7859	30	13	0.221	0.876	2.246	0.079	2	Medium	0.009	
**LF_WT**		155	170	HVDALRTHLAPYSDEL	1835.9166	3.7942	300	13	1.951	0.967	2.48	0.322	2	Medium	0.036	
**LF_WT**		155	170	HVDALRTHLAPYSDEL	1835.9166	3.7773	3000	13	8.657	1.88	4.82	0.907	2	Medium	0.101	
**LF_WT**		155	170	HVDALRTHLAPYSDEL	1835.9166	3.788	9000	13	11.716	2.625	6.731	0.15	2	Medium	0.017	
**LF_WT**		159	168	LRTHLAPYSD	1171.5986	2.6683	0	7	0	0	0	n/a	2	Medium	0	
**LF_WT**		159	168	LRTHLAPYSD	1171.5986	2.6313	30	7	0.131	0.81	5.786	0.14	2	Medium	0.016	
**LF_WT**		159	168	LRTHLAPYSD	1171.5986	2.6231	300	7	1.143	0.923	6.593	0.346	2	Medium	0.038	
**LF_WT**		159	168	LRTHLAPYSD	1171.5986	2.605	3000	7	4.818	1.995	14.251	0.103	2	Medium	0.011	
**LF_WT**		159	168	LRTHLAPYSD	1171.5986	2.6016	9000	7	6.467	2.445	17.461	0.361	2	Medium	0.04	
**LF_WT**		159	169	LRTHLAPYSDE	1300.6412	2.7779	0	8	0	0	0	n/a	2	High	0	
**LF_WT**		159	169	LRTHLAPYSDE	1300.6412	2.7114	30	8	0.163	0.686	4.288	0.214	2	High	0.024	
**LF_WT**		159	169	LRTHLAPYSDE	1300.6412	2.703	300	8	1.416	0.712	4.453	0.082	2	High	0.009	
**LF_WT**		159	169	LRTHLAPYSDE	1300.6412	2.7095	3000	8	5.777	1.353	8.457	0.052	2	High	0.006	
**LF_WT**		159	169	LRTHLAPYSDE	1300.6412	2.7102	9000	8	7.467	1.894	11.836	0.225	2	High	0.025	
**LF_WT**		159	170	LRTHLAPYSDEL	1413.7252	3.6964	0	9	0	0	0	n/a	2	High	0	
**LF_WT**		159	170	LRTHLAPYSDEL	1413.7252	3.6674	30	9	0.167	0.741	4.117	0.29	2	High	0.032	
**LF_WT**		159	170	LRTHLAPYSDEL	1413.7252	3.658	300	9	1.46	0.76	4.221	0.099	2	High	0.011	
**LF_WT**		159	170	LRTHLAPYSDEL	1413.7252	3.6629	3000	9	6.14	1.433	7.961	0.006	2	High	0.001	
**LF_WT**		159	170	LRTHLAPYSDEL	1413.7252	3.6632	9000	9	8.21	2	11.111	0.294	2	High	0.033	
**LF_WT**		159	173	LRTHLAPYSDELRQR	1853.986	3.3508	0	12	0	0	0	n/a	2	Medium	0	
**LF_WT**		159	173	LRTHLAPYSDELRQR	1853.986	3.3708	30	12	0.193	0.791	1.647	0.706	2	Medium	0.079	
**LF_WT**		159	173	LRTHLAPYSDELRQR	1853.986	3.3726	300	12	1.705	0.804	1.675	0.065	2	Medium	0.007	
**LF_WT**		159	173	LRTHLAPYSDELRQR	1853.986	3.378	3000	12	7.827	1.455	3.031	0.554	2	Medium	0.062	
**LF_WT**		159	173	LRTHLAPYSDELRQR	1853.986	3.3804	9000	12	10.913	2.053	4.276	0.01	2	Medium	0.001	

**LF_WT**		159	174	LRTHLAPYSDELRQRL	1967.0701	3.5058	0	13	0	0	0	n/a	2	High	0	
**LF_WT**		159	174	LRTHLAPYSDELRQRL	1967.0701	3.4939	30	13	0.196	0.978	1.699	0.143	4	Medium	0.09	
**LF_WT**		159	174	LRTHLAPYSDELRQRL	1967.0701	3.502	300	13	1.738	1.003	1.752	0.187	4	Medium	0.118	
**LF_WT**		159	174	LRTHLAPYSDELRQRL	1967.0701	3.504	3000	13	8.113	1.69	2.952	0.29	4	High	0.182	
**LF_WT**		159	174	LRTHLAPYSDELRQRL	1967.0701	3.4913	9000	13	11.55	2.369	4.122	0.208	4	High	0.131	
**LF_WT**		159	176	LRTHLAPYSDELRQRLAA	2109.1443	3.5246	0	15	0	0	0	n/a	2	High	0	
**LF_WT**		159	176	LRTHLAPYSDELRQRLAA	2109.1443	3.527	30	15	0.222	0.988	1.646	0.557	2	High	0.062	
**LF_WT**		159	176	LRTHLAPYSDELRQRLAA	2109.1443	3.5168	300	15	1.984	1.107	1.845	0.11	2	High	0.012	
**LF_WT**		159	176	LRTHLAPYSDELRQRLAA	2109.1443	3.5141	3000	15	9.564	1.881	3.135	0.276	2	High	0.031	
**LF_WT**		159	176	LRTHLAPYSDELRQRLAA	2109.1443	3.5002	9000	15	13.502	2.75	4.584	1.48	2	Medium	0.165	
**LF_WT**		159	178	LRTHLAPYSDELRQRLAARL	2378.3295	3.9968	0	17	0	0	0	n/a	2	High	0	
**LF_WT**		159	178	LRTHLAPYSDELRQRLAARL	2378.3295	3.9789	30	17	0.234	0.879	0.955	0.185	4	Medium	0.117	
**LF_WT**		159	178	LRTHLAPYSDELRQRLAARL	2378.3295	3.9739	300	17	2.096	1.14	1.229	0.025	4	High	0.016	
**LF_WT**		159	178	LRTHLAPYSDELRQRLAARL	2378.3295	3.9722	3000	17	10.407	1.921	2.07	0.128	4	High	0.08	
**LF_WT**		159	178	LRTHLAPYSDELRQRLAARL	2378.3295	3.9897	9000	17	15.052	2.57	2.766	0.141	4	High	0.088	
**LF_WT**		159	180	LRTHLAPYSDELRQRLAARLEA	2578.4092	4.0667	0	19	0	0	0	n/a	2	Medium	0	
**LF_WT**		159	180	LRTHLAPYSDELRQRLAARLEA	2578.4092	4.0687	30	19	0.257	0.691	0.824	0.09	4	Medium	0.056	
**LF_WT**		159	180	LRTHLAPYSDELRQRLAARLEA	2578.4092	4.0434	300	19	2.317	1.031	1.229	0.129	4	Medium	0.081	
**LF_WT**		159	180	LRTHLAPYSDELRQRLAARLEA	2578.4092	4.0474	3000	19	11.715	1.843	2.205	0.372	4	Medium	0.234	
**LF_WT**		159	180	LRTHLAPYSDELRQRLAARLEA	2578.4092	4.0623	9000	19	16.914	2.732	3.264	0.733	4	Medium	0.461	
**LF_WT**		160	169	RTHLAPYSDE	1187.5571	2.3738	0	7	0	0	0	n/a	2	High	0	
**LF_WT**		160	169	RTHLAPYSDE	1187.5571	2.381	30	7	0.156	0.596	4.257	0.029	2	Medium	0.003	
**LF_WT**		160	169	RTHLAPYSDE	1187.5571	2.3665	300	7	1.347	0.624	4.46	0.097	2	High	0.011	
**LF_WT**		160	169	RTHLAPYSDE	1187.5571	2.3704	3000	7	5.264	1.217	8.691	0.175	2	High	0.02	
**LF_WT**		160	169	RTHLAPYSDE	1187.5571	2.3709	9000	7	6.583	1.662	11.874	0.289	2	High	0.032	
**LF_WT**		160	174	RTHLAPYSDELRQRL	1853.986	3.3508	0	12	0	0	0	n/a	2	Medium	0	
**LF_WT**		160	174	RTHLAPYSDELRQRL	1853.986	3.3708	30	12	0.189	0.792	1.649	0.706	2	Medium	0.079	
**LF_WT**		160	174	RTHLAPYSDELRQRL	1853.986	3.3726	300	12	1.668	0.805	1.677	0.065	2	Medium	0.007	
**LF_WT**		160	174	RTHLAPYSDELRQRL	1853.986	3.374	3000	12	7.6	1.472	3.068	0.341	2	Medium	0.038	
**LF_WT**		160	174	RTHLAPYSDELRQRL	1853.986	3.3804	9000	12	10.665	2.054	4.278	0.01	2	Medium	0.001	
**LF_WT**		170	176	LRQRLAA	826.5137	1.7726	0	5	0	0	0	n/a	2	High	0	
**LF_WT**		170	176	LRQRLAA	826.5137	1.7926	30	5	0.05	0.552	5.524	0.123	2	Medium	0.014	
**LF_WT**		170	176	LRQRLAA	826.5137	1.7753	300	5	0.472	0.432	4.317	0.055	2	Medium	0.006	
**LF_WT**		170	176	LRQRLAA	826.5137	1.7664	3000	5	3.008	0.518	5.181	0.123	2	Medium	0.014	
**LF_WT**		170	176	LRQRLAA	826.5137	1.8067	9000	5	4.492	0.6	5.997	0.176	2	High	0.02	
**LF_WT**		170	177	LRQRLAAR	982.6148	2.2317	0	6	0	0	0	n/a	2	High	0	
**LF_WT**		170	177	LRQRLAAR	982.6148	2.2062	30	6	0.058	0.576	3.201	0.135	2	High	0.015	
**LF_WT**		170	177	LRQRLAAR	982.6148	2.1993	300	6	0.55	0.476	2.646	0.036	2	High	0.004	
**LF_WT**		170	177	LRQRLAAR	982.6148	2.2057	3000	6	3.565	0.581	3.227	0.027	2	High	0.003	
**LF_WT**		170	177	LRQRLAAR	982.6148	2.1771	9000	6	5.405	0.728	4.046	0.078	2	High	0.009	
**LF_WT**		170	178	LRQRLAARL	1095.6989	2.4685	0	7	0	0	0	n/a	2	High	0	
**LF_WT**		170	178	LRQRLAARL	1095.6989	2.4584	30	7	0.061	0.638	3.039	0.218	2	High	0.024	
**LF_WT**		170	178	LRQRLAARL	1095.6989	2.4464	300	7	0.583	0.539	2.568	0.091	2	High	0.01	
**LF_WT**		170	178	LRQRLAARL	1095.6989	2.4567	3000	7	3.851	0.635	3.024	0.045	2	High	0.005	
**LF_WT**		170	178	LRQRLAARL	1095.6989	2.4673	9000	7	6.042	0.79	3.761	0.071	2	High	0.008	
**LF_WT**		170	180	LRQRLAARLEA	1295.7786	2.5198	0	9	0	0	0	n/a	2	High	0	
**LF_WT**		170	180	LRQRLAARLEA	1295.7786	2.4991	30	9	0.085	0.641	2.087	0.045	4	High	0.028	
**LF_WT**		170	180	LRQRLAARLEA	1295.7786	2.4848	300	9	0.804	0.563	1.833	0.032	4	High	0.02	
**LF_WT**		170	180	LRQRLAARLEA	1295.7786	2.4964	3000	9	5.159	0.734	2.387	0.042	4	High	0.027	
**LF_WT**		170	180	LRQRLAARLEA	1295.7786	2.5043	9000	9	7.904	0.961	3.114	0.042	4	High	0.026	
**LF_WT**		171	178	RQRLAARL	982.6148	2.2317	0	6	0	0	0	n/a	2	High	0	
**LF_WT**		171	178	RQRLAARL	982.6148	2.2062	30	6	0.051	0.576	3.201	0.135	2	High	0.015	
**LF_WT**		171	178	RQRLAARL	982.6148	2.1993	300	6	0.485	0.476	2.646	0.036	2	High	0.004	
**LF_WT**		171	178	RQRLAARL	982.6148	2.2057	3000	6	3.21	0.581	3.227	0.027	2	High	0.003	
**LF_WT**		171	178	RQRLAARL	982.6148	2.1771	9000	6	5.088	0.728	4.046	0.078	2	High	0.009	
**LF_WT**		171	180	RQRLAARLEA	1182.6945	2.2546	0	8	0	0	0	n/a	2	High	0	
**LF_WT**		171	180	RQRLAARLEA	1182.6945	2.2533	30	8	0.074	0.66	2.749	0.251	2	High	0.028	
**LF_WT**		171	180	RQRLAARLEA	1182.6945	2.2169	300	8	0.707	0.609	2.537	0.416	2	High	0.046	
**LF_WT**		171	180	RQRLAARLEA	1182.6945	2.2528	3000	8	4.517	0.752	3.133	0.115	2	High	0.013	
**LF_WT**		171	180	RQRLAARLEA	1182.6945	2.2597	9000	8	6.95	1.016	4.235	0.318	2	High	0.035	
**LF_WT**		179	189	EALKENGGARL	1156.62	1.9827	0	9	0	0	0	n/a	2	High	0	
**LF_WT**		179	189	EALKENGGARL	1156.62	1.9837	30	9	0.121	4.833	26.851	0.286	2	High	0.032	
**LF_WT**		179	189	EALKENGGARL	1156.62	1.9765	300	9	1.106	4.297	23.871	0.23	2	High	0.026	
**LF_WT**		179	189	EALKENGGARL	1156.62	1.9772	3000	9	5.692	4.434	24.635	0.288	2	High	0.032	
**LF_WT**		179	189	EALKENGGARL	1156.62	1.9704	9000	9	7.965	4.442	24.676	0.39	2	High	0.043	
**LF_WT**		179	191	EALKENGGARLAE	1356.6997	2.265	0	11	0	0	0	n/a	2	Medium	0	
**LF_WT**		179	191	EALKENGGARLAE	1356.6997	2.2084	30	11	0.142	5.709	17.3	0.772	2	Medium	0.086	
**LF_WT**		179	191	EALKENGGARLAE	1356.6997	2.2284	300	11	1.308	5.882	17.823	1.265	2	Medium	0.141	
**LF_WT**		179	191	EALKENGGARLAE	1356.6997	2.2232	3000	11	7.002	5.757	17.444	1.394	2	Medium	0.155	
**LF_WT**		179	191	EALKENGGARLAE	1356.6997	2.2175	9000	11	9.883	5.703	17.282	0.706	2	Medium	0.079	
**LF_WT**		179	203	EALKENGGARLAEYHAKATEHLSTL	2708.3882	3.2805	0	23	0	0	0	n/a	2	High	0	

**LF_WT**		179	203	EALKENGGARLAEYHAKATEHLSTL	2708.3882	3.2239	30	23	0.348	11.964	17.339	0.69	2	Medium	0.077	
**LF_WT**		179	203	EALKENGGARLAEYHAKATEHLSTL	2708.3882	3.1985	300	23	3.083	11.394	16.513	0.821	2	High	0.091	
**LF_WT**		179	203	EALKENGGARLAEYHAKATEHLSTL	2708.3882	3.2041	3000	23	14.85	11.618	16.838	2.151	2	High	0.239	
**LF_WT**		179	203	EALKENGGARLAEYHAKATEHLSTL	2708.3882	3.2312	9000	23	20.808	11.264	16.325	1.514	2	High	0.168	
**LF_WT**		181	190	LKENGGARLA	1027.5774	1.8726	0	8	0	0	0	n/a	2	Medium	0	
**LF_WT**		181	190	LKENGGARLA	1027.5774	1.8648	30	8	0.123	4.286	22.262	0.126	4	Medium	0.079	
**LF_WT**		181	190	LKENGGARLA	1027.5774	1.8378	300	8	1.122	3.877	20.117	0.214	4	Medium	0.134	
**LF_WT**		181	190	LKENGGARLA	1027.5774	1.8876	3000	8	5.638	3.888	20.127	0.245	4	Medium	0.154	
**LF_WT**		181	190	LKENGGARLA	1027.5774	1.8895	9000	8	7.456	3.88	20.136	0.171	4	Medium	0.107	
**LF_WT**		181	191	LKENGGARLAE	1156.62	1.9937	0	9	0	0	0	n/a	2	High	0	
**LF_WT**		181	191	LKENGGARLAE	1156.62	1.989	30	9	0.133	4.998	18.511	0.971	2	Medium	0.108	
**LF_WT**		181	191	LKENGGARLAE	1156.62	1.9778	300	9	1.221	4.443	16.454	0.527	2	Medium	0.059	
**LF_WT**		181	191	LKENGGARLAE	1156.62	1.9862	3000	9	6.285	4.519	16.736	0.335	2	Medium	0.037	
**LF_WT**		181	191	LKENGGARLAE	1156.62	1.9833	9000	9	8.412	4.514	16.719	0.315	2	Medium	0.035	
**LF_WT**		190	195	AEYHAK	717.3446	1.3954	0	4	0	0	0	n/a	2	Medium	0	
**LF_WT**		190	195	AEYHAK	717.3446	1.5106	30	4	0.08	2.286	28.576	0.157	2	Medium	0.017	
**LF_WT**		190	195	AEYHAK	717.3446	1.4218	300	4	0.707	1.848	23.096	0.234	2	Medium	0.026	
**LF_WT**		190	195	AEYHAK	717.3446	1.3828	3000	4	2.992	1.891	23.639	0.091	2	Medium	0.01	
**LF_WT**		190	195	AEYHAK	717.3446	1.3949	9000	4	3.795	1.97	24.63	0.817	2	Medium	0.091	
**LF_WT**		190	200	AEYHAKATEHL	1268.6149	2.0555	0	9	0	0	0	n/a	2	High	0	
**LF_WT**		190	200	AEYHAKATEHL	1268.6149	2.0405	30	9	0.186	4.314	15.977	0.414	2	Medium	0.046	
**LF_WT**		190	200	AEYHAKATEHL	1268.6149	2.0464	300	9	1.577	3.865	14.315	0.241	2	Medium	0.027	
**LF_WT**		190	200	AEYHAKATEHL	1268.6149	2.0145	3000	9	6.422	4.025	14.907	0.346	2	Medium	0.038	
**LF_WT**		190	200	AEYHAKATEHL	1268.6149	1.9782	9000	9	8.488	4.203	15.568	0.737	2	Medium	0.082	
**LF_WT**		190	201	AEYHAKATEHLS	1355.647	1.6607	0	10	0	0	0	n/a	2	Medium	0	
**LF_WT**		190	201	AEYHAKATEHLS	1355.647	1.6756	30	10	0.195	4.627	23.134	0.81	2	Medium	0.09	
**LF_WT**		190	201	AEYHAKATEHLS	1355.647	1.6793	300	10	1.667	4.195	20.974	0.807	2	High	0.09	
**LF_WT**		190	201	AEYHAKATEHLS	1355.647	1.6886	3000	10	7.032	4.224	21.119	1.22	2	High	0.136	
**LF_WT**		190	201	AEYHAKATEHLS	1355.647	1.666	9000	10	9.428	4.327	21.636	0.999	2	High	0.111	
**LF_WT**		190	202	AEYHAKATEHLST	1456.6947	1.9737	0	11	0	0	0	n/a	2	High	0	
**LF_WT**		190	202	AEYHAKATEHLST	1456.6947	1.8991	30	11	0.203	5.415	24.612	0.696	2	High	0.077	
**LF_WT**		190	202	AEYHAKATEHLST	1456.6947	1.8448	300	11	1.747	5.047	22.94	0.38	2	High	0.042	
**LF_WT**		190	202	AEYHAKATEHLST	1456.6947	1.8866	3000	11	7.598	5.111	23.23	0.313	2	High	0.035	
**LF_WT**		190	202	AEYHAKATEHLST	1456.6947	1.8799	9000	11	10.347	5.127	23.303	0.702	2	High	0.078	
**LF_WT**		190	203	AEYHAKATEHLSTL	1569.7787	2.7512	0	12	0	0	0	n/a	2	High	0	
**LF_WT**		190	203	AEYHAKATEHLSTL	1569.7787	2.719	30	12	0.206	5.755	23.98	0.667	2	High	0.074	
**LF_WT**		190	203	AEYHAKATEHLSTL	1569.7787	2.7361	300	12	1.775	5.235	21.813	0.554	2	High	0.062	
**LF_WT**		190	203	AEYHAKATEHLSTL	1569.7787	2.7438	3000	12	7.848	5.337	22.238	0.038	2	High	0.004	
**LF_WT**		190	203	AEYHAKATEHLSTL	1569.7787	2.7433	9000	12	10.925	5.351	22.296	0.48	2	High	0.053	
**LF_WT**		190	205	AEYHAKATEHLSTLSE	1785.8533	2.5578	0	14	0	0	0	n/a	2	High	0	
**LF_WT**		190	205	AEYHAKATEHLSTLSE	1785.8533	2.5347	30	14	0.232	7.119	25.424	1.368	2	High	0.152	
**LF_WT**		190	205	AEYHAKATEHLSTLSE	1785.8533	2.5434	300	14	2.016	6.442	23.007	0.512	2	High	0.057	
**LF_WT**		190	205	AEYHAKATEHLSTLSE	1785.8533	2.5316	3000	14	9.263	6.583	23.511	0.412	2	High	0.046	
**LF_WT**		190	205	AEYHAKATEHLSTLSE	1785.8533	2.5493	9000	14	12.858	6.578	23.494	0.53	2	High	0.059	
**LF_WT**		190	211	AEYHAKATEHLSTLSEKAKPAL	2394.2543	2.6988	0	19	0	0	0	n/a	2	High	0	
**LF_WT**		190	211	AEYHAKATEHLSTLSEKAKPAL	2394.2543	2.6247	30	19	0.275	9.645	12.691	2.16	2	High	0.24	
**LF_WT**		190	211	AEYHAKATEHLSTLSEKAKPAL	2394.2543	2.6251	300	19	2.434	8.685	11.427	0.914	2	High	0.102	
**LF_WT**		190	211	AEYHAKATEHLSTLSEKAKPAL	2394.2543	2.6303	3000	19	12.095	8.849	11.644	0.01	2	High	0.001	
**LF_WT**		190	211	AEYHAKATEHLSTLSEKAKPAL	2394.2543	2.6311	9000	19	17.33	8.866	11.666	1.272	2	High	0.142	
**LF_WT**		190	212	AEYHAKATEHLSTLSEKAKPALE	2523.2969	2.5883	0	20	0	0	0	n/a	2	High	0	
**LF_WT**		190	212	AEYHAKATEHLSTLSEKAKPALE	2523.2969	2.5322	30	20	0.282	10.364	17.273	0.329	2	Medium	0.037	
**LF_WT**		190	212	AEYHAKATEHLSTLSEKAKPALE	2523.2969	2.5523	300	20	2.5	9.158	15.263	0.978	2	High	0.109	
**LF_WT**		190	212	AEYHAKATEHLSTLSEKAKPALE	2523.2969	2.5576	3000	20	12.586	9.389	15.648	0.239	2	High	0.027	
**LF_WT**		190	212	AEYHAKATEHLSTLSEKAKPALE	2523.2969	2.5544	9000	20	18.199	9.352	15.587	0.785	2	High	0.087	
**LF_WT**		190	213	AEYHAKATEHLSTLSEKAKPALED	2638.3239	2.6147	0	21	0	0	0	n/a	2	High	0	
**LF_WT**		190	213	AEYHAKATEHLSTLSEKAKPALED	2638.3239	2.5527	30	21	0.335	10.937	17.36	0.631	2	High	0.07	
**LF_WT**		190	213	AEYHAKATEHLSTLSEKAKPALED	2638.3239	2.5741	300	21	2.917	9.652	15.32	1.335	2	High	0.149	
**LF_WT**		190	213	AEYHAKATEHLSTLSEKAKPALED	2638.3239	2.5819	3000	21	13.582	9.878	15.68	0.118	2	High	0.013	
**LF_WT**		190	213	AEYHAKATEHLSTLSEKAKPALED	2638.3239	2.5875	9000	21	19.199	9.831	15.605	0.828	2	High	0.092	
**LF_WT**		190	214	AEYHAKATEHLSTLSEKAKPALEDL	2751.4079	3.4887	0	22	0	0	0	n/a	2	High	0	
**LF_WT**		190	214	AEYHAKATEHLSTLSEKAKPALEDL	2751.4079	3.4419	30	22	0.342	11.273	13.702	0.988	4	High	0.621	
**LF_WT**		190	214	AEYHAKATEHLSTLSEKAKPALEDL	2751.4079	3.4527	300	22	2.986	10.098	12.263	0.178	4	High	0.112	
**LF_WT**		190	214	AEYHAKATEHLSTLSEKAKPALEDL	2751.4079	3.4729	3000	22	14.093	10.261	12.451	0.094	4	Medium	0.059	
**LF_WT**		190	214	AEYHAKATEHLSTLSEKAKPALEDL	2751.4079	3.4528	9000	22	20.082	10.314	12.534	0.276	4	High	0.173	
**LF_WT**		190	219	AEYHAKATEHLSTLSEKAKPALEDLRQGLL	3318.7572	4.1109	0	27	0	0	0	n/a	2	High	0	
**LF_WT**		190	219	AEYHAKATEHLSTLSEKAKPALEDLRQGLL	3318.7572	4.0549	30	27	0.377	14.037	12.997	1.326	2	High	0.148	
**LF_WT**		190	219	AEYHAKATEHLSTLSEKAKPALEDLRQGLL	3318.7572	4.061	300	27	3.326	12.943	11.985	1.261	2	High	0.14	

**LF_WT**		190	219	AEYHAKATEHLSTLSEKAKPALEDLRQGLL	3318.7572	4.0694	3000	27	16.525	13.171	12.195	0.287	2	High	0.032	
**LF_WT**		190	219	AEYHAKATEHLSTLSEKAKPALEDLRQGLL	3318.7572	4.0705	9000	27	24.236	13.146	12.172	1.454	2	High	0.162	
**LF_WT**		190	222	AEYHAKATEHLSTLSEKAKPALEDLRQGLLPV	3627.9624	4.6208	0	29	0	0	0	n/a	2	Medium	0	
**LF_WT**		190	222	AEYHAKATEHLSTLSEKAKPALEDLRQGLLPV	3627.9624	4.5763	30	29	0.381	14.691	14.597	0.717	3	Medium	0.289	
**LF_WT**		190	222	AEYHAKATEHLSTLSEKAKPALEDLRQGLLPV	3627.9624	4.5867	300	29	3.366	14.119	12.98	0.107	4	Medium	0.067	
**LF_WT**		190	222	AEYHAKATEHLSTLSEKAKPALEDLRQGLLPV	3627.9624	4.5653	3000	29	16.898	14.477	13.256	0.513	4	Medium	0.323	
**LF_WT**		190	222	AEYHAKATEHLSTLSEKAKPALEDLRQGLLPV	3627.9624	4.6133	9000	29	25.155	14.395	12.092	0.587	3	Medium	0.236	
**LF_WT**		192	203	YHAKATEHLSTL	1369.699	2.5985	0	10	0	0	0	n/a	2	Medium	0	
**LF_WT**		192	203	YHAKATEHLSTL	1369.699	2.6145	30	10	0.166	4.616	15.387	0.897	2	Medium	0.1	
**LF_WT**		192	203	YHAKATEHLSTL	1369.699	2.574	300	10	1.421	4.279	14.265	0.794	2	Medium	0.088	
**LF_WT**		192	203	YHAKATEHLSTL	1369.699	2.5935	3000	10	6.328	4.307	14.355	0.009	2	Medium	0.001	
**LF_WT**		192	203	YHAKATEHLSTL	1369.699	2.6016	9000	10	9.01	4.339	14.464	0.308	2	Medium	0.034	
**LF_WT**		192	211	YHAKATEHLSTLSEKAKPAL	2194.1746	2.5703	0	17	0	0	0	n/a	2	High	0	
**LF_WT**		192	211	YHAKATEHLSTLSEKAKPAL	2194.1746	2.5383	30	17	0.235	8.375	16.422	0.156	2	Medium	0.017	
**LF_WT**		192	211	YHAKATEHLSTLSEKAKPAL	2194.1746	2.5304	300	17	2.08	7.704	15.106	1.155	2	High	0.129	
**LF_WT**		192	211	YHAKATEHLSTLSEKAKPAL	2194.1746	2.5268	3000	17	10.575	7.821	15.336	0.505	2	High	0.056	
**LF_WT**		192	211	YHAKATEHLSTLSEKAKPAL	2194.1746	2.5269	9000	17	15.416	7.805	15.305	1.199	2	High	0.133	
**LF_WT**		193	211	HAKATEHLSTLSEKAKPAL	2031.1113	2.4238	0	16	0	0	0	n/a	2	High	0	
**LF_WT**		193	211	HAKATEHLSTLSEKAKPAL	2031.1113	2.386	30	16	0.203	8.152	13.616	0.284	4	Medium	0.178	
**LF_WT**		193	211	HAKATEHLSTLSEKAKPAL	2031.1113	2.3823	300	16	1.796	7.416	12.392	0.232	4	Medium	0.146	
**LF_WT**		193	211	HAKATEHLSTLSEKAKPAL	2031.1113	2.3799	3000	16	9.611	7.479	12.516	0.226	4	Medium	0.142	
**LF_WT**		193	211	HAKATEHLSTLSEKAKPAL	2031.1113	2.4134	9000	16	14.416	7.424	12.38	0.158	4	Medium	0.1	
**LF_WT**		199	211	HLSTLSEKAKPAL	1393.7929	2.5485	0	10	0	0	0	n/a	2	Medium	0	
**LF_WT**		199	211	HLSTLSEKAKPAL	1393.7929	2.5193	30	10	0.09	5.584	18.612	0.447	2	Medium	0.05	
**LF_WT**		199	211	HLSTLSEKAKPAL	1393.7929	2.4972	300	10	0.857	5.105	17.017	0.376	2	Medium	0.042	
**LF_WT**		199	211	HLSTLSEKAKPAL	1393.7929	2.5191	3000	10	5.673	5.159	17.197	0.161	2	Medium	0.018	
**LF_WT**		199	211	HLSTLSEKAKPAL	1393.7929	2.5004	9000	10	8.843	5.153	17.176	0.378	2	Medium	0.042	
**LF_WT**		201	212	STLSEKAKPALE	1272.6925	2.3327	0	9	0	0	0	n/a	2	Medium	0	
**LF_WT**		201	212	STLSEKAKPALE	1272.6925	2.2996	30	9	0.079	5.731	31.838	0.556	2	Medium	0.062	
**LF_WT**		201	212	STLSEKAKPALE	1272.6925	2.2594	300	9	0.753	5.505	30.585	0.046	2	Medium	0.005	
**LF_WT**		201	212	STLSEKAKPALE	1272.6925	2.2697	3000	9	4.989	5.487	30.484	0.267	2	Medium	0.03	
**LF_WT**		201	212	STLSEKAKPALE	1272.6925	2.2819	9000	9	7.852	5.479	30.441	0.072	2	Medium	0.008	
**LF_WT**		201	219	STLSEKAKPALEDLRQGLL	2068.1528	4.4083	0	16	0	0	0	n/a	2	Medium	0	
**LF_WT**		201	219	STLSEKAKPALEDLRQGLL	2068.1528	4.4488	30	16	0.174	9.601	20.001	1.616	2	Medium	0.18	
**LF_WT**		201	219	STLSEKAKPALEDLRQGLL	2068.1528	4.4114	300	16	1.579	9.447	19.681	0.592	2	Medium	0.066	
**LF_WT**		201	219	STLSEKAKPALEDLRQGLL	2068.1528	4.3806	3000	16	8.928	9.666	20.138	0.138	2	Medium	0.015	
**LF_WT**		201	219	STLSEKAKPALEDLRQGLL	2068.1528	4.3959	9000	16	13.889	9.718	20.246	0.832	2	Medium	0.093	
**LF_WT**		203	212	LSEKAKPALE	1084.6128	1.9478	0	7	0	0	0	n/a	2	High	0	
**LF_WT**		203	212	LSEKAKPALE	1084.6128	1.9325	30	7	0.067	4.209	30.061	0.23	2	High	0.026	
**LF_WT**		203	212	LSEKAKPALE	1084.6128	1.8833	300	7	0.635	3.853	27.524	0.445	2	High	0.05	
**LF_WT**		203	212	LSEKAKPALE	1084.6128	1.9137	3000	7	4.129	3.9	27.854	0.107	2	High	0.012	
**LF_WT**		203	212	LSEKAKPALE	1084.6128	1.9138	9000	7	6.334	3.911	27.936	0.003	2	High	0	
**LF_WT**		203	213	LSEKAKPALED	1199.6398	2.0472	0	8	0	0	0	n/a	2	High	0	
**LF_WT**		203	213	LSEKAKPALED	1199.6398	2.0329	30	8	0.119	4.721	29.507	0.506	2	High	0.056	
**LF_WT**		203	213	LSEKAKPALED	1199.6398	2.0049	300	8	1.052	4.294	26.838	0.287	2	High	0.032	
**LF_WT**		203	213	LSEKAKPALED	1199.6398	2.0414	3000	8	5.125	4.32	27.002	0.045	2	High	0.005	
**LF_WT**		203	213	LSEKAKPALED	1199.6398	2.0439	9000	8	7.334	4.318	26.99	0.386	2	High	0.043	
**LF_WT**		203	214	LSEKAKPALEDL	1312.7238	3.6207	0	9	0	0	0	n/a	2	High	0	
**LF_WT**		203	214	LSEKAKPALEDL	1312.7238	3.5982	30	9	0.126	5.024	27.91	0.032	2	Medium	0.004	
**LF_WT**		203	214	LSEKAKPALEDL	1312.7238	3.6058	300	9	1.121	4.689	26.05	0.451	2	High	0.05	
**LF_WT**		203	214	LSEKAKPALEDL	1312.7238	3.617	3000	9	5.636	4.786	26.588	0.004	2	High	0	
**LF_WT**		203	214	LSEKAKPALEDL	1312.7238	3.6263	9000	9	8.217	4.788	26.599	0.2	2	High	0.022	
**LF_WT**		203	219	LSEKAKPALEDLRQGLL	1880.0731	4.231	0	14	0	0	0	n/a	2	High	0	
**LF_WT**		203	219	LSEKAKPALEDLRQGLL	1880.0731	4.2203	30	14	0.162	8.288	29.601	1.732	2	Medium	0.193	
**LF_WT**		203	219	LSEKAKPALEDLRQGLL	1880.0731	4.2048	300	14	1.461	7.808	27.885	1.643	2	Medium	0.183	
**LF_WT**		203	219	LSEKAKPALEDLRQGLL	1880.0731	4.2044	3000	14	8.068	8.022	28.648	0.198	2	Medium	0.022	
**LF_WT**		203	219	LSEKAKPALEDLRQGLL	1880.0731	4.2068	9000	14	12.371	8.03	28.679	1.097	2	Medium	0.122	
**LF_WT**		203	222	LSEKAKPALEDLRQGLLPVL	2189.2784	4.9463	0	16	0	0	0	n/a	2	Medium	0	
**LF_WT**		203	222	LSEKAKPALEDLRQGLLPVL	2189.2784	4.9285	30	16	0.166	9.553	19.902	1.505	2	Medium	0.168	
**LF_WT**		203	222	LSEKAKPALEDLRQGLLPVL	2189.2784	4.9274	300	16	1.501	9.272	19.316	0.9	2	Medium	0.1	
**LF_WT**		203	222	LSEKAKPALEDLRQGLLPVL	2189.2784	4.9279	3000	16	8.441	9.468	19.725	0.135	2	Medium	0.015	
**LF_WT**		203	222	LSEKAKPALEDLRQGLLPVL	2189.2784	4.9321	9000	16	13.29	9.488	19.766	0.902	2	Medium	0.1	
**LF_WT**		204	211	SEKAKPAL	842.4862	1.8345	0	5	0	0	0	n/a	2	High	0	
**LF_WT**		204	211	SEKAKPAL	842.4862	1.8016	30	5	0.044	3.112	31.117	0.314	2	High	0.035	
**LF_WT**		204	211	SEKAKPAL	842.4862	1.7559	300	5	0.418	2.857	28.569	0.372	2	High	0.041	
**LF_WT**		204	211	SEKAKPAL	842.4862	1.826	3000	5	2.832	2.826	28.264	0.036	2	High	0.004	
**LF_WT**		204	211	SEKAKPAL	842.4862	1.7743	9000	5	4.473	2.894	28.937	0.077	2	High	0.009	
**LF_WT**		204	212	SEKAKPALE	971.5288	1.5799	0	6	0	0	0	n/a	2	High	0	
**LF_WT**		204	212	SEKAKPALE	971.5288	1.5609	30	6	0.05	3.668	30.563	0.754	2	High	0.084	
**LF_WT**		204	212	SEKAKPALE	971.5288	1.5704	300	6	0.483	3.295	27.456	0.486	2	High	0.054	

**LF_WT**			204	212	SEKAKPALE	971.5288	1.6188	3000	6	3.323	3.33	27.752	0.315	2	High	0.035	
**LF_WT**			204	212	SEKAKPALE	971.5288	1.5749	9000	6	5.341	3.357	27.979	0.311	2	High	0.035	
**LF_WT**			204	213	SEKAKPALED	1086.5557	1.7339	0	7	0	0	0	n/a	2	High	0	
**LF_WT**			204	213	SEKAKPALED	1086.5557	1.7296	30	7	0.103	4.141	29.577	0.511	2	High	0.057	
**LF_WT**			204	213	SEKAKPALED	1086.5557	1.7179	300	7	0.901	3.717	26.551	0.037	2	High	0.004	
**LF_WT**			204	213	SEKAKPALED	1086.5557	1.7255	3000	7	4.319	3.816	27.254	0.188	2	High	0.021	
**LF_WT**			204	213	SEKAKPALED	1086.5557	1.727	9000	7	6.341	3.815	27.251	0.29	2	High	0.032	
**LF_WT**			204	214	SEKAKPALEDL	1199.6398	3.4859	0	8	0	0	0	n/a	2	High	0	
**LF_WT**			204	214	SEKAKPALEDL	1199.6398	3.4851	30	8	0.11	4.739	29.618	0.545	2	High	0.061	
**LF_WT**			204	214	SEKAKPALEDL	1199.6398	3.4745	300	8	0.97	4.293	26.833	0.593	2	High	0.066	
**LF_WT**			204	214	SEKAKPALEDL	1199.6398	3.4782	3000	8	4.829	4.37	27.313	0.212	2	High	0.024	
**LF_WT**			204	214	SEKAKPALEDL	1199.6398	3.4807	9000	8	7.224	4.374	27.336	0.648	2	High	0.072	
**LF_WT**			204	219	SEKAKPALEDLRQGLL	1766.989	4.1526	0	13	0	0	0	n/a	2	High	0	
**LF_WT**			204	219	SEKAKPALEDLRQGLL	1766.989	4.1381	30	13	0.145	8.434	32.439	0.31	2	Medium	0.034	
**LF_WT**			204	219	SEKAKPALEDLRQGLL	1766.989	4.0799	300	13	1.309	7.817	30.064	0.263	2	Medium	0.029	
**LF_WT**			204	219	SEKAKPALEDLRQGLL	1766.989	4.0543	3000	13	7.262	7.727	29.718	0.909	2	Medium	0.101	
**LF_WT**			204	219	SEKAKPALEDLRQGLL	1766.989	4.1285	9000	13	11.378	7.711	29.657	0.004	2	Medium	0	
**LF_WT**			204	222	SEKAKPALEDLRQGLLPVL	2076.1943	4.8907	0	15	0	0	0	n/a	2	Medium	0	
**LF_WT**			204	222	SEKAKPALEDLRQGLLPVL	2076.1943	4.8773	30	15	0.149	9.14	25.406	0.364	4	Medium	0.229	
**LF_WT**			204	222	SEKAKPALEDLRQGLLPVL	2076.1943	4.8686	300	15	1.35	8.815	24.512	0.201	4	Medium	0.126	
**LF_WT**			204	222	SEKAKPALEDLRQGLLPVL	2076.1943	4.8729	3000	15	7.635	8.999	25.02	0.075	4	Medium	0.047	
**LF_WT**			204	222	SEKAKPALEDLRQGLLPVL	2076.1943	4.8619	9000	15	12.297	9.121	25.308	0.166	4	Medium	0.104	
**LF_WT**			206	222	KAKPALEDLRQGLLPVL	1860.1197	4.8341	0	13	0	0	0	n/a	2	Medium	0	
**LF_WT**			206	222	KAKPALEDLRQGLLPVL	1860.1197	4.8136	30	13	0.127	8.175	26.17	0.378	4	Medium	0.237	
**LF_WT**			206	222	KAKPALEDLRQGLLPVL	1860.1197	4.8311	300	13	1.132	7.739	24.837	0.167	4	Medium	0.105	
**LF_WT**			206	222	KAKPALEDLRQGLLPVL	1860.1197	4.8507	3000	13	6.266	7.886	25.359	0.24	4	Medium	0.151	
**LF_WT**			206	222	KAKPALEDLRQGLLPVL	1860.1197	4.8313	9000	13	10.36	7.963	25.552	0.34	4	Medium	0.214	
**LF_WT**			212	219	EDLRQGLL	942.5134	4.0452	0	6	0	0	0	n/a	2	High	0	
**LF_WT**			212	219	EDLRQGLL	942.5134	4.0415	30	6	0.042	3.588	44.914	0.136	4	Medium	0.086	
**LF_WT**			212	219	EDLRQGLL	942.5134	4.037	300	6	0.408	3.436	43.009	0.081	4	Medium	0.051	
**LF_WT**			212	219	EDLRQGLL	942.5134	4.041	3000	6	2.944	3.534	44.201	0.028	4	Medium	0.018	
**LF_WT**			212	219	EDLRQGLL	942.5134	4.0408	9000	6	5.037	3.559	44.471	0.051	4	Medium	0.032	
**LF_WT**			212	222	EDLRQGLLPVL	1251.7187	5.1795	0	8	0	0	0	n/a	2	High	0	
**LF_WT**			212	222	EDLRQGLLPVL	1251.7187	5.1852	30	8	0.046	4.81	30.062	0.083	2	High	0.009	
**LF_WT**			212	222	EDLRQGLLPVL	1251.7187	5.1302	300	8	0.449	4.866	30.41	0.079	2	High	0.009	
**LF_WT**			212	222	EDLRQGLLPVL	1251.7187	5.1781	3000	8	3.316	4.95	30.94	0.013	2	High	0.001	
**LF_WT**			212	222	EDLRQGLLPVL	1251.7187	5.1342	9000	8	5.956	5.007	31.297	0.187	2	High	0.021	
**LF_WT**			213	219	DLRQGLL	813.4709	4.1694	0	5	0	0	0	n/a	2	High	0	
**LF_WT**			213	219	DLRQGLL	813.4709	4.152	30	5	0.035	3.056	61.124	0.193	2	High	0.022	
**LF_WT**			213	219	DLRQGLL	813.4709	4.1203	300	5	0.339	2.959	59.175	0.001	2	High	0	
**LF_WT**			213	219	DLRQGLL	813.4709	4.1476	3000	5	2.433	3	60.001	0.035	2	High	0.004	
**LF_WT**			213	219	DLRQGLL	813.4709	4.1474	9000	5	4.154	3.008	60.162	0.294	2	High	0.033	
**LF_WT**			213	222	DLRQGLLPVL	1122.6761	5.3036	0	7	0	0	0	n/a	2	High	0	
**LF_WT**			213	222	DLRQGLLPVL	1122.6761	5.2938	30	7	0.039	4.321	30.865	0.291	2	High	0.032	
**LF_WT**			213	222	DLRQGLLPVL	1122.6761	5.2788	300	7	0.38	4.298	30.698	0.458	2	High	0.051	
**LF_WT**			213	222	DLRQGLLPVL	1122.6761	5.2833	3000	7	2.805	4.42	31.57	0.06	2	High	0.007	
**LF_WT**			213	222	DLRQGLLPVL	1122.6761	5.2482	9000	7	5.073	4.511	32.219	0.298	2	High	0.033	
**LF_WT**			214	222	LRQGLLPVL	1007.6492	4.944	0	6	0	0	0	n/a	2	Medium	0	
**LF_WT**			214	222	LRQGLLPVL	1007.6492	4.9666	30	6	0.034	3.662	45.623	0.143	4	Medium	0.09	
**LF_WT**			214	222	LRQGLLPVL	1007.6492	4.957	300	6	0.328	3.634	45.224	0.116	4	Medium	0.073	
**LF_WT**			214	222	LRQGLLPVL	1007.6492	4.9685	3000	6	2.39	3.734	46.448	0.099	4	Medium	0.062	
**LF_WT**			214	222	LRQGLLPVL	1007.6492	4.9579	9000	6	4.273	3.795	47.342	0.066	4	Medium	0.042	
**LF_WT**			215	222	RQGLLPVL	894.5651	4.8242	0	5	0	0	0	n/a	2	High	0	
**LF_WT**			215	222	RQGLLPVL	894.5651	4.7472	30	5	0.024	3.054	30.543	0.465	2	High	0.052	
**LF_WT**			215	222	RQGLLPVL	894.5651	4.7112	300	5	0.23	3.093	30.933	0.018	2	High	0.002	
**LF_WT**			215	222	RQGLLPVL	894.5651	4.7638	3000	5	1.748	3.158	31.576	0.053	2	High	0.006	
**LF_WT**			215	222	RQGLLPVL	894.5651	4.7616	9000	5	3.319	3.182	31.821	0.433	2	High	0.048	
**LF_WT**			223	229	ESFKVSF	842.4174	4.4663	0	5	0	0	0	n/a	2	Medium	0	
**LF_WT**			223	229	ESFKVSF	842.4174	4.4476	30	5	0.03	3.246	64.924	0.092	2	Medium	0.01	
**LF_WT**			223	229	ESFKVSF	842.4174	4.4583	300	5	0.295	3.055	61.099	0.08	2	Medium	0.009	
**LF_WT**			223	229	ESFKVSF	842.4174	4.4743	3000	5	2.211	3.149	62.971	0.433	2	Medium	0.048	
**LF_WT**			223	229	ESFKVSF	842.4174	4.4858	9000	5	3.967	3.16	63.191	0.793	2	Medium	0.088	
**LF_WT**			226	232	KVSFLSA	750.4276	3.8693	0	5	0	0	0	n/a	2	Medium	0	
**LF_WT**			226	232	KVSFLSA	750.4276	3.8676	30	5	0.041	2.818	56.355	0.289	2	High	0.032	
**LF_WT**			226	232	KVSFLSA	750.4276	3.8565	300	5	0.393	2.818	56.361	0.437	2	High	0.049	
**LF_WT**			226	232	KVSFLSA	750.4276	3.8434	3000	5	2.659	2.929	58.571	0.126	2	High	0.014	
**LF_WT**			226	232	KVSFLSA	750.4276	3.857	9000	5	4.246	2.934	58.69	0.005	2	High	0.001	
**LF_WT**			230	235	LSALEE	660.333	3.0243	0	4	0	0	0	n/a	2	High	0	
**LF_WT**			230	235	LSALEE	660.333	3.0329	30	4	0.044	2.299	57.475	0.033	2	Medium	0.004	
**LF_WT**			230	235	LSALEE	660.333	3.028	300	4	0.414	2.329	58.216	0.334	2	Medium	0.037	
**LF_WT**			230	235	LSALEE	660.333	2.9772	3000	4	2.434	2.476	61.912	0.157	2	Medium	0.017	
**LF_WT**			230	235	LSALEE	660.333	3.0159	9000	4	3.559	2.495	62.364	0.392	2	Medium	0.044	
**LF_WT**			233	243	LEEYTKKLNTQ	1365.714	2.3221	0	9	0	0	0	n/a	2	Medium	0	
**LF_WT**			233	243	LEEYTKKLNTQ	1365.714	2.3072	30	9	0.072	5.144	38.361	0.357	3	Medium	0.144	
**LF_WT**			233	243	LEEYTKKLNTQ	1365.714	2.3148	300	9	0.686	4.992	41.966	0.259	4	Medium	0.162	
**LF_WT**			233	243	LEEYTKKLNTQ	1365.714	2.3202	3000	9	4.527	5.037	42.126	0.198	4	High	0.125	
**LF_WT**			233	243	LEEYTKKLNTQ	1365.714	2.2978	9000	9	7.358	5.205	43.95	0.616	4	Medium	0.387	
**LF_WT**			234	243	EEYTKKLNTQ	1252.6299	1.8991	0	8	0	0	0	n/a	2	High	0	
**LF_WT**			234	243	EEYTKKLNTQ	1252.6299	1.8655	30	8	0.052	5.087	63.587	0.875	2	High	0.097	
**LF_WT**			234	243	EEYTKKLNTQ	1252.6299	1.8487	300	8	0.503	4.805	60.059	0.546	2	High	0.061	
**LF_WT**			234	243	EEYTKKLNTQ	1252.6299	1.839	3000	8	3.661	4.959	61.992	0.375	2	High	0.042	
**LF_WT**			234	243	EEYTKKLNTQ	1252.6299	1.8583	9000	8	6.361	4.943	61.789	0.217	2	High	0.024	
**LF_WT**			236	243	YTKKLNTQ	994.5447	1.4271	0	6	0	0	0	n/a	2	High	0	
**LF_WT**			236	243	YTKKLNTQ	994.5447	1.4263	30	6	0.039	3.637	60.625	0.609	2	High	0.068	
**LF_WT**			236	243	YTKKLNTQ	994.5447	1.4228	300	6	0.375	3.42	57.003	0.242	2	High	0.027	
**LF_WT**			236	243	YTKKLNTQ	994.5447	1.431	3000	6	2.705	3.464	57.732	0.037	2	High	0.004	
**LF_WT**			236	243	YTKKLNTQ	994.5447	1.4281	9000	6	4.666	3.493	58.211	0.274	2	High	0.031	
